# Kinetic Overview of Polynuclear Platinum(II) Complexes in Heparan Sulfate Substitution: Mimetic Model Analysis and Mechanistic Perspectives

**DOI:** 10.1021/acsomega.5c13472

**Published:** 2026-04-28

**Authors:** Frederico Henrique do C. R. Ferreira, Nicholas P. Farrell, Luiz Antônio S. Costa

**Affiliations:** † NEQC − Núcleo de Estudos em Química Computacional, Departamento de Química, Instituto de Ciências Exatas, Universidade Federal de Juiz de Fora, Rua José Lourenço Kelmer, S/N, Campus Universitário, Juiz de Fora, Minas Gerais 36036-900, Brazil; ‡ Department of Chemistry, 6889 Virginia Commonwealth University, Richmond, Virginia 23284−2006, United States

## Abstract

Polynuclear platinum­(II) complexes (PPCs) represent a promising class of anticancer agents, offering enhanced efficacy and reduced toxicity compared with traditional mononuclear platinum drugs. Their interactions extend beyond DNA to include noncovalent and covalent binding with biologically relevant anions, such as sulfates and carboxylates, in glycosaminoglycans (GAGs) like heparan sulfate (HS) and chondroitin sulfate. This study investigates the chloride substitution mechanisms in PPCs using a Cl–PtN3 model, evaluating both aquation-driven and direct substitution pathways with GAG mimetic models of iduronic acid, IdoA­(2S), and glucose, GlcNS­(6S). A computational benchmarking analysis identified the double-hybrid DFT functional B2PLYP as the most accurate method, displaying an absolute deviation of only 1.05 kcal mol^–1^ from the DLPNO–CCSD­(T) reference. Free energy profiles revealed similar energy transition-state species ranging from ca. 26 to 30 kcal mol^–1^, while direct substitutions exhibit lower activation barriers but are thermodynamically less favorable. The inclusion of explicit water molecules in the solvation layer was also addressed and significantly drove the results toward experimental data, with a better description of the active complex. These findings, added to our microkinetic analysis, provide insight into PPC ligand-exchange mechanisms, contributing to the rational design of next-generation platinum-based anticancer therapeutics.

## Graphical abstract

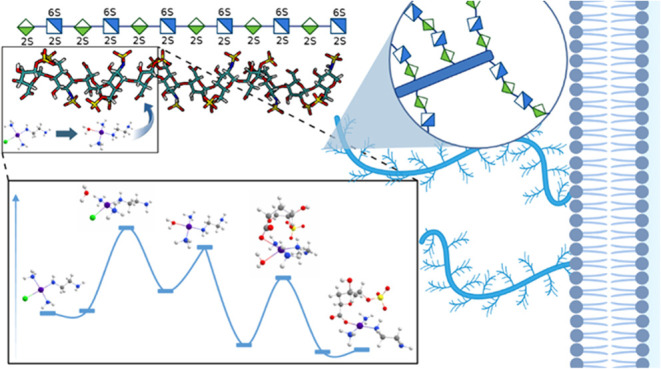

## 1 Introduction

Cancer is estimated as either the first or the second greatest cause of premature deaths in 112 out of 183 countries worldwide, and the third or fourth in 23 others.^1^ Among the therapeutic approaches against this disease, chemotherapy is one of the most well-known and can be employed alone or with another method simultaneously. Cisplatin, formally cis-diamminodichloroplatinum (II) (Figure 1a), was first synthesized by Peyrone in 1845 ^2–4^ and had its anticancer properties described by Rosenberg in 1965.^5–8^ In 1978, the FDA approved it for certain cancer treatments, making it the first platinum drug authorized for cancer chemotherapy. After the success of cisplatin, some direct analogues were developed and approved worldwide for clinical administration, such as oxaliplatin (Figure 1b) and carboplatin (Figure 1c). Nedaplatin (Figure 1d) and lobaplatin (Figure 1e) were also approved in certain countries.^9^ The mechanism of action of these compounds is considered to rely, in short, in the double aquation of the complex once it passes through the membrane toward the DNA, followed by a direct substitution of the aqua ligands to form covalent adducts with guanines and cytosines in DNA, leading to blockage in DNA transcription, inhibition of replication, and activation of cellular apoptosis mechanisms.^10–12^

**Figure 1:**
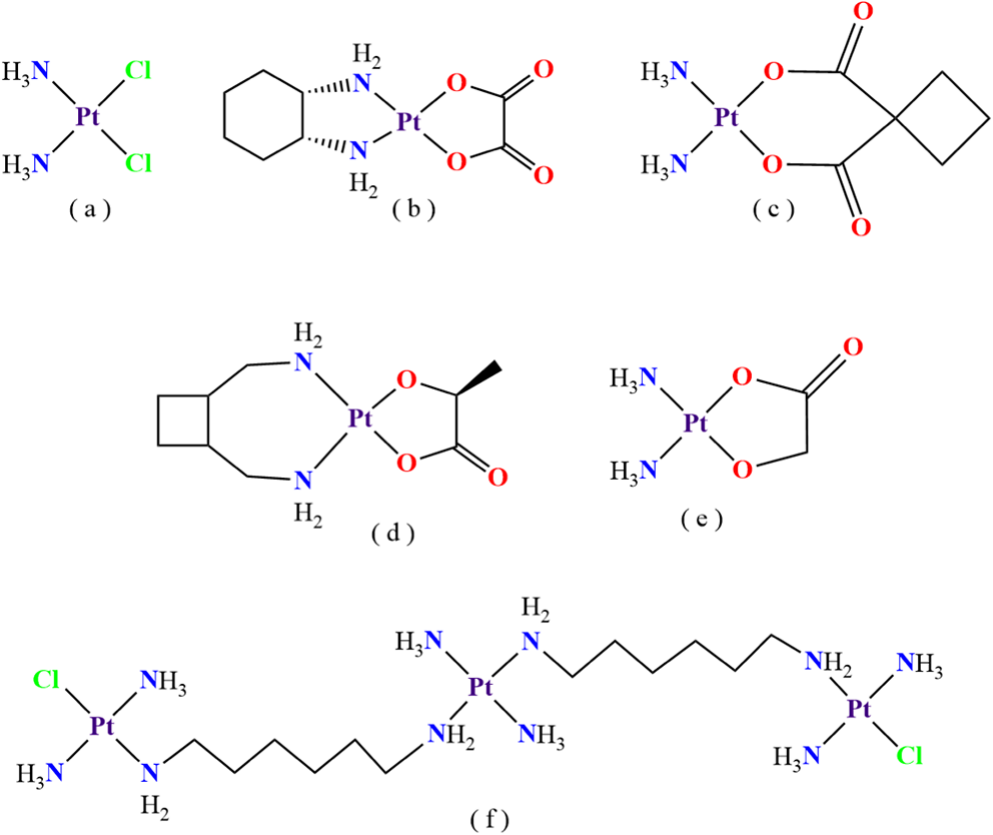
Platinum compounds administered as chemotherapy agents: (a) cisplatin, (b) oxaliplatin, (c) carboplatin, (d) lobaplatin, and (e) nedaplatin. The compound (f), Triplatin (BBR3464), was the first nonanalogue of cisplatin to reach phase 2 in clinical trials.

Many different approaches have been pursued so far, aiming at the improvement of antineoplastic effects, including, among others, ligand structure modifications,^13^ multimetallic compounds,^14^ coordination polymer complexes^15^ and drug combinations.^16^ Triplatin (BBR3464) was the first cisplatin nonanalogue to enter human clinical trials.^17,18^ Triplatin is part of a structurally distinct class of compounds named polynuclear platinum complexes (PPCs), which comprise a series of PtN centers linked through diamine chains, as shown in Figure 1f.^19,20^ Triplatin forms covalent DNA adducts, especially long-range {Pt,Pt} interstrand cross-links, which are structurally distinct from those formed by cisplatin and its congeners. Furthermore, the central Pt­(tetramine) center donates hydrogen bonds to the phosphates in the structure of DNA and may dictate the structural preferences of the final DNA adducts.^21,22^

More recently, the importance of glycosaminoglycans (GAGs), such as heparan sulfate (HS), in the action of these PPCs has been described.^23^ GAGs such as HS have several important biological roles, i.e., angiogenesis, cellular migration, recognition, adhesion, differentiation, and proliferation. Considering their importance, this biomolecule has emerged as a novel target for a cancer antimetastatic chemotherapy drug.^24^ For PPCs, we can consider binding to GAGs to be a two-step process, like DNA binding, involving molecular recognition through H-bonding to the DNA or sugar backbone, followed by covalent “fixation” to N-donors on DNA bases or to the O-donors of the polysaccharide.

Platinum-O-donor interactions have not been as extensively studied as those to “soft” bases such as N­(DNA, RNA) and S­(protein) donors.^25^ The aquation process of Triplatin has been studied by {1H,15N HSQC} NMR spectroscopy.^26^ Zhang and coworkers also used NMR to obtain kinetic and thermodynamic parameters for acetate and phosphate interactions of PPC compounds, including Triplatin.^27^ Sulfate interactions have also been probed employing the same methodology to compare with acetate and phosphate interactions.^28^ More recently, the interaction of Triplatin with monosaccharides^29^ and site-specific substituted disaccharides GlcNS­(6S)-GlcA and GlcNS­(6S)-IdoA­(2S) has been reported.^30^ Theoretically, our group has already studied the interaction between PPCs and biomolecules through molecular dynamics simulations to investigate the formation of cyclic interactions.^31–34^

Building on the established experimental foundation of PPC reactivity with O-donors, particularly in relation to GAGs, this study provides a detailed mechanistic approach to the substitution reactions using density functional theory (DFT). Our objective is to offer an atomic-level perspective of the process to enhance future understanding of the importance of Pt–O-donor interactions in the cellular biology of platinum anticancer agents.^35^

## 2 Methodology

In order to simulate the systems and represent the biological reactions that can occur with the heparan sulfate proteoglycan sugar rings, mimetic models were modeled based on the heparin structure available on the PDB under the code 1HPN.^36^ That structure is composed of alternating units of iduronic acid (IdoA (2S)) and glucose (GlcNS­(6S)) and is usually employed as a model for heparan sulfate due to its structural similarities. Figure 2 represents the HS structure available on the PDB and the systematic build of the monosaccharide models, completing the carbon valences with hydrogen atoms.

**Figure 2:**
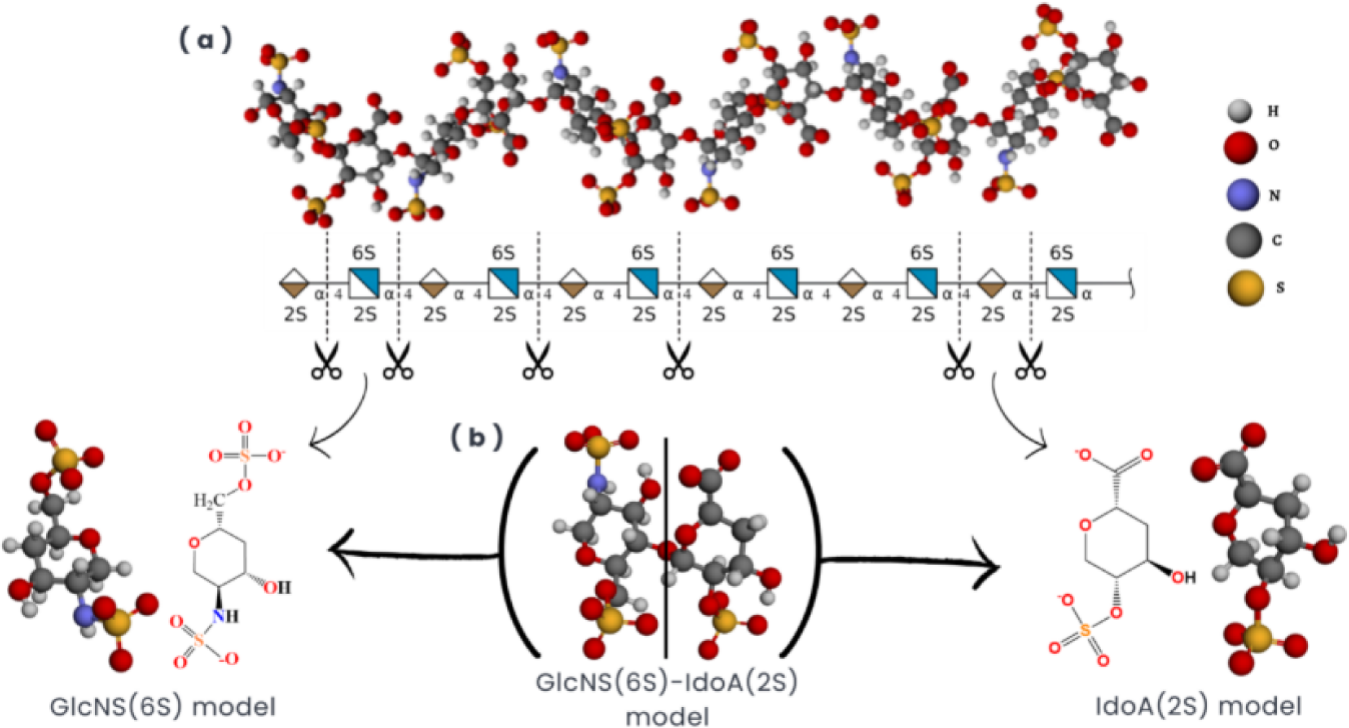
Representation of heparin employed to build the molecules. (a) Illustration of the structure of heparin available in the PDB under the code 1HPN, with its monosaccharide sequence in (b). The models used are displayed at the bottom with their respective adopted nomenclature. The valence after the cut has been completed with hydrogen atoms. Model structures differ from those of monosaccharides by two hydroxyl groups, which are involved in glycosidic bonds in heparin’s macrostructure.

All structures were obtained employing optimization calculations in the DFT^37,38^ functional BHandH,^39^ along with 6-31+G­(d,p) Pople’s basis set^40^ for light atoms and SDD^41^ for platinum. The optimized geometries were verified as true minima on the potential energy surface by performing harmonic frequency calculations at the same level of theory. The transition states were verified by the presence of a single imaginary vibrational frequency in the harmonic frequency calculations, indicating that they are first-order saddle points on the potential energy surface. All calculations were performed including the polarized continuum model IEF-PCM (water)^42^ and were carried out in the Gaussian 09 D1 software package (G09)^43^ unless indicated otherwise in the manuscript. To test the influence of some modifications in the computational protocol during the calculations of the smallest mimetic system, some alternative methodologies, which were not implemented in G09, were appraised, such as novel DFT functionals and wave function-based methods; for those, the software package ORCA 5.0.4 ^44–46^ was adopted. All ORCA methodologies and results are indicated with a respective subscript and were implemented using the CPCM solvation model.^47,48^

Single-point energy calculations were performed at different levels of theory (at the same basis set) in order to evaluate the influence of the DFT functional in the Born–Oppenheimer energy (*E*_BO_) of the chemical process of interest, which is here defined as the sum of the electronic energy (*E*_el_) and nuclear repulsion (*V*_NN_) terms, as shown in eq 1:

1 $$E_{BO_{g}}=E_{el_{g}}+V_{NN_{g}}$$

Among the computational methods evaluated in the smallest system, there are DFT functionals such as BHandH_G09_, wB97XD_G09_,^49^ M06_G09_,^50^ PBE0_G09_,^51,52^ B3LYP_G09_,^53^ CAM-B3LYP_G09_,^54^ B2PLYP_G09_,^55^ SOGGA11X_G09_,^56^ r2SCAN_ORCA_,^57^ wB97M-V_ORCA_,^58^ and the wave function-based method employed as reference DLPNO–CCSD­(T)_ORCA_,^59,60^ which demonstrated an energy deviation of only 0.5 kcal mol^–1^ in relation to CCSD­(T).^61^ This last method, with the basis set def2-TZVPP^62^ and auxiliary basis set def2-TZVPP/C,^63^ which speeds up the calculations at the cost of a small error. These methodologies selected for single-point calculations were among the best-performing DFT functionals for reaction barriers, based on a work conducted by Mardirossian and Head-Gordon.^64^

In order to properly represent the species in solution, the thermodynamic standard state was changed from 1 atm to 1 mol L^–1^ and the final free energy in the solution phase was calculated as a sum of the Born–Oppenheimer energy (*E*_BO_), the solvation contribution to the free energy, the thermal correction to the energy (*G*_n_), which refers to nuclear contributions (translational, vibrational, and rotational motions), and the energy variation, which describes the standard state change (1.89 kcal mol^–1^). The terms are displayed according to eq 2 below.

2 $$G_{\mathrm{sol}}=E_{BO_{g}}+\Delta G_{\mathrm{solv}}+G_{n}+1.89\;\mathrm{kcal}\;mol^{-1}$$

Also, enthalpies for the reactions were calculated to evaluate the entropy contribution for the reaction steps of interest when contrasting the energies with their respective free energies. For this, eq 3 demonstrates the enthalpy in solution (*H*_sol_) calculation as a sum of the Born–Oppenheimer energy in the gas phase $\left(E_{BO_{g}}\right)$ , the solvation energy (Δ*H*_solv_), and the thermal correction to the enthalpy (*H*_n_).

3 $$H_{\mathrm{sol}}=E_{BO_{g}}+\Delta H_{\mathrm{solv}}+H_{n}$$

In the thermodynamic properties (*G*_sol_ *and* *H*_sol_), both thermal corrections (*G*_n_ and *H*_n_) were calculated at the same level as the optimization procedure, BHandH/6-31+G­(d,p)-SDD­(Pt), by harmonic frequency calculations at 298.15 K (25 °C) and 1 atm. Additionally, CHELPG atomic charges were computed for 35 points along the reaction path, encompassing 17 points both preceding and following the transition state.

All steps of the reactions were assigned with its respective velocity, *k* (eq 4), and equilibrium constants, *K*_eq_ (eq 5), according to the Gibbs free energy of the step. In eqs 4 and 5, *k*_b_ is the Boltzmann constant, *T* is the temperature considered as 25 °C unless stated otherwise in the manuscript, *h* is the Planck constant, and *R* is the universal gas constant. For the equilibrium constant Δ*G* indicates the solution free energy for the reaction step, while in the rate constant equation, Δ*G* ^‡^ indicates the solution activation energy for the reaction to proceed.

4 $$k\left(t\right)=\frac{k_{b}T}{h}e^{-\Delta G^{‡}/RT}$$

5 $$K_{\mathrm{eq}}=e^{-\frac{\Delta G}{RT}}$$

To properly describe the reaction energetics, we employed a hybrid solvation model in which the first solvation shell of water molecules is treated explicitly to capture anisotropic solvent effects, while a PCM continuum accounts for the bulk contribution. The number of explicit water molecules included in the DFT calculations was determined from molecular dynamics simulations of the frozen chloride/water transition state and then applied consistently to all related systems. A full description of the hybrid solvation protocol and the MD simulations is provided in the Supporting Information.

Microkinetic modeling was performed with KINTECUS software package,^65^ which integrates all kinetic laws and simulates species concentrations over the reaction time.

### 2.1 Molecular Dynamics Simulation

To predict the ideal number of explicit water molecules to be present in the hybrid solvation model (*n* shown in eq 7), molecular dynamics calculations were performed, comprising the aquation step in the AMBER 24 suite.^66,67^ The metallic structures were parametrized with GAFF2 force-field parameters^68,69^ and the metal-related values were extracted from the DFT harmonic frequency approach BHandH/6–31+G­(d,p)/SDD­(Pt) with VFFDT toolkit^70^ which employs the Seminario method to calculate force constants relative to bond lengths (r_eq_) and angles (θ_eq_).^71^ A population analysis was also performed on the optimized structure of the complexes to obtain the atomic ChelPG charges in the HF level of theory, along with 6-31+G­(d,p) basis set and SDD pseudopotential for platinum. Nonbound Lennard-Jones parameters of platinum atoms were described according to Yao and collaborators’ work.^72^ A 10 Å radius truncated-octahedron water box was constructed around the solute, modeled according to TIP3P parameters^73^ and the system’s net charge was neutralized using chloride anions. The simulations encompassed ∼11,500 atoms, varying according to the size of the ligands.

Simulation protocol starts with a two-step minimization. Initially, 1000 cycles of the steepest descent method were performed, followed by 1500 cycles using conjugate gradients. During the first minimization phase, a restraint force constant of 500 kcal mol^–1^ Å^–2^ was applied to the solute. In contrast, the second minimization phase was carried out without restraints, enabling the entire system to minimize. Then, 6 alternating heating-equilibrium steps were performed, each increasing the temperature of the system by 50 K under constant volume periodic boundaries (NVT) and equilibrating under constant pressure periodic boundaries (NPT, *p* = 1 bar). The heating procedure was carried out in 2800 cycles (dt = 2 fs), and the equilibrium phase was 5000 frames long (dt = 2 fs). The molecular dynamics simulations were 200 ns long (dt = 2 fs) in the NPT ensemble, with a mean temperature of 300 K and 1 bar, using the Langevin thermostat and Berendsen barostat.^74,75^ The SHAKE algorithm^76^ was turned on to restrain hydrogen stretching.

## 3 Results and Discussion

First, we present the results of a brief benchmarking study to evaluate the most suitable computational protocol for this study (Section 3.1). We understand that the Cl-PtN3 chemical model can represent Triplatin quite well, so we have established this model (Figure 3) to be studied for different mechanisms. Section 3.2 demonstrates the reaction mechanism and free energy profiles through a first aquation step (3.2.1), which better represents the system experimentally studied, as mentioned in the introduction. The direct chloride substitution for a covalent adduct to the monosaccharide is also analyzed (3.2.2), since it is a relevant and viable mechanism for GAG expression on the surface of the cell, where salt concentrations are expected to inhibit the aquation process. This section has been separated by the substitution target for the sake of clarity. Section 3.3 comprises a kinectic analysis based on the aquation-driven reactions and Section 3.4 discusses the influence of the inclusion of explicit water molecules on the thermodynamics. Finally, Sections 3.5 and 3.6 present a kinectic analysis employing the hybrid solvation method and a microkinetic analysis derived from the reported free energies, respectively.

**Figure 3:**
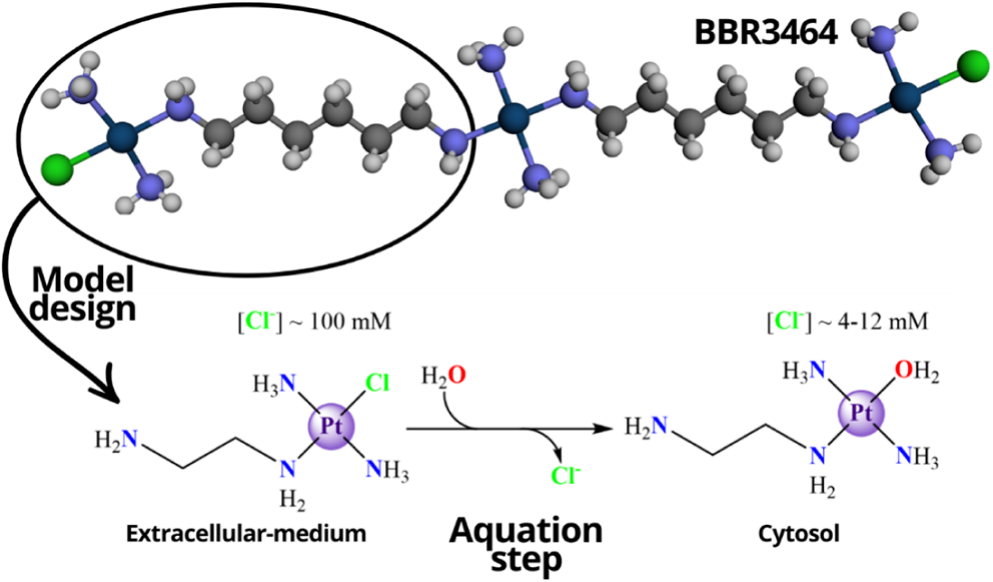
The Cl-PtN3 model from BBR3464 (Triplatin) and the aquation process.

### 3.1 Benchmark Evaluation

A brief DFT functional benchmarking was performed based on the aqueous-driven substitution reaction between Cl-PtN3 and the iduronic acid IdoA­(2S) model. We performed a series of single-point calculations with different functionals to compare them with DLPNO–CCSD­(T), which is employed as the reference in this work.^33,34^ The Born–Oppenheimer energies were calculated according to eq 1 for each different method, and the Gibbs free energy in solution was obtained according to eq 2. All equations and detailed methodologies are displayed in Supporting Information.

Table S1 displays the energy results for the reaction of interest, and Table S2 demonstrates the deviation from the reference [DLPNO-CCSD­(T)-PCM/def2-TZVPP//BHandH-PCM/6-31+G­(d,p)]. The steps will be further discussed in the next section. First, here, a comparison of the energy fluctuation as a function of DFT functionals along with 6-31+G­(d,p)-SDD­(Pt) is done. The first energy evaluated was the Gibbs free energy in solution employing the DFT functional BHandH itself; however, the performance of this methodology was not satisfactory for the calculation of electronic structure. BHandH demonstrated a correlation to the reference in the aquation step with an absolute deviation of ca. 5.9 kcal mol^–1^; moreover, it performed unsuitably even in the subsequent saccharide substitution. The overall mean absolute deviation (MAD) was approximately 13.0 kcal mol^–1^. Its performance was almost twice as poor as that of wb97XD, in which the MAD was 7.1 kcal mol^–1^ from DLPNO–CCSD­(T). The Hartree–Fock method performed similarly to the previous DFT functional (MAD of ca. 6.6 kcal mol^–1^); both presented quite similar behavior with lower deviations in the aquation step (ca. 1.4–2.1 kcal mol^–1^) and worse performance in the description of the latter process. B3LYP, which is a very popular functional among experimentalists, displayed an overall reference MAD of 3.7 kcal mol^–1^ (only 0.8 kcal mol^–1^ in the aquation step), followed by wb97M-V and M06 with MADs of 2.4 and 2.3 kcal mol^–1^, respectively. Both functionals performed a little better in the Cl^–^/H_2_O substitution process (wb97M-V: 3.0 kcal mol^–1^; M06:2.1 kcal mol^–1^) than they did in the saccharide substitution step (wb97M-V: 4.2 kcal mol^–1^; M06:4.9 kcal mol^–1^). However, the energy contrast between the processes fades as the overall deviations get lower.

Functionals r2SCAN and CAM-B3LYP displayed similar behaviors, with deviations of 1.8 and 1.7 kcal mol^–1^, respectively. CAM-B3LYP had an improvement of 118% in comparison to B3LYP, which means the deviation from DLPNO-CCSD­(T) was lower than half. The energy profiles of those functionals were close to those from PBE0 (overall deviation of 1.3 kcal mol^–1^) and SOGGA11X (MAD of 1.2 kcal mol^–1^).

The most accurate DFT functional tested in this work was B2PLYP, a double-hybrid DFT functional that achieved a MAD of only 1.0 kcal mol^–1^. Although computationally more expensive than the other methods employed in this study, B2PLYP remains orders of magnitude cheaper than CCSD­(T) with DLPNO approximations and, thus, was selected as a reference method for the subsequent reaction evaluation.

### 3.2 Reaction Mechanism and Free-Energy Profile

The mechanisms and free energy profiles of each reaction were assessed. All aqueous-driven reactions start with a common aquation step (Section 3.2.1, Scheme 1) and then the aquated complex undergoes a H_2_O/saccharide substitution that can occur in different positions in the structure of the sugar models (Section 3.2.1.1, Section 3.2.1.2). In Section 3.2.1.1, the reaction with the IdoA (2S) model was evaluated, which can occur either at the carboxylate (CO) or the sulfate (SO) motif (Scheme 2, Figure 6), whereas the GlcNS (6S) model (Section 3.2.1.2) can react either at the sulfate (SO) or the sulfonate (NS) groups (Scheme 3, Figure 7). It is also possible to study the direct substitution reaction for both sugar models at the same motifs (Section 3.2.2.1 and Section 3.2.2.2, Scheme 4 and Scheme 5, Figures 8 and 9).

**Scheme 1:**
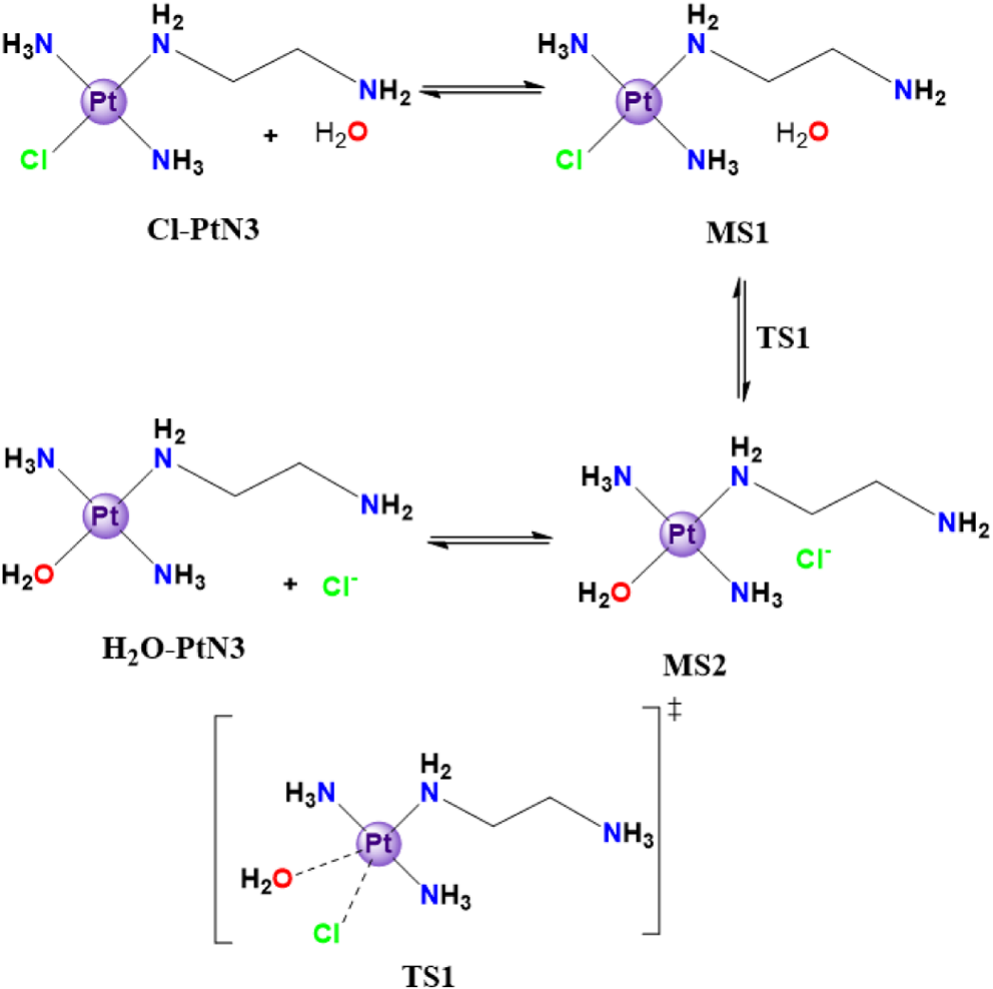
Cl^–^/H_2_O Substitution in the Structure of the Cl-PtN3 Model^a^

a MS1 indicates the cluster formed by the interaction of a water molecule. TS1 indicates the ligand exchange (Cl^–^/H_2_O) leading to MS2. H_2_O-PtN3 indicates the product molecule infinitely separated.

**Scheme 2:**
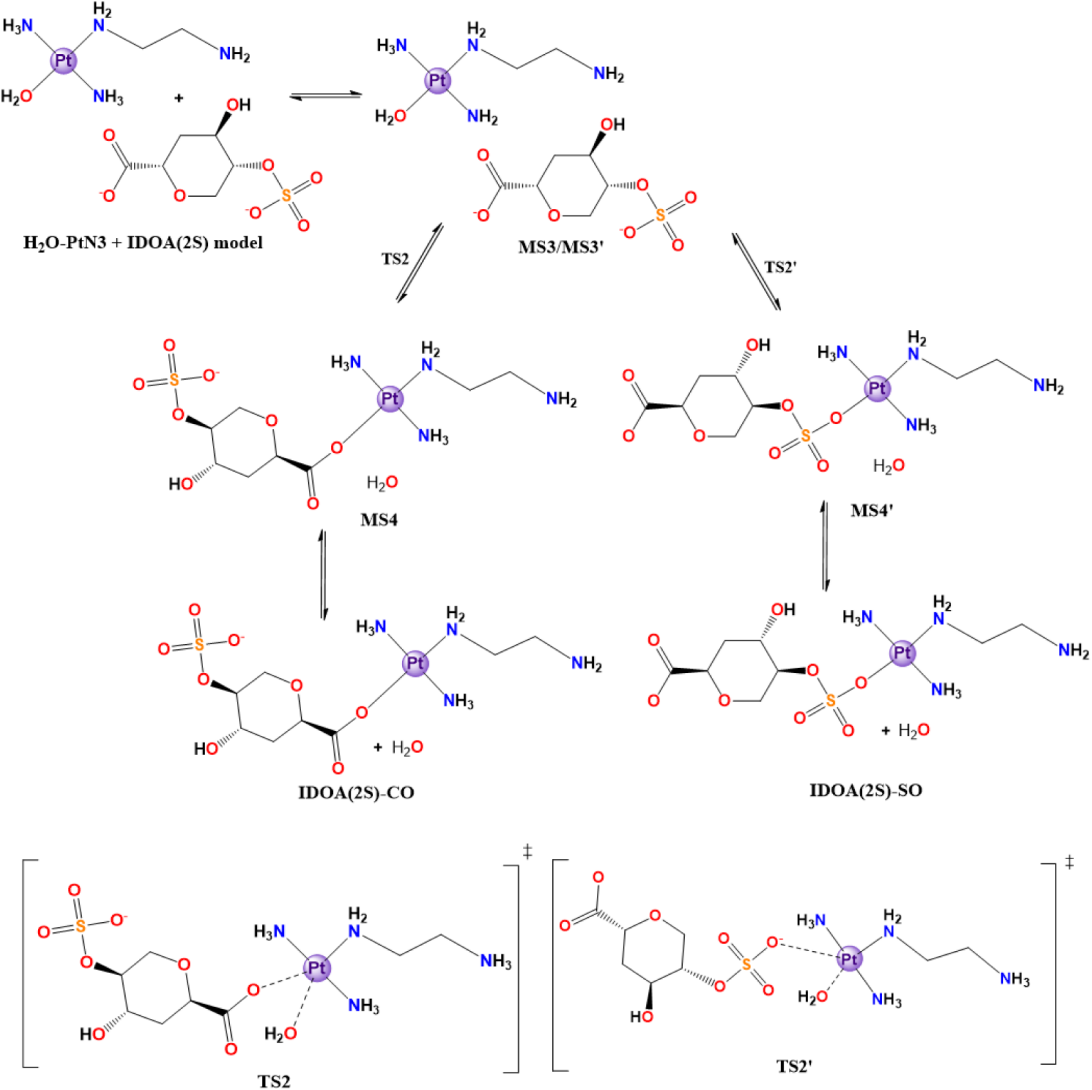
H_2_O/IdoA­(2S)-Model Substitution in the Structure of the H_2_O-PtN3 Model[Fn sch2-fn2]

a MS3/MS3′ indicates the molecular complex between the species in their respective effective pose for each transition state. TS2 indicates the square-based pyramid active species comprising the COO^–^ group of sugar, whereas TS2’ refers to the same active species, but with the RSO_3_^–^ motif. MS4/MS4’ indicates the respective products as the molecular complex of IdoA­(2S)-PtN3 and the molecule of water, and the last line demonstrates each product: carboxylate-coordinated (CO) or sulfate-coordinated (SO).

**Scheme 3:**
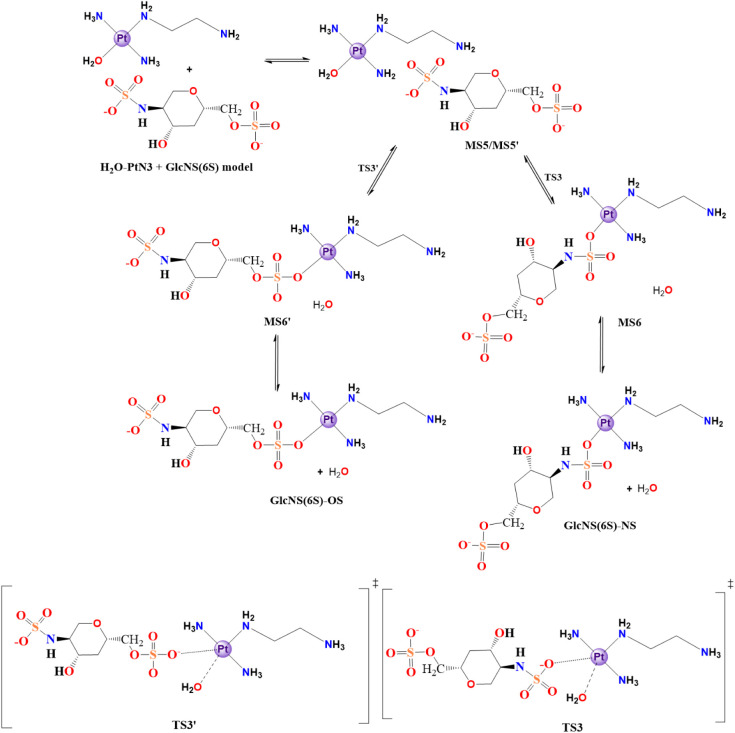
H_2_O/GlcNS­(6S)-Model Substitution in the Structure of the H_2_O-PtN3 Model[Fn sch3-fn3]

a MS5/MS5′ indicates the molecular complex between the species in their respective effective pose for each transition state. TS3 indicates the square-based pyramid active species comprising the NSO_3_^–^ group of sugar, whereas TS3′ refers to the same active species, but with the RSO_4_^–^ motif. MS6/MS6’ indicates the respective products as the molecular complex of GlcNS­(6S)-PtN3 and the molecule of water, and the last line demonstrates each product: sulfonate-coordinated (NS) or sulfate-coordinated (OS).

**Scheme 4:**
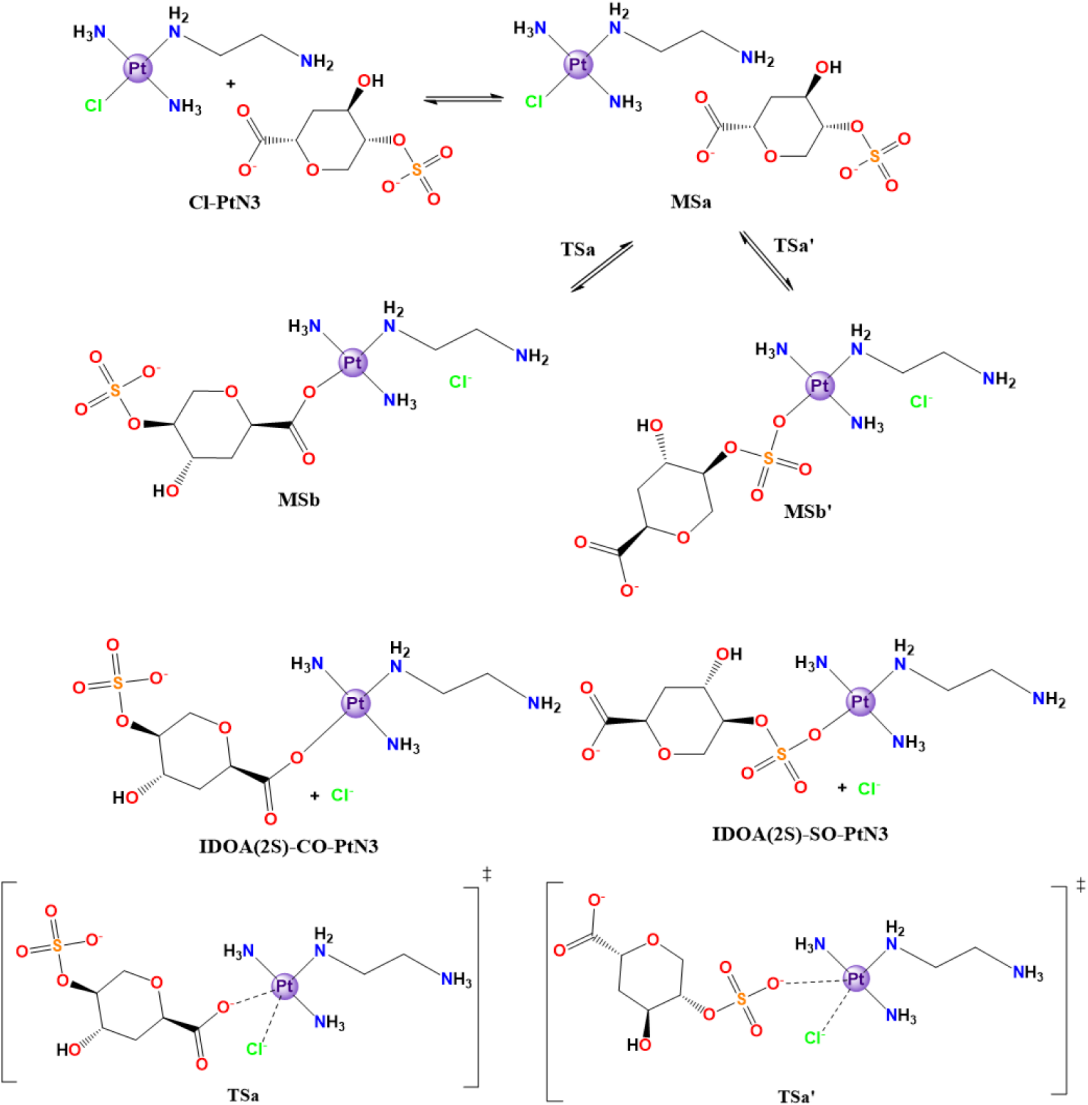
Cl^–^/IdoA­(2S)-Model Direct Substitution in the Structure of the Cl-PtN3 Model[Fn sch4-fn4]

a MSa/MSa’ indicates the molecular complex between the species in their respective effective pose for each transition state. TSa indicates the square-based pyramidal active species comprising the COO^–^ group of sugar, whereas TSa’ refers to the same active species, but with the RSO_4_^–^ motif. MSb/MSb’ indicates the respective products as the molecular complex of IdoA­(2S)-PtN3 and the molecule of chloride. The last line demonstrates each product: carboxylate-coordinated (CO) or sulfate-coordinated (SO).

**Scheme 5:**
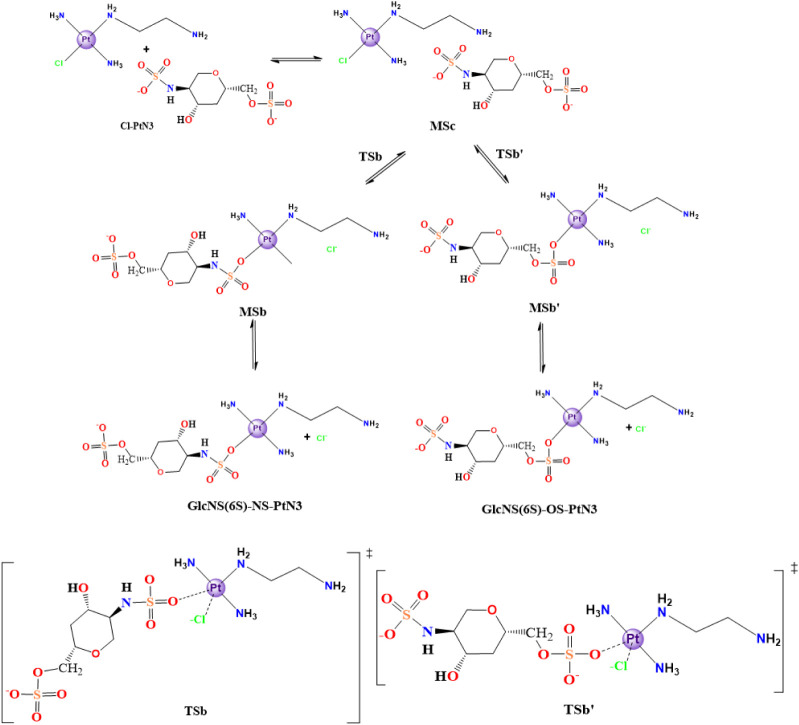
Cl^–^/GlcNS­(6S)-Model Direct Substitution in the Structure of the Cl-PtN3 Model[Fn sch5-fn5]

a MSc/MSc’ indicates the molecular complex between the species in their respective effective pose for each transition state. TSb indicates the square-based pyramidal active species comprising the NSO_3_^–^ group of sugar, whereas TSb’ refers to the same active species, but with the RSO_4_^–^ motif. MSd/MSd’ indicates the respective products as the molecular complex of GlcNS­(6S)-PtN3 and the molecule of chloride. The last line demonstrates each product: sulfonate-coordinated (NS) or sulfate-coordinated (OS).

#### 3.2.1 Substitution Reactions through an Aquation Step

First, the free energy profile of the chloride/water substitution was investigated, given that it is a common starting point for each sequential covalent adduct, as described in Scheme 1. The reaction starts with an entering water molecule, resulting in the solution free energy growth of about 4.5 kcal mol^–1^ and forming the molecular complex MS1. This step is followed by a transition state (TS1) with a barrier of 24.8 kcal mol^–1^ relative to the previous product and comprises a cisplatin-like square-based pyramidal transition structure, characterized by both the leaving chloride and the entering water molecule to the coordination sphere of platinum. This results in a Pt–Cl bond length (2.69 Å) longer than the Pt-aquo one (2.35 Å) in the transition species, forming an (Cl–Pt–OH_2_) angle of 68.7° (Tables S21 and S22). As a matter of comparison, the distance between Pt–NH_3_ is 2.04 Å, which is similar to that of Pt–NH_2_(CH_2_)_2_NH_2_. Upon overcoming the barrier, the product motif (MS2) is slightly higher in energy, with an overall ΔG of 11.2 kcal mol^–1^ with respect to the reactants. This Gibbs free energy variation grants the inverse reaction a barrier of 18.0 kcal mol^–1^, representing ΔΔ*G* ^‡^ = 6.8 kcal mol^–1^. Nevertheless, the separation of the chloride from the product complex, which would lead to the H_2_O–PtN3 structure, requires a concentration of 9.7 kcal mol^–1^. That final step forms a higher-energy complex (H_2_O–PtN3) of 20.9 kcal mol^–1^ compared with the starting material MS1.

More recently, some electronic analyses were applied to study metal-containing systems.^77–81^ An analysis of the atomic charge fluctuations along the aquation reaction coordinate reveals significant electronic redistributions (Figure 4). During the initial phase (−18<ζ<0), as the chloride ligand is displaced by the incoming water molecule, a progressive charge transfer occurs from both the platinum­(II) center (ΔqPt = 0.20 au) and the aqua oxygen (ΔqO23 = 0.11 au) toward the transammine nitrogen (ΔqN1 = −0.35 au), persisting until the critical distance is reached (ζ = 0). The chloride also removes electron density as it leaves the coordination site (ΔqCl22 = −0.26 au). The incoming water molecule displays an oxygen CHELPG charge (qO23) of −0.87 au, close to the qO23 = −0.90 au from isolated water. It forms a hydrogen bond to N4, which displays a slightly less negative charge than N6 (Δ*q* = 0.01 au). Around the critical distance, the metal center displays its highest charge of 0.42 au, before it starts decreasing as the water contribution becomes dominant.

**Figure 4:**
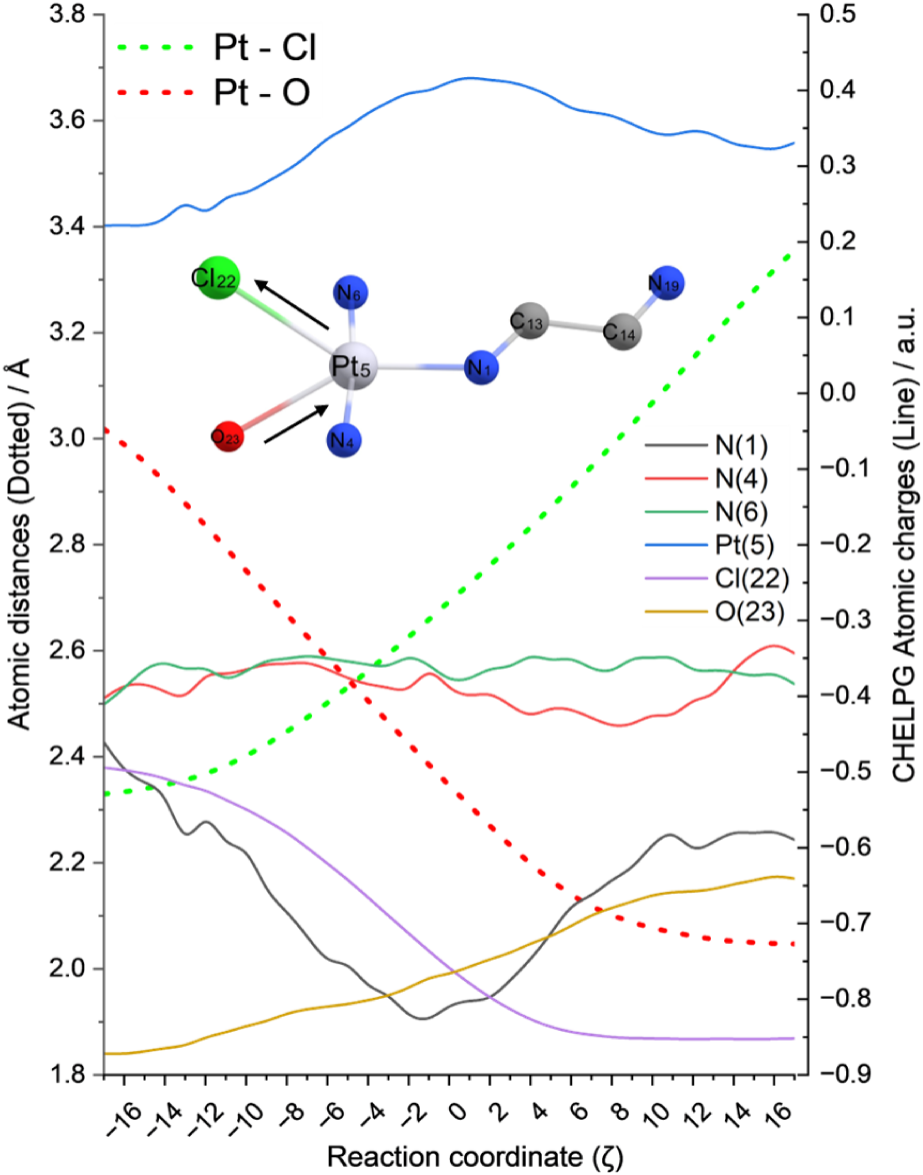
Bond distances and atomic CHELPG charges as a function of an arbitrary reaction coordinate in the aquation process. Calculations were performed at 17 arbitrary structures forward and backward from the transition state (ζ = 0).

Beyond the transition state (0 < ζ < 18), as the system descends the potential energy surface toward the product state, the oxygen atom exhibits a steady increase in charge density (ΔqO23 of 0.13 au since the TS, accounting for an overall ΔqO23 of 0.23 au). This occurs concurrently with the ammine charge reconstitution (qN1 = 0.59 au) and a slight electron density transfer surrounding the metal center, which drops to qPt = 0.33 au in the product. Although the electron density dynamics follow opposite tendencies along the aquation process, it does not express a symmetrical profile, as Pt­(II) is slightly more oxidized in the products than it is in the reagents (ΔqPt = 0.11 au), as well as the nitrogen from the diamine carries more atomic charge in the product (ΔqN1 = −0.13 au). Especially, this latter appears to have an important role in the substitution process, which is evidenced by a frontier orbital analysis shown in Figure 5.

**Figure 5:**
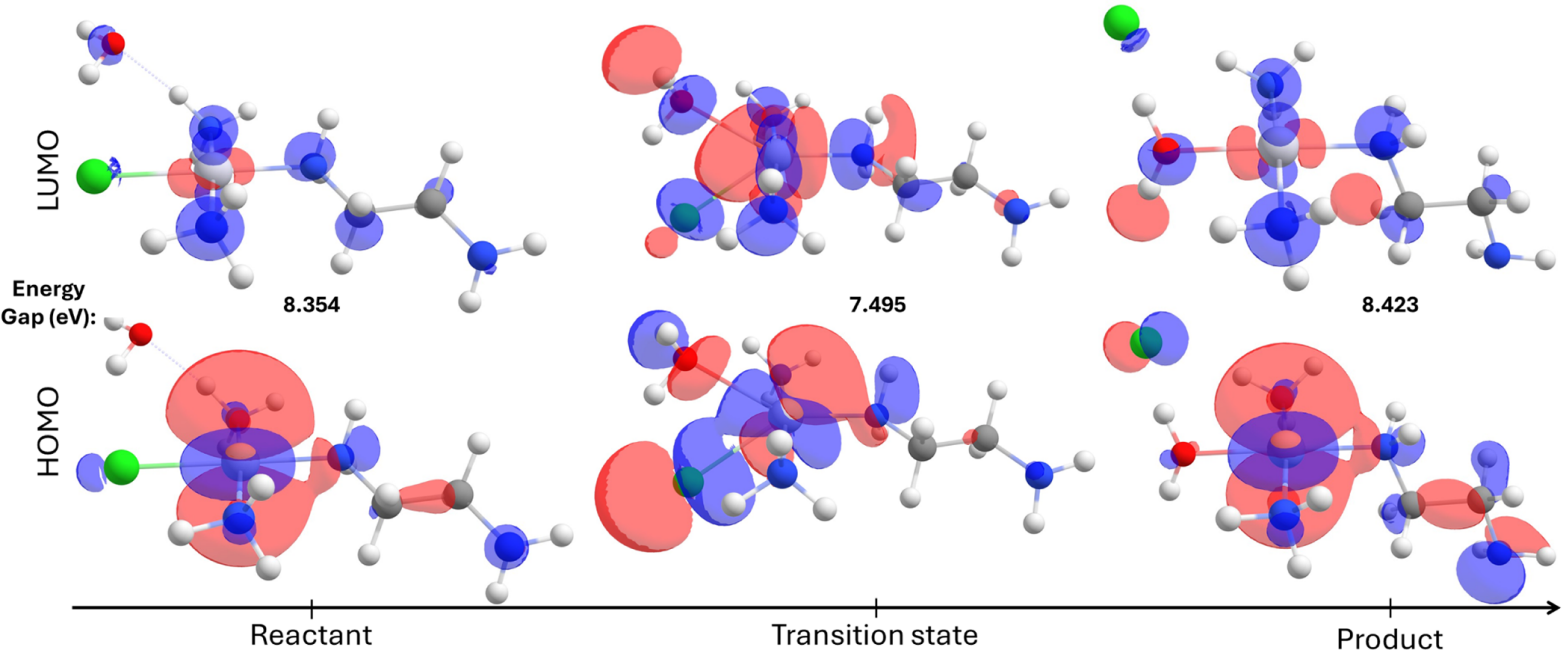
Molecular orbitals comprised of the species in the aquation process. Red and blue represent opposite phase isosurfaces (contour value = 0.025).

In general, the complexes demonstrate a very similar metal-centered HOMO orbital in both reactants and products, including a significant contribution from the diamine nitrogen atomic orbitals. This behavior is shared even with further substitutions using the carbohydrate models (see Figures S1–S8). Also, the variations in HOMO energies range from −8.2704 eV (reactants), passing through a slight increase in the TS (−7.6301 eV), before decreasing once again to −8.4081 eV (products). The stability of the complex (reactant or product) can be verified by LUMO energies. The reactant shows 0.0838 eV, while the product displays 0.0145 eV; the TS has a LUMO energy equal to −0.1348 eV, which agrees with a slight destabilization. HOMO–LUMO energies are displayed in Tables S9–S13.

An Atoms in Molecules (AIM) analysis was also carried out. In Tables S14–S16 , one can see the quantum-mechanical observable electron density (ρ) and the Laplacian of ρ, ∇^2^(ρ), which provide a measure of the local charge concentration or depletion. Such quantities reflect the behavior of the chemical bonds, recovering the shell structure of atoms. In all analyzed species—R, TS, and P—the negative ∇^2^(ρ) is located on the bonds that are intrinsically related to the oxygen of the water molecule. Also, the ∇^2^(ρ) values (in atomic units) for Pt–Cl at reactant (0.2596 au) and TS (0.1346 au) and Pt–O bonds at TS (0.2385 au) and product (0.5772 au) are quite representative for this ligand exchange, confirming the lability of the chloride atom.

##### 3.2.1.1 Reaction with the IdoA­(2S) Model

The first reaction path studied was the one involving the IdoA­(2S) model. This model comprises a carboxylate and sulfate group and, therefore, demonstrates two viable reaction pathways: the prior interaction with the COO^–^ (MS3) leading to the aquo ligand exchange through TS2 to a bond with one of the carboxylate oxygens (MS4), and the interaction with the RSO_3_^–^ group (MS3′), which passes through TS2’ to a covalent bond with the sulfate species (MS4’). Scheme 2 illustrates both of the processes.

In the first case, the minimum structure (MS3) for the interacting IdoA­(2S) model gave an overall solution free energy of 10.5 kcal mol^–1^. That minimum structure goes through a square-base pyramid transition state as the aquo ligand is substituted by one of the oxygens from the carboxylate group in the monosaccharide model, with a barrier of 17.9 kcal mol^–1^ in relation to MS3 (Δ*G* = 28.4 kcal mol^–1^ in relation to Cl-PtN3). This transition species consists of a leaving water molecule with a Pt–OH_2_ bond length of 2.42 Å and an incoming carboxylate oxygen with a Pt–OCO^–^ bond distance of 2.37 Å, forming a (H_2_O–Pt–OCO^–^) 62.6° angle, which is narrower than the angle formed in the aquation process (Tables S21 and S23). That transition step leads to the substituted product, which is 4.4 kcal mol^–1^ higher in energy compared to the starting material. After this step, the removal of the water molecule would decrease the free energy of the solution by 3.5 kcal mol^–1^ to the formation of IdoA­(2S)-CO-PtN3. The thermodynamic energy difference between the products of TS2 and the corresponding reagents creates a slightly higher reverse reaction barrier of 24.0 kcal mol^–1^ (ΔΔ*G* ^‡^ = 4.4 kcal mol^–1^).

On the other hand, the interaction mode MS3′, with the sulfate group near the aquo ligand of the platinum complex, resulted in a global solution free energy of 5.5 kcal mol^–1^, which is even more stable than MS3 (ΔΔ*G* = −5.0 kcal mol^–1^). Although the reported barrier for the sulfate-oxygen/H_2_O exchange is 21.0 kcal mol^–1^ in relation to MS3′, 3.1 kcal mol^–1^ higher than the analogous carboxylate substitution. For that, the reaction goes through the square-pyramid transition state TS2’. In this transition state, there is a slightly longer Pt–OH_2_ bond length (2.50 Å) compared to TS2, as well as the Pt–OSO_3_ distance (2.39 Å). The OH_2_–Pt–OSO_4_^3+^ angle is 67.3° (4.7° wider than TS2). After TS2’, it reaches the minimum structure MS4’ (Δ*G* = 12.1 kcal mol^–1^). Nonetheless, the differences in the minimum structures before and after TS2’ create significant substrate energy differences, with the backward reaction barrier of 14.4 kcal mol^–1^, which represents a ΔΔ*G* ^‡^ of −6.6 kcal mol^–1^ in relation to the forward barrier. Finally, different from the formation of IdoA­(2S)-CO-PtN3, the removal of the water molecule from MS4’, leading to IdoA­(2S)-SO-PtN3, stabilizes the product 6.0 kcal mol^–1^ more, with a global solution free energy of 6.1 kcal mol^–1^ (Figure 6).

**Figure 6:**
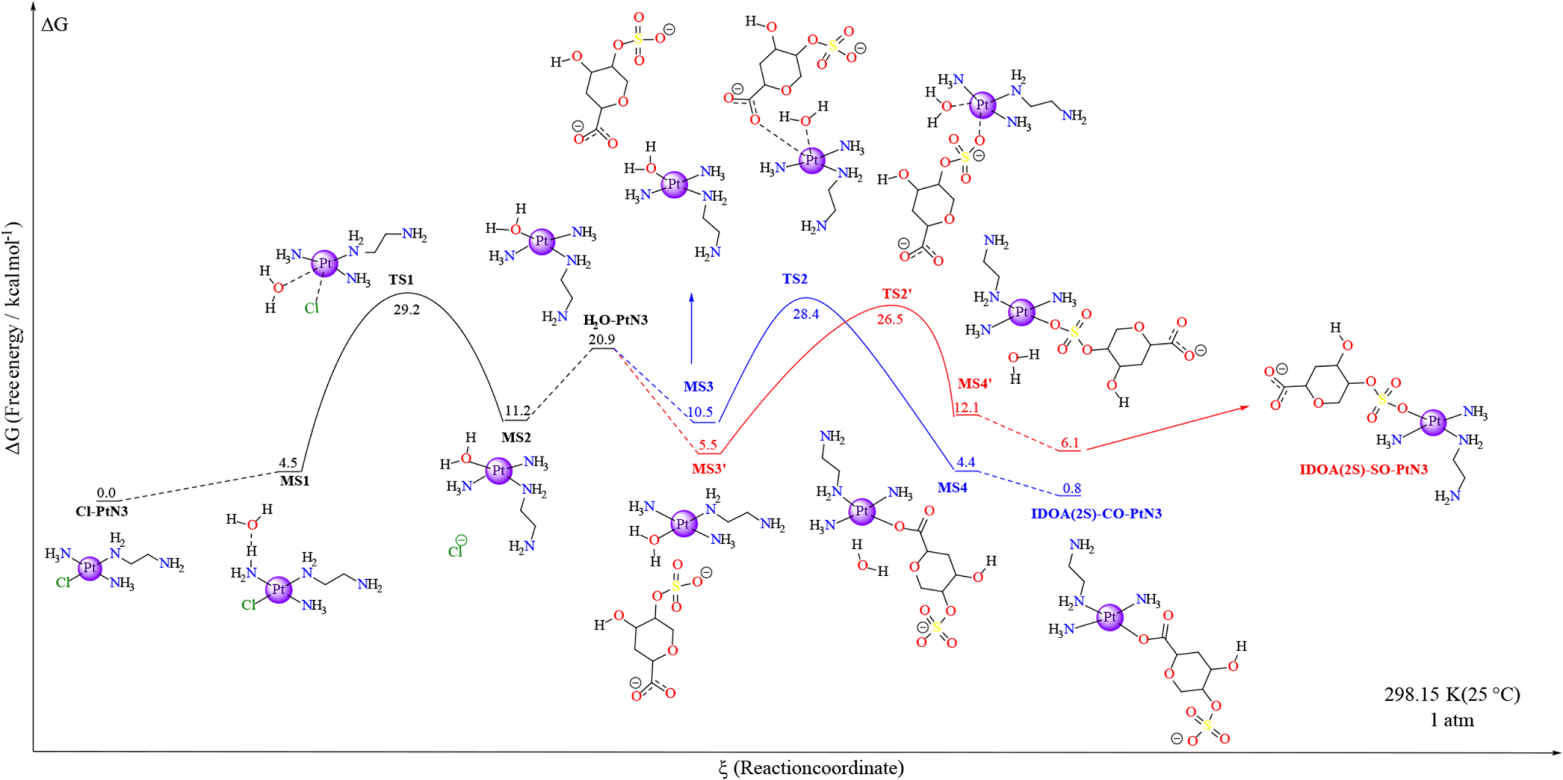
Free energy profile for the IdoA (2S) model substitution in the Cl-PtN3 through a previous aquation step, as shown in Scheme 1 and Scheme 2. The black line indicates the previous aquation step, and both blue and red illustrate the two possible paths for the reaction. Each marker is labeled with the respective nomenclature and in-solution Gibbs free energy in relation to the starting material (Cl-PtN3) in units of kcal mol^–1^. The platinum center’s 2+ charge and other reagents apart from the main ones have been omitted for clarity. The level of theory employed was B2PLYP/6-31+g­(d,p)//BHandH/6-31+G­(d,p), and Δ*G* was calculated according to eq 2 in the Methodology Section. All individual energetics are displayed in the SI under Table S1 and Table S3.

##### 3.2.1.2 Reaction with the GlcNS­(6S) Model

The other possible target for the substitution reaction is GlcNS­(6S), which comprises two sulfate groups (Scheme 3). The first way the substitution can occur is through an interaction mode with both the saccharide sulfate and the aquo ligand in the complex nearby (MS5). In that case, there is aquo substitution to the oxygen of the RSO_3_^–^ motif, which passes through the transition state TS2 in the direction of the product MS6 and then the GlcNS­(6S)-NS. Differently, it can undergo an aquo substitution to the NHSO_3_ group (TS2’) if the interaction between the species favors the proximity of the sulfonate group to the aquo ligand in the complex. This mechanism would result in the minimum structure MS6’, which results in the free GlcNS­(6S)-OS.

The first reaction route starts with the interaction of both monosaccharide and H_2_O-PtN3 (MS5), which displays a global aqueous free energy of ca. 0.0 kcal mol^–1^. From that point on the potential energy surface, the reaction must go through a barrier of 29.2 kcal mol^–1^ reaching the active species (TS3). The transition state is constituted of a square-base pyramid comprising the departure of the water (d­(Pt–OH_2_) = 2.51 Å) with the incorporation of the oxygen from the sulfonate group of the sugar model (d­(Pt-OSO2N) = 2.33 Å). The H_2_O–Pt–OSO2N angle is 67.0° (1.7° narrower than the previous TS in the aquation step [TS1] and 0.3° narrower than the analogous TS2’ in the IdoA­(2S)-model with respect to RSO_4_^–^, though it was reported to be 4.4° wider than the RCOO TS [TS2] in this latter monosaccharide model), whereas the ammine ligands in the coordination sphere of platinum form an angle of 179.0° (Tables S21 and S23). The decrease in energy from TS3 leads to the formation of MS6 (Δ*G* = 11.6 kcal mol^–1^), which converts to the product GlcNS­(6S)-NS-PtN3 with the removal of the interacting water, generating 5.0 kcal mol^–1^ of stabilization in the system (Δ*G* = 6.6 kcal mol^–1^). The differences in energy after and before TS3 produce a slender barrier of 17.6 kcal mol^–1^ in the backward reaction when compared to the direct route (ΔΔ*G* ^‡^ = −11.6 kcal mol^–1^).

The other possible course for the reaction to proceed is through an interaction that approximates the saccharide RSO_4_^–^ group and the aquo ligand in the complex coordination sphere (MS5′), with a global solution Gibbs free energy of 9.9 kcal mol^–1^ (Figure 7). The system must pass over an energy barrier of 20.2 kcal mol^–1^ to reach the transition state structure (TS3′, Δ*G* = 30.1 kcal mol^–1^) that will then relax to a minimum structure (MS6’) that contains the platinum complex interacting with a water molecule, with a solution Gibbs free energy of −12.1 kcal mol^–1^. The transition state TS3′ demonstrated a slightly narrower H_2_O–Pt–OSO_3_R angle in relation to TS3 (a difference of only 0.3°), whereas it showed one of the shortest Pt–H_2_O distance (2.43 Å) among the last four transition states discussed so far. Regarding the distance between the platinum and the ROSO_4_ group in the disaccharide model, the bond length in TS3′ is 2.37 Å, and the Pt–NH_3_ distances are the same as in the other models, 2.04 Å (Tables S21 and S23). The removal of the water molecule causes a slight difference in the free energy of the products, inducing an extra stabilization of 7.4 kcal mol^–1^. The final step leads to the separated product GlcNS­(6S)-OS-PtN3, which has a global Δ*G* of 4.8 kcal mol^–1^ in relation to the starting material (MS1). Again, the energy difference between the species before and after TS3′ creates a backward reaction barrier of 17.9 kcal mol^–1^, which is 2.3 kcal mol^–1^ lower than the one from the forward reaction.

**Figure 7:**
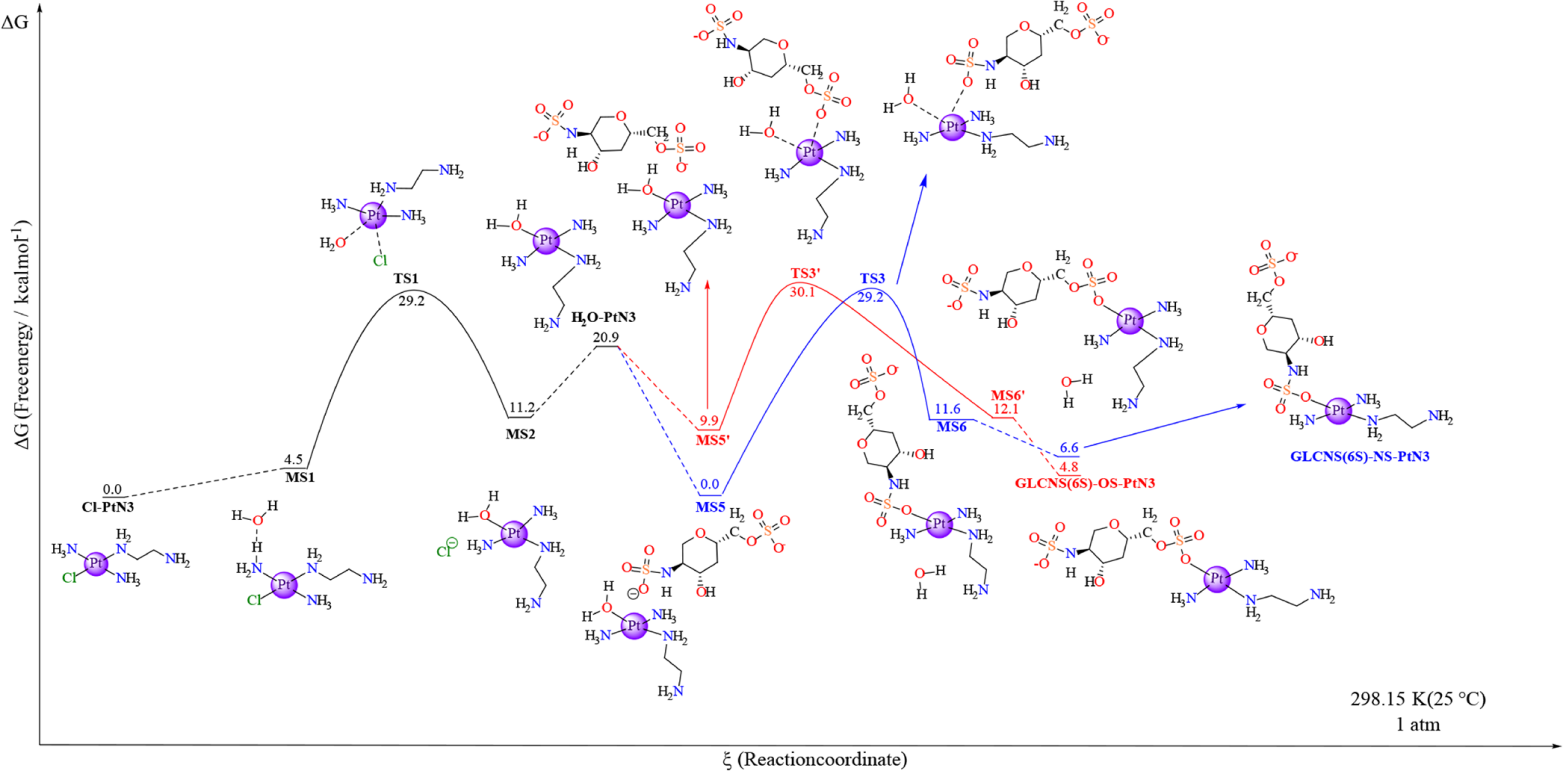
Free energy profile for the GlcNS (6S) model substitution in Cl-PtN3 through a previous aquation step, as shown in Scheme 1 and Scheme 3. The black line indicates the previous aquation step, and both blue and red illustrate the two possible paths for the reaction. Each marker is labeled with the respective nomenclature and in-solution Gibbs free energy in relation to the starting material (Cl-PtN3) in units of kcal mol^–1^. The platinum center’s 2+ charge and other reagents apart from the main ones have been omitted for clarity. The level of theory employed was B2PLYP/6-31+g­(d,p)//BHandH/6-31+G­(d,p), and ΔG was calculated according to eq 2 in the Methodology Section. All individual energetics are displayed in SI under Table S7 and Table S8.

#### 3.2.2 Direct Substitution Reactions

The salt concentration in the extracellular environment suggests that direct substitution of the chloride in Cl-PtN3 to the sugar sulfate oxygen species is a viable option for the reaction mechanism. In this section, we discuss both possible reaction routes for each monosaccharide model when undergoing a direct substitution reaction (2.2.1 and 2.2.2).

##### 3.2.2.1 Reaction with the IdoA­(2S) Model

The IdoA­(2S) model comprises both the carboxylate (COO^–^) and the sulfate groups (RSO_3_^–^) that can undergo a direct substitution with the Cl-PtN3 metallic complex. In comparison to the isolated Cl-PtN3 species, the interaction between the platinum complex and the monosaccharide model forms a molecular complex (MSa) that is −4.92 kcal mol^–1^ more stable than the starting material. This minimum species can undergo an energy barrier of 28.32 kcal mol^–1^ (23.40 kcal mol^–1^ relative to Cl-PtN3) through an active species (TSa) to reach MSb, which comprises IdoA­(2S)-CO-PtN3 interacting with a chloride anion, with an in-solution Gibbs free energy of 0.80 kcal mol^–1^ relative to the starting material. The final product, IdoA­(2S)-CO-PtN3, infinitely isolated from the Cl^–^ displays an in-solution Gibbs free energy of 0.85 kcal mol^–1^ relative to the starting material, which accounts for an energy increase of 0.05 kcal mol^–1^ considering the molecular complex Figure 8.

**Figure 8:**
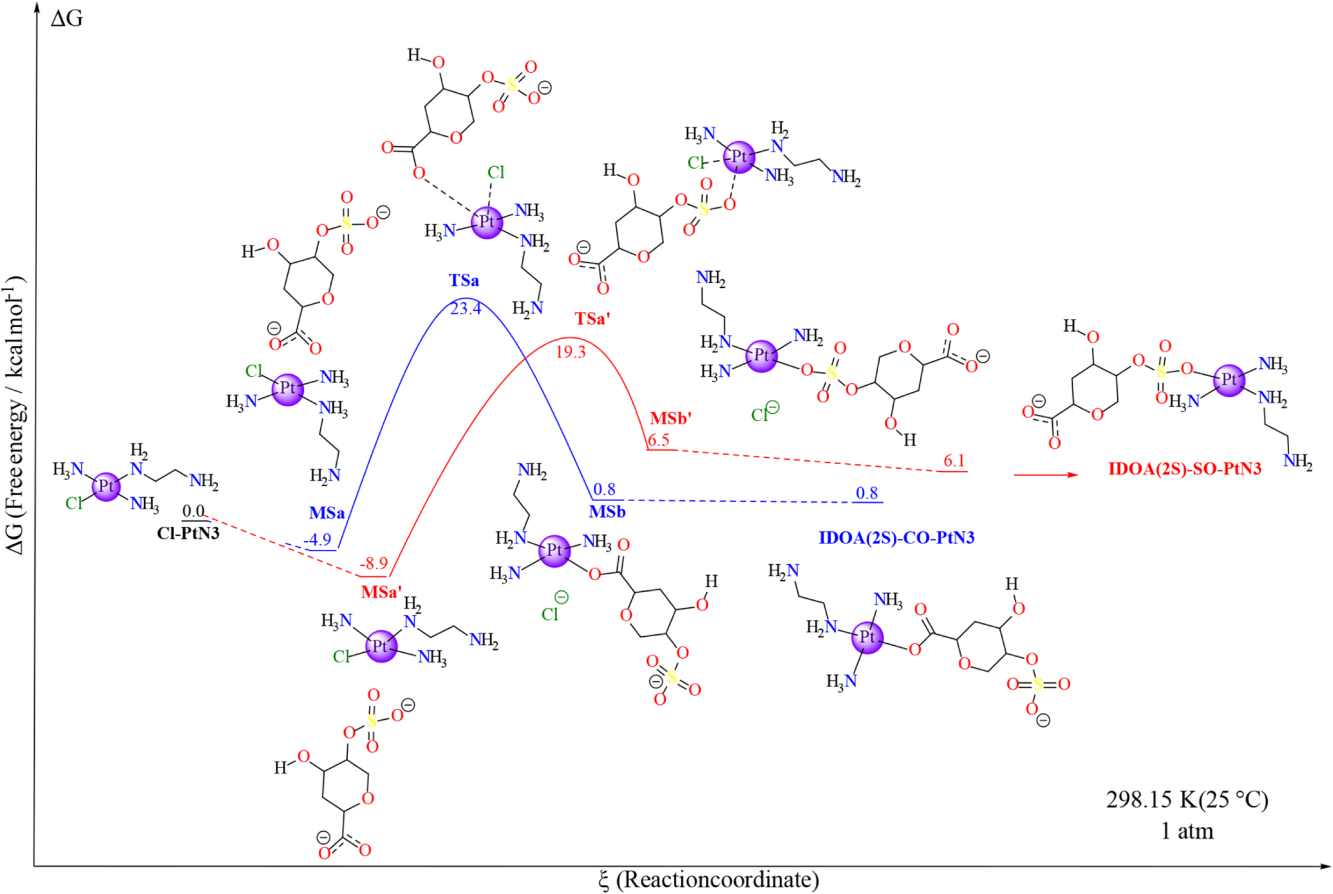
Free energy profile for the IdoA (2S) model direct substitution in the Cl-PtN3, as shown in Scheme 4. The black marker indicates the Cl-PtN3 metallic complex reagent, and both blue and red illustrate the two possible paths for the reaction. Each marker is labeled with the respective nomenclature and in-solution Gibbs free energy relative to the starting material (Cl-PtN3) in units of kcal mol^–1^. The platinum center’s 2+ charge and other reagents, apart from the main ones, have been omitted for clarity. The level of theory employed was B2PLYP/6-31+g­(d,p)//BHandH/6-31+G­(d,p), and Δ*G* was calculated according to eq 2 in the Methodology Section. All individual energetics are displayed in the SI under Table S17 and Table S18.

The energy difference between MSa, MSb, and the active species TSa infers for the inverse reaction barrier of 22.6 kcal mol^–1^, which is 5.7 kcal mol^–1^ lower than the direct reaction barrier in this step. The transition state TSa exhibits a “leaving-ligand” platinum distance, involving the chloride ion that is longer (2.63 Å) than the one with the water molecule in the water-driven process TS2 (2.42 Å) and in the same range as the one observed in the aquation process TS1 (Pt–Cl, 2.69 Å). It is also 0.32 Å longer than the same distance in the reactants (Cl-PtN3). Besides, the saccharide-model distance in the active species was marginally shorter compared to those in the previously studied process (∼0.6 Å shorter), and 0.28 Å longer than the one in the product IdoA­(2S)-CO-PtN3. Moreover, the (Cl–Pt–O–COR, 79.1°) angle between the labile ligands is wider in relation to the respective H_2_O–Pt–O–COR angle in the aquo-driven discussion (62.6°), and even regarding the angles present in the aquation step (Cl–Pt–H_2_O, 68.7°). All other distances, e.g., the ones between Pt and either NH_3_ or “dangling amine”, are maintained (Tables S21 and S24).

The other possibility is the sulfate oxygen substitution instead of the carboxylate oxygen in the platinum center, forming IdoA (2S)-SO-PtN3 (Figure 6). The interaction between the species that leads to a favorable RSO_3_^–^ interaction stabilizes the metallic complex at −8.9 kcal mol^–1^ (MSa’), in contrast to the analogous step for the previous substitution (MSa), which stabilizes at only −4.9 kcal mol^–1^ (ΔΔ*G* = 4.0 kcal mol^–1^). Then, the reaction is driven toward a transition state (TSa’) with an in-solution Gibbs free energy of 19.3 kcal mol^–1^, which represents an energy barrier of 28.2 kcal mol^–1^ in relation to the previous minimum structure (MSa’). Although lower than TSa (ΔΔ*G* ^‡^ = 4.1 kcal mol^–1^ in relation to the starting material, the barrier in relation to the previous minimum structure is quite similar, with a ΔΔ*G* ^‡^ of only 0.1 kcal mol^–1^. After going through the transition state, the reaction falls to a product minimum structure MSb’ that comprises the IdoA­(2S)-SO-PtN3 in complex with a chloride anion, displaying a free energy of 6.5 kcal mol^–1^ in relation to the starting material. The removal of the anion from the product results in an additional stabilization of 0.4 kcal mol^–1^, leaving the product IdoA­(2S)-SO-PtN3 with an overall in-solution Gibbs free energy of 6.1 kcal mol^–1^. The product is indeed 5.3 kcal mol^–1^ more unfavorable than IdoA­(2S)-CO-PtN3. The energy differences in the neighborhood of the transition state TSa’ demonstrate an inverse reaction activation energy of 12.8 kcal mol^–1^, which is lower than the direct reaction’s one (ΔΔ*G* ^‡^ = 15.4 kcal mol^–1^). The transition state TSa’ suffered similar structural modifications compared to TSa in relation to the analogous aquated path. A Pt–Cl distance of 2.72 Å is reported, which is longer than both Pt–Cl (TS1 aquation step, 2.69 Å) and Pt–OH_2_ (in the TS2’ structure, 2.50 Å). This also accounts for a 0.41 Å stretch in the Pt–Cl distance in the minimum reactant structure Cl-PtN3. In addition, Pt-OSO_4_R is 2.34 Å long, which is shorter than the analogous Pt-OSO_4_R (in TS2’, 2.39 Å) and 0.27 Å longer than the minimum product structure IdoA­(2S)-SO-PtN3. The angle between the leaving and coordinating ligands (Cl–Pt–OSO_4_R) is 75.6°, wider than both 67.3° in TS2’ (H_2_O–Pt–OSO_4_R) and 68.7° in TS1 (Cl–Pt–OH_2_). The other structural parameters farther from the ligand exchange triangle did not undergo significant changes (Tables S21 and S24).

##### 3.2.2.2 Reaction with the GlcNS­(6S) Model

Similar to the reactions with the previous monosaccharide model, GlcNS­(6S) can also undergo aquation reaction in two different groups: the sulfate (RSO_4_^–^) or the sulfonate (NSO_4_^–^). Considering the coordination via the sulfonate group first (NSO_3_^–^), there is in solution a Gibbs free energy stabilization of −2.6 kcal mol^–1^ in the formation of the molecular complex (MSc) (Figure 9). The reaction has an energy barrier of 26.2 kcal mol^–1^ (23.6 kcal mol^–1^ in relation to the starting material, Cl-PtN3), reaching a product molecular complex with an overall Gibbs free energy of 9.7 kcal mol^–1^ in solution. The final substitution product can be obtained with the removal of the chloride to an infinite distance, which results in a solution Gibbs free energy of 6.6 kcal mol^–1^ in relation to Cl-PtN3. The energy difference in the neighborhood of TSb provides the backward reaction with a barrier of 13.9 kcal mol^–1^ in relation to MSd (a ΔΔ*G* ^‡^ of 12.3 kcal mol^–1^ in relation to the direct reaction calculated from the previous minimum structure MSa). This reaction is thermodynamically unfavored due to the positive variation in the in-solution Gibbs free energy, even though it is a difference of 6.6 kcal mol^–1^; the species are expected to be in equilibrium once in solution. The transition state for this reaction, TSb, has a Pt–Cl bond length of 2.70 Å, which is longer than the corresponding leaving species in TS3 Pt–OH_2_ of 2.51 Å, and is comparable to the leaving chloride distance from platinum in TS1 (2.69 Å in the aquation-step TS). That bond distance corresponds to a stretch of 0.39 Å in comparison to the minimum metallic complex Cl-PtN3. Nonetheless, the angle Cl–Pt–NSO_3_^–^R is reported as 76.7°, wider than both the 67.0° in H_2_O–Pt–NSO_3_^–^R (TS3) and 68.7° in Cl–Pt–OH_2_ (TS1). Pt–NSO_3_^–^R is 2.33 Å, the same distance as that in TS3, which is 0.28 Å stretched from the minimum structure GlcNS­(6S)-NS-PtN3 (Tables S21 and S24).

**Figure 9:**
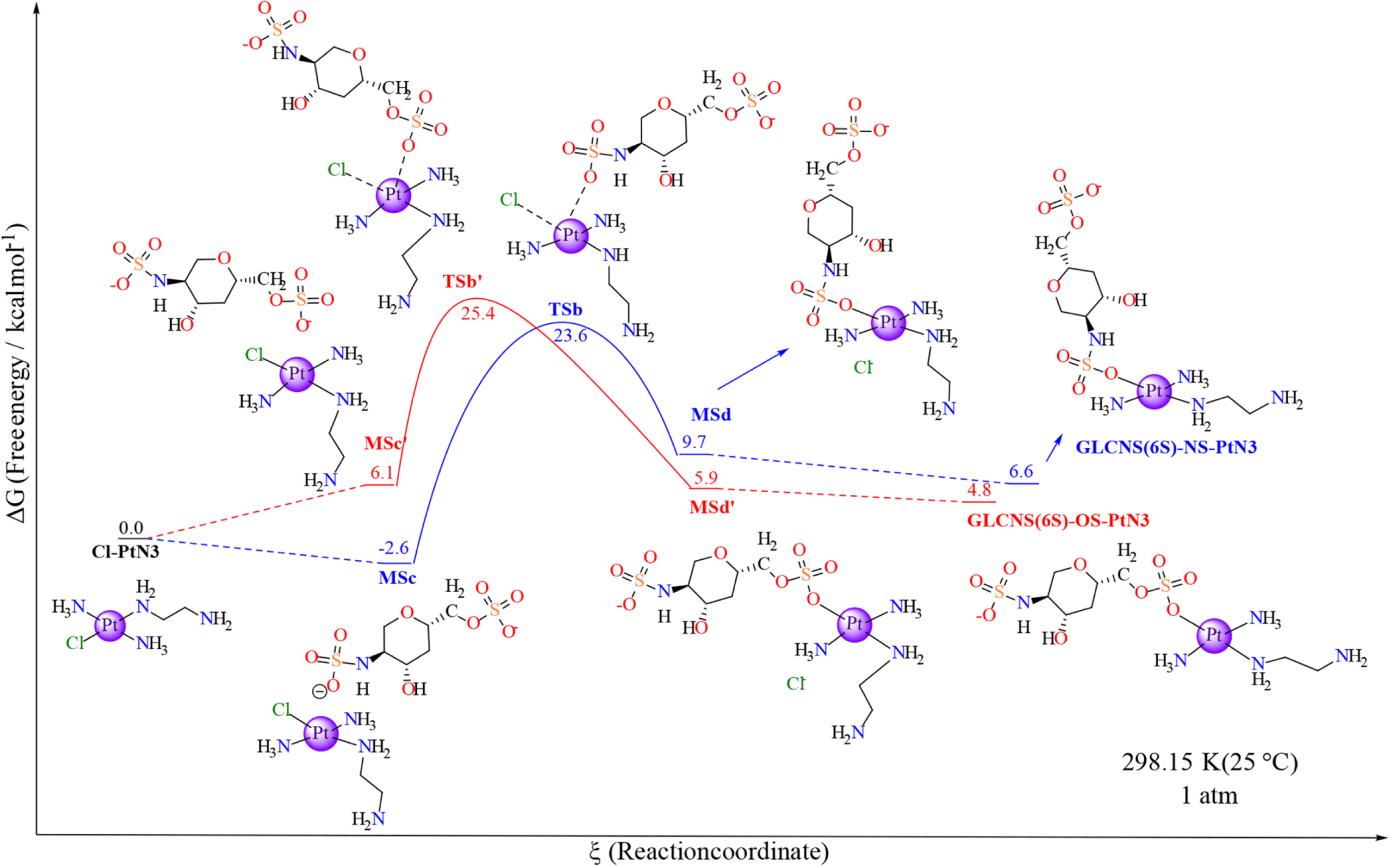
Free energy profile for the GlcNS­(6S) model direct substitution in Cl-PtN3, as shown in Scheme 5. The gray marker indicates the Cl-PtN3 metallic complex reagent, and both blue and orange illustrate the two possible paths for the reaction. Each marker is labeled with the respective nomenclature and in-solution Gibbs free energy relative to the starting material (Cl-PtN3), in units of kcal mol^–1^. Other reagents, apart from the main ones, are hidden for the sake of clarity. Supporting Information Tables S19 and S20 comprise the data shown here. The level of theory employed was B2PLYP/6-31+g­(d,p)//BHandH/6-31+G­(d,p) and Δ*G* was calculated according to eq 2 in the Methodology Section.

Differently, Cl-PtN3 can interact with an upcoming GlcNS­(6S) model molecule in an optimum geometry for undergoing a Cl^–^/RSO_4_^–^ substitution, forming a molecular complex (MSc’), with an energy increase of 6.1 kcal mol^–1^ (differing from the analog MSc, in which the molecular complexation with a molecule of water stabilized the system, demonstrating a ΔΔ*G* of 8.7 kcal mol^–1^ for this step). This reaction proceeds through TSb’ with an overall in-solution Gibbs free energy of 25.4 kcal mol^–1^, representing a barrier of 19.3 kcal mol^–1^ relative to MSc’ and reaches the product molecular complex (MSd’) with an in-solution Gibbs free energy of 5.9 kcal mol^–1^. The energy level of MSd’ provides the inverse reaction with a barrier of 19.5 kcal mol^–1^, which is 0.2 kcal mol^–1^ higher than that of the direct pathway. Again, analogous to the substitution with NSO_3_, the reaction is thermodynamically unfavorable due to the positive variation in the in-solution Gibbs free energy, although it is present in chemical equilibrium. The transition state reports a Pt–Cl distance of 2.70 Å, identical to the RNSO_3_^–^ substitution alternative (which corresponds to a difference of 0.27 Å relative to the minimum product GlcNS­(6S)-OS-PtN3) and a Pt-NSO_3_^–^ distance of 2.32 Å (Δ = 0.39 Å relative to Cl-PtN3, minimum structure of the reactant). The angle between Cl–Pt–NSO_3_^–^ is 78.1°, and no other structural parameter showed significant changes (Tables S21 and S24).

### 3.3 Substitution Reactions through an Aquation Step: Kinetic Analysis (PCM)

A simple kinetic analysis comprising the aquation step employing the PCM-only solvation method indicated a forward-reaction velocity constant (*k*_1_) of 2.44 × 10^–9^ s^–1^ and a backward velocity constant (*k*_–1_) of 3.96 × 10^–1^ s^–1^. The species involved in this transition state, considering the direct reaction, allow us to determine the following rate law:

6 $$R=k_{\mathrm{obs}}\left[Cl-PtN_{3}\right]$$

where

7 $$k_{\mathrm{obs}}=k_{1}\left[H_{2}O\right]$$

Because the utilized solvent is water, its concentration is constant, with a value of 55.5 mol L^–1^; thus we can calculate a value of ca. 1.35 × 10^–7^ L mol^–1^ s^–1^ for *k*_obs_. This is a pseudo-first order rate constant, which corresponds to an effective $\Delta G_{\mathrm{obs}}^{‡}$ of 26.8 kcal mol^–1^; thus at room temperature, it is possible for the reaction to occur; nonetheless, at a very slow rate.

Experimentally, Gorle et al. and Ruhayel et al. determined the rate constants for both monosaccharide and disaccharide species.^28–30^ Comparing the obtained rate constant for the aquation process (*k*_1_ = 2.44 × 10^–9^ s^–1^), it differs from the experimental ones (1.56–3.40) ×10^–5^ s^–1^ in 4 orders of magnitude. A better comparison can be done with the calculated barriers for this process: employing eq 4 in the Methodology Section the experimental aquation barrier of Δ*G* ^‡^ = ca. 23.6 kcal mol^–1^ is obtained, which is 5.65 kcal mol^–1^ lower than the calculated one (29.25 kcal mol^–1^/B2PLYP). These results indicate that solvation waters may play an important role in stabilizing the transition state and are, therefore, important for the correct description of the reaction.

### 3.4 The Effect of a Hybrid Solvation Model

To further improve the description of the chemical process, an explicit solvent layer was considered. The number of water molecules to be included in the solvation sphere was evaluated based on a radial distribution function (RDF) of molecular dynamics simulation regarding the aquation process and inferred to the other systems of interest. The PCM continuum model was retained, representing the bulk solvent effect. Based on the MD, the number of water molecules present in the solvation layer is 6, apart from the incoming water that undergoes the substitution reaction. We evaluated two hybrid solvation layer approaches comprising both 3 (1/2 of the solvent molecules evaluated) and 6 explicit solvent entities. Information regarding the molecular dynamic’s simulation is available in Figures S9–S16. Nonetheless, in order to include the thermodynamic effects of explicit water molecules in the calculated free energies, we employed eq S17, which was derived in the SI (eqs S6 to S17).

In general, the relative free energy of minima states was underestimated, and transition states were overestimated by the lack of an explicit solvation layer. Along the aquation process, the inclusion of 3 water molecules slightly reduces the described free energy of MS1, which is restored to a higher stage with the inclusion of 3 more entities. The free energy of TS1 drops by 4.09 kcal mol^–1^ in the presence of 3 WAT and decreases further to 23.71 kcal mol^–1^ (a reduction of an additional 1.45 kcal mol^–1^) when considering 6 solvating waters. This comprises a consistent decrease in the direct reaction barrier and an increase in the inverse reaction barrier, attributing a continuous favorability to the products of the reaction (MS1→MS2, WAT[0] = 24.79 kcal mol^–1^, WAT[3] = 22.57 kcal mol^–1^ and WAT[6] = 18.98 kcal mol^–1^; MS2→MS1, WAT[0] = 18.03 kcal mol^–1^, WAT[3] = 19.08 kcal mol^–1^ and WAT[6] = 21.56 kcal mol^–1^). These data agree with the necessity of including explicit water molecules to properly describe the free energy potential of the platinum reaction, especially those comprising polar solvents with hydrogen bond capabilities. All processes of the reactions of interest are displayed in Tables S25–S29 as a function of explicit water molecules in the solvent layer.

This effect can be associated with the solvent anisotropic solvation and the hydrogen bond framework that is formed and stabilizes the transition state. The starting material demonstrates ca. 5 hydrogen bonds among Cl-PtN3 and water molecules, whereas when undergoing the active species barrier, this number increases to ca. 8, indicating the importance of considering the first solvation layer when treating this reaction. Supporting Information Figures S7 and S8 summarize the hydrogen bond framework formed along the aquation procedure.

### 3.5 Kinetic Analyses (Hybrid Solvation Model)

#### 3.5.1 Aquation Step and Reaction with the IdoA­(2S) Model

The newly calculated activation energies for the aquation process, Δ*G* ^‡^ = 23.71 kcal mol^–1^, represents an error of only 0.1 kcal mol^–1^ in relation to the experimental one, which corresponds to a rate constant of 2.631 × 10^–5^ s^–1^. This new estimated rate constant is in the experimental range and describes the aquation step. According to eq 7, the new effective barrier $\left(\Delta G_{\mathrm{obs}}^{‡}\right)$ drops to 21.3 kcal mol^–1^ and indicates the possibility of the reaction occurring at room temperature at reasonable rates. Herein, for the aquation-driven reaction of Cl-PtN3 and IdoA­(2S), the chloride/water exchange is the rate-determining step, and the kinetic law follows the one deduced previously.

#### 3.5.2 Reaction with the GlcNS­(6S) Model

Similarly, regarding the aquation-driven reaction with GlcNS­(6S) (eqs S34 and S35), we have one *k*_obs_ for each product, which, considering a water concentration of 55.5 mol L^–1^ results in *k*_obsMS6_ of 8.35 × 10^–11^ and *k*_obsMS6*′*_ of 1.70 × 10^–10^. Employing eq 4, we obtain an effective barrier heights of 31.2 kcal mol^–1^ and 30.8 kcal mol^–1^, respectively.

#### 3.5.3 Direct Substitution Reactions

For direct reaction substitutions, there are two pathways that may impact the reaction. Because the first step involves a fast equilibrium for the formation of a preassociation complex between disaccharide and Cl-PtN3, and is followed by the slow step, which leads to the formation of the final product, we used the quasi-equilibrium approximation for determination of the kinetic law. Regarding model IdoA­(2S), the reaction corresponds to a second-order *k*_*obs*_ of −9.33 s^–1^, the effective barrier is calculated as 16.1 kcal mol^–1^, as derived in eq S39. In contrast, model GlcNS­(2S), considering eq S43, comprises an observable rate constant (*k*_obs_) of −1.63 × 10^–5^ s^–1^, which corresponds to an effective reaction barrier of 24.0 kcal mol^–1^.

### 3.6 Microkinetic Analysis

The hybrid solvation models were evaluated microkinetically. All steps are considered in equilibrium, and initial concentrations of the monosaccharide and platinum complex models are defined as 0.005 M and 0.001 M, respectively. The ionic force of the environment was also acknowledged by an initial 0.001 M concentration of chloride. Figure 10 depicts the microkinetic simulations for the aquation procedures. In respect to the IdoA­(2S) model, there is a fast conversion of the starting material into the aqua-compound complex MS2 and H_2_O-PtN3, which is majorly converted into MS3. The [Cl–PtN3] = [MS3] = ∼0.39 mmolL^–1^ at 798 s, along with hardly ∼0.067 mmolL^–1^ of MS3′. This is followed by the formation of MS4 and IdoA­(2S)-CO-PtN3 with equilibrium concentrations of 1.39 and 0.24 μmolL^–1^, respectively, at 3997 s. The formation of the sulfate-complexed IdoA­(2S)-SO-PtN3 is only observable at the picomolar scale in the microkinetic simulations. Nonetheless, it presents an initial rapid production with a maximum at ∼585 s due to kinetic favorability; however, it rapidly decays in concentration due to the formation of the previous thermodynamic product.

**Figure 10:**
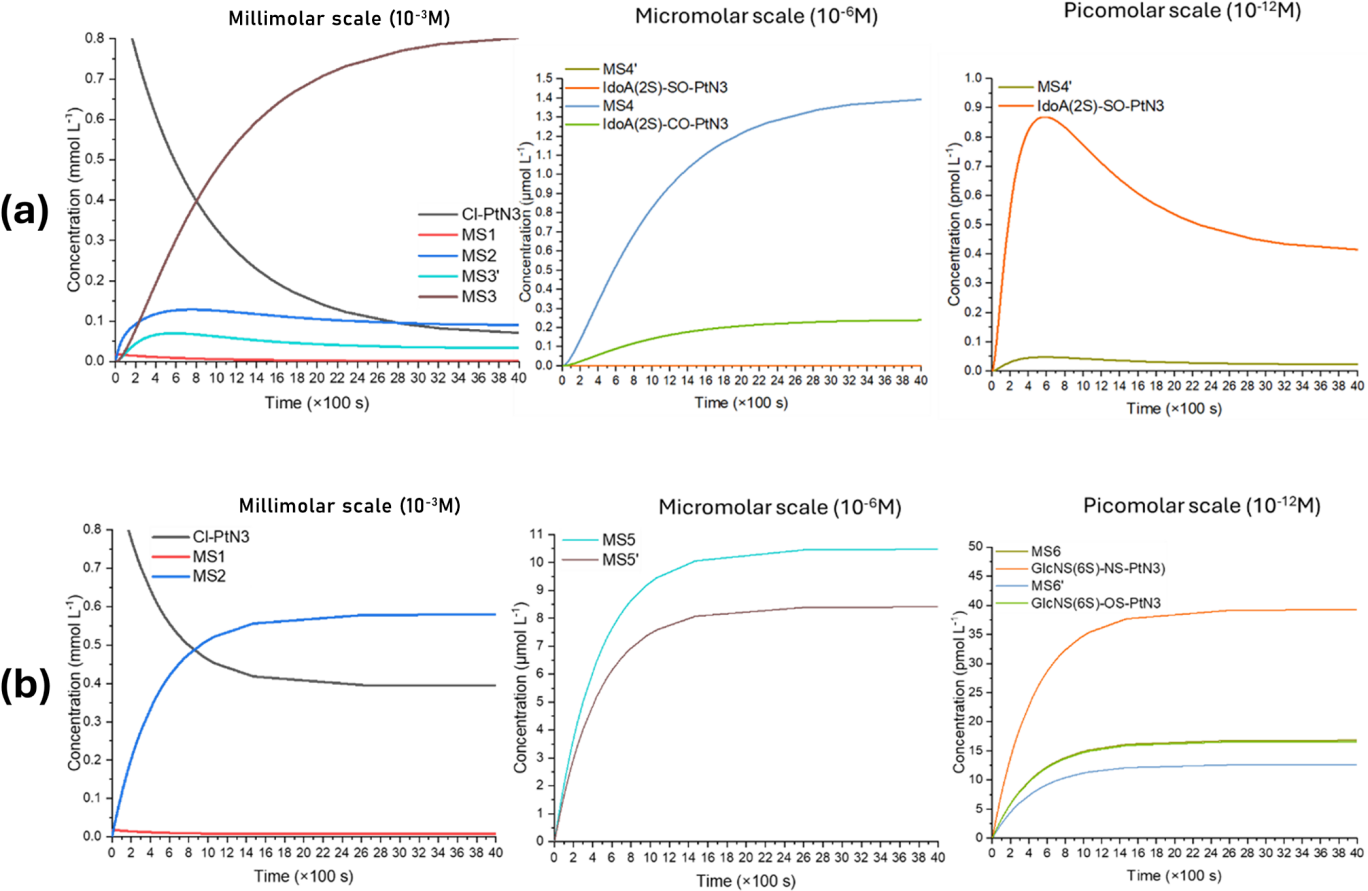
Microkinetic modeling of the reaction involving both (a) IdoA2S and (b) GlcNS­(6S) monosaccharide models, along with Cl-PtN3, via a previous aquation step.

Regarding GlcNS­(6S), starting at the millimolar scale, the formation of MS2 is observed predominantly and reaches the same [Cl-PtN3] concentration of 4.9 mmol L^–1^ at ∼860s. Final equilibrium is achieved at a concentration of 5.8 mmol L^–1^ after about 2577 s. This advancement in reaction is followed by the formation, in the micromolar scale, of the aquation product in both interaction sites of GlcNS­(6S) (MS5 and MS5′), which leads to the active species TS3 and TS3′. Finally, when examined on the picomolar scale, the predominant formation of GlcNS­(6S)-NS-PtN3 is observed over GlcNS­(6S)-NS-PtN3, along with their water-complex products MS6 and MS6’, where chemical equilibrium is achieved after 2591 s.

Both direct reaction microkinetic simulations show the formation of the product complexes and isolated substituted platinum species on the micromolar scale. The IdoA­(2S) model also demonstrates a majority formation of the carboxylate-coordinated metal complex IdoA­(2S)-CO-PtN3, with an equilibrium concentration of MSb of ∼36.10 μmolL^–1^. Regarding the GlcNS­(6S) model, both products were formed at an equilibrium ratio of approximately 1:8 (OS:NS). Figure 11 depicts the direct substitution microkinetic modeling.

**Figure 11:**
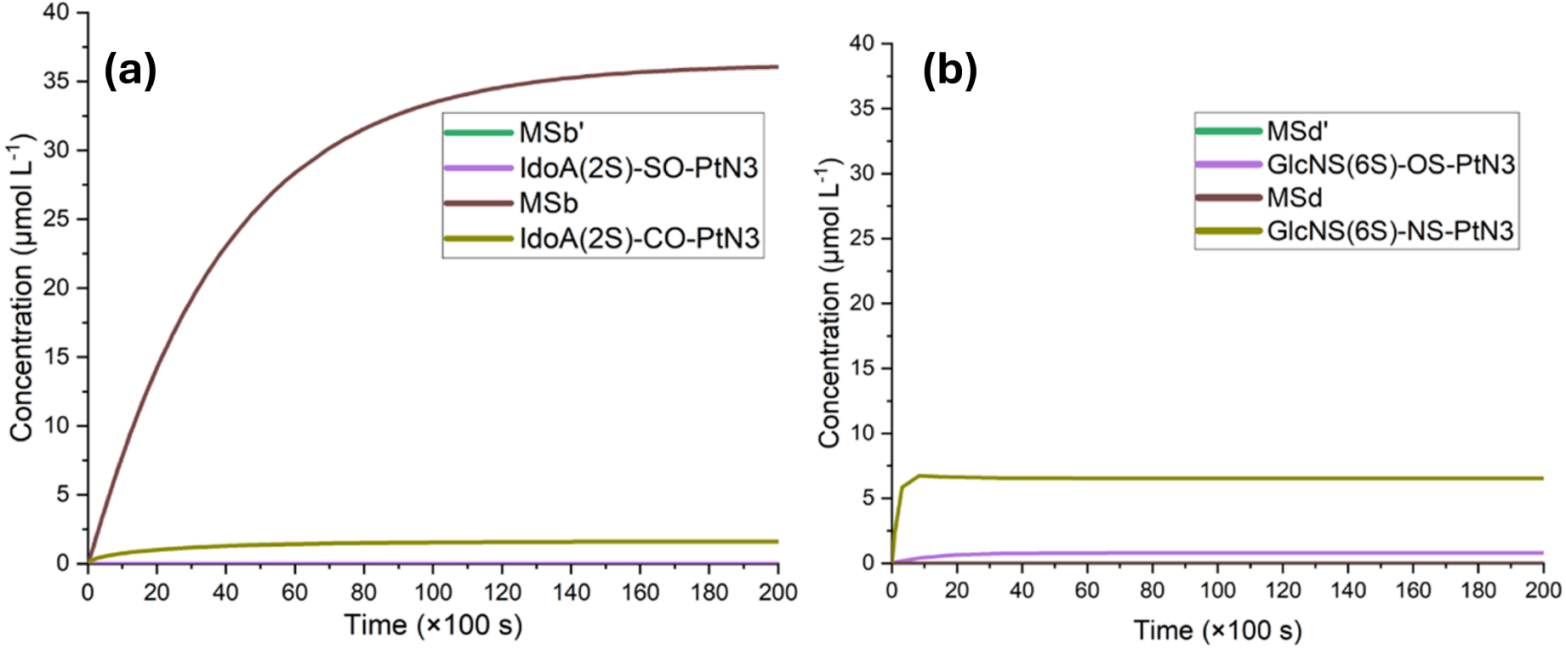
Microkinetic modeling of the reaction involving both (a) IdoA2S and (b) GlcNS­(6S) monosaccharide models, along with Cl-PtN3, via a direct substitution reaction.

## 4 Conclusion

The performed benchmarking demonstrated B2PLYP as the preferred DFT functional to describe the mechanism of the substitution reactions. This method performed well in contrast to the reference (DLPNO–CCSD­(T), with an absolute deviation of only 1.05 kcal mol^–1^) and was employed to generate the in-solution Gibbs free energy for all reaction procedures. The free energy profile for each reaction studied was evaluated and compared to the available data in the literature. The substitution with the IdoA­(2S)-model presented a carboxylate substitution barrier of ca. 21.5 kcal mol^–1^ and a higher sulfate substitution barrier of ca. 27.5 kcal mol^–1^, both in relation to the starting material. On the other hand, a GlcNS­(6S)-model substitution displayed a sulfate substitution barrier of ca. 26.7 kcal mol^–1^ in comparison to the sulfonate barrier of ca. 27.0 kcal mol^–1^ of the sulfonate one. Moreover, the direct substitution reactions demonstrated similar values of reaction barrier in the rate-determining reaction step for GlcNS­(6S)-model, whereas barrier heights for direct substitution regarding IdoA­(2S)-model were considerably lower. The Cl^–^/IdoA­(2S)-model displayed a carboxylate substitution barrier of ca. 16.3 kcal mol^–1^, whereas the sulfate barrier is ca. 17.0 kcal mol^–1^. In addition, the microkinetic analysis displayed the formation of products regarding the IdoA­(2S) model on the micromolar scale.

Extrapolated to a biological context, the analysis confirms that the carboxylate and sulfate exocyclic oxygens of polysaccharides are valid ligands for substitution reactions of platinum antitumor agents. The situation is somewhat analogous to that of carbonate, where the intracellular presence of cisplatin–carbonato complexes has been demonstrated.^11,82^ In the present case, while formally weak donor ligands, their location in the extracellular matrix and stroma of tumor cells suggests that, in an environment of localized high concentration, these ligand exchange reactions, even of a transient nature, may be considered pertinent to the observed tumor accumulation of polynuclear platinums in tumors with high GAG concentrations.^23^

## Supplementary Material



## Data Availability

The optimized geometry structures presented here are freely and publicly available at the Supporting Information file and as.xyz files at https://github.com/fred4h/A-kinetic-overview-of-Polynuclear-PlatinumII-Complexes-and-heparan-sulfate-substitution-reaction

## Notes

SI includes: benchmarking study details, including equations and specific references; additional figures and tables with complementary data; and all optimized structures in the format of xyz coordinates. All Supporting Information is available as a separate file accompanying this manuscript (PDF)
