# Supplementary material for: Kinetic Overview of Polynuclear Platinum(II) Complexes in Heparan Sulfate Substitution: Mimetic Model Analysis and Mechanistic Perspectives
Source: ACS Omega. 2026 Apr 28;11(18):26779–96. doi: 10.1021/acsomega.5c13472 (PMC13176995; doi:10.1021/acsomega.5c13472)
Supplement: Supplementary file 1 [file ao5c13472_si_001.pdf]

Supplementary Information (SI)

Kinetic Overview of Polynuclear Platinum(II) Complexes in Heparan Sulphate Substitution: Mimetic Model Analysis and Mechanistic Perspectives

Frederico Henrique do Carmo Rufino Ferreira[1], Nicholas Patrick Farrell[2], Luiz Antônio Sodré Costa[1]*

1. NEQC – Departamento de Química – Universidade Federal de Juiz de Fora, Juiz de Fora, MG - Brasil.

2. Department of Chemistry, Virginia Commonwealth University, Richmond, VA 23284 - 2006, USA.

Supplementary Methodology

Benchmarking Study

All structures were obtained employing optimization calculations in the DFT [1,2] functional BHandH [3] along with 6-31+G(d,p) Pople's basis set [4] for light atoms and SDD [5] for platinum. The optimized geometries were verified as true minima on the potential energy surface by performing harmonic frequency calculations at the same level of theory. The transition states were verified by the presence of a single imaginary vibrational frequency in the harmonic frequency calculations, indicating that they are first-order saddle points on the potential energy surface. All calculations were performed including the polarized continuum model IEF-PCM (water) [6] and were carried out in Gaussian 09 D1 software package (G09) [7] unless indicated otherwise in the manuscript. In order to test the influence of some modifications in the computational protocol during the calculations of the smallest mimetic system some alternative methodologies, which were not implemented in G09, were appraised such as novel DFT functionals and wavefunction-based methods; for those the software package ORCA 5.0.4 [8–10] was adopted. All ORCA methodologies and results are indicated with a respective subscript and implemented CPCM solvation model [11,12]. Some comparisons between G09 and ORCA calculations were performed assuring the equivalence of the results in the same computational protocol and are available in the supplementary material.

Single point energy calculations were performed in different levels of theory in order to evaluate the influence of the DFT functional in the Born-Oppenheimer energy (E_{BO}) of the chemical process of interest, here defined as the sum of electronic energy (E_{el}) and nuclear repulsion (V_{NN}) terms as shown in equation S1:

$$E_{BO_g} = E_{el_g} + V_{NN_g} \qquad \text{(Eq. S1)}$$

Among the computational methods evaluated in the smallest system there are DFT functionals such as $BHandH_{G09}$, $wB97XD_{G09}$ [13], $M06_{G09}$ [14], $PBE0_{G09}$ [15,16], $B3LYP_{G09}$ [17], $CAM\text{-}B3LYP_{G09}$ [18], $B2PLYP_{G09}$ [19], $SOGGA11X_{G09}$ [20], $r2SCAN_{ORCA}$ [21], $wB97M\text{-}V_{ORCA}$[22] and the wavefunction-based method employed as reference $DLPNO\text{-}CCSD(T)_{ORCA}$ [23,24] which demonstrated an energy deviation of only 0.5 kcal mol^{-1} in relation to CCSD(T) [25]. This last with the basis set def2-TZVPP [26] and auxiliary basis set def2-TZVPP/C [27] which speeds up the calculations at a cost of a small error. Those methodologies selected for single point calculations were among the best performing DFT functionals for reaction barriers, based on a work conducted by Mardirossian and collaborators [28].

In order to properly represent the species in solution, the thermodynamic standard state was changed from 1 atm to 1 mol L^{-1} and the final free energy in solution phase was calculated as a sum of the Born-Oppenheimer energy (E_{BO}), the solvation contribution to the free energy, the thermal correction to the energy (G_n) which refers to nuclear contributions (translational, vibrational and rotational motions) and the energy variation, which describes the standard state change (1.89 kcal mol^{-1}). The terms are displayed according to the equation S2 below.

$$G_{sol} = E_{BO_g} + \Delta G_{solv} + G_n + 1.89\ kcal\ mol^{-1} \qquad \text{(Eq. S2)}$$

Also, enthalpies for the reactions were calculated as a way to evaluate the entropy contribution for the reaction steps of interest when contrasting the energies with their respective free energies. For this, equation S3 demonstrates the enthalpy in solution (H_{sol}) calculation as a sum of the Born-Oppenheimer energy in gas (E_{BO_g}) with the solvation energy (ΔH_{solv}) and the thermal correction to the enthalpy (H_n).

$$H_{sol} = E_{BO_g} + \Delta H_{solv} + H_n \qquad \text{(Eq. S3)}$$

In the thermodynamic properties (G_{sol} and H_{sol}) both thermal corrections (G_n and H_n) were calculated in the same level as the optimization procedure BHandH/6-31+G(d,p)-SDD(Pt) by harmonic frequency calculations.

All steps of the reactions were assigned with its respective rate constant, k (Eq. S4) and equilibrium, K_{eq} (Eq. S5) constants according to the Gibbs free energy of the step. In Eqs S4 and S5 k_b is the Boltzmann constant, T is the temperature considered as 25 °C unless said otherwise in the manuscript, h is the Planck constant, and R is the universal gas constant. For the equilibrium constant ΔG indicates the solution free energy for the reaction step and in the rate constant equation $\Delta G^{\ddagger}$ indicates the solution activation energy for the reaction to proceed.

$$k(t) = \frac{k_b T}{h} e^{\frac{-\Delta G^{\ddagger}}{RT}} \quad \text{(Eq. S4 – Eyring's equation)} \qquad K_{eq} = e^{-\frac{\Delta G}{RT}} \quad \text{(Eq. S5)}$$

Microkinetic modelling was performed with KINTECUS software package [29], which integrates all kinetic laws and simulate species concentration along the reaction time.

Hybrid Solvation

For the hybrid models, regarding the aquation step,

$$Cl - PtN3^{+} + H_2O \rightarrow H_2O - PtN3^{2+} + Cl^{-} \qquad \Delta G^{*}_{sol} \qquad \text{(Eq. S6)}$$

It was considered both 3 and 6 explicit water molecules. For n molecules of water, the solvation of the starting material can be written as

$$Cl - PtN3^{+} + nH_2O \rightarrow \ Cl - PtN3(H_2O)^{+}_{n} \qquad \Delta G^{*}_{clust} \qquad \text{(Eq. S7)}$$

Thus, reaction S6 can be rewritten in two steps, S8 demonstrates the interaction with the incoming molecule of water that will undergo the substitution reaction, followed by S9 that describes the path to the transition state, $Cl/H_2O - PtN3(H_2O)^{+}_{n}$.

$$Cl - PtN3(H_2O)^{+}_{n} + H_2O \overset{K_{eq}}{\rightleftharpoons} \ Cl - PtN3(H_2O)^{+}_{n+1} \qquad \Delta G_{bind} \qquad \text{(Eq. S8)}$$

$$Cl - PtN3(H_2O)^{+}_{n+1} \overset{k_1}{\rightarrow} Cl/H_2O - PtN3(H_2O)^{+}_{n} \qquad \Delta G_{bind} \qquad \text{(Eq. S9)}$$

Thus,

$$\frac{d[H_2O-PtN3(H_2O)^{+}_{n}]}{dt} = k_1[\ Cl - PtN3(H_2O)^{+}_{n+1}] \qquad \text{(Eq. S10)}$$

$$K_{eq} = \frac{[Cl-PtN3(H_2O)^{+}_{n+1}]}{[Cl-PtN3(H_2O)^{+}_{n}]+[H_2O]} = e^{-\frac{\Delta G_{bind}}{RT}} \qquad \text{(Eq. S11)}$$

Combining equations S10 and S11

$$\frac{d[H_2O-PtN3(H_2O)^{+}_{n}]}{dt} = k_1\left(e^{-\frac{\Delta G_{bind}}{RT}}\right)\ [Cl - PtN3(H_2O)^{+}_{n}][H_2O] \qquad \text{(Eq. S12)}$$

Thus, combining all constants in a term called observed rate constant, k_{obs}

$$k_{obs} = k_1[H_2O]\left(e^{-\frac{\Delta G_{bind}}{RT}}\right) \qquad \text{(Eq. S13)}$$

Employing the transition state theory for eq. S10 and the observed rate constant:

$$k_1 = \frac{k_bT}{h}e^{-\frac{\Delta G^{\ddagger}}{RT}} \qquad \text{(Eq. S14)}$$

$$k_{obs} = \frac{k_bT}{h}e^{-\frac{\Delta G^{\ddagger}_{obs}}{RT}} \qquad \text{(Eq. S15)}$$

Substituting expressions S14 and S15 in S13:

$$\frac{k_bT}{h}e^{-\frac{\Delta G^{\ddagger}_{obs}}{RT}} = \left(\frac{k_bT}{h}e^{-\frac{\Delta G^{\ddagger}}{RT}}\right)[H_2O]\left(e^{-\frac{\Delta G_{bind}}{RT}}\right)$$

Dividing both sides for $\frac{k_bT}{h}$ and applying natural logarithm and simplifying:

$$\Delta G^{\ddagger}_{obs} = \Delta G^{\ddagger} + \Delta G_{bind} - RTln[H_2O] \qquad \text{(Eq. S16)}$$

However, one may see $\Delta G^{\ddagger}_{sol} = \Delta G^{\ddagger}_{bind} + \Delta G^{\ddagger}$ as equations S8 and S9 combined takes to $Cl - PtN3(H_2O)^+_n + H_2O \rightarrow Cl/H_2O - PtN3(H_2O)^+_n$, which represents the reaction comprising explicit water molecules. Substituting this in equation S16:

$$\Delta G^{\ddagger}_{obs} = \Delta G^{\ddagger}_{sol} - RTln[H_2O] \qquad \text{(Eq. S17)}$$

Where the first term in the righthand is calculated according to expression shown in equation 2 of the manuscript.

Kinetic analysis regarding aquation-driven substitution of <u>GlcNS(6S) Model</u>

Regarding the aquation-driven reaction with GlcNS(6S), barrier heights are similar and there is no easily defined rate determining step. In this case, it was employed the stationary state approximation to equate MS2 concentration as following:

$$\frac{d[MS2]}{dt} = k_1[MS1] - k_{-1}[MS2] - k_2[MS2] = 0 \quad \text{(Eq. S18)}$$

$$[MS2] = \frac{k_1}{(k_2 + k_{-1})}[MS1] \quad \text{(Eq. S19)}$$

Which can take into account the equilibrium formed between the reactants and MS1 (Eqs. S20 and S21) to get to Eq. S22.

$$K_1 = \frac{[MS1]}{[GlcNS(6S)][Cl-PtN3]} = e^{\frac{-\Delta G_{MS1}}{RT}} \text{ (Eq. S20)}$$

$$[MS1] = K_{MS1}\,[PtN3 - Cl][H_2O] \quad \text{(Eq. S21)}$$

$$[MS2] = \frac{k_1}{(k_2 + k_{-1})} K_{MS1}\,[PtN3 - Cl][H_2O] \quad \text{(Eq. S22)}$$

MS2 is an intermediate that is in a rapid equilibrium with H2O-PtN3, in this way, the formation of $H_2O - PtN3$ can be written as a chemical equilibrium (Eq. S23), which direct product interacts with the saccharide model to form intermediates MS5 or MS5' (Eq.S24 and S25)

$$K_2 = \frac{[PtN3-H2O]}{[MS2]} = e^{\frac{-\Delta G_{(PtN3-H_2O)}}{RT}} \text{ (Eq. S23) and } K_3 = \frac{[MS5]}{[GlcNS(6S)][H_2O-PtN3]} = e^{\frac{-\Delta G_{MS5}}{RT}} \text{ (Eq. S24)}$$

$$K_{3'} = \frac{[MS5']}{[GlcNS(6S)][H_2O-PtN3]} = e^{\frac{-\Delta G_{MS5'}}{RT}} \text{ (Eq. S25)}$$

Thus, MS5 concentration can be written as

$$[MS5] = K_2K_3[GlcNS(6S)][\mathrm{MS2}] \quad \text{(Eq. S26)}$$

$$[MS5'] = K_2K_{3'}[GlcNS(6S)][\mathrm{MS2}] \quad \text{(Eq. S27)}$$

Substituting Eq. S22 in Eq. S26 and S27, we can obtain:

$$[MS5] = K_2K_3\frac{k_1}{(k_2+k_{-1})}K_{MS1}[H_2O][PtN3-Cl][GlcNS(6S)] \quad \text{(Eq. S28)}$$

$$[MS5'] = K_2K_{3'}\frac{k_1}{(k_2+k_{-1})}K_{MS1}[H_2O][PtN3-Cl][GlcNS(6S)] \quad \text{(Eq. S29)}$$

Considering the product formation is governed by the following kinetic laws,

$$\frac{d[MS6]}{dt} = k_3[MS5] \quad \text{(Eq. S30) and } \frac{d[MS6']}{dt} = k_{3'}[MS5'] \quad \text{(Eq. S31)}$$

And substituting the concentrations of MS5 and MS5’ obtained before, we have

$$\frac{d[MS6]}{dt} = k_3K_2K_3\frac{k_1}{(k_2+k_{-1})}K_{MS1}[H_2O][PtN3-Cl][GlcNS(6S)] \quad \text{(Eq. S32)}$$

And

$$\frac{d[MS6\prime]}{dt} = k_{3'}K_2K_3\frac{k_1}{(k_2+k_{-1})}K_{MS1}[H_2O][PtN3-Cl][GlcNS(6S)] \quad \text{(Eq. S33)}$$

Or simply

$$\frac{d[MS6]}{dt} = k_{obs}[PtN3-Cl][GlcNS(6S)] \quad \text{(Eq. S34)}$$

$$\frac{d[MS6\prime]}{dt} = k_{obs'}[PtN3-Cl][GlcNS(6S)] \quad \text{(Eq. S35)}$$

Kinetic analysis regarding direct substitution of <u>IdoA(2S) Model</u>

Initially, IdoA(2S) model forms a “pre-association” complex with Cl-PtN3, in a fast equilibrium:

$$K_{MSa} = \frac{[MSa]}{[IdoA(2S)][Cl-PtN3]} = e^{\frac{-\Delta G_{MSa}}{RT}} \text{ (Eq. S36); } K_{MSa'} = \frac{[MSa']}{[IdoA(2S)][Cl-PtN3]} = e^{\frac{-\Delta G_{MSa'}}{RT}} \text{ (Eq. S37)}$$

Each intermediate is consumed in the substitution step, in this way, we can write the kinetic law as a function of reactant disappearance:

$$\frac{d[Cl-PtN3]}{dt} = -k_1[MSa] - k_2[MSa'] \quad \text{(Eq. S38)}$$

Considering the equations S36 and S37 for obtaining the concentration of MSa and MSa’, we can write:

$$\frac{d[Cl-PtN3]}{dt} = (-k_1K_{MSa} - k_2K_{MSa'})[IdoA(2S)][Cl-PtN3] \quad \text{(Eq. S39)}$$

Kinetic analysis regarding direct substitution of <u>GlcNS(6S) Model</u>

For the GlcNS(6S) model, and formation of a complex with Cl-PtN3, in a fast equilibrium:

$K_{MSc} = \frac{MSc}{[\text{GlcNS(6S)}][Cl{-}PtN3]} = e^{\frac{-\Delta G_{MSc}}{RT}}$ (Eq. 40); and $K_{MSc\prime} = \frac{MSc\prime}{[\text{GlcNS(6S)}]\,[Cl{-}PtN3]} = e^{\frac{-\Delta G_{MSc\prime}}{RT}}$

(Eq. S41)

Each intermediate it is consumed in the substitution step we can write the kinetic law as a function of reagent disappearance:

$$K_{obs} = -k_1[MSc] - k_2[MSc'] \qquad \text{(Eq. S42)}$$

Considering the equations S40 and S41 for obtaining the concentration of MSc and MSc', we can write:

$$\frac{d[Cl{-}PtN3]}{dt} = (-k_1K_{MSc} - k_2K_{MSc'})[\text{GlcNS(6S)}][Cl - PtN3] \qquad \text{(Eq. S43)}$$

Figures and Tables

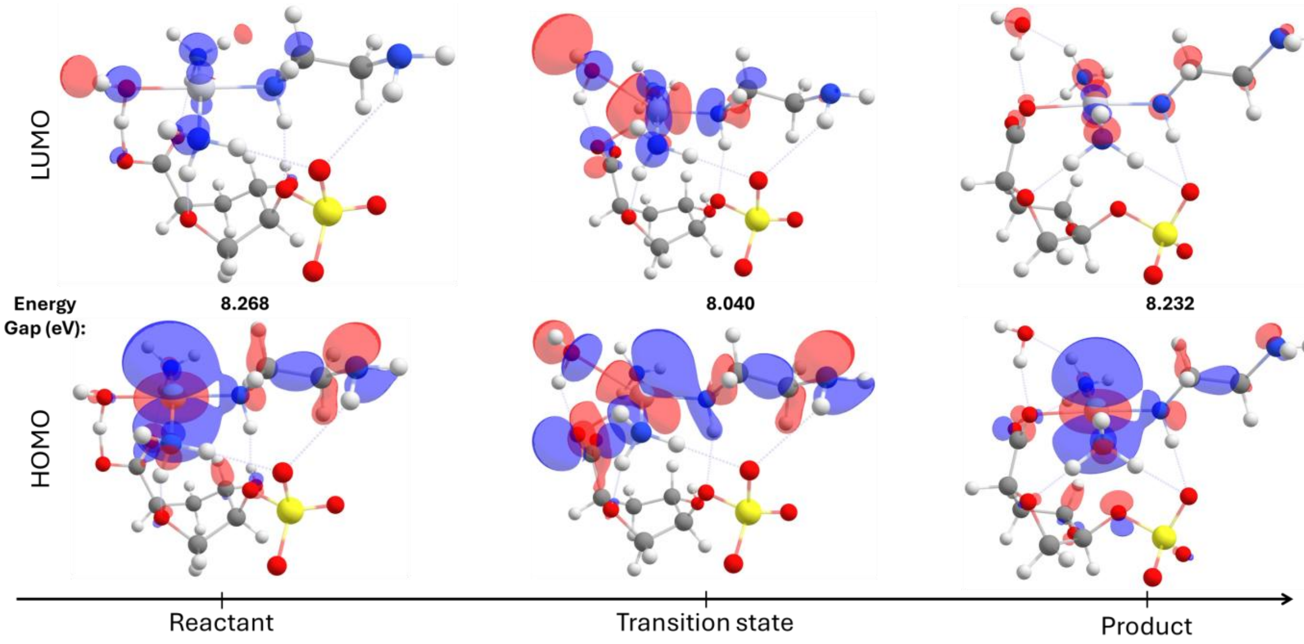

Figure S 1 - Selected molecular orbitals for stationary points and representative structures along the reaction coordinate for the water substitution by the IDOA(2S) carboxylate ligand, highlighting MS3, transition state (TS2) and the resulting MS4. (contour value = 0.025)

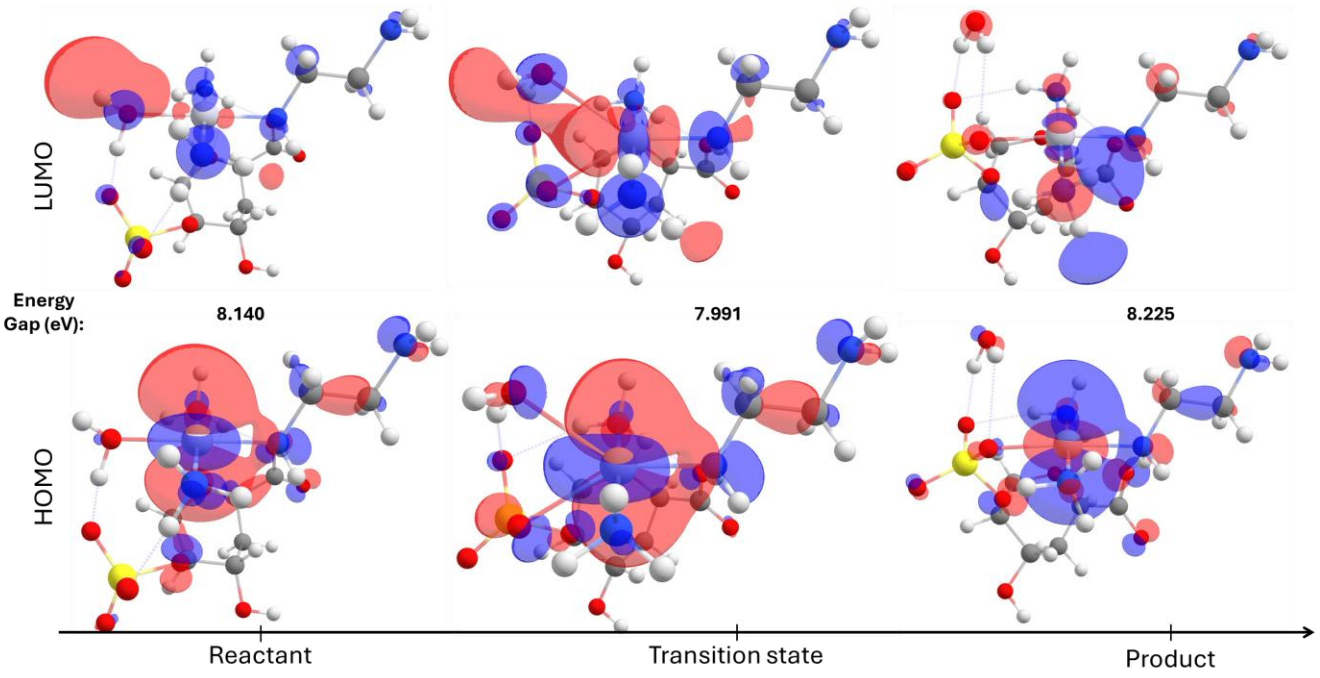

Figure S 2 - Selected molecular orbitals for stationary points and representative structures along the reaction coordinate for the water substitution by the IDOA(2S) carboxylate ligand, highlighting MS3′, transition state (TS2′) and the resulting MS4′. (contour value = 0.025)

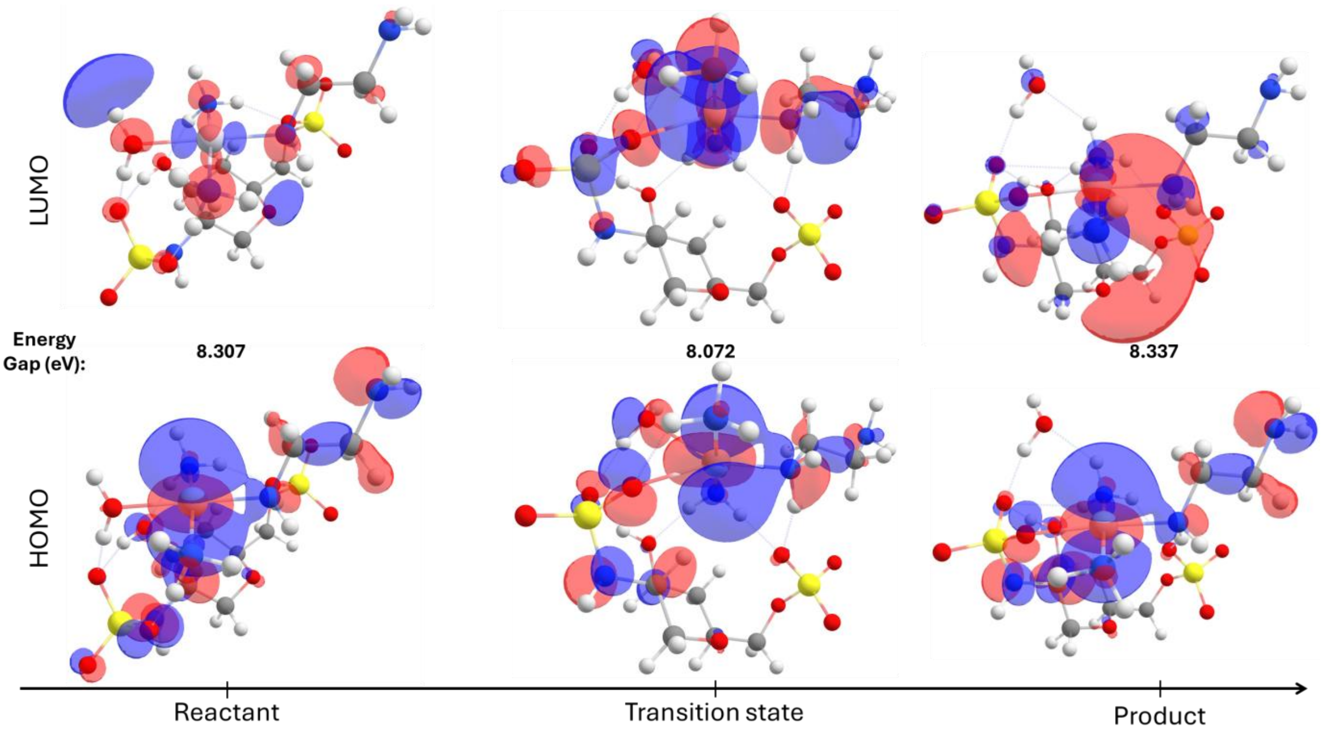

Figure S 3 - Selected molecular orbitals for stationary points and representative structures along the reaction coordinate for the water substitution by the GLCNS(6S) carboxylate ligand, highlighting MS5, transition state (TS3) and the resulting MS6. (contour value = 0.025)

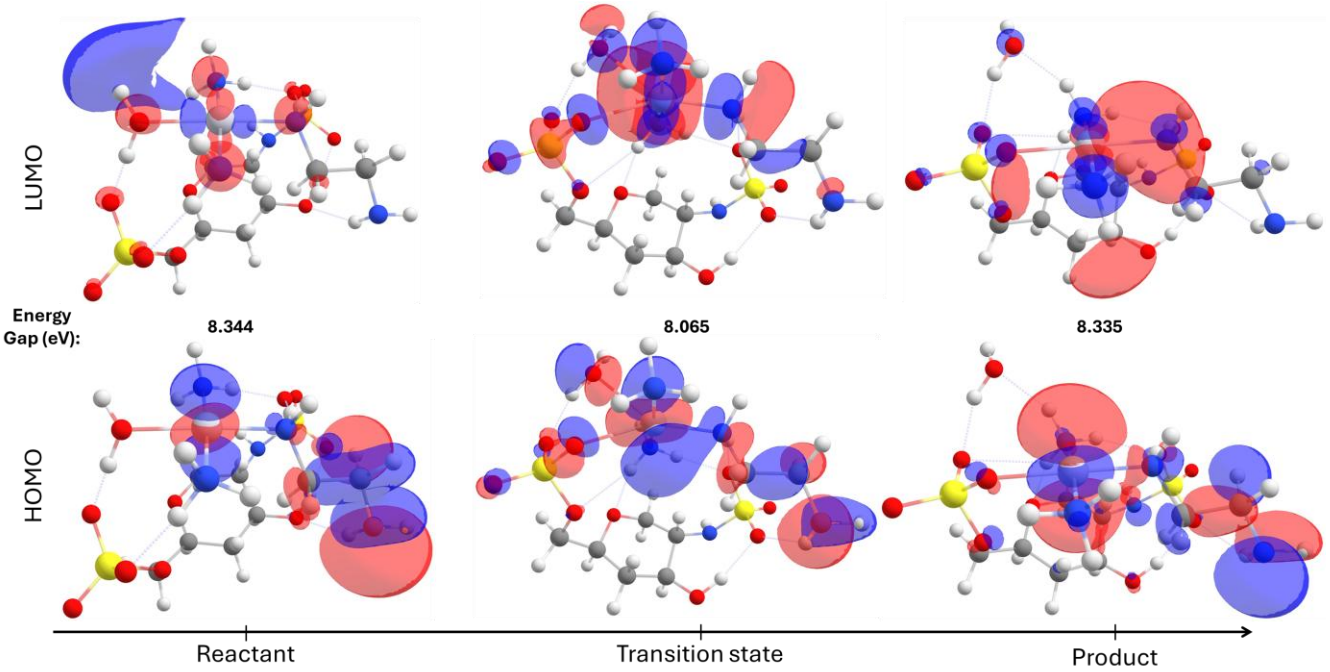

Figure S 4 - Selected molecular orbitals for stationary points and representative structures along the reaction coordinate for the water substitution by the GLCNS(6S) carboxylate ligand, highlighting MS5´, transition state (TS3´) and the resulting MS6´. (contour value = 0.025)

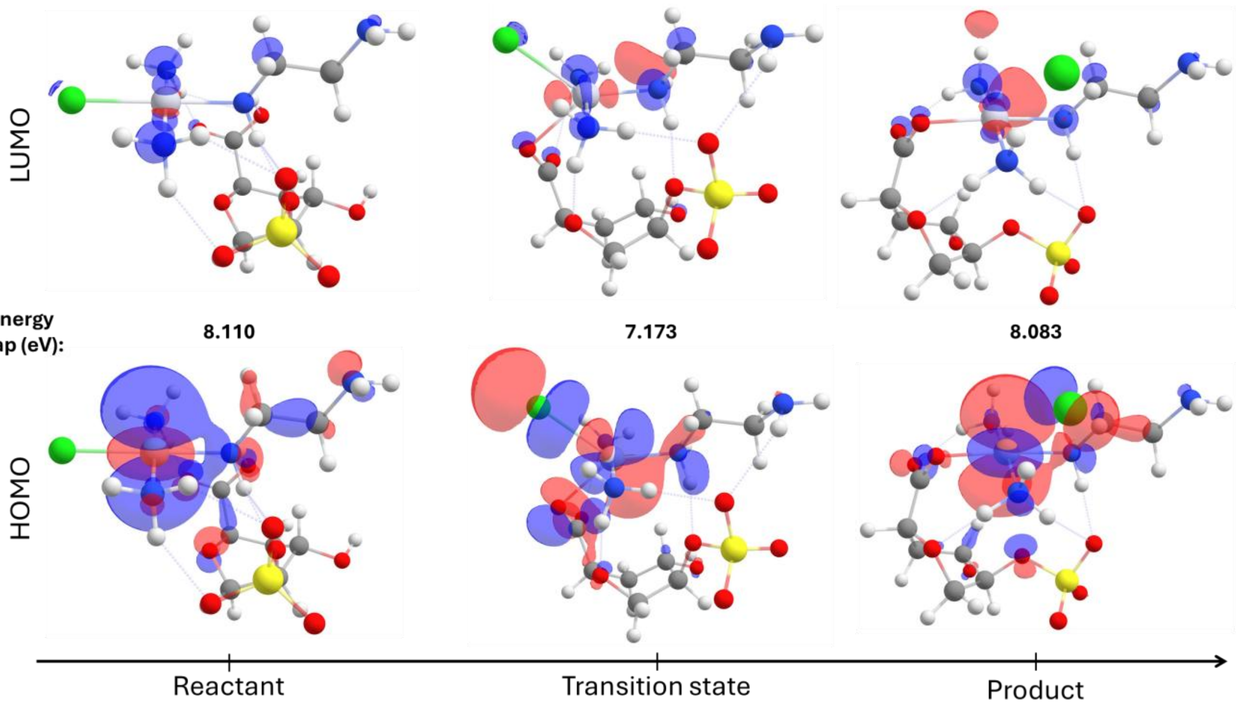

Figure S 5 - Selected molecular orbitals for stationary points and representative structures along the reaction coordinate for the chloride substitution by the IDOA(2S) carboxylate ligand, highlighting MSa, transition state (TSa) and the resulting MSb. (contour value = 0.025)

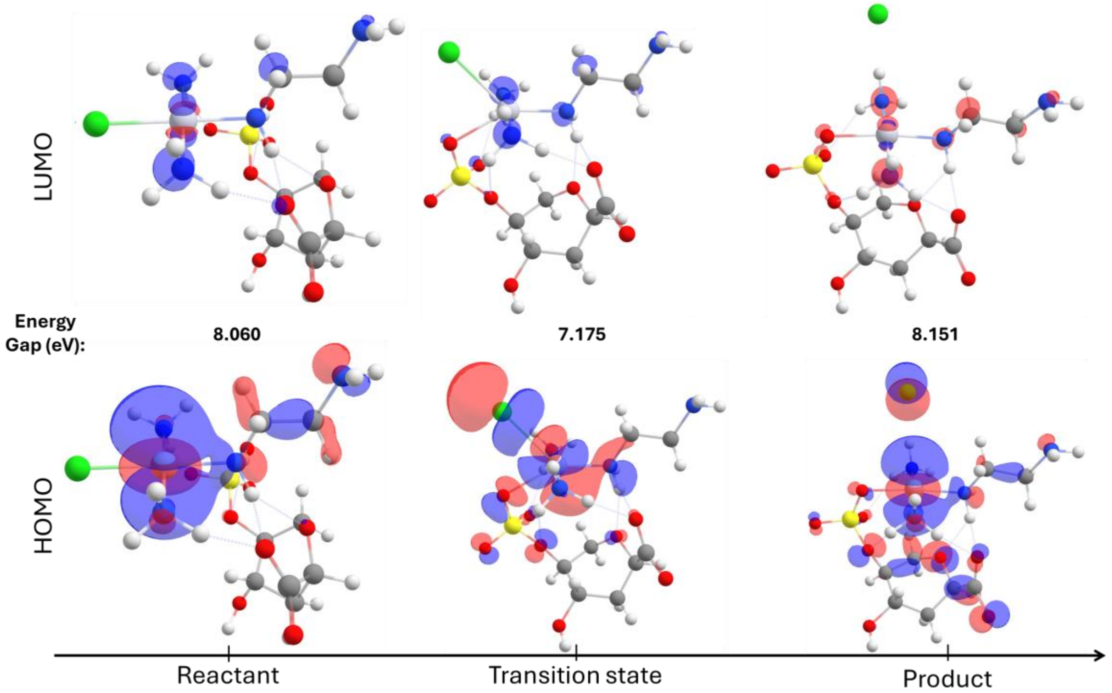

Figure S 6 - Selected molecular orbitals for stationary points and representative structures along the reaction coordinate for the chloride substitution by the IDOA(2S) carboxylate ligand, highlighting MSa′, transition state (TSa′) and the resulting MSb′. (contour value = 0.025)

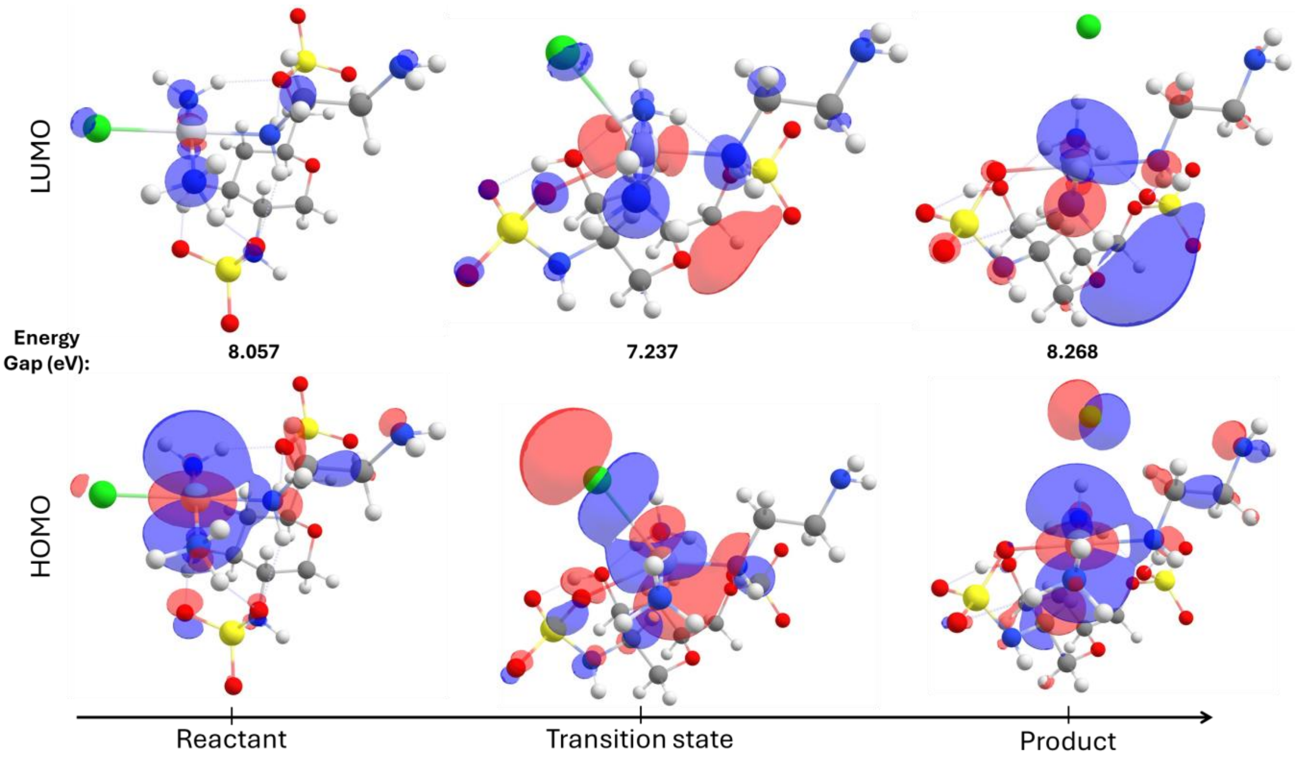

Figure S 7 - Selected molecular orbitals for stationary points and representative structures along the reaction coordinate for the chloride substitution by the GLCNS(6S) carboxylate ligand, highlighting MSc, transition state (TSb) and the resulting MSd. (contour value = 0.025)

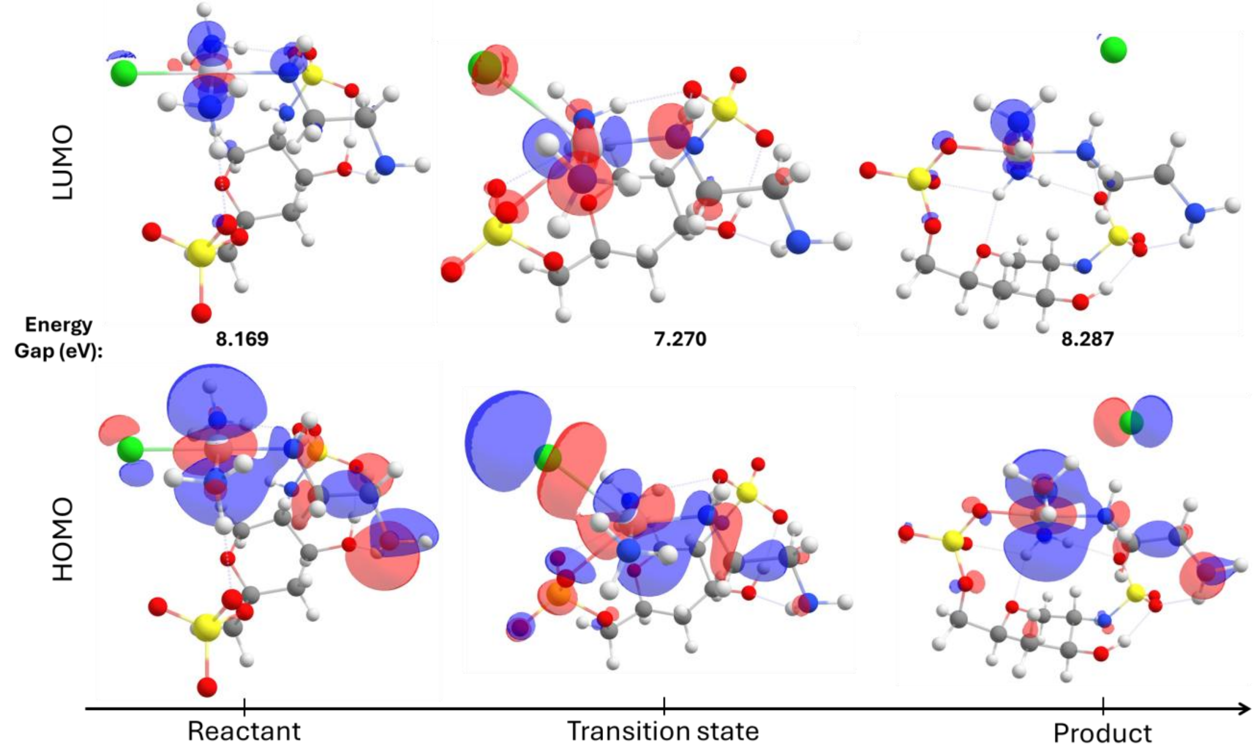

Figure S 8 - Selected molecular orbitals for stationary points and representative structures along the reaction coordinate for the chloride substitution by the GLCNS(6S) carboxylate ligand, highlighting MSc′, transition state (TSb′) and the resulting MSd′. (contour value = 0.025)

Table S 1. Gibbs free energy of reaction and activation processes for the IdoA(2S) monosaccharide model in relation to the starting material (Cl-PtN3) in different computational protocols.

Process	ΔG_{sol} BHandH	ΔG_{sol} wb97xd	ΔG_{sol} HF	ΔG_{sol} B3LYP	ΔG_{sol} wb97m-v	ΔG_{sol} M06	ΔG_{sol} r2SCAN	ΔG_{sol} CAM-B3LYP	ΔG_{sol} PBE0	ΔG_{sol} SOGGA11X	ΔG_{sol} **B2PLYP**	ΔG_{sol} DLPNO-CCSD(T)
Cl-PtN3 → Cl-PtN3	0.00	0.00	0.00	0.00	0.00	0.00	0.00	0.00	0.00	0.00	0.00	0.00
Cl-PtN3 + H_2O → MS1	-1.14	3.21	9.14	5.26	5.15	3.51	4.96	3.94	3.99	4.62	4.46	4.89
Cl-PtN3 + H_2O → TS1	23.13	27.60	31.20	29.88	26.06	26.90	27.11	28.22	29.02	29.06	29.25	29.05
Cl-PtN3 + H_2O → MS2	4.64	9.79	13.21	11.11	11.39	12.95	10.40	9.35	10.98	11.82	11.22	11.59
Cl-PtN3 + H_2O → H_2O-PtN3 + Cl^-	19.29	19.16	12.35	19.71	17.47	22.99	17.89	18.74	21.52	19.98	20.90	21.46
Cl-PtN3 + H_2O + IDOA(2S) → MS3 + Cl^-	-11.18	-1.10	25.05	17.77	7.71	6.95	10.98	10.36	12.34	13.04	10.53	9.12
Cl-PtN3 + H_2O + IDOA(2S) → TS2 + Cl^-	8.54	16.95	41.32	36.14	24.16	23.10	29.07	29.35	30.76	29.96	28.40	26.92
Cl-PtN3 + H_2O + IDOA(2S) → MS4 + Cl^-	-13.17	-5.36	12.44	10.98	4.06	2.20	7.88	4.95	6.51	6.37	4.38	4.19
Cl-PtN3 + IDOA(2S) → IDOA(2S)-CO-PtN3 + Cl^-	-10.57	-7.38	4.26	6.03	0.17	-0.41	3.94	1.71	3.03	2.37	0.85	0.39
Cl-PtN3 + H_2O + IDOA(2S) → MS3' + Cl^-	-12.68	-4.16	13.76	9.74	5.75	4.45	8.03	4.26	6.24	7.23	5.52	7.15
Cl-PtN3 + H_2O + IDOA(2S) → TS2' + Cl^-	9.91	18.04	34.39	31.82	23.72	23.15	27.38	26.50	27.87	27.45	26.50	27.96
Cl-PtN3 + H_2O + IDOA(2S) → MS4' + Cl^-	-3.19	3.59	18.95	17.48	23.11	11.23	15.19	12.38	14.00	14.14	12.10	14.23
Cl-PtN3 + IDOA(2S)→ IDOA(2S)-SO-PtN3 + Cl^-	-4.07	-0.36	9.72	9.91	6.46	6.27	9.27	6.45	7.84	7.75	6.14	8.36

ΔG was calculated with In-solution Gibbs free energy according to equation 2 in the methodology section of the article with E_{BO} of each protocol in the columns. Values given in kcal mol $^{-1}$.

Table S 2. Step deviation and mean absolute deviation (MAD) of Gibbs free energy between the DFT functionals and the reference DLPNO-CCSD(T) for the IdoA(2S) monosaccharide model reaction in relation to the starting material.

**Process**	ΔG_{sol} **BHandH**	ΔG_{sol} **Wb97XD**	ΔG_{sol} **HF**	ΔG_{sol} **B3LYP**	ΔG_{sol} **WB97M-V**	ΔG_{sol} **M06**	ΔG_{sol} **r2SCAN**	ΔG_{sol} **CAM-B3LYP**	ΔG_{sol} **PBE0**	ΔG_{sol} **SOGGA11X**	ΔG_{sol} **B2PLYP**
Cl-PtN3 + **H_2O** → MS1	6.03	1.68	4.25	0.37	0.26	1.38	0.07	0.95	0.9	0.27	0.43
Cl-PtN3 + **H_2O** → TS1	5.92	1.45	2.15	0.83	2.99	2.15	1.94	0.83	0.03	0.01	0.2
Cl-PtN3 + **H_2O** → MS2	6.95	1.8	1.62	0.48	0.2	1.36	1.19	2.24	0.61	0.23	0.37
Cl-PtN3 + **H_2O** → H_2O-PtN3 + **Cl^-**	2.17	2.3	9.11	1.75	3.99	1.53	3.57	2.72	0.06	1.48	0.56
Cl-PtN3 + **H_2O** + IDOA(2S) → MS3 + **Cl^-**	20.3	10.22	15.93	8.65	1.41	2.17	1.86	1.24	3.22	3.92	1.41
Cl-PtN3 + **H_2O** + IDOA(2S) → TS2 + **Cl^-**	18.38	9.97	14.4	9.22	2.76	3.82	2.15	2.43	3.84	3.04	1.48
Cl-PtN3 + **H_2O** + IDOA(2S) → MS4 + **Cl^-**	17.36	9.55	8.25	6.79	0.13	1.99	3.69	0.76	2.32	2.18	0.19
Cl-PtN3 + IDOA(2S) → IDOA(2S)-CO-PtN3 + **Cl^-**	10.96	7.77	3.87	5.64	0.22	0.8	3.55	1.32	2.64	1.98	0.46
Cl-PtN3 + **H_2O** + IDOA(2S) → MS3’ + **Cl^-**	19.83	11.31	6.61	2.59	1.4	2.7	0.88	2.89	0.91	0.08	1.63
Cl-PtN3 + **H_2O** + IDOA(2S) → TS2’ + **Cl^-**	18.05	9.92	6.43	3.86	4.24	4.81	0.58	1.46	0.09	0.51	1.46
Cl-PtN3 + **H_2O** + IDOA(2S) → MS4’ + **Cl^-**	17.42	10.64	4.72	3.25	8.88	3	0.96	1.85	0.23	0.09	2.13
Cl-PtN3 + IDOA(2S)→ IDOA(2S)-SO-PtN3 + **Cl^-**	12.43	8.72	1.36	1.55	1.9	2.09	0.91	1.91	0.52	0.61	2.22
**MAD**	**12.98**	**7.11**	**6.56**	**3.75**	**2.37**	**2.32**	**1.78**	**1.72**	**1.28**	**1.20**	**1.05**

ΔG was calculated with In-solution Gibbs free energy according to equation 2 in the methodology section of the article with E_{BO} of each protocol in the columns. The colour indicates the deviation to the DLPNO-CCSD(T) result: The closer the value is to the reference the greener it is, the farther the result the redder it gets. Values given in kcal mol $^{-1}$.

Table S 3. Gibbs free energy of reaction and activation processes for the IdoA(2S) monosaccharide model in relation to each separate reaction step in different computational protocols.

**Process**	ΔG_{sol} BHandH	ΔG_{sol} wb97xd	ΔG_{sol} HF	ΔG_{sol} B3LYP	ΔG_{sol} wb97m-v	ΔG_{sol} M06	ΔG_{sol} r2SCAN	ΔG_{sol} CAMB3LYP	ΔG_{sol} PBE0	ΔG_{sol} SOGGA11X	ΔG_{sol} **B2PLYP**	ΔG_{sol} DLPNO-CCSD(T)	ΔG_{sol} Exp.
Cl-PtN3 + H_2O → MS1	-1.14	3.21	9.14	5.26	5.15	3.51	4.96	3.94	3.99	4.62	4.46	4.89	-
MS1→TS1	24.27	24.39	22.06	24.63	20.91	23.39	22.15	24.28	25.03	24.44	24.79	24.16	24.1[a]
TS1→ MS2	-18.49	-17.81	-17.98	-18.77	-14.67	-13.96	-16.71	-18.87	-18.04	-17.25	-18.03	-17.46	-19.4[a]
MS2 → H2O-PtN3 + Cl^-	14.64	9.36	-0.86	8.60	6.08	10.04	7.49	9.38	10.54	8.16	9.68	9.87	-
H_2O-PtN3 + IDOA(2S) → MS3	-30.46	-20.25	12.70	-1.95	-9.77	-16.04	-6.91	-8.38	-9.19	-6.94	-10.37	-12.34	-
MS3 →TS2*	19.72	16.95	16.27	18.38	16.45	16.15	18.09	18.99	18.42	16.92	17.87	17.80	-
TS2→MS4	-21.71	-1.20	-28.88	-25.16	-20.09	-20.90	-21.19	-24.40	-24.25	-23.59	-24.02	-22.72	-
MS4 → IDOA(2S)-CO-PtN3 + H2O	2.60	-25.43	-8.18	-4.95	-3.89	-2.61	-3.94	-3.24	-3.48	-4.00	-3.53	-3.80	-
H2O-PtN3 + IDOA(2S) → MS3'	-31.96	-23.31	1.41	-9.98	-11.72	-18.54	-9.87	-14.47	-15.28	-12.74	-15.38	-14.31	-
MS3' →TS2'*	22.59	18.04	20.64	22.08	17.97	18.70	19.35	22.24	21.63	20.21	20.98	20.81	-
TS2'→MS4'	-13.10	7.74	-15.45	-14.34	-0.61	-11.92	-12.19	-14.12	-13.87	-13.31	-14.40	-13.73	-
MS4' → IDOA(2S)-SO-PtN3 + H2O	-0.88	-18.40	-9.23	-7.57	-16.65	-4.96	-5.92	-5.93	-6.17	-6.39	-5.96	-5.87	-

ΔG was calculated with In-solution Gibbs free energy according to equation 2 in the methodology section of the article with E_{BO} of each protocol in the columns. [a] Mean ΔG values obtained experimentally by Gorle and collaborators in 2023 and 2019 in different kinetic models. Values given in kcal mol $^{-1}$.

Table S 4. Enthalpy of reaction and activation processes for the IdoA(2S) monosaccharide model in relation to the starting material (Cl-PtN3) in different computational protocols.

Species	ΔH_{sol} BHandH	ΔH_{sol} wb97xd	ΔH_{sol} HF	ΔH_{sol} B3LYP	ΔH_{sol} wb97m-v	ΔH_{sol} M06	ΔH_{sol} r2SCAN	ΔH_{sol} CAMB3LYP	ΔH_{sol} PBE0	ΔH_{sol} SOGGA11X	ΔH_{sol} B2PLYP	ΔH_{sol} DLPNO-CCSD(T)
Cl-PtN3 → Cl-PtN3	0.00	0.00	0.00	0.00	0.00	0.00	0.00	0.00	0.00	0.00	0.00	0.00
Cl-PtN3 → MS1	-8.45	-4.10	1.83	-2.05	-2.15	-3.79	-2.35	-3.36	-3.31	-2.69	-2.85	-2.42
Cl-PtN3 → TS1	14.91	19.38	22.98	21.66	17.84	18.68	18.89	20.00	20.80	20.84	21.03	20.83
Cl-PtN3 → MS2	-4.27	0.88	4.30	2.20	2.48	4.04	1.49	0.45	2.07	2.91	2.31	2.68
Cl-PtN3 → H_2O-PtN3	17.17	17.04	10.24	17.60	15.36	20.88	15.78	16.62	19.41	17.86	18.79	19.34
Cl-PtN3 → MS3	-30.33	-20.25	5.90	-1.38	-11.44	-12.20	-8.17	-8.79	-6.82	-6.11	-8.62	-10.03
Cl-PtN3 → TS2	-10.82	-2.41	21.96	16.78	4.80	3.74	9.71	9.99	11.40	10.60	9.04	7.56
Cl-PtN3 → MS4	-29.07	-21.26	-3.47	-4.92	-11.84	-13.71	-8.03	-10.95	-9.39	-9.53	-11.53	-11.71
Cl-PtN3 → IDOA(2S)-CO-PtN3	-19.35	-16.17	-4.53	-2.75	-8.61	-9.19	-4.84	-7.07	-5.75	-6.41	-7.93	-8.39
Cl-PtN3 → MS3’	-30.28	-21.76	-3.84	-7.86	-11.85	-13.15	-9.57	-13.34	-11.36	-10.37	-12.08	-10.45
Cl-PtN3 → TS2’	-6.76	1.37	17.72	15.15	7.05	6.48	10.71	9.83	11.20	10.78	9.83	11.29
Cl-PtN3 → MS4’	-18.49	-11.72	3.64	2.17	7.81	-4.07	-0.11	-2.92	-1.30	-1.16	-3.20	-1.07
Cl-PtN3 → IDOA(2S)-SO-PtN3	-11.92	-8.21	1.86	2.06	-1.39	-1.59	1.42	-1.41	-0.02	-0.10	-1.71	0.51

ΔG was calculated with In-solution Gibbs free energy according to equation 2 in the methodology section of the article with E_{BO} of each protocol in the columns. Values given in kcal mol $^{-1}$.

Table S 5. Absolute enthalpy deviation between the DFT functionals and the reference DLPNO-CCSD(T) for the IdoA(2S) monosaccharide model reaction in relation to the starting material.

Species	ΔH_{sol} BHandH	ΔH_{sol} Wb97XD	ΔH_{sol} HF	ΔH_{sol} B3LYP	ΔH_{sol} WB97M-V	ΔH_{sol} M06	ΔH_{sol} r2SCAN	ΔH_{sol} CAM-B3LYP	ΔH_{sol} PBE0	ΔH_{sol} SOGGA11X	ΔH_{sol} B2PLYP
Cl-PtN3 → **MS1**	6.03	1.68	4.25	0.37	0.27	1.37	0.07	0.94	0.89	0.27	0.43
Cl-PtN3 → **TS1**	5.92	1.45	2.15	0.83	2.99	2.15	1.94	0.83	0.03	0.01	0.20
Cl-PtN3 → **MS2**	6.95	1.80	1.62	0.48	0.20	1.36	1.19	2.23	0.61	0.23	0.37
Cl-PtN3 → **H_2O-PtN3**	2.17	2.30	9.10	1.74	3.98	1.54	3.56	2.72	0.07	1.48	0.55
Cl-PtN3 → **MS3**	20.30	10.22	15.93	8.65	1.41	2.17	1.86	1.24	3.21	3.92	1.41
Cl-PtN3 → **TS2**	18.38	9.97	14.40	9.22	2.76	3.82	2.15	2.43	3.84	3.04	1.48
Cl-PtN3 → **MS4**	17.36	9.55	8.24	6.79	0.13	2.00	3.68	0.76	2.32	2.18	0.18
Cl-PtN3 → **IDOA(2S)-CO-PtN3**	10.96	7.78	3.86	5.64	0.22	0.80	3.55	1.32	2.64	1.98	0.46
Cl-PtN3 → **MS3’**	19.83	11.31	6.61	2.59	1.40	2.70	0.88	2.89	0.91	0.08	1.63
Cl-PtN3 → **TS2’**	18.05	9.92	6.43	3.86	4.24	4.81	0.58	1.46	0.09	0.51	1.46
Cl-PtN3 → **MS4’**	17.42	10.65	4.71	3.24	8.88	3.00	0.96	1.85	0.23	0.09	2.13
Cl-PtN3 → **IDOA(2S)-SO-PtN3**	12.43	8.72	1.35	1.55	1.90	2.10	0.91	1.92	0.53	0.61	2.22
**Mean error**	12.98	7.11	6.55	3.75	2.37	2.32	1.78	1.72	1.28	1.20	1.04

ΔG was calculated with In-solution Gibbs free energy according to equation 2 in the methodology section of the article with E_{BO} of each protocol in the columns. The colour indicates the deviation to the DLPNO-CCSD(T) result: The closer the value is to the reference the greener it is, the farther the result the redder it gets. Values given in kcal mol $^{-1}$.

Table S 6. Enthalpy of reaction and activation processes for the IdoA(2S) monosaccharide model in relation to each separate reaction step in different computational protocols.

**Process**	ΔH_{sol} BHandH	ΔH_{sol} wb97xd	ΔH_{sol} HF	ΔH_{sol} B3LYP	ΔH_{sol} wb97m-v	ΔH_{sol} M06	ΔH_{sol} r2SCAN	ΔH_{sol} CAMB3LYP	ΔH_{sol} PBE0	ΔH_{sol} SOGGA11X	ΔH_{sol} B2PLYP	ΔH_{sol} DLPNO-CCSD(T)
Cl-PtN3 + H_2O → MS1	-8.45	-4.10	1.83	-2.05	-2.15	-3.79	-2.35	-3.36	-3.31	-2.69	-2.85	-2.42
MS1→TS1	23.36	23.48	21.15	23.71	19.99	22.48	21.24	23.37	24.12	23.53	23.88	23.25
TS1→ MS2	-19.18	-18.50	-18.67	-19.46	-15.36	-14.65	-17.40	-19.56	-18.73	-17.94	-18.72	-18.15
MS2 → H2O-PtN3 + Cl^-	21.44	16.16	5.93	15.40	12.88	16.84	14.28	16.18	17.34	14.95	16.48	16.66
H_2O-PtN3 + Dis2 → MS3	-47.50	-37.29	-4.34	-18.98	-26.80	-33.08	-23.95	-25.42	-26.22	-23.98	-27.41	-29.38
MS3 →TS2*	19.51	-2.41	16.06	18.17	16.24	15.94	17.88	18.78	18.22	16.71	17.67	17.59
TS2→MS4	-18.25	0.49	-25.42	-21.71	-16.64	-17.44	-17.74	-20.94	-20.80	-20.13	-20.57	-19.27
MS4 → IDOA(2S)-CO-PtN3 + H2O	9.72	-17.54	-1.06	2.17	3.23	4.52	3.18	3.88	3.65	3.12	3.60	3.32
H2O-PtN3 + Dis2 → MS3’	-47.45	-38.80	-14.08	-25.46	-27.21	-34.02	-25.35	-29.96	-30.77	-28.23	-30.86	-29.79
MS3’ →TS2’*	23.52	1.37	21.57	23.01	18.90	19.63	20.28	23.17	22.56	21.14	21.91	21.74
TS2’→MS4’	-11.73	10.04	-14.08	-12.97	0.75	-10.55	-10.82	-12.76	-12.50	-11.94	-13.03	-12.37
MS4’ → IDOA(2S)-SO-PtN3 + H2O	6.57	-9.58	-1.78	-0.12	-9.20	2.48	1.53	1.51	1.28	1.06	1.49	1.58

ΔG was calculated with In-solution Gibbs free energy according to equation 2 in the methodology section of the article with E_{BO} of each protocol in the columns. Values given in kcal mol $^{-1}$.

Table S 7. Reaction and activation thermodynamic properties for the GlcNS(6S) monosaccharide model in relation to the starting material (Cl-PtN3).

**Species**	ΔG_{sol} B2PLYP	ΔH_{sol} B2PLYP
Cl-PtN3 → Cl-PtN3	0.00	0.00
Cl-PtN3 → MS1	4.46	-2.85
Cl-PtN3 → TS1	29.25	21.03
Cl-PtN3 → MS2	11.22	2.31
Cl-PtN3 → H_2O-PtN3	20.90	18.79
Cl-PtN3 → MS5	0.01	0.04
Cl-PtN3 → TS3	29.21	43.71
Cl-PtN3 → MS6	11.63	29.70
Cl-PtN3 → GlcNS(6S)-NS-PtN3	6.60	31.09
Cl-PtN3 → MS5'	9.93	23.67
Cl-PtN3 → TS3'	30.09	44.54
Cl-PtN3 → MS6'	12.15	27.18
Cl-PtN3 → GlcNS(6S)-OS-PtN3	4.76	29.16

ΔG was calculated with In-solution Gibbs free energy according to equation 2 in the methodology section of the article. Values given in kcal mol $^{-1}$.

Table S 8. Reaction and activation thermodynamic properties for the GlcNS(6S) monosaccharide model.

**Process**	ΔG_{sol} B2PLYP	ΔH_{sol} B2PLYP	ΔG_{sol}Exp.
Cl-PtN3 + H_2O → MS1	4.46	-2.85	-
MS1→TS1	24.79	23.88	24.1[a]
TS1→ MS2	-18.03	-18.72	-19.4[a]
MS2 → H2O-PtN3 + Cl^-	9.68	16.48	-
H_2O-PtN3 + Dis1 → MS5	-20.89	-18.75	-
MS5 →TS3*	29.20	43.67	21.7[a]
TS3→MS6	-17.57	-14.01	-22.7[a]
MS6 → GlcNS(6S)-NS-PtN3+ H2O	-5.04	1.39	-
H2O-PtN3 + Dis1 → MS5’	-10.97	4.89	-
MS5’ →TS3’*	20.16	20.87	-
TS3’→MS6’	-17.95	-17.37	-
MS6’ → GlcNS(6S)-OS-PtN3+ H2O	-7.38	1.98	-

ΔG was calculated with In-solution Gibbs free energy according to equation 2 in the methodology section of the article with E_{BO} of each protocol in the columns. [a] Mean ΔG values obtained experimentally by Gorle and collaborators in 2023 and 2019 in different kinetic models. Values given in kcal mol $^{-1}$.

Table S 9. Frontier orbitals energy for the aquation process (in eV).

	AQUATION		
	R(MS1)	TS1	P(MS2)
LUMO	0.083792	-0.13482	0.014471
HOMO	-8.27041	-7.63007	-8.40807

Table S 10. Frontier orbitals energy for the IDOA(2S) model substitution after the aquation process (in eV).

	R(MS3)	TS2	P(MS4)	R(MS3´)	TS2'	P(MS4´)
LUMO	0.181239	0.134109	0.248765	0.160772	0.115283	0.181239
HOMO	-8.08668	-7.90488	-7.98276	-7.97973	-7.87558	-8.05494

Table S 11. Frontier orbitals energy for the IDOA(2S) model substitution after the aquation process (in eV).

	R(MS5)	TS3	P(MS6)	R(MS5´)	TS3'	P(MS6´)
LUMO	0.191884	0.127974	0.183387	0.143659	0.114392	0.212863
HOMO	-8.11485	-7.94451	-8.15385	-8.19993	-7.95051	-8.12246

Table S 12. Frontier orbitals energy for the direct GLCNS(6S) model substitution (in eV).

	R(MSA)	TSA	P(MSB)	R(MSA´)	TSA'	P(MSB´)
LUMO	0.312241	0.28555	0.29512	0.288031	0.272672	0.298618
HOMO	-7.79803	-6.88785	-7.78794	-7.77215	-6.90225	-7.85196

Table S 13. Frontier orbitals energy for the direct GLCNS(6S) model substitution (in eV).

	R(MSC)	TSB	P(MSD)	R(MSC´)	TSB'	P(MSD´)
LUMO	0.292019	0.246578	0.255658	0.162421	0.252092	0.333944
HOMO	-7.76507	-6.98997	-8.01261	-8.0091	-7.01746	-7.95267

Table S 14. Topological parameters of the electron density calculated at the bond critical points (BCPs) for the reactant species (MS1). Electron density (ρ, a.u.) and Laplacian of the electron density ($\nabla^2\rho$, a.u.) are provided.

Species/ QTAIM	O23-H8		H8-N4		O23-H25		H25-Cl22		N4-Pt		Cl22-Pt	
	ρ	$\nabla^2\rho$	ρ	$\nabla^2\rho$	ρ	$\nabla^2\rho$	ρ	$\nabla^2\rho$	ρ	$\nabla^2\rho$	ρ	$\nabla^2\rho$
Reactant	0.0365	0.1128	0.3200	-1.7488	0.3498	-2.2341	0.02255	0.0635	0.1171	0.4837	0.0878	0.2596

Table S 15. Topological parameters of the electron density calculated at the bond critical points (BCPs) for the transition state species (TS1). Electron density (ρ, a.u.) and Laplacian of the electron density ($\nabla^2\rho$, a.u.) are provided.

Species/ QTAIM	O23-H25		Cl22-H25		Cl22-Pt		N6-Pt		N4-Pt		O23-Pt	
	ρ	$\nabla^2\rho$	ρ	$\nabla^2\rho$	ρ	$\nabla^2\rho$	ρ	$\nabla^2\rho$	ρ	$\nabla^2\rho$	ρ	$\nabla^2\rho$
TS	0.3441	-2.2546	0.0273	0.0847	0.0449	0.1346	0.1156	0.5013	0.1174	0.5043	0.0539	0.2385

Table S 16. Topological parameters of the electron density calculated at the bond critical points (BCPs) for the product species (MS2). Electron density (ρ, a.u.) and Laplacian of the electron density ($\nabla^2\rho$, a.u.) are provided.

Species/ QTAIM	O23-H25		Cl22-H25		Cl22-H11		H11-N4		N4-Pt		O23-Pt	
	ρ	$\nabla^2\rho$	ρ	$\nabla^2\rho$	ρ	$\nabla^2\rho$	ρ	$\nabla^2\rho$	ρ	$\nabla^2\rho$	ρ	$\nabla^2\rho$
Product	0.3033	-1.8255	0.0492	0.0841	0.0261	0.0645	0.3222	-1.7559	0.1173	0.4847	0.0967	0.5772

Table S 17. Reaction and activation thermodynamic properties for the direct substitution of IdoA(2S) monosaccharide model in relation to the starting material (Cl-PtN3).

Process	ΔG_{sol} B2PLYP	ΔH_{sol} B2PLYP
Cl-PtN3 → Cl-PtN3	0.00	0.00
Cl-PtN3 → MSa	-4.92	-18.65
Cl-PtN3 → TSa	23.40	7.07
Cl-PtN3 → MSb	0.80	-12.32
Cl-PtN3 → IDOA(2S)-CO-PtN3	0.85	-7.93
Cl-PtN3 → MSa'	-8.92	-22.17
Cl-PtN3 → TSa'	19.32	5.27
Cl-PtN3 → MSb'	6.51	-6.68
Cl-PtN3 → IDOA(2S)-SO-PtN3	6.14	-1.71

ΔG was calculated with In-solution Gibbs free energy according to equation 2 in the methodology section of the article with E_{BO} of each protocol in the columns. Values given in kcal mol $^{-1}$.

Table S 18. Reaction and activation thermodynamic properties for the direct substitution of IdoA(2S) monosaccharide model.

**Process**	ΔG_{sol} B2PLYP	ΔH_{sol} B2PLYP
Cl-PtN3 + Dis1 → MSa	-4.92	-18.65
MSa → TSa	28.32	25.72
TSa → MSb	-22.61	-19.39
MSb → IDOA(2S)-CO-PtN3 + Cl^-	0.05	4.38
Cl-PtN3 + Dis1 → MSa'	-8.92	-22.17
MSa' → TSa'	28.24	27.44
TSa' → MSb'	-12.82	-11.94
MSb' → IDOA(2S)-SO-PtN3 + Cl^-	-0.36	4.97

ΔG was calculated with In-solution Gibbs free energy according to equation 2 in the methodology section of the article with E_{BO} of each protocol in the columns. Values given in kcal mol $^{-1}$.

Table S 19. Reaction and activation thermodynamic properties for the direct substitution of GlcNS(6S) monosaccharide model in relation to the starting material (Cl-PtN3).

Process	ΔG_{sol} B2PLYP	ΔH_{sol} B2PLYP
Cl-PtN3 + dis1 → Cl-PtN3 + dis1	0.00	0.00
Cl-PtN3 + dis1 → MSc	-2.64	-17.45
Cl-PtN3 + dis1 → TSb	23.58	9.30
Cl-PtN3 + dis1 → MSd	9.69	-4.71
Cl-PtN3 + dis1 → GlcNS(6S)-NS-PtN3 + Cl^-	6.60	-1.61
Cl-PtN3 + dis1 → MSc'	6.11	-10.10
Cl-PtN3 + dis1 → TSb'	25.37	10.08
Cl-PtN3 + dis1 → MSd'	5.90	-9.58
Cl-PtN3 + dis1 → GlcNS(6S)-OS-PtN3 + Cl^-	4.76	-3.54

ΔG was calculated with In-solution Gibbs free energy according to equation 2 in the methodology section of the article with E_{BO} of each protocol in the columns. Values given in kcal mol $^{-1}$.

Table S 20. Reaction and activation thermodynamic properties for the direct substitution of GlcNS(6S) monosaccharide model.

Process	ΔG_{sol} B2PLYP	ΔH_{sol} B2PLYP
Cl-PtN3 + dis1 → MSc	-2.64	-17.45
MSc → TSb	26.22	26.75
TSb → MSd	-13.89	-14.01
MSd → GlcNS(6S)-NS-PtN3 + Cl^-	-3.10	3.10
Cl-PtN3 + dis1 → MSc'	6.11	-10.10
MSc' → TSb'	19.26	20.18
TSb' → MSd'	-19.48	-19.67
MSd' → GlcNS(6S)-OS-PtN3 + Cl^-	-1.13	6.04

ΔG was calculated with In-solution Gibbs free energy according to equation 2 in the methodology section of the article with E_{BO} of each protocol in the columns. Values given in kcal mol $^{-1}$.

Table S 21. Structural parameters for the minimum structures studied in this manuscript. All values are reported in terms of Angstroms (Å)

			IDOA(2S) Model		***GlcNS(6S) Model***	
	**Cl-PtN3**	**H2O-PtN3**	**IDOA(2S)-CO-PtN3**	**IDOA(2S)-SO-PtN3**	**GlcNS(6S)-NS-PtN3**	**GlcNS(6S)-OS-PtN3**
d(Pt-L)	2.31	2.06	2.03	2.07	2.05	2.05
d(Pt-NH3[N4])	2.04	2.05	2.04	2.04	2.04	2.04
d(Pt-NH3[N6])	2.04	2.04	2.03	2.04	2.04	2.04
d(Pt-EDA)	2.05	2.01	2.02	2.01	2.01	2.02
a(L-Pt-EDA)	178.4	178.3	174.9	175.2	176.3	175.4
a(NH3-Pt-NH3)	176.7	176.6	174.0	178.8	177.3	177.5
a(L-Pt-NH3[N4])	88.8	92.7	89.2	84.6	93.3	96.4
a(L-Pt-NH3[N6])	87.9	83.9	85.0	94.2	84.4	81.1

EDA=ethylenediamine, L=Cl/H_2O/O-R(Sugar)

Table S 22. Structural parameters for the transition structure of the aquation process studied in this manuscript. All values are reported in terms of Angstroms (Å).

	TS1
d(Pt-Cl)	2.69
d(Pt-H_2O)	2.35
d(Pt-NH_3[N4])	2.03
d(Pt-NH_3[N6])	2.04
d(Pt-EDA)	2.04
a(Cl-Pt-H_2O)	68.7
a(Cl-Pt-EDA)	141.7
a(H_2O-Pt-EDA)	149.1
a(NH_3-Pt-NH_3)	174.5

Table S 23. Structural parameters for the transition structure of the water-monosaccharide substitution processes studied in this manuscript. All values are reported in terms of Angstroms (Å).

	IDOA(2S) Model		***GlcNS(6S) Model***	
	**RCOO**	**RSO4**	**NSO3**	**RSO4**
	**TS2**	**TS2'**	**TS3**	**TS3'**
d(Pt-H_2O)	2.42	2.50	2.51	2.43
d(Pt-DIS[O])	2.37	2.39	2.33	2.37
d(Pt-NH_3[N4])	2.03	2.04	2.04	2.03
d(Pt-NH_3[N6])	2.03	2.03	2.04	2.03
d(Pt-EDA)	2.01	2.01	2.01	2.01
a(H_2O-Pt-DIS[O])	62.6	67.3	67.0	66.7
a(H_2O-Pt-EDA)	151.5	144.5	144.1	144.8
a(DIS[O]-Pt-EDA)	145.7	147.9	148.8	147.9
a(NH_3-Pt-NH_3)	171.8	178.3	179.0	176.9

Table S 24. Structural parameters for the transition structure of the direct substitution processes studied in this manuscript. All values are reported in terms of Angstroms (Å).

	IDOA(2S) Model		***GlcNS(6S) Model***	
	**RCOO**	**RSO4**	**NSO3**	**RSO4**
	**TSa**	**TSa'**	**TSb**	**TSb'**
d(Pt-Cl)	2.63	2.72	2.70	2.70
d(Pt-DIS[O])	2.31	2.34	2.33	2.32
d(Pt-NH_3[N4])	2.03	2.03	2.04	2.04
d(Pt-NH_3[N6])	2.03	2.03	2.03	2.03
d(Pt-EDA)	2.04	2.02	2.02	2.03
a(Cl-Pt-DIS[O])	79.1	75.6	76.7	78.1
a(Cl-Pt-EDA)	138.2	135.4	131.2	134.5
a(DIS[O]-Pt-EDA)	142.5	148.9	151.4	146.2
a(NH_3-Pt-NH_3)	171.7	174.5	177.0	175.2

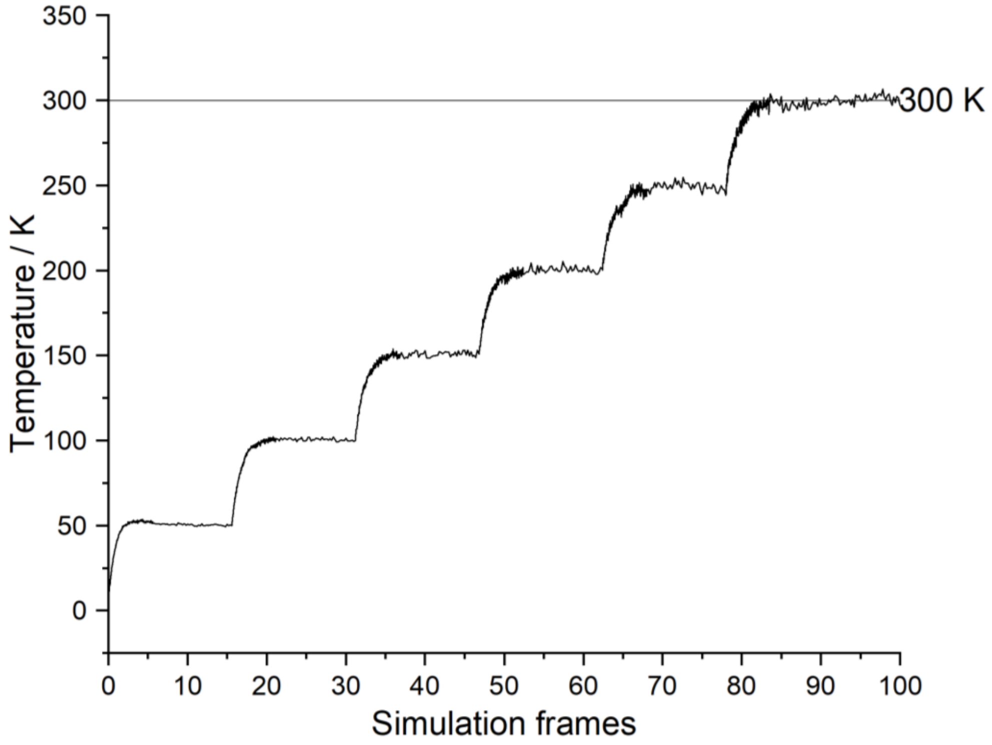

Figure S 9 – Preproduction (Heating and Equilibria step) of the molecular dynamic's simulations. Thermal protocol is shown to increase the temperature in 50K intervals from 0K to 300K.

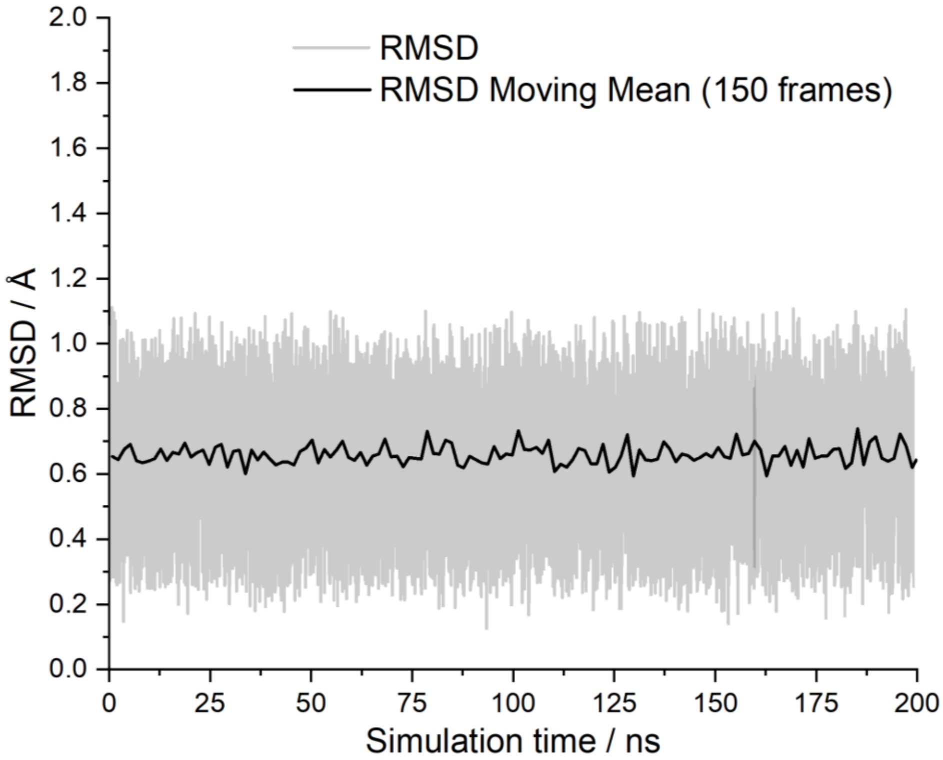

Figure S 10 – Root Mean Square Deviation (RMSD) of Cl-PtN3 along the simulated trajectory.

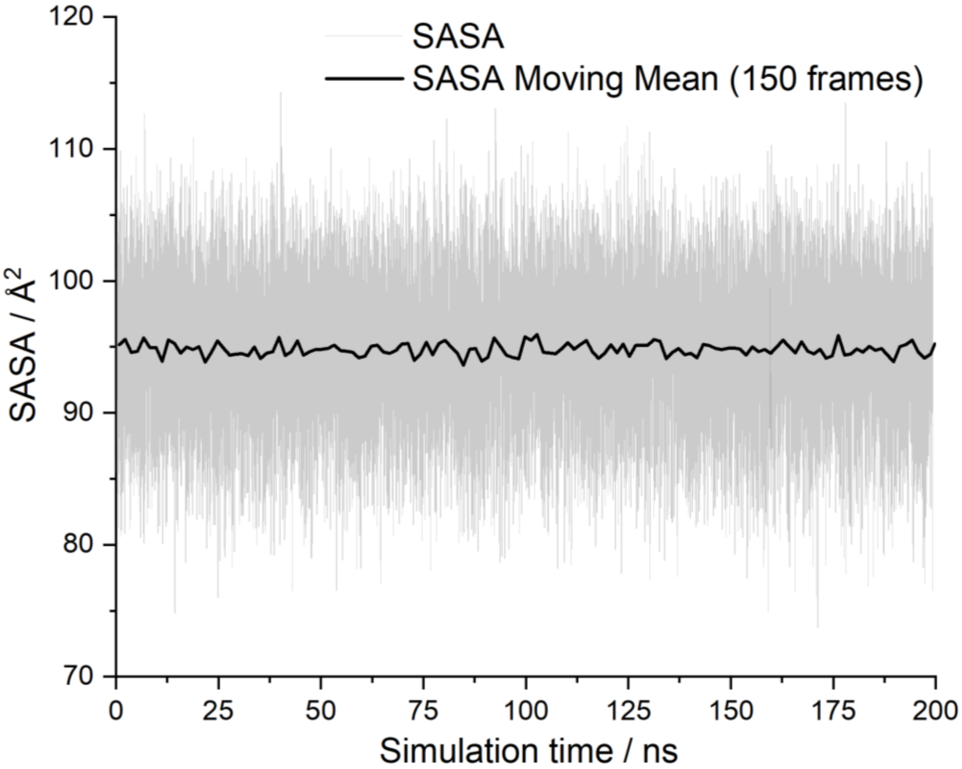

Figure S 11 – Solvent Accessible Surface Area (SASA) of Cl-PtN3 along the simulated trajectory.

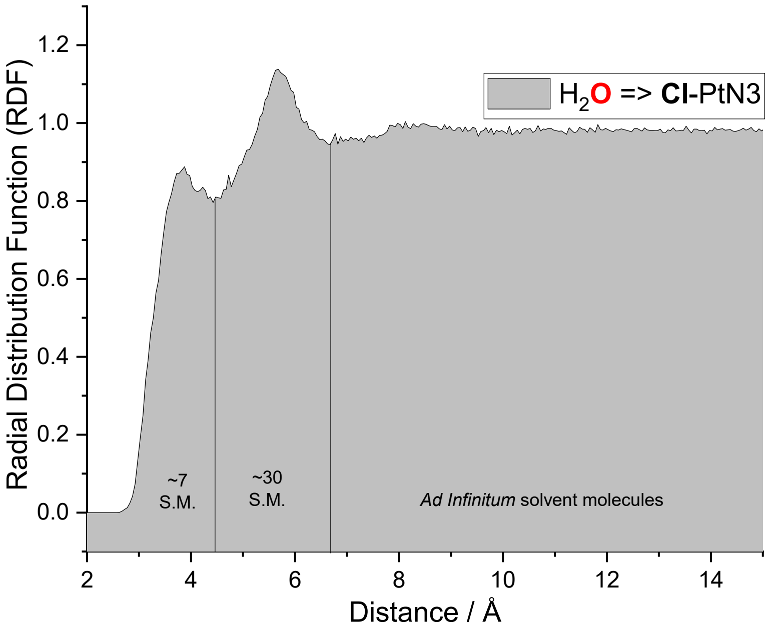

Figure S 12. Water radial distribution function (RDF) around Cl-PtN3 species. Number of solvent molecules are shown in the graph for each solvation layer observed in MD simulations.

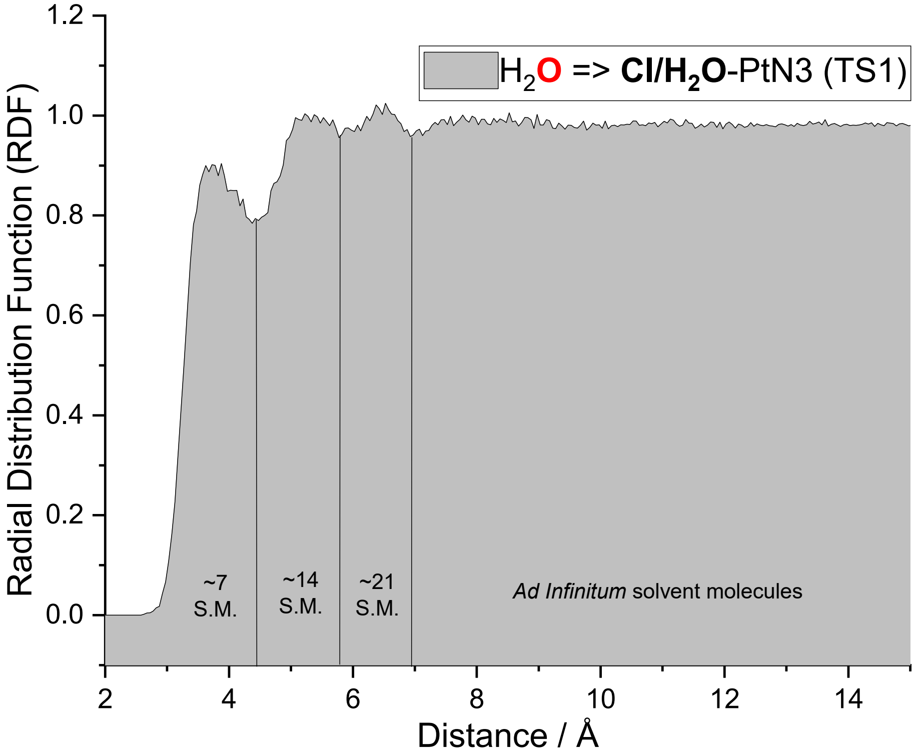

Figure S 13. Water radial distribution function (RDF) around Cl/H_2O-PtN3 species. Number of solvent molecules are shown in the graph for each solvation layer observed in MD simulations.

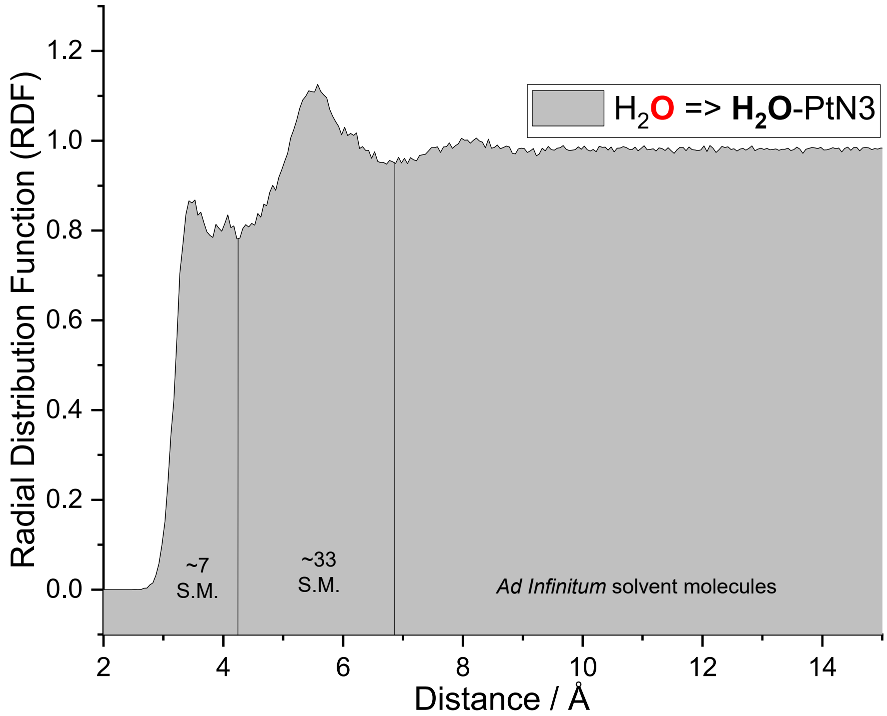

Figure S 14. Water radial distribution function (RDF) around H_2O-PtN3 species. Number of solvent molecules are shown in the graph for each solvation layer observed in MD simulations.

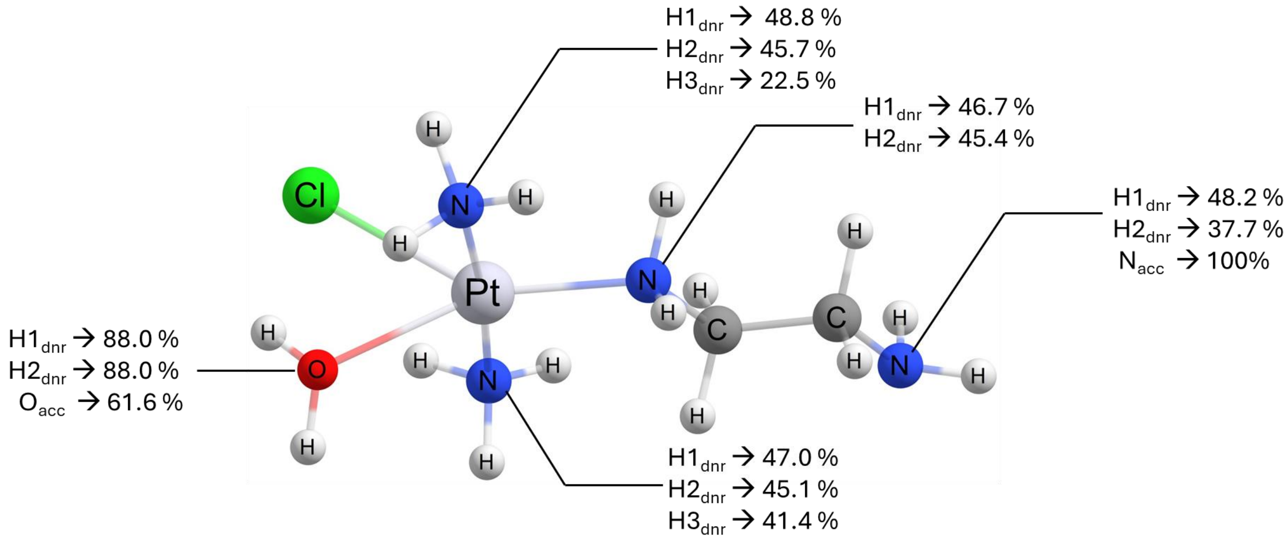

Figure S 15 – Percentage of simulation frames regarding the aquation transition state with corresponding atoms committed in hydrogen bonds with the solvent. Captions indicate hydrogen bond donors (dnr) and acceptors (acc).

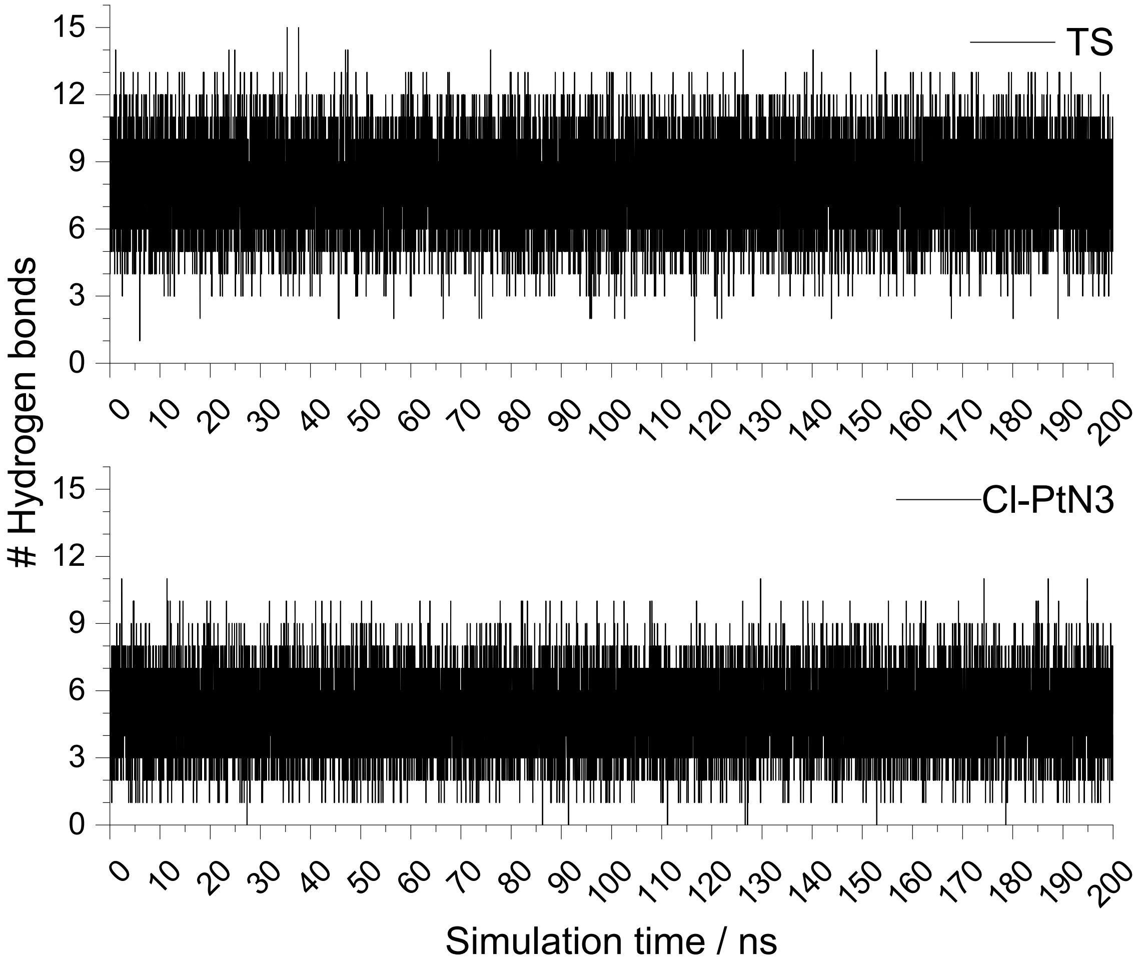

Figure S 16 – Total number of hydrogen bonds along the simulation. Upper graph indicates the aquation transition state trajectory, and the bottom one is regarding the starting material (Cl-PtN3).

Table S 25 - Gibbs free energy of reaction and activation processes for the IdoA(2S) monosaccharide model in relation to the starting material (Cl-PtN3) in different solvation protocol. Reaction via aquation.

Process	ΔG_{sol} (PCM continuum)	ΔG_{sol} (3 WAT + PCM continuum)	ΔG_{sol} (6 WAT + PCM continuum)
Cl-PtN3 → Cl-PtN3	0.00	0.00	0.00
Cl-PtN3 + H_2O → MS1	4.46	2.59	4.73
**Cl-PtN3 + H_2O → TS1**	**29.25**	**25.16**	**23.71**
Cl-PtN3 + H_2O → MS2	11.22	6.08	2.15
Cl-PtN3 + H_2O → H_2O-PtN3 + Cl^-	20.90	10.50	13.42
Cl-PtN3 + H_2O + IDOA(2S) → MS3 + Cl^-	10.53	7.89	1.31
**Cl-PtN3 + H_2O + IDOA(2S) → TS2 + Cl^-**	**28.40**	**28.60**	**21.47**
Cl-PtN3 + H_2O + IDOA(2S) → MS4 + Cl^-	4.38	2.80	5.07
Cl-PtN3 + IDOA(2S) → IDOA(2S)-CO-PtN3 + Cl^-	0.85	-0.07	3.73
Cl-PtN3 + H_2O + IDOA(2S) → MS3' + Cl^-	5.52	2.95	3.22
**Cl-PtN3 + H_2O + IDOA(2S) → TS2' + Cl^-**	**26.50**	**25.14**	**27.48**
Cl-PtN3 + H_2O + IDOA(2S) → MS4' + Cl^-	12.10	10.57	15.72
Cl-PtN3 + IDOA(2S)→ IDOA(2S)-SO-PtN3 + Cl^-	6.14	8.10	11.62

ΔG was calculated with In-solution Gibbs free energy according to equation 2 in the methodology section of the article. Values given in kcal mol $^{-1}$.

Table S 26 - Gibbs free energy of reaction and activation processes for the GlcNS(6S) monosaccharide model in relation to the starting material (Cl-PtN3) in different solvation protocol. Reaction via aquation

Species	ΔG_{sol} (PCM continuum)	ΔG_{sol} (3 WAT + PCM continuum)	ΔG_{sol} (6 WAT + PCM continuum)
Cl-PtN3 → Cl-PtN3	0.00	0.00	0.00
Cl-PtN3 + H_2O → MS1	4.46	2.59	4.73
**Cl-PtN3 + H_2O → TS1**	**29.25**	**25.16**	**23.71**
Cl-PtN3 + H_2O → MS2	11.22	6.08	2.15
Cl-PtN3 → H_2O-PtN3	20.90	10.50	13.42
Cl-PtN3 → MS5	0.01	8.63	5.46
**Cl-PtN3 → TS3**	**29.21**	**27.92**	**27.02**
Cl-PtN3 → MS6	11.63	13.25	13.36
Cl-PtN3 → GlcNS(6S)-NS-PtN3	6.60	8.25	10.48
Cl-PtN3 → MS5'	9.93	8.07	5.59
**Cl-PtN3 → TS3'**	**30.09**	**26.88**	**26.73**
Cl-PtN3 → MS6'	12.15	13.80	13.23
Cl-PtN3 → GlcNS(6S)-OS-PtN3	4.76	8.94	10.69

ΔG was calculated with In-solution Gibbs free energy according to equation 2 in the methodology section of the article. Values given in kcal mol $^{-1}$.

Table S 27 - Gibbs free energy of reaction and activation processes for the IdoA(2S) monosaccharide model in relation to the starting material (Cl-PtN3) in different solvation protocol. *Direct substitution reaction.*

**Process**	ΔG_{sol} (PCM continuum)	ΔG_{sol} (3 WAT + PCM continuum)	ΔG_{sol} (6 WAT + PCM continuum)
Cl-PtN3 → Cl-PtN3	0.00	0.00	0.00
Cl-PtN3 → MSa	-4.92	0.69	-3.85
**Cl-PtN3 → TSa**	**23.40**	**20.25**	**16.29**
Cl-PtN3 → MSb	0.80	-2.00	-6.01
Cl-PtN3 → IDOA(2S)-CO-PtN3	0.85	-0.07	3.73
Cl-PtN3 → MSa'	-8.92	-4.91	-7.95
**Cl-PtN3 → TSa'**	**19.32**	**17.40**	**16.98**
Cl-PtN3 → MSb'	6.51	0.97	5.45
Cl-PtN3 → IDOA(2S)-SO-PtN3	6.14	6.21	11.62

ΔG was calculated with In-solution Gibbs free energy according to equation 2 in the methodology section of the article. Values given in kcal mol $^{-1}$.

Table S 28 - Gibbs free energy of reaction and activation processes for the GlcNS(6S) monosaccharide model in relation to the starting material (Cl-PtN3) in different solvation protocol. *Direct substitution reaction.*

**Process**	ΔG_{sol} (PCM continuum)	ΔG_{sol} (3 WAT + PCM continuum)	ΔG_{sol} (6 WAT + PCM continuum)
Cl-PtN3 + dis1 → Cl-PtN3 + dis1	0.00	0.00	0.00
Cl-PtN3 + dis1 → MSc	-2.64	-2.13	1.82
**Cl-PtN3 + dis1 → TSb**	**23.58**	**20.50**	**25.42**
Cl-PtN3 + dis1 → MSd	9.69	7.87	9.37
Cl-PtN3 + dis1 → GlcNS(6S)-NS-PtN3 + Cl^-	6.60	8.25	10.48
Cl-PtN3 + dis1 → MSc'	6.11	-0.35	-6.28
**Cl-PtN3 + dis1 → TSb'**	**25.37**	**24.03**	**24.04**
Cl-PtN3 + dis1 → MSd'	5.90	2.41	8.27
Cl-PtN3 + dis1 → GlcNS(6S)-OS-PtN3 + Cl^-	4.76	8.94	10.69

ΔG was calculated with In-solution Gibbs free energy according to equation 2 in the methodology section of the article. Values given in kcal mol $^{-1}$.

Table S 29 – Summary activation free energies for the reactions studied comprising both IdoA(2S) and GlnNS(6S) monosaccharide models.

Process	ΔG_{sol} (PCM continuum)	ΔG_{sol} (3 WAT + PCM continuum)	ΔG_{sol} (6 WAT + PCM continuum)	ΔG_{sol} Experimental
**Cl-PtN3 + H_2O → TS1**	**29.25**	**25.16**	**23.71**	**~ 24.1 kcal mol^{-1}**
**Cl-PtN3 + H_2O + IDOA(2S) → TS2 + Cl^-**	**28.40**	**28.60**	**21.47**	**19.1 ~ 22.7 kcal mol^{-1} For similar carbohydrate fragments such as GlcNS(6S), GlcNS, GlcNAc(6S), GlcNS(6S)-GlcA and GlcNS(6S)-IdoA(2S) ***
**Cl-PtN3 + H_2O + IDOA(2S) → TS2’ + Cl^-**	**26.50**	**25.14**	**27.48**	
**Cl-PtN3 + H_2O + GlcNS(6S) → TS3 + Cl^-**	**29.21**	**27.92**	**27.02**	
**Cl-PtN3 + H_2O + GlcNS(6S) → TS3’ + Cl^-**	**30.09**	**26.88**	**26.73**	
**Cl-PtN3 + IDOA(2S) → TSa + Cl^-**	**23.40**	**20.25**	**16.29**	-
**Cl-PtN3 + IDOA(2S) → TSa' + Cl^-**	**19.32**	**17.40**	**16.98**	
**Cl-PtN3 + GlcNS(6S) → TSb + Cl^-**	**23.58**	**20.50**	**25.42**	
**Cl-PtN3 + GlcNS(6S) → TSb' + Cl^-**	**25.37**	**24.03**	**24.04**	

Gorle, A. K., Malde, A. K., Chang, C.-W., Rajaratnam, P., von Itzstein, M., Berners-Price, S. J., & Farrell, N. P. (2023). Probing Disaccharide Binding to Triplatin as Models for Tumor Cell Heparan Sulfate (GAG) Interactions. *Inorganic Chemistry*, *62*(33), 13212–13220. https://doi.org/10.1021/acs.inorgchem.3c01391

Gorle, A. K., Rajaratnam, P., Chang, C. W., von Itzstein, M., Berners-Price, S. J., & Farrell, N. P. (2019). Glycans as Ligands in Bioinorganic Chemistry. Probing the Interaction of a Trinuclear Platinum Anticancer Complex with Defined Monosaccharide Fragments of Heparan Sulfate. *Inorganic Chemistry*, *58*(11), 7146–7155. https://doi.org/10.1021/acs.inorgchem.8b03035

Ruhayel, R. A., Corry, B., Braun, C., Thomas, D. S., Berners-Price, S. J., & Farrell, N. P. (2010). Determination of the kinetic profile of a dinuclear platinum anticancer complex in the presence of sulfate: Introducing a new tool for the expedited analysis of 2D [1H,15N] HSQC NMR spectra. *Inorganic Chemistry*, *49*(23), 10815–10819. https://doi.org/10.1021/ic100576k

Reaction diagrams

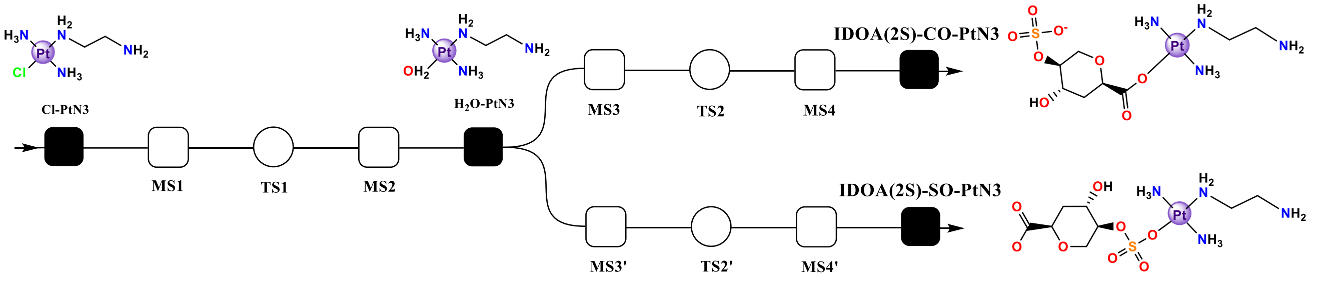

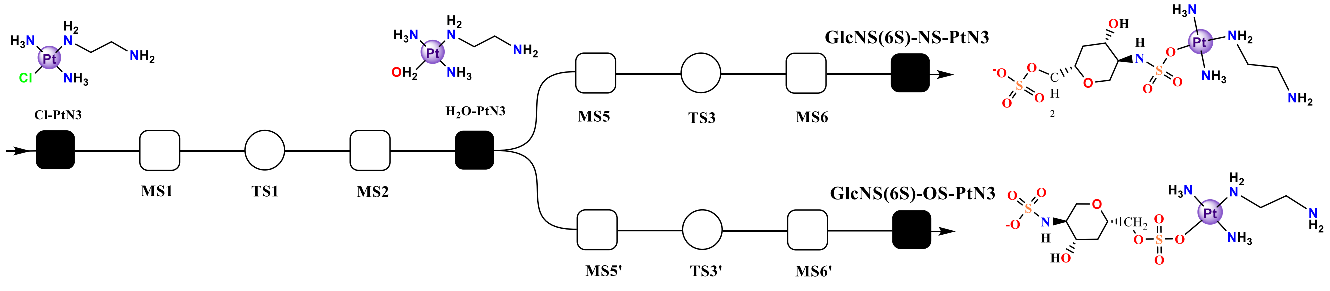

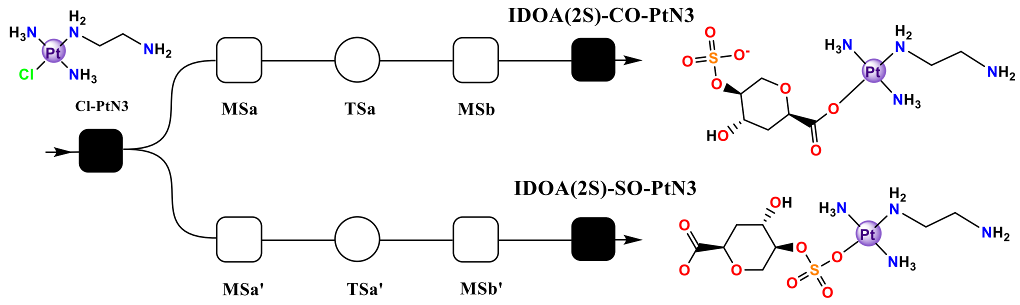

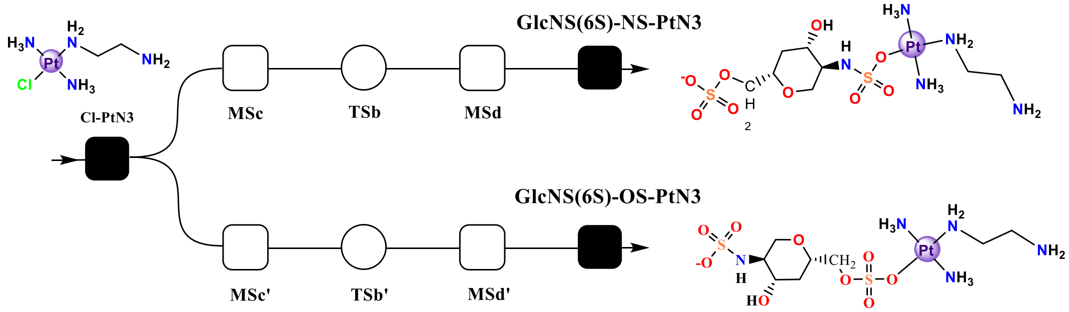

REFERENCES

(1) Kohn, W.; Sham, L. J. Self-Consistent Equations Including Exchange and Correlation Effects. *Physical Review* **1965**, *140* (4A), A1133–A1138. https://doi.org/10.1103/PhysRev.140.A1133.

(2) Parr, R. G.; Weitao, Y. *Density-Functional Theory of Atoms and Molecules*; Oxford University Press, 1995. https://doi.org/10.1093/oso/9780195092769.001.0001.

(3) Becke, A. D. A New Mixing of Hartree-Fock and Local Density-Functional Theories. *J. Chem. Phys.* **1993**, *98* (2), 1372–1377. https://doi.org/10.1063/1.464304.

(4) Hehre, W. J.; Ditchfield, K.; Pople, J. A. Self-Consistent Molecular Orbital Methods. XII. Further Extensions of Gaussian-Type Basis Sets for Use in Molecular Orbital Studies of Organic Molecules. *J. Chem. Phys.* **1972**, *56* (5), 2257–2261. https://doi.org/10.1063/1.1677527.

(5) Andrae, D.; Häußermann, U.; Dolg, M.; Stoll, H.; Preuß, H. Energy-Adjustedab Initio Pseudopotentials for the Second and Third Row Transition Elements. *Theor. Chim. Acta* **1990**, *77* (2), 123–141. https://doi.org/10.1007/BF01114537.

(6) Scalmani, G.; Frisch, M. J. Continuous Surface Charge Polarizable Continuum Models of Solvation. I. General Formalism. *Journal of Chemical Physics* **2010**, *132* (11). https://doi.org/10.1063/1.3359469.

(7) Frisch, M. J.; Trucks, G. W.; Schlegel, H. B.; Scuseria, G. E.; Robb, M. A.; Cheeseman, J. R.; Scalmani, G.; Barone, V.; Mennucci, B.; Petersson, G. A.; Nakatsuji, H.; Caricato, M.; Li, X.; Hratchian, H. P.; Izmaylov, A. F.; Bloino, J.; Zheng, G.; Sonnenberg, J. L.; Hada, M.; Ehara, M.; Toyota, K.; Fukuda, R.; Hasegawa, J.; Ishida, M.; Nakajima, T.; Honda, Y.; Kitao, O.; Nakai, H.; Vreven, T.; Montgomery, J. A.; Peralta, J. E.; Ogliaro, F.; Bearpark, M.; Heyd, J. J.; Brothers, E.; Kudin, K. N.; Staroverov, V. N.; Kobayashi, R.; Normand, J.; Raghavachari, K.; Rendell, A.; Burant, J. C.; Iyengar, S. S.; Tomasi, J.; Cossi, M.; Rega, N.; Millam, J. M.; Klene, M.; Knox, J. E.; Cross, J. B.; Bakken, V.; Adamo, C.; Jaramillo, J.; Gomperts, R.; Stratmann, R. E.; Yazyev, O.; Austin, A. J.; Cammi, R.; Pomelli, C.; Ochterski, J. W.; Martin, R. L.; Morokuma, K.; Zakrzewski, V. G.; Voth, G. A.; Salvador, P.; Dannenberg, J. J.; Dapprich, S.; Daniels, A. D.; Farkas, Ö.; Foresman, J. B.; Ortiz, J. V; Cioslowski, J.; Fox, D. J. Gaussian 09 Revision A.2. 2009.

(8) Neese, F.; Wennmohs, F.; Becker, U.; Riplinger, C. The ORCA Quantum Chemistry Program Package. *Journal of Chemical Physics* **2020**, *152* (22). https://doi.org/10.1063/5.0004608.

(9) Neese, F. The ORCA Program System. *Wiley Interdiscip. Rev. Comput. Mol. Sci.* **2012**, *2* (1), 73–78. https://doi.org/10.1002/wcms.81.

(10) Neese, F. Software Update: The <scp>ORCA</Scp> Program System—Version 5.0. *WIREs Computational Molecular Science* **2022**, *12* (5). https://doi.org/10.1002/wcms.1606.

(11) Barone, V.; Cossi, M. Quantum Calculation of Molecular Energies and Energy Gradients in Solution by a Conductor Solvent Model. *J. Phys. Chem. A* **1998**, *102* (11), 1995–2001. https://doi.org/10.1021/jp9716997.

(12) Takano, Y.; Houk, K. N. Benchmarking the Conductor-like Polarizable Continuum Model (CPCM) for Aqueous Solvation Free Energies of Neutral and Ionic Organic Molecules. *J. Chem. Theory Comput.* **2005**, *1* (1), 70–77. https://doi.org/10.1021/ct049977a.

(13) Chai, J. Da; Head-Gordon, M. Long-Range Corrected Hybrid Density Functionals with Damped Atom-Atom Dispersion Corrections. *Physical Chemistry Chemical Physics* **2008**, *10* (44), 6615–6620. https://doi.org/10.1039/b810189b.

(14) Zhao, Y.; Truhlar, D. G. The M06 Suite of Density Functionals for Main Group Thermochemistry, Thermochemical Kinetics, Noncovalent Interactions, Excited States, and Transition Elements: Two New Functionals and Systematic Testing of Four M06-Class Functionals and 12 Other Functionals. *Theor. Chem. Acc.* **2008**, *120* (1–3), 215–241. https://doi.org/10.1007/s00214-007-0310-x.

(15) Ernzerhof, M.; Scuseria, G. E. Assessment of the Perdew-Burke-Ernzerhof Exchange-Correlation Functional. *Journal of Chemical Physics* **1999**, *110* (11), 5029–5036. https://doi.org/10.1063/1.478401.

(16) Adamo, C.; Barone, V. Toward Reliable Density Functional Methods without Adjustable Parameters: The PBE0 Model. *Journal of Chemical Physics* **1999**, *110* (13), 6158–6170. https://doi.org/10.1063/1.478522.

(17) Stephens, P. J.; Devlin, F. J.; Chabalowski, C. F.; Frisch, M. J. Ab Initio Calculation of Vibrational Absorption and Circular Dichroism Spectra Using Density Functional Force Fields. *J. Phys. Chem.* **1994**, *98* (45), 11623–11627. https://doi.org/10.1021/j100096a001.

(18) Yanai, T.; Tew, D. P.; Handy, N. C. A New Hybrid Exchange-Correlation Functional Using the Coulomb-Attenuating Method (CAM-B3LYP). *Chem. Phys. Lett.* **2004**, *393* (1–3), 51–57. https://doi.org/10.1016/j.cplett.2004.06.011.

(19) Grimme, S. Semiempirical Hybrid Density Functional with Perturbative Second-Order Correlation. *J. Chem. Phys.* **2006**, *124* (3). https://doi.org/10.1063/1.2148954.

(20) Peverati, R.; Truhlar, D. G. Communication: A Global Hybrid Generalized Gradient Approximation to the Exchange-Correlation Functional That Satisfies the Second-Order Density-Gradient Constraint and Has Broad Applicability in Chemistry. *J. Chem. Phys.* **2011**, *135* (19). https://doi.org/10.1063/1.3663871.

(21) Furness, J. W.; Kaplan, A. D.; Ning, J.; Perdew, J. P.; Sun, J. Accurate and Numerically Efficient R2SCAN Meta-Generalized Gradient Approximation. *Journal of Physical Chemistry Letters* **2020**, *11* (19), 8208–8215. https://doi.org/10.1021/acs.jpclett.0c02405.

(22) Mardirossian, N.; Head-Gordon, M. *ω* B97M-V: A Combinatorially Optimized, Range-Separated Hybrid, Meta-GGA Density Functional with VV10 Nonlocal Correlation. *J. Chem. Phys.* **2016**, *144* (21). https://doi.org/10.1063/1.4952647.

(23) Riplinger, C.; Neese, F. An Efficient and near Linear Scaling Pair Natural Orbital Based Local Coupled Cluster Method. *J. Chem. Phys.* **2013**, *138* (3). https://doi.org/10.1063/1.4773581.

(24) Riplinger, C.; Sandhoefer, B.; Hansen, A.; Neese, F. Natural Triple Excitations in Local Coupled Cluster Calculations with Pair Natural Orbitals. *Journal of Chemical Physics* **2013**, *139* (13). https://doi.org/10.1063/1.4821834.

(25) Sandler, I.; Chen, J.; Taylor, M.; Sharma, S.; Ho, J. Accuracy of DLPNO-CCSD(T): Effect of Basis Set and System Size. *Journal of Physical Chemistry A* **2021**, *125* (7), 1553–1563. https://doi.org/10.1021/acs.jpca.0c11270.

(26) Weigend, F.; Ahlrichs, R. Balanced Basis Sets of Split Valence, Triple Zeta Valence and Quadruple Zeta Valence Quality for H to Rn: Design and Assessment of Accuracy. *Physical Chemistry Chemical Physics* **2005**, *7* (18), 3297–3305. https://doi.org/10.1039/b508541a.

(27) Hellweg, A.; Hättig, C.; Höfener, S.; Klopper, W. Optimized Accurate Auxiliary Basis Sets for RI-MP2 and RI-CC2 Calculations for the Atoms Rb to Rn. *Theor. Chem. Acc.* **2007**, *117* (4), 587–597. https://doi.org/10.1007/s00214-007-0250-5.

(28) Mardirossian, N.; Head-Gordon, M. Thirty Years of Density Functional Theory in Computational Chemistry: An Overview and Extensive Assessment of 200 Density Functionals. *Mol. Phys.* **2017**, *115* (19), 2315–2372. https://doi.org/10.1080/00268976.2017.1333644.

(29) Ianni, J. C. Kintecus. *Windows Version 6.01* **2017**, No. www.kintecus.com.

Optimized Structures in the Format of xyz Coordinates.

Cl-PtN3

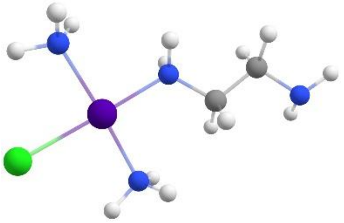

N	-1.341680	0.742192	0.167552
H	-1.459458	1.145145	1.094441
H	-1.443246	1.517429	-0.482859
N	1.243610	1.992077	-0.055000
Pt	0.586496	0.064692	0.030310
N	0.040821	-1.896777	0.102461
H	2.259405	1.986965	-0.017502
H	0.920096	2.573927	0.711485
H	-0.370961	-2.160552	0.992228
H	0.877238	-2.462945	-0.014992
H	0.985153	2.456414	-0.920313
H	-0.607128	-2.168844	-0.629717
C	-2.427693	-0.203487	-0.075287
C	-3.779020	0.457490	-0.016454
H	-2.286393	-0.656394	-1.056638
H	-2.370503	-0.990431	0.678166
H	-3.850114	1.197787	-0.817043
H	-3.873110	1.002280	0.933133
N	-4.796810	-0.536374	-0.220402
H	-4.904034	-1.125149	0.594021
H	-5.693228	-0.111355	-0.404892
Cl	2.781663	-0.640121	-0.143266

H_2O-PtN3

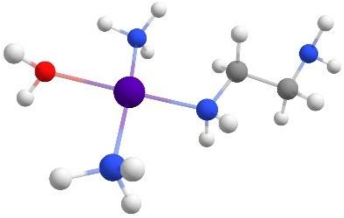

N	1.144210	-0.724667	-0.212213
H	1.247012	-1.531445	0.399390
H	1.230802	-1.087528	-1.160135
N	-1.411043	-1.933291	0.104891
Pt	-0.728342	-0.008589	-0.015431
N	-0.163854	1.945066	-0.122699
H	-2.426425	-1.967334	0.116153
H	-1.105028	-2.399375	0.954302
H	0.478061	2.231113	0.609830
H	-0.998914	2.516732	-0.019164
H	-1.120054	-2.510697	-0.678818
H	0.252627	2.195056	-1.014439
C	2.265808	0.180213	0.045184
C	3.589088	-0.522520	-0.103347
H	2.220260	1.010314	-0.657862
H	2.160045	0.572449	1.057583
H	3.697611	-0.862640	-1.135839
H	3.597278	-1.418888	0.532098
O	-2.623475	0.778205	0.167230
H	-3.236886	0.622033	-0.558579
H	-3.084445	0.635091	1.000457
N	4.644774	0.406576	0.189191
H	4.720579	0.577832	1.182291
H	5.537997	0.059632	-0.126698

IDOA(2S)-Model

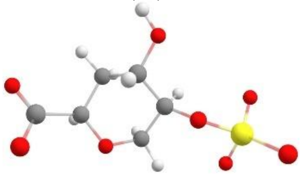

C	-0.20880	-0.88870	-1.07930
C	0.41790	0.33160	-0.44030
C	-0.65240	1.18150	0.18450
C	-1.89290	1.09810	-0.66390
C	-2.41990	-0.33290	-0.65170
C	-3.52770	-0.50720	0.38180
O	1.32280	-0.02240	0.59020
O	-0.15690	2.48240	0.29400
O	-1.38320	-1.24450	-0.42020
O	-4.49910	0.25440	0.21580
O	-3.39570	-1.36580	1.26360
S	2.85270	-0.24570	0.15610
O	3.47210	-0.61220	1.41480
O	2.82540	-1.30980	-0.83790
O	3.29600	1.02970	-0.38690
H	0.48730	-1.72290	-1.01690
H	0.94810	0.93460	-1.18410
H	-0.85890	0.76820	1.17900
H	-1.64220	1.42600	-1.67700
H	-2.87720	-0.55270	-1.62600
H	-0.76790	3.01610	0.80350
H	-2.67000	1.76500	-0.29030
H	-0.41030	-0.69170	-2.14010

GlcNS(6S)-Model

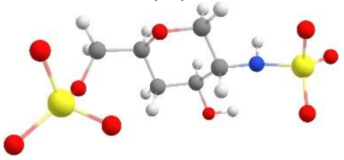

C	0.76090	0.21350	1.45820
C	1.34690	0.51750	0.09970
C	0.76100	1.79840	-0.43440
C	-0.73510	1.70460	-0.42470
C	-1.21300	1.34210	0.95940
C	-2.70730	1.21510	1.04220
N	2.78350	0.63990	0.08930
O	1.22460	2.03860	-1.72520
O	-0.62860	0.14590	1.39470
O	-3.31510	0.70110	-0.12030
S	3.65500	-0.76860	-0.06270
O	3.19640	-1.69660	0.97200
O	3.35090	-1.24460	-1.40720
O	5.03120	-0.32570	0.12760
S	-3.43780	-0.88660	-0.34710
O	-4.60020	-0.97080	-1.21500
O	-2.20370	-1.30000	-0.99400
O	-3.63470	-1.45520	0.97760
H	1.13240	-0.74390	1.81960
H	1.08040	-0.28420	-0.59650
H	1.07580	2.61720	0.23310
H	-1.05680	0.92300	-1.11810
H	-0.92380	2.13660	1.66630
H	-2.97640	0.59880	1.90180
H	-3.14260	2.20640	1.16260
H	3.15070	1.17990	0.86550
H	2.16650	1.83310	-1.71530
H	1.06860	0.99730	2.16950
H	-1.18100	2.65120	-0.74030

IDOA(2S)-CO-PtN3

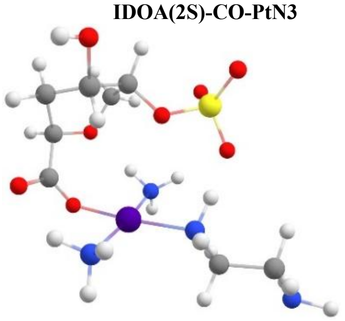

N	2.139888	-0.104600	0.715144
H	1.820890	0.871183	0.644118
H	2.241660	-0.303177	1.706122
N	0.555103	-2.550946	1.433638
Pt	0.670361	-1.220667	-0.113394
N	0.592864	0.028321	-1.706804
H	0.668288	-2.152376	2.359084
H	1.230497	-3.302555	1.344387
H	1.115003	-0.310529	-2.507300
H	0.898615	0.976969	-1.486068
H	-0.386718	-2.956471	1.382872
H	-0.403495	0.036498	-1.944586
C	3.447572	-0.209069	0.072929
C	4.432395	0.765779	0.660171
H	3.814246	-1.230174	0.173004
H	3.317461	-0.010114	-0.992860
H	4.592806	0.522821	1.713714
H	3.994720	1.773538	0.625491
N	5.687309	0.651342	-0.033143
H	5.622043	1.029302	-0.968483
H	6.417308	1.159194	0.444107
C	-2.646638	1.241672	-1.262097
C	-2.264336	1.584613	0.150239
C	-2.739171	0.514810	1.116105
C	-3.661267	-0.453087	0.421640
C	-2.953644	-1.058696	-0.769760
C	-1.940780	-2.101034	-0.326587
O	-0.851214	1.622017	0.262809
O	-3.348097	1.153553	2.196469
O	-2.303551	-0.085356	-1.552694
O	-2.194803	-2.811622	0.633184
O	-0.868587	-2.190109	-1.023579
S	-0.061321	2.983696	-0.013281
O	1.290430	2.448572	-0.200379
O	-0.634933	3.551942	-1.218595
O	-0.215458	3.805836	1.168027
H	-2.092986	1.874122	-1.953917
H	-2.693992	2.546526	0.443315
H	-1.843646	-0.026910	1.446549
H	-4.575354	0.059244	0.115952
H	-3.682420	-1.586502	-1.396685
H	-3.567816	0.509026	2.871267
H	-3.940370	-1.263323	1.094604
H	-3.715018	1.419168	-1.417112

IDOA(2S)-SO-PtN3

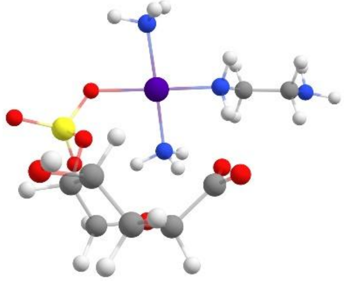

Pt	-1.005435	-0.970897	0.213573
N	-2.349355	0.498059	0.487456
N	-1.420098	-1.847373	2.010117
C	-3.573963	0.442584	-0.302599
H	-1.815660	1.365831	0.268995
H	-2.600836	0.560210	1.470062
N	-0.555605	-0.119030	-1.580048
H	-1.238097	-1.244851	2.806441
H	-0.817745	-2.659817	2.112638
H	-2.378723	-2.171674	2.085680
C	-4.439724	1.648500	-0.051093
H	-4.110736	-0.475065	-0.056055
H	-3.314790	0.396387	-1.360235
H	-1.343592	-0.119462	-2.218109
H	0.209665	-0.607347	-2.044895
H	-0.270307	0.860002	-1.402053
N	-5.646577	1.543788	-0.825184
H	-3.897386	2.544043	-0.364370
H	-4.615020	1.744632	1.030106
H	-6.260420	0.833873	-0.449735
H	-6.158671	2.413462	-0.824055
C	0.361049	2.987760	0.106185
C	1.718740	2.844647	-0.577898
O	-0.595884	2.363244	-0.408514
O	0.306760	3.740171	1.080565
C	2.856572	2.734796	0.412933
O	1.677905	1.753523	-1.463533
H	1.852638	3.764121	-1.161079
C	2.924441	1.346417	0.991082
H	2.679819	3.465274	1.202175
H	3.811795	2.975580	-0.059006
C	2.806037	0.936293	-1.459997
C	2.999877	0.301568	-0.110756
O	4.033056	1.167952	1.819626
H	1.998582	1.148129	1.544433
H	2.653260	0.178795	-2.224735
H	3.713410	1.494941	-1.714194
O	1.944459	-0.608415	0.197228
H	3.959393	-0.219413	-0.055906
H	3.983223	1.775795	2.559284
S	1.800278	-2.029715	-0.476205
O	1.758720	-1.834534	-1.911641
O	0.463558	-2.418073	0.066875
O	2.844296	-2.889063	0.009602

GlcNS(6S)-SO-PtN3

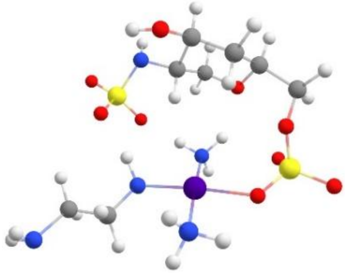

N	-1.855021	-1.327066	-0.276789
H	-2.162369	-0.391345	0.037249
H	-2.075675	-1.354564	-1.270046
N	0.295868	-0.175858	-1.647812
Pt	0.145348	-1.486322	-0.094059
N	0.080774	-2.814306	1.456369
H	-0.551272	0.391937	-1.770680
H	0.469890	-0.679130	-2.511834
H	-0.415569	-3.673660	1.245325
H	-0.323267	-2.427015	2.302920
H	1.092366	0.452211	-1.507706
H	1.041842	-3.066825	1.673665
C	-2.688757	-2.310968	0.405311
C	-4.148167	-2.089359	0.105519
H	-2.396497	-3.316103	0.099595
H	-2.518860	-2.216000	1.479547
H	-4.321816	-2.263381	-0.959636
H	-4.392796	-1.037758	0.304603
N	-4.934828	-3.030979	0.857060
H	-4.959619	-2.783714	1.836932
H	-5.889745	-3.051096	0.530933
C	0.573410	2.906559	-0.981493
C	-0.331076	2.648568	0.193610
C	0.327627	3.181418	1.436158
C	1.641036	2.467126	1.591772
C	2.479491	2.520501	0.332094
C	3.627344	1.560584	0.397466
N	-1.665982	3.165157	0.045561
O	-0.464640	2.941036	2.553189
O	1.748346	2.173068	-0.815073
O	3.119624	0.275134	0.723110
S	-2.743648	2.076472	-0.582497
O	-2.097138	1.410579	-1.724186
O	-2.960212	1.108379	0.497183
O	-3.900028	2.872883	-0.935069
S	3.288494	-0.925147	-0.292062
O	4.547435	-1.565722	-0.019114
O	2.161665	-1.805508	0.133996
O	3.113499	-0.397102	-1.623258
H	0.105437	2.581936	-1.910324
H	-0.413754	1.563455	0.331067
H	0.491817	4.262497	1.312199
H	1.421649	1.422470	1.829243
H	2.890279	3.530225	0.194792
H	4.139848	1.531921	-0.563920
H	4.327100	1.843938	1.182226
H	-1.733731	4.051359	-0.442226
H	-1.371842	3.146088	2.300732
H	0.798974	3.978672	-1.065753
H	2.213379	2.888622	2.421055

GlcNS(6S)-NS-PtN3

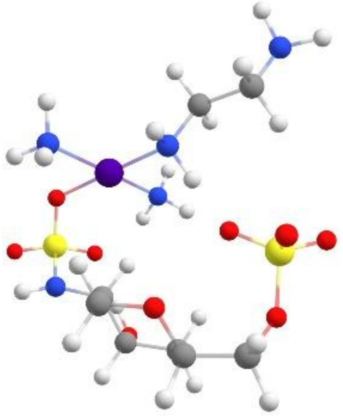

N	1.113873	1.816591	0.524852
H	1.251512	2.038106	1.506890
H	1.569072	0.906607	0.356634
N	-0.312830	0.770950	-1.706309
Pt	-0.831444	1.476532	0.138575
N	-1.433924	2.209451	1.941567
H	0.543133	0.210300	-1.665241
H	-0.162583	1.530058	-2.363268
H	-1.260083	3.205621	2.033563
H	-1.019266	1.755042	2.748744
H	-1.072258	0.194934	-2.077906
H	-2.439558	2.071845	2.006148
C	1.793416	2.820426	-0.288582
C	3.256448	2.898850	0.057443
H	1.684114	2.550431	-1.338826
H	1.305230	3.784702	-0.139966
H	3.715588	1.923458	-0.126002
H	3.360040	3.098889	1.133707
N	3.894704	3.879632	-0.777517
H	3.626559	4.817149	-0.511371
H	4.899772	3.823450	-0.705445
C	-0.657503	-1.768929	1.772202
C	-1.344276	-1.801528	0.430007
C	-1.159619	-3.159399	-0.195387
C	0.323745	-3.414134	-0.280794
C	1.026405	-3.206678	1.038652
C	2.520118	-3.263504	0.895896
N	-2.733357	-1.416053	0.531441
O	-1.725102	-3.247933	-1.466791
O	0.705605	-1.967396	1.610358
O	3.001903	-2.662914	-0.290472
S	-3.322134	-0.303341	-0.493664
O	-2.817512	1.073543	-0.135826
O	-2.758037	-0.695831	-1.785040
O	-4.752969	-0.319677	-0.358719
S	3.277716	-1.094195	-0.375624
O	4.246306	-1.007635	-1.446011
O	1.993565	-0.471871	-0.722423
O	3.747307	-0.679705	0.932368
H	-0.791937	-0.791309	2.235797
H	-0.812765	-1.075379	-0.190048
H	-1.639818	-3.903427	0.455555
H	0.739645	-2.717100	-1.012421
H	0.737603	-3.998924	1.746173
H	2.993454	-2.794331	1.759787
H	2.843040	-4.300719	0.825806
H	-3.152383	-1.380465	1.451599
H	-2.160272	-2.415315	-1.701312
H	-1.085327	-2.533524	2.438467
H	0.510635	-4.428311	-0.641678

Common Aquation Step

MS1

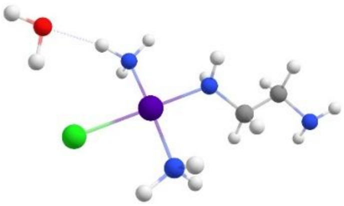

N	1.474126	0.799033	0.082969
H	1.564213	1.460703	-0.684420
H	1.463791	1.360129	0.931859
N	-1.156008	1.697470	-0.632285
Pt	-0.354563	-0.093675	-0.081014
N	0.355759	-1.923085	0.477725
H	-0.564707	2.507260	-0.476392
H	-2.026895	1.850336	-0.101321
H	0.691761	-1.938565	1.435411
H	1.100798	-2.271979	-0.116835
H	-1.392249	1.691981	-1.619388
H	-0.406761	-2.593617	0.422405
C	2.667136	-0.043773	0.091340
C	3.930305	0.773890	0.147141
H	2.630039	-0.705894	0.955294
H	2.656499	-0.655459	-0.812438
H	3.954995	1.328472	1.088265
H	3.914542	1.517463	-0.662111
N	5.063437	-0.108424	0.102883
H	5.196617	-0.488308	-0.824156
H	5.913663	0.372181	0.356479
Cl	-2.444182	-1.099389	-0.265004
O	-3.432124	1.550028	0.965201
H	-4.304436	1.916225	0.816592
H	-3.473681	0.609485	0.743410

TS1
(-182.32 cm⁻¹)

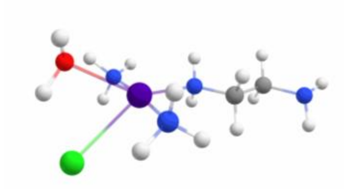

N	1.505653	0.712247	-0.225096
H	1.568510	1.052321	-1.181507
H	1.680662	1.516386	0.372584
N	-1.086962	1.991949	-0.435967
Pt	-0.429026	0.147223	0.096579
N	0.041991	-1.751151	0.659001
H	-0.411467	2.748808	-0.451112
H	-1.809732	2.245549	0.233962
H	0.148680	-1.845128	1.664422
H	0.874707	-2.140299	0.231320
H	-1.523266	1.956980	-1.352673
H	-0.738028	-2.339678	0.373019
C	2.573999	-0.258948	-0.008863
C	3.935192	0.348867	-0.225081
H	2.504503	-0.641516	1.008910
H	2.428231	-1.086322	-0.705820
H	4.093610	1.140277	0.511347
H	3.966875	0.822139	-1.216112
N	4.936006	-0.663873	-0.031843
H	4.967504	-1.304053	-0.813182
H	5.855028	-0.258153	0.062754
Cl	-2.517374	-1.032554	-1.127930
O	-2.364530	0.255996	1.418171
H	-2.323100	-0.090471	2.312344
H	-2.939065	-0.318490	0.887022

MS2

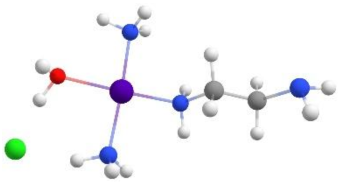

N	-1.582930	0.415829	-0.701157
H	-1.511346	1.395957	-0.963753
H	-1.853080	-0.056571	-1.561286
N	0.944093	1.645580	-0.102836
Pt	0.218694	-0.261939	-0.095276
N	-0.415543	-2.202232	-0.082956
H	0.537504	2.213042	0.635314
H	0.777351	2.126348	-0.981605
H	-1.069945	-2.448770	-0.818907
H	-0.843179	-2.457770	0.802447
H	1.963692	1.651386	0.033143
H	0.391136	-2.809199	-0.204215
C	-2.651793	0.279133	0.291984
C	-3.973083	0.769993	-0.236279
H	-2.735156	-0.768144	0.580993
H	-2.354911	0.843268	1.176559
H	-4.270905	0.151463	-1.086558
H	-3.854118	1.795657	-0.612806
N	-4.966726	0.642456	0.793982
H	-4.842128	1.342117	1.512701
H	-5.897675	0.756356	0.421570
Cl	4.084162	0.969480	0.166824
O	2.025607	-1.005457	0.516557
H	2.018959	-1.234303	1.452024
H	2.747082	-0.313288	0.393984

IdoA(2S)-model (Via aquation)

MS3

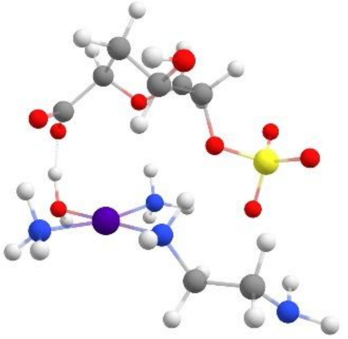

N	0.523879	-1.447355	0.951993
H	1.014101	-0.553276	0.855657
H	0.319953	-1.545705	1.942493
N	-2.424637	-1.651142	1.403619
Pt	-1.159555	-1.278193	-0.145708
N	-0.050286	-0.609403	-1.726443
H	-2.044941	-2.024961	2.265314
H	-3.169874	-2.271555	1.104587
H	-0.272037	-1.074423	-2.600259
H	0.966424	-0.651120	-1.604537
H	-2.835927	-0.728801	1.589575
H	-0.298139	0.379579	-1.817637
C	1.434794	-2.516133	0.540728
C	2.777532	-2.395639	1.222068
H	0.960726	-3.477376	0.735044
H	1.586201	-2.429532	-0.533242
H	2.758122	-2.897791	2.191721
H	2.980634	-1.332036	1.411884
N	3.776180	-3.006657	0.385890
H	3.934312	-2.401939	-0.412522
H	4.653200	-3.105619	0.876130
O	-2.895577	-1.105959	-1.195495
H	-2.873442	-1.461982	-2.085886
H	-3.143616	-0.053497	-1.204521
C	0.497964	2.925234	-0.924709
C	1.143692	2.347257	0.306009
C	0.121914	2.198645	1.410643
C	-1.030444	3.139728	1.179954
C	-1.703863	2.793914	-0.129407
C	-2.700678	1.654943	-0.005517
O	1.615028	1.034810	0.030038
O	0.773243	2.406435	2.625653
O	-0.767479	2.358951	-1.097935
O	-2.956168	1.170913	1.095129
O	-3.174935	1.261216	-1.108378
S	3.078275	0.809607	-0.617655
O	2.880427	-0.519638	-1.188505
O	3.250130	1.870778	-1.588543
O	4.012417	0.846572	0.487340
H	1.095534	2.686359	-1.802134
H	1.970451	2.972252	0.653723
H	-0.272943	1.176505	1.355614
H	-0.672941	4.171815	1.184443
H	-2.239130	3.666318	-0.523287
H	0.188586	2.175997	3.349352
H	-1.762649	3.032539	1.980326
H	0.432192	4.015173	-0.842627

TS2

(-215.74 cm⁻¹)

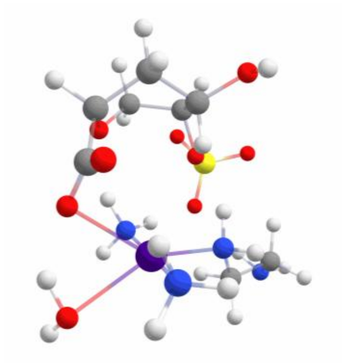

N	0.404535	-1.482001	0.988543
H	0.993945	-0.647417	0.921270
H	0.178782	-1.587595	1.973474
N	-2.533364	-1.199521	1.475845
Pt	-1.236374	-1.025442	-0.080398
N	-0.076752	-0.601793	-1.695616
H	-2.178954	-1.487325	2.380562
H	-3.265911	-1.851156	1.208394
H	-0.340313	-1.129741	-2.520605
H	0.934335	-0.698767	-1.567318
H	-2.957231	-0.266315	1.571408
H	-0.283716	0.389965	-1.857385
C	1.183945	-2.638140	0.545394
C	2.530932	-2.685289	1.229107
H	0.600889	-3.541521	0.718086
H	1.346681	-2.541567	-0.525470
H	2.454797	-3.210198	2.183702
H	2.848547	-1.655822	1.449412
N	3.459067	-3.376784	0.374903
H	3.690631	-2.767038	-0.401363
H	4.315348	-3.592535	0.864284
O	-3.309866	-1.645522	-1.163988
H	-3.255924	-2.090600	-2.011084
H	-3.427057	-0.686749	-1.318904
C	0.751689	2.814715	-1.074424
C	1.323964	2.290913	0.215044
C	0.279732	2.317874	1.312315
C	-0.857833	3.225588	0.927860
C	-1.491337	2.729507	-0.354992
C	-2.469758	1.591025	-0.108219
O	1.702906	0.929552	0.058185
O	0.911676	2.704335	2.493490
O	-0.526462	2.288419	-1.282225
O	-3.123573	1.582422	0.934291
O	-2.541851	0.720552	-1.021562
S	3.153393	0.547256	-0.543968
O	2.840574	-0.762486	-1.108857
O	3.463443	1.572623	-1.519042
O	4.052145	0.499088	0.589757
H	1.375869	2.499929	-1.907992
H	2.188268	2.883234	0.525725
H	-0.114419	1.297854	1.407011
H	-0.490979	4.248358	0.819553
H	-2.074290	3.541768	-0.807998
H	0.302168	2.620665	3.228594
H	-1.625182	3.223448	1.701810
H	0.732251	3.909191	-1.059269

MS4

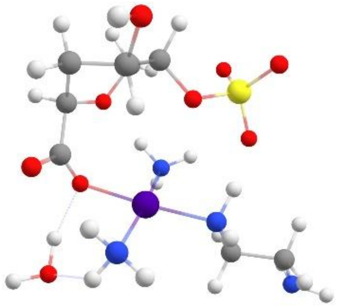

N	2.093340	0.241082	0.719580
H	1.713493	1.189822	0.595970
H	2.189379	0.104289	1.721693
N	0.751493	-2.334176	1.496293
Pt	0.714697	-1.009388	-0.059091
N	0.550966	0.188253	-1.688013
H	1.323840	-2.107904	2.300738
H	1.053420	-3.233511	1.107600
H	1.100330	-0.141471	-2.474042
H	0.794153	1.161528	-1.498899
H	-0.206717	-2.483082	1.812655
H	-0.441046	0.125128	-1.932548
C	3.417555	0.195320	0.103847
C	4.321169	1.258535	0.668406
H	3.849233	-0.793575	0.254687
H	3.295437	0.340550	-0.971429
H	4.475207	1.068179	1.733680
H	3.815786	2.231150	0.585335
N	5.595752	1.205334	0.004497
H	5.526780	1.548080	-0.944012
H	6.279812	1.775539	0.479390
O	0.714653	-4.359499	-0.422149
H	0.254629	-5.196643	-0.367119
H	0.065401	-3.687604	-0.707240
C	-2.716318	1.189848	-1.284795
C	-2.370242	1.616619	0.114079
C	-2.776125	0.556326	1.120843
C	-3.634102	-0.493467	0.464756
C	-2.886796	-1.101122	-0.701217
C	-1.838127	-2.086556	-0.219062
O	-0.964751	1.758886	0.232477
O	-3.425404	1.195876	2.176369
O	-2.285050	-0.124396	-1.514398
O	-2.073004	-2.786365	0.745800
O	-0.747511	-2.162657	-0.910731
S	-0.270238	3.159645	-0.103108
O	1.111565	2.706000	-0.288421
O	-0.893388	3.643354	-1.320326
O	-0.464258	4.012364	1.049834
H	-2.198464	1.826091	-2.000393
H	-2.869876	2.556409	0.364341
H	-1.847318	0.086807	1.469722
H	-4.576267	-0.051034	0.136448
H	-3.583324	-1.688638	-1.312170
H	-3.602977	0.565954	2.876824
H	-3.867115	-1.292146	1.168382
H	-3.792128	1.289052	-1.456052

MS3'

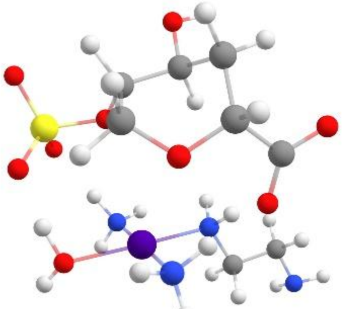

Pt	0.844576	-1.249121	0.356428
N	2.031687	-0.003215	-0.683775
N	0.489490	-2.427018	-1.280769
C	3.461479	-0.295468	-0.627190
H	1.869694	0.946298	-0.297636
H	1.737197	0.039789	-1.655777
N	1.135475	-0.097642	1.998733
H	0.953926	-2.127140	-2.131176
H	-0.522335	-2.429920	-1.469511
H	0.783016	-3.385312	-1.121767
C	4.260920	0.733699	-1.380436
H	3.634582	-1.292851	-1.036076
H	3.771533	-0.310907	0.417827
H	1.973808	-0.356855	2.507343
H	0.351699	-0.193719	2.634963
H	1.189698	0.897590	1.714714
N	5.657248	0.396131	-1.316746
H	4.110286	1.708160	-0.909217
H	3.871916	0.811191	-2.406007
H	5.863823	-0.410763	-1.889444
H	6.233487	1.154981	-1.649422
C	0.836886	3.263002	0.566363
C	-0.604986	3.104916	1.035671
O	1.614900	2.292471	0.744111
O	1.132393	4.350117	0.072059
C	-1.589875	3.523230	-0.040085
O	-0.810425	1.776287	1.431848
H	-0.716837	3.765323	1.905337
C	-1.854035	2.350119	-0.944564
H	-1.155100	4.353911	-0.595757
H	-2.533122	3.868365	0.390670
C	-2.123635	1.332442	1.294713
C	-2.520347	1.249059	-0.159785
O	-2.678620	2.662713	-2.024195
H	-0.895444	1.949923	-1.300802
H	-2.190649	0.348477	1.756730
H	-2.817521	1.999048	1.819258
O	-2.097492	0.037210	-0.772663
H	-3.605357	1.333300	-0.268884
H	-2.222229	3.262965	-2.615583
S	-2.948560	-1.287306	-0.572952
O	-2.735170	-1.699498	0.826693
O	-2.317922	-2.194890	-1.515068
O	-4.327573	-0.955829	-0.839636
O	-0.421964	-2.394318	1.494859
H	-0.266602	-3.341727	1.492187
H	-1.380186	-2.199722	1.239613

TS2'

(-181.83 cm^{-1})

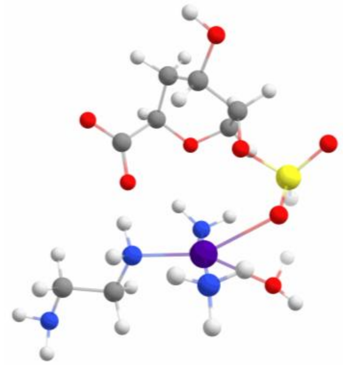

Pt	1.252797	-0.898219	-0.174533
N	2.163505	0.835705	-0.605185
N	1.965224	-1.831192	-1.847280
C	3.348996	1.161705	0.181203
H	1.445321	1.572278	-0.445129
H	2.401484	0.872354	-1.591771
N	0.497027	-0.004572	1.481404
H	2.110183	-1.230063	-2.651568
H	1.273112	-2.524123	-2.121526
H	2.839214	-2.319291	-1.680005
C	3.893464	2.513744	-0.197885
H	4.099630	0.384276	0.030875
H	3.077794	1.161101	1.236935
H	1.188995	0.056636	2.220804
H	-0.283136	-0.551765	1.842738
H	0.147911	0.946766	1.261175
N	5.058236	2.800974	0.594720
H	3.130512	3.268339	0.008511
H	4.080757	2.536037	-1.281303
H	5.844232	2.234150	0.307332
H	5.335427	3.766861	0.500638
C	-0.804636	3.101493	0.004940
C	-2.095795	2.781772	0.754720
O	0.218719	2.451873	0.325846
O	-0.867411	4.000033	-0.834673
C	-3.262381	2.632870	-0.196015
O	-1.912561	1.641298	1.554519
H	-2.282166	3.640992	1.409180
C	-3.168763	1.317509	-0.921665
H	-3.225127	3.463001	-0.901063
H	-4.213626	2.687890	0.338062
C	-2.913317	0.674561	1.472168
C	-3.062441	0.168051	0.064910
O	-4.274373	1.075208	-1.738265
H	-2.246916	1.306112	-1.515959
H	-2.626738	-0.138432	2.137917
H	-3.879318	1.061806	1.814070
O	-1.907525	-0.564517	-0.330620
H	-3.944532	-0.471195	-0.028121
H	-4.327317	1.750561	-2.416599
S	-1.746048	-2.092434	0.056485
O	-1.530061	-2.134814	1.506444
O	-0.511999	-2.431786	-0.658543
O	-2.927200	-2.784797	-0.391231
O	1.132813	-2.782394	1.466463
H	1.333174	-3.622163	1.049748
H	0.177859	-2.789579	1.645475

MS4'

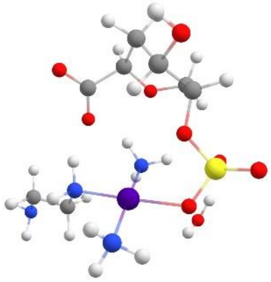

Pt	1.008146	-0.783247	-0.428787
N	2.300769	0.750464	-0.542685
N	1.424319	-1.441732	-2.317202
C	3.524946	0.652574	0.244894
H	1.740903	1.571740	-0.232480
H	2.550575	0.925294	-1.511992
N	0.553622	-0.142072	1.450465
H	1.188809	-0.767358	-3.038357
H	0.864361	-2.271006	-2.496254
H	2.396643	-1.701329	-2.448189
C	4.358897	1.899238	0.113410
H	4.086116	-0.223498	-0.084386
H	3.263509	0.501456	1.291970
H	1.325276	-0.275182	2.095049
H	-0.240581	-0.640213	1.845989
H	0.312016	0.864384	1.392560
N	5.560909	1.755111	0.888690
H	3.789723	2.747468	0.501713
H	4.541537	2.099014	-0.952293
H	6.196793	1.098247	0.457548
H	6.049808	2.633985	0.973186
C	-0.479440	3.105877	0.195099
C	-1.820351	2.824312	0.869926
O	0.508758	2.448911	0.600936
O	-0.468618	3.988235	-0.664058
C	-2.965540	2.796327	-0.118645
O	-1.729005	1.631939	1.608952
H	-1.980321	3.656795	1.566059
C	-2.986099	1.488604	-0.864019
H	-2.826350	3.625509	-0.811925
H	-3.923361	2.938241	0.386734
C	-2.830835	0.785174	1.517784
C	-3.023187	0.316109	0.102509
O	-4.088763	1.372563	-1.710414
H	-2.054393	1.392694	-1.434650
H	-2.644788	-0.056289	2.181409
H	-3.751682	1.276721	1.849811
O	-1.938776	-0.517286	-0.316002
H	-3.965125	-0.225996	-0.012432
H	-4.061782	2.066678	-2.371126
S	-1.765985	-2.012013	0.138728
O	-1.726584	-2.025830	1.594740
O	-0.411293	-2.293689	-0.425894
O	-2.787398	-2.819229	-0.462008
O	0.696902	-3.221497	2.250617
H	0.994690	-3.200347	1.338992
H	-0.221462	-2.926178	2.203251

GlcNS(6S)-model (Via aquation)

MS5

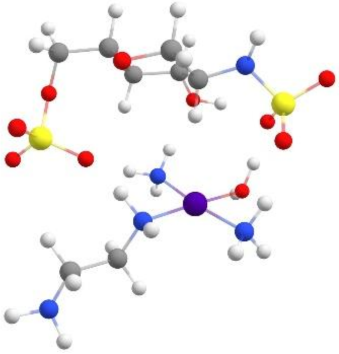

N	1.039394	-1.770738	-0.766118
H	0.839180	-1.910052	-1.752575
H	1.516049	-0.861773	-0.691605
N	0.300958	-0.678259	1.794987
Pt	-0.661506	-1.687384	0.302704
N	-1.772452	-2.553369	-1.165453
H	1.219160	-0.334892	1.499320
H	0.430127	-1.238977	2.630847
H	-2.316028	-3.323257	-0.788960
H	-1.281345	-2.906100	-1.978780
H	-0.264467	0.145610	2.038671
H	-2.425227	-1.819624	-1.463848
C	1.981667	-2.792586	-0.314409
C	3.264527	-2.745380	-1.100401
H	2.192605	-2.622848	0.741731
H	1.507184	-3.771151	-0.404694
H	3.745768	-1.779639	-0.926258
H	3.032958	-2.800327	-2.173876
N	4.141055	-3.789596	-0.643515
H	3.816712	-4.698088	-0.945455
H	5.075164	-3.669066	-1.005909
C	-0.563058	2.788892	-1.533728
C	-1.321821	2.055381	-0.453001
C	-0.964119	2.597918	0.915029
C	0.520663	2.777660	1.072583
C	1.131337	3.467034	-0.117015
C	2.628486	3.581612	-0.021784
N	-2.741713	2.197493	-0.697757
O	-1.391843	1.728593	1.928238
O	0.802138	2.774641	-1.291150
O	3.252295	2.550784	0.718006
S	-3.721813	0.895204	-0.576191
O	-3.254673	-0.178591	-1.448778
O	-3.565950	0.494836	0.850460
O	-5.040737	1.392616	-0.893717
S	3.622822	1.153501	0.041375
O	4.747982	0.701574	0.829099
O	2.433467	0.310935	0.233123
O	3.899927	1.425898	-1.353281
H	-0.736567	2.312523	-2.498785
H	-1.038644	0.996004	-0.494088
H	-1.468589	3.568326	1.017088
H	0.995155	1.800591	1.158787
H	0.738124	4.491015	-0.208706
H	3.055335	3.623691	-1.024919
H	2.889622	4.489295	0.518748
H	-3.003858	2.772366	-1.488544
H	-2.289321	1.417350	1.723737
H	-0.923043	3.828135	-1.594451
H	0.730589	3.332579	1.989668
O	-2.430055	-1.672750	1.337678
H	-2.938109	-0.813809	1.171807
H	-2.374431	-1.841351	2.281152

TS3

(-162.90 cm⁻¹)

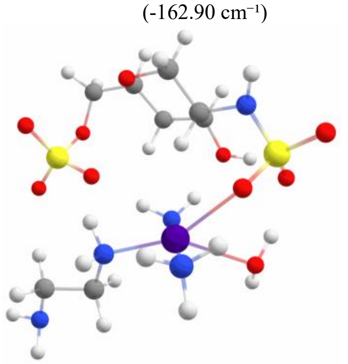

N	0.964944	-2.188197	-0.605525
H	0.956518	-2.650073	-1.510018
H	1.535979	-1.336345	-0.715897
N	0.005560	-0.513066	1.403233
Pt	-0.867855	-1.506132	-0.147602
N	-1.760051	-2.515092	-1.674716
H	0.939499	-0.198218	1.121926
H	0.103951	-1.125025	2.207256
H	-1.990572	-3.472377	-1.429058
H	-1.238811	-2.543608	-2.544614
H	-0.500400	0.322209	1.728957
H	-2.629505	-2.020352	-1.864342
C	1.626043	-3.060707	0.363563
C	3.078443	-3.252455	0.011459
H	1.557517	-2.612653	1.354063
H	1.095216	-4.012947	0.395375
H	3.580642	-2.280896	0.056328
H	3.155582	-3.611966	-1.024711
N	3.686913	-4.131271	0.973926
H	3.392330	-5.088226	0.836584
H	4.693277	-4.113285	0.900949
C	-0.313120	3.064324	-1.343061
C	-1.026968	2.261690	-0.282980
C	-0.664435	2.818045	1.074420
C	0.830688	2.895773	1.221617
C	1.505793	3.529433	0.031341
C	3.000596	3.373567	0.084165
N	-2.455582	2.319051	-0.508473
O	-1.139429	2.008025	2.113578
O	1.058543	2.968909	-1.171426
O	3.437470	2.137434	0.616122
S	-3.378480	1.004716	-0.186315
O	-2.711475	-0.198899	-0.724455
O	-3.422018	0.902224	1.283936
O	-4.645327	1.283711	-0.818168
S	3.506139	0.825531	-0.293320
O	4.490992	0.012520	0.387963
O	2.159974	0.239492	-0.223780
O	3.867922	1.264574	-1.624640
H	-0.546884	2.675401	-2.334714
H	-0.667854	1.229015	-0.370207
H	-1.110980	3.818769	1.145740
H	1.222573	1.884833	1.333067
H	1.293829	4.609069	0.007243
H	3.418143	3.508309	-0.914677
H	3.422344	4.118872	0.755994
H	-2.739585	2.715114	-1.396116
H	-2.085385	1.816227	1.989491
H	-0.637116	4.115628	-1.298221
H	1.084327	3.446337	2.130105
O	-2.764432	-1.707821	1.484317
H	-3.094538	-0.800022	1.611871
H	-2.532421	-2.058798	2.344911

MS6

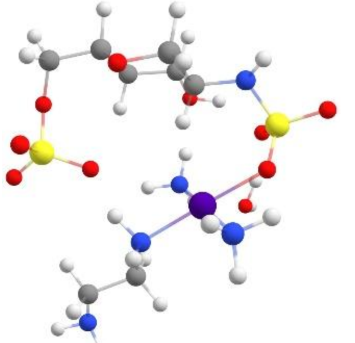

N	0.912548	-2.116219	-0.586652
H	1.089932	-2.433362	-1.535613
H	1.585836	-1.352484	-0.405732
N	-0.357556	-0.619635	1.409209
Pt	-0.889288	-1.239540	-0.472112
N	-1.425051	-1.877886	-2.332170
H	0.653965	-0.639884	1.526203
H	-0.821750	-1.217133	2.099548
H	-1.458249	-2.888707	-2.417641
H	-0.826139	-1.526619	-3.072608
H	-0.633243	0.336526	1.658458
H	-2.362316	-1.527469	-2.514682
C	1.179419	-3.219030	0.332923
C	2.627153	-3.628889	0.272027
H	0.933582	-2.907396	1.348016
H	0.524096	-4.053413	0.079183
H	3.244480	-2.778017	0.574793
H	2.892051	-3.860049	-0.769600
N	2.856498	-4.717284	1.182698
H	2.446645	-5.574553	0.838015
H	3.843307	-4.886848	1.309341
C	-0.256838	2.852887	-1.438533
C	-0.930470	2.183057	-0.266967
C	-0.519010	2.884882	1.005790
C	0.981576	2.928893	1.083402
C	1.627835	3.405209	-0.191724
C	3.121014	3.212488	-0.170766
N	-2.367750	2.166511	-0.445771
O	-0.949328	2.197093	2.145068
O	1.118420	2.725677	-1.308597
O	3.558650	2.106208	0.593435
S	-3.273515	0.898859	0.033640
O	-2.751732	-0.371369	-0.568869
O	-3.117588	0.825914	1.487179
O	-4.596789	1.165019	-0.460643
S	3.580424	0.625550	-0.008278
O	4.656177	-0.002994	0.727899
O	2.267403	0.056367	0.324832
O	3.795546	0.764202	-1.434271
H	-0.544917	2.365727	-2.370753
H	-0.532495	1.162767	-0.258149
H	-0.947751	3.895913	0.986856
H	1.329351	1.913961	1.285383
H	1.439051	4.479455	-0.335343
H	3.489248	3.120926	-1.193493
H	3.593551	4.067961	0.308173
H	-2.711942	2.520659	-1.329773
H	-1.900798	2.023831	2.097750
H	-0.545731	3.913396	-1.496078
H	1.292613	3.560533	1.918270
O	-2.608866	-1.546941	2.717869
H	-2.954556	-0.720661	2.341842
H	-2.953981	-1.620287	3.607243

MS5'

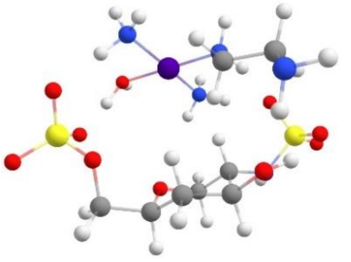

N	-1.031867	-1.882365	0.878469
H	-1.865932	-1.651649	0.321715
H	-1.156347	-2.842967	1.188989
N	-0.568006	-1.507216	-1.954136
Pt	0.605196	-1.764373	-0.300072
N	1.878515	-2.099569	1.253414
H	-1.537876	-1.244780	-1.734784
H	-0.606666	-2.368953	-2.490805
H	1.455376	-2.175342	2.170350
H	2.590589	-1.355847	1.294727
H	-0.189694	-0.795959	-2.572194
H	2.367775	-2.973675	1.086904
C	-1.030736	-0.986363	2.035498
C	-2.344543	-0.995586	2.768501
H	-0.226317	-1.255388	2.718687
H	-0.838479	0.024939	1.677673
H	-2.498455	-1.969359	3.241496
H	-3.153194	-0.842170	2.041393
N	-2.289598	0.024526	3.784115
H	-2.259568	0.936529	3.340536
H	-3.103708	-0.008867	4.379851
C	-0.687886	2.233814	-1.791271
C	-1.601981	1.672956	-0.728734
C	-1.540069	2.576151	0.483256
C	-0.102212	2.735201	0.902335
C	0.793680	3.131592	-0.244961
C	2.243720	3.075328	0.118144
N	-2.936397	1.564034	-1.273023
O	-2.244192	2.069387	1.574539
O	0.620108	2.274980	-1.338094
O	2.515970	1.821111	0.719296
S	-3.878043	0.300561	-0.790856
O	-3.049528	-0.923069	-0.840626
O	-4.248581	0.606393	0.591262
O	-4.974423	0.283680	-1.734165
S	3.790003	1.016269	0.228674
O	4.913999	1.920006	0.283403
O	3.825481	-0.094204	1.164766
O	3.488893	0.581829	-1.144975
H	-0.705446	1.607288	-2.684891
H	-1.212839	0.685088	-0.446191
H	-1.955813	3.548628	0.180993
H	0.249369	1.780945	1.303225
H	0.571674	4.161839	-0.560157
H	2.848220	3.205723	-0.780184
H	2.493017	3.857294	0.835502
H	-2.988283	1.646755	-2.281232
H	-3.085509	1.671848	1.282203
H	-1.031162	3.238538	-2.080076
H	-0.032063	3.474673	1.703218
O	2.311016	-1.608531	-1.417052
H	2.284112	-1.902148	-2.330167
H	2.772270	-0.708262	-1.351541

TS3'
(-175.54 cm⁻¹)

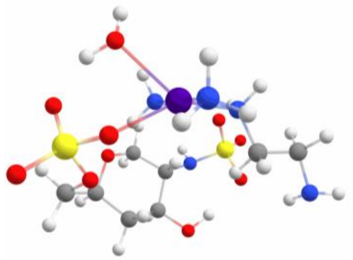

N	-0.641619	-2.323001	-0.120143
H	-1.291784	-1.924511	-0.806907
H	-0.541528	-3.306361	-0.360158
N	0.370930	-0.005673	-1.485496
Pt	1.157563	-1.436534	-0.271002
N	2.043854	-2.834352	0.912885
H	-0.640399	-0.101870	-1.614008
H	0.796867	-0.073834	-2.404966
H	1.433495	-3.410313	1.482413
H	2.667639	-2.333197	1.541288
H	0.554422	0.943502	-1.139781
H	2.615424	-3.464469	0.358273
C	-1.314170	-2.191081	1.173564
C	-2.588409	-2.994904	1.204338
H	-0.643095	-2.495396	1.976700
H	-1.553226	-1.136349	1.310435
H	-2.351848	-4.058990	1.284427
H	-3.111425	-2.849046	0.248518
N	-3.368358	-2.590586	2.341714
H	-3.794326	-1.695415	2.131811
H	-4.108141	-3.250989	2.529612
C	-1.225010	2.779377	-1.366119
C	-2.048401	2.025892	-0.357541
C	-2.094824	2.802450	0.929805
C	-0.663891	2.980896	1.382092
C	0.261751	3.505171	0.303847
C	1.699747	3.279856	0.658293
N	-3.349684	1.671696	-0.869244
O	-2.812378	2.135810	1.920346
O	0.092280	2.838484	-0.922435
O	1.836140	1.886241	0.872895
S	-3.650848	0.053475	-0.782345
O	-2.506994	-0.649240	-1.400225
O	-3.688837	-0.192088	0.663096
O	-4.897364	-0.152132	-1.485731
S	3.194840	1.180005	0.470033
O	4.240871	1.763064	1.273701
O	2.887459	-0.217154	0.787010
O	3.353088	1.380641	-0.968420
H	-1.218527	2.260419	-2.326167
H	-1.502331	1.103117	-0.130866
H	-2.566226	3.775297	0.735091
H	-0.311189	1.998428	1.706912
H	0.101962	4.579741	0.148395
H	2.335730	3.591894	-0.171801
H	1.979272	3.815634	1.565687
H	-3.570656	2.034246	-1.788774
H	-3.225887	1.334151	1.559737
H	-1.629384	3.787659	-1.525993
H	-0.619716	3.648345	2.245303
O	3.149836	-1.204378	-1.647596
H	3.043962	-1.467790	-2.562809
H	3.377702	-0.256473	-1.626072

MS6'

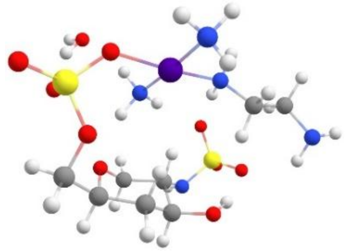

N	-1.068474	-2.013883	-0.331332
H	-1.586667	-1.411738	-0.984755
H	-1.050469	-2.940875	-0.750726
N	0.536557	0.027627	-1.569011
Pt	0.830715	-1.383481	-0.129138
N	1.244869	-2.787614	1.298670
H	-0.431713	0.102388	-1.881498
H	1.135356	-0.172542	-2.380303
H	0.839461	-2.565650	2.202476
H	2.253884	-2.798170	1.425681
H	0.829296	0.943095	-1.213046
H	0.963680	-3.733004	1.059850
C	-1.870425	-2.043948	0.893228
C	-3.226053	-2.650178	0.636979
H	-1.347607	-2.604861	1.667096
H	-1.987432	-1.015605	1.237286
H	-3.128368	-3.733748	0.531899
H	-3.604810	-2.259359	-0.316629
N	-4.089416	-2.354347	1.747654
H	-4.391397	-1.390844	1.667463
H	-4.913416	-2.937139	1.734443
C	-0.538167	2.994727	-0.731980
C	-1.516825	2.099669	-0.020778
C	-1.464416	2.367804	1.459442
C	-0.035811	2.168236	1.904107
C	0.954250	2.937402	1.060674
C	2.371949	2.552815	1.350676
N	-2.838179	2.233519	-0.584307
O	-2.272505	1.490636	2.178943
O	0.753693	2.699741	-0.309264
O	2.485048	1.136974	1.289591
S	-3.562763	0.800660	-0.966213
O	-2.564290	-0.035976	-1.660415
O	-3.898094	0.230394	0.342021
O	-4.694627	1.157790	-1.794248
S	3.409227	0.449660	0.216111
O	4.751059	0.396818	0.726176
O	2.811509	-0.913305	0.158566
O	3.239004	1.177598	-1.027088
H	-0.574424	2.823811	-1.809403
H	-1.173303	1.068836	-0.157572
H	-1.782909	3.405727	1.634703
H	0.186223	1.099589	1.818359
H	0.859349	4.016306	1.247227
H	3.036419	3.017316	0.622170
H	2.658338	2.854242	2.356881
H	-2.908837	2.877514	-1.362767
H	-2.997748	1.159013	1.624274
H	-0.774159	4.051494	-0.546015
H	0.076215	2.451349	2.953112
O	2.748172	-0.239679	-3.261036
H	2.921756	0.158539	-4.113774
H	3.137779	0.336505	-2.583794

Direct Substitution

IdoA(2S)-Model (Direct Substitution)

Msa

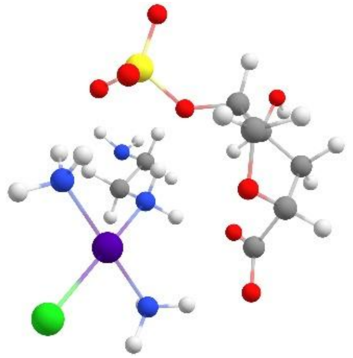

N	-0.271979	-0.186104	1.187333
H	0.532475	-0.264872	0.557248
H	-0.139952	0.739812	1.616514
N	-2.614990	1.477767	0.965220
Pt	-1.938640	-0.200642	0.036098
N	-1.252940	-1.861101	-0.936509
H	-2.664179	1.372522	1.972758
H	-3.555119	1.682817	0.641940
H	-2.032128	-2.376749	-1.331505
H	-0.706264	-2.497295	-0.357286
H	-2.017676	2.303722	0.756377
H	-0.614829	-1.624191	-1.700005
C	-0.173303	-1.256651	2.170131
C	1.196460	-1.315396	2.804882
H	-0.960103	-1.136793	2.914844
H	-0.345636	-2.204667	1.661639
H	1.250217	-0.633320	3.656213
H	1.931899	-0.974622	2.065362
N	1.441089	-2.660755	3.257533
H	1.592241	-3.252719	2.448954
H	2.273040	-2.709381	3.827615
C	1.971124	1.175967	-1.946802
C	2.823316	0.528282	-0.892189
C	3.088575	1.484182	0.255199
C	2.699539	2.893561	-0.111718
C	1.266554	2.977152	-0.595266
C	0.221948	2.995495	0.510310
O	2.140321	-0.575614	-0.308432
O	4.443827	1.381379	0.585744
O	0.901534	1.838287	-1.350801
O	0.504880	2.445158	1.590103
O	-0.884649	3.484397	0.213778
S	2.104334	-2.011547	-1.028242
O	1.400526	-2.788625	-0.019461
O	1.329007	-1.821448	-2.249008
O	3.484277	-2.380668	-1.257996
H	1.553481	0.417854	-2.606918
H	3.772473	0.177844	-1.306491
H	2.463764	1.169294	1.094713
H	3.382033	3.257019	-0.884683
H	1.132889	3.869951	-1.214470
H	4.612771	1.861791	1.397513
H	2.819275	3.543786	0.756835
H	2.583506	1.861107	-2.544254
Cl	-3.855934	-0.217366	-1.288260

Tsa

(-137.85 cm^{-1})

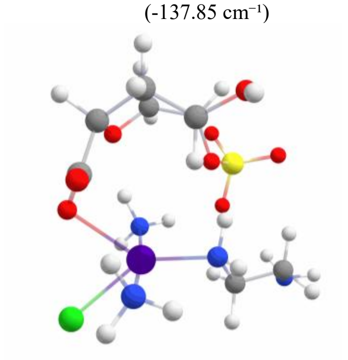

N	0.182447	-1.330430	1.170372
H	0.928694	-0.650464	1.008937
H	-0.022509	-1.296749	2.164175
N	-2.654964	-0.444464	1.631404
Pt	-1.386190	-0.650146	0.055799
N	-0.215222	-0.591052	-1.597932
H	-2.347399	-0.763445	2.542575
H	-3.524968	-0.921798	1.415938
H	-0.669287	-1.080424	-2.362000
H	0.744756	-0.930913	-1.510660
H	-2.838254	0.569318	1.679001
H	-0.186384	0.411164	-1.812453
C	0.690211	-2.654945	0.819958
C	2.031521	-2.932854	1.456659
H	-0.055427	-3.401076	1.092503
H	0.813652	-2.686978	-0.260503
H	1.900175	-3.325516	2.467483
H	2.580618	-1.984286	1.546029
N	2.731825	-3.900777	0.653962
H	3.034491	-3.442936	-0.198797
H	3.554801	-4.242502	1.128604
C	1.377015	2.564135	-1.254391
C	1.914984	1.995094	0.030786
C	0.986903	2.313545	1.184281
C	0.059345	3.436818	0.808653
C	-0.768239	3.022997	-0.389889
C	-1.951762	2.145693	0.001736
O	1.972535	0.577519	-0.054764
O	1.774000	2.597587	2.300225
O	0.002657	2.331329	-1.347258
O	-2.498766	2.362374	1.092021
O	-2.308863	1.275452	-0.828556
S	3.264740	-0.148424	-0.695927
O	2.655483	-1.404047	-1.121442
O	3.706452	0.709808	-1.777307
O	4.217004	-0.288955	0.386297
H	1.852762	2.070557	-2.099368
H	2.909239	2.394524	0.247093
H	0.374987	1.419751	1.363554
H	0.637378	4.340619	0.603937
H	-1.193149	3.919202	-0.860121
H	1.215454	2.694134	3.073134
H	-0.627975	3.652385	1.626294
H	1.603965	3.633586	-1.314122
Cl	-3.371245	-1.650372	-1.347287

MSb

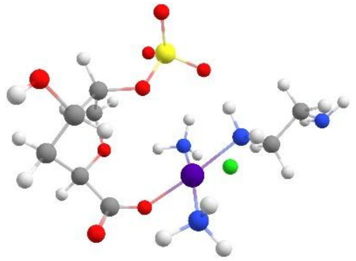

N	-1.773317	0.166021	-1.298280
H	-1.406747	1.104503	-1.088962
H	-1.709270	0.054731	-2.305625
N	-0.216534	-2.343574	-1.922846
Pt	-0.558108	-1.116468	-0.318562
N	-0.738702	0.023532	1.340933
H	-0.111440	-1.869756	-2.812968
H	-0.942841	-3.042326	-2.036422
H	-1.550042	-0.232308	1.919803
H	-0.814228	1.014109	1.111387
H	0.666143	-2.826665	-1.719755
H	0.132796	-0.133322	1.850284
C	-3.178023	0.107415	-0.896585
C	-3.961093	1.266695	-1.450717
H	-3.602394	-0.837638	-1.235932
H	-3.226031	0.109527	0.196046
H	-3.969470	1.215623	-2.542805
H	-3.447534	2.199732	-1.176765
N	-5.317212	1.188770	-0.976148
H	-5.360020	1.355217	0.020442
H	-5.901976	1.880695	-1.421247
C	2.636911	0.936626	1.677149
C	2.624165	1.392446	0.244479
C	3.223670	0.338813	-0.667562
C	3.882919	-0.746484	0.142229
C	2.870107	-1.349778	1.088439
C	1.893966	-2.241000	0.339086
O	1.287677	1.579543	-0.188248
O	4.111286	0.977284	-1.534224
O	2.146270	-0.369047	1.791590
O	2.286940	-2.892229	-0.617209
O	0.696718	-2.273865	0.789798
S	0.579453	2.999589	-0.005158
O	-0.823435	2.602117	-0.143731
O	0.926386	3.460372	1.326657
O	1.063019	3.848912	-1.074144
H	1.983390	1.576798	2.267228
H	3.191287	2.321132	0.135433
H	2.388945	-0.099601	-1.229436
H	4.735571	-0.337920	0.688144
H	3.384336	-1.995173	1.810886
H	4.430239	0.351931	-2.186877
H	4.248990	-1.539257	-0.509815
H	3.650267	1.016447	2.083180
Cl	-3.511944	-0.537161	2.790913

MSa’

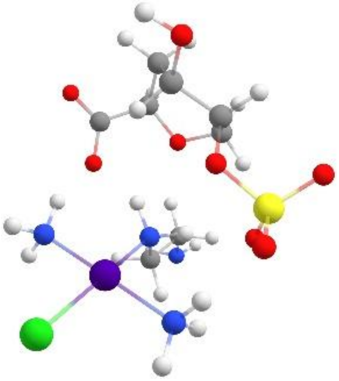

N	0.404587	0.534511	1.012524
H	-0.315905	1.120356	0.555505
H	-0.042095	-0.356197	1.249535
N	2.204538	-1.669215	0.543793
Pt	1.923563	0.177181	-0.277561
N	1.660935	2.014784	-1.111138
H	2.068277	-1.686065	1.549268
H	3.149248	-1.986904	0.353979
H	2.303448	2.686683	-0.704628
H	0.697794	2.368861	-0.977535
H	1.550554	-2.361275	0.158006
H	1.866323	1.981501	-2.103812
C	0.798873	1.194963	2.248714
C	-0.364718	1.309500	3.197747
H	1.609959	0.639320	2.723068
H	1.186188	2.186858	2.001764
H	-0.697667	0.301140	3.462214
H	-1.200663	1.788301	2.673280
N	0.054549	1.987903	4.398271
H	0.230989	2.966397	4.214299
H	-0.661858	1.946536	5.108429
C	-3.053166	-0.327459	0.833778
C	-2.780211	-1.098296	-0.433507
C	-2.898443	-0.192004	-1.635674
C	-3.768046	0.989027	-1.300705
C	-3.104123	1.787644	-0.193312
C	-2.041820	2.716569	-0.766334
O	-1.460585	-1.622082	-0.490877
O	-3.390630	-0.952675	-2.696861
O	-2.509925	0.952741	0.763430
O	-2.431484	3.539026	-1.597696
O	-0.871103	2.575208	-0.346335
S	-1.029016	-2.776552	0.526075
O	0.073313	-3.411821	-0.180454
O	-0.580297	-2.071678	1.727091
O	-2.197943	-3.604330	0.730733
H	-2.597965	-0.821606	1.691789
H	-3.486672	-1.926658	-0.527281
H	-1.885853	0.166333	-1.861864
H	-4.760764	0.640440	-1.005458
H	-3.851768	2.425539	0.292505
H	-3.334103	-0.447177	-3.509004
H	-3.890822	1.640230	-2.166350
H	-4.134847	-0.290074	1.003891
Cl	3.680672	-0.233441	-1.754688

TSa’
(-127.73 cm^{-1})

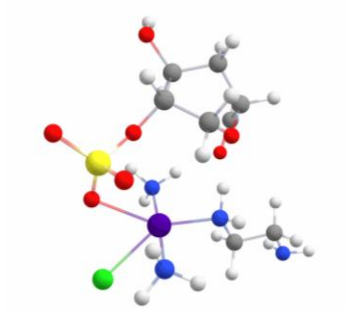

N	0.952545	1.554684	0.655708
H	0.029763	1.822793	0.262125
H	0.811273	1.510384	1.660472
N	2.094731	-0.971912	1.694284
Pt	1.349005	-0.288093	-0.069645
N	0.473989	0.252480	-1.818624
H	2.295247	-0.284915	2.412051
H	2.956435	-1.474085	1.498769
H	1.144143	0.415832	-2.561819
H	-0.130071	1.080467	-1.712448
H	1.403067	-1.630659	2.059766
H	-0.118475	-0.524646	-2.098277
C	1.922352	2.593040	0.337955
C	1.476712	3.934111	0.857807
H	2.896117	2.328096	0.751049
H	2.025438	2.631125	-0.748882
H	1.427588	3.897251	1.949576
H	0.458209	4.125005	0.493015
N	2.433123	4.940946	0.477569
H	2.395019	5.117754	-0.517156
H	2.245085	5.817098	0.942273
C	-2.137123	-0.085016	1.628731
C	-2.598984	-1.019104	0.539673
C	-3.512947	-0.306297	-0.433762
C	-4.016523	0.978013	0.165529
C	-2.837419	1.871468	0.487563
C	-2.286928	2.529728	-0.775391
O	-1.513545	-1.492713	-0.243754
O	-4.536305	-1.187774	-0.783439
O	-1.805269	1.172763	1.129617
O	-3.104761	3.131021	-1.473900
O	-1.055297	2.430523	-0.990022
S	-0.604668	-2.702351	0.260005
O	0.615304	-2.464183	-0.526032
O	-0.401455	-2.529570	1.691250
O	-1.293764	-3.921519	-0.097793
H	-1.245196	-0.489634	2.101678
H	-3.136091	-1.866728	0.975616
H	-2.904157	-0.065450	-1.314918
H	-4.608801	0.762178	1.057854
H	-3.174706	2.688433	1.136303
H	-5.054737	-0.813413	-1.497428
H	-4.654996	1.508731	-0.540627
H	-2.923978	-0.013800	2.388408
Cl	3.490665	-1.491196	-1.234338

MSb’

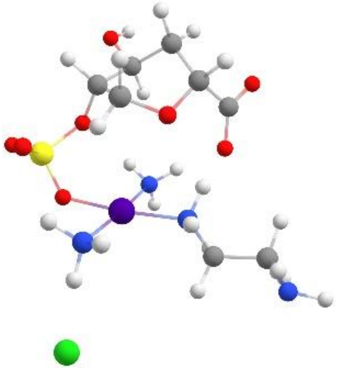

N	1.219553	1.580378	0.490877
H	0.283984	2.018645	0.390669
H	1.433374	1.582586	1.483911
N	1.938100	-1.172821	1.359844
Pt	0.949380	-0.291384	-0.184897
N	-0.115323	0.499623	-1.728805
H	1.999699	-0.621628	2.208638
H	2.903991	-1.404272	1.071387
H	0.445258	0.690337	-2.552333
H	-0.577962	1.373100	-1.425819
H	1.447545	-2.032772	1.600408
H	-0.832991	-0.168977	-1.994099
C	2.203530	2.389261	-0.217698
C	2.228086	3.802514	0.299550
H	3.187578	1.929045	-0.124169
H	1.938474	2.387987	-1.277342
H	2.551344	3.796492	1.343957
H	1.203184	4.197996	0.279176
N	3.168577	4.577417	-0.465855
H	2.821742	4.748522	-1.399945
H	3.333021	5.477493	-0.039776
C	-1.904212	-0.322726	1.613069
C	-2.650035	-1.060209	0.529488
C	-3.568025	-0.108455	-0.205593
C	-3.901846	1.073285	0.666321
C	-2.630611	1.828141	1.012575
C	-2.251249	2.840474	-0.062380
O	-1.803472	-1.593330	-0.494341
O	-4.690888	-0.828457	-0.607569
O	-1.549741	0.958115	1.202033
O	-3.128114	3.640608	-0.387929
O	-1.078363	2.796414	-0.509800
S	-0.749324	-2.756361	-0.253610
O	0.477705	-2.167971	-0.882938
O	-0.597483	-2.936001	1.172771
O	-1.194769	-3.896579	-1.004309
H	-0.991644	-0.849379	1.876356
H	-3.237732	-1.873905	0.959670
H	-3.015949	0.251584	-1.083391
H	-4.415818	0.725068	1.565342
H	-2.799031	2.407088	1.929486
H	-5.226296	-0.294152	-1.196197
H	-4.573872	1.755963	0.145624
H	-2.541121	-0.288851	2.504619
Cl	4.852445	-1.635677	0.288576

GlcNS(6S)-model (Direct substitution)

MSc

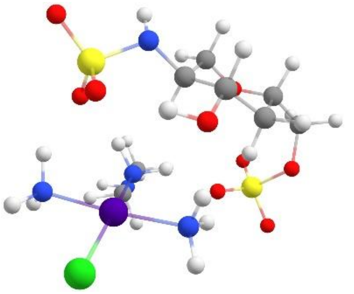

N	-0.215943	-0.924608	-1.066372
H	-0.570111	-0.146577	-1.629418
H	0.687071	-0.648610	-0.669060
N	-0.140135	-1.020881	1.853249
Pt	-1.574643	-1.195314	0.423530
N	-3.043394	-1.155724	-0.988620
H	0.798305	-1.260401	1.523951
H	-0.359192	-1.575715	2.672889
H	-3.956307	-1.192240	-0.546600
H	-3.011196	-1.903960	-1.672286
H	-0.140770	-0.029421	2.120832
H	-2.948504	-0.255618	-1.471461
C	0.059897	-2.088679	-1.900689
C	1.130293	-1.781303	-2.914307
H	0.384877	-2.905056	-1.256091
H	-0.860505	-2.403434	-2.397303
H	2.049136	-1.526312	-2.378119
H	0.833735	-0.890354	-3.487244
N	1.372242	-2.939158	-3.732945
H	0.608367	-3.099164	-4.375267
H	2.209447	-2.828181	-4.285559
C	1.657001	2.802454	-1.051178
C	0.411055	2.419171	-0.293898
C	0.645264	2.539664	1.194706
C	1.935040	1.858916	1.577223
C	3.085651	2.203359	0.668119
C	4.304696	1.366653	0.941348
N	-0.682784	3.247482	-0.749530
O	-0.373373	1.923400	1.931313
O	2.728895	2.012618	-0.672857
O	4.027472	0.045981	1.359606
S	-2.143443	2.517343	-0.977160
O	-1.938665	1.323760	-1.816702
O	-2.570878	2.147906	0.374869
O	-2.958367	3.530534	-1.618614
S	3.656568	-1.112386	0.319291
O	4.183357	-2.295448	0.965589
O	2.189442	-1.119688	0.261174
O	4.276081	-0.760282	-0.941025
H	1.500969	2.656170	-2.120760
H	0.202027	1.368492	-0.510778
H	0.691312	3.611267	1.433173
H	1.783842	0.781468	1.512531
H	3.378439	3.256271	0.798080
H	4.932455	1.344995	0.049461
H	4.866434	1.795554	1.769162
H	-0.464995	3.820382	-1.556148
H	-1.235753	2.056312	1.492103
H	1.882518	3.866291	-0.877799
H	2.186039	2.095939	2.613657
Cl	-3.161794	-1.533785	2.085492

TSb

(-122.27 cm^{-1})

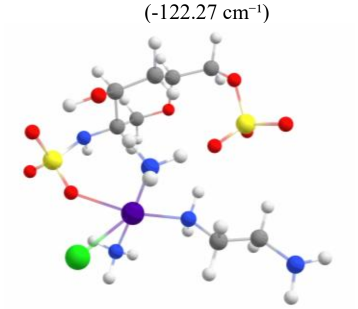

N	0.619758	-1.971505	-0.827311
H	0.543508	-2.189237	-1.815987
H	1.330440	-1.231983	-0.742589
N	-0.114489	-0.528281	1.501166
Pt	-1.120484	-1.205103	-0.133820
N	-2.215343	-1.875691	-1.709590
H	0.896856	-0.494111	1.356132
H	-0.320028	-1.139270	2.285522
H	-2.732320	-2.704580	-1.432211
H	-1.731843	-2.085120	-2.575499
H	-0.406740	0.425265	1.761253
H	-2.893844	-1.140916	-1.898327
C	1.113283	-3.144688	-0.110668
C	2.489177	-3.538551	-0.580233
H	1.145267	-2.913629	0.954362
H	0.403857	-3.962449	-0.248014
H	3.183114	-2.725822	-0.349191
H	2.478766	-3.655037	-1.673530
N	2.913062	-4.719923	0.122502
H	2.406865	-5.535478	-0.194179
H	3.895094	-4.900588	-0.024153
C	0.474597	2.871463	-1.529689
C	-0.449117	2.279390	-0.491068
C	-0.102898	2.875311	0.857389
C	1.375981	2.743305	1.112436
C	2.228734	3.175669	-0.051266
C	3.679148	2.828875	0.142027
N	-1.800850	2.578785	-0.916064
O	-0.740410	2.219838	1.918239
O	1.796615	2.579209	-1.241582
O	3.906002	1.616225	0.833658
S	-3.094583	1.805777	-0.245890
O	-2.845230	0.351997	-0.222599
O	-3.205054	2.334416	1.116754
O	-4.197000	2.158091	-1.116754
S	3.795542	0.207766	0.086738
O	4.631289	-0.650421	0.898835
O	2.375770	-0.158561	0.167569
O	4.254340	0.421269	-1.270279
H	0.256060	2.451047	-2.512668
H	-0.269827	1.195670	-0.461495
H	-0.400153	3.932803	0.836272
H	1.591420	1.693945	1.315952
H	2.172655	4.267888	-0.177199
H	4.175653	2.793341	-0.829010
H	4.156674	3.583212	0.764553
H	-1.911647	2.536159	-1.922855
H	-1.709172	2.319677	1.818582
H	0.319985	3.960003	-1.580444
H	1.641250	3.315249	2.004230
Cl	-2.971672	-2.230563	1.550922

MSd

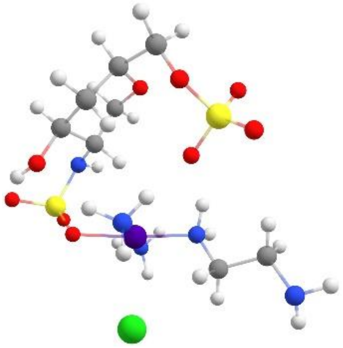

N	-0.874746	1.813834	-1.197144
H	-0.778376	2.027602	-2.185114
H	-1.442550	0.956522	-1.140278
N	-0.117900	0.642164	1.275122
Pt	0.880810	1.270352	-0.371782
N	2.013684	1.851959	-1.981848
H	-0.984117	0.170950	1.001663
H	-0.342152	1.415450	1.919938
H	2.123818	2.860465	-2.013343
H	1.641159	1.563139	-2.880312
H	0.470129	-0.037666	1.767520
H	2.948278	1.446257	-1.903822
C	-1.605969	2.899019	-0.542335
C	-3.043212	2.919032	-0.990298
H	-1.553906	2.771250	0.540195
H	-1.108992	3.841724	-0.778838
H	-3.519309	1.985129	-0.674675
H	-3.080557	2.942198	-2.089274
N	-3.725791	4.020354	-0.365980
H	-3.423298	4.903632	-0.753265
H	-4.723695	3.958736	-0.503683
C	0.660197	-3.064215	-1.251010
C	1.203257	-2.039012	-0.284861
C	1.126970	-2.634010	1.100184
C	-0.315970	-2.985214	1.359523
C	-0.977906	-3.768149	0.249339
C	-2.474439	-3.768263	0.390306
N	2.530590	-1.670974	-0.723764
O	1.500663	-1.740735	2.110791
O	-0.691753	-3.250308	-1.018154
O	-3.012176	-2.516367	0.768993
S	3.426433	-0.526752	0.034872
O	2.534098	0.557075	0.573018
O	4.054201	-1.158737	1.180342
O	4.295169	-0.017670	-1.007348
S	-3.277726	-1.358340	-0.305037
O	-4.354105	-0.604874	0.304250
O	-2.028159	-0.590730	-0.366311
O	-3.609835	-2.020940	-1.549524
H	0.763439	-2.716731	-2.280209
H	0.525202	-1.179118	-0.336496
H	1.765474	-3.526750	1.131695
H	-0.860902	-2.048753	1.492064
H	-0.644336	-4.816092	0.281017
H	-2.927810	-4.089676	-0.548479
H	-2.771916	-4.446400	1.188499
H	2.566800	-1.481329	-1.718392
H	2.459829	-1.608174	2.095174
H	1.213492	-4.009842	-1.151978
H	-0.394264	-3.543434	2.294454
Cl	-0.866306	3.089689	3.139374

MSc'

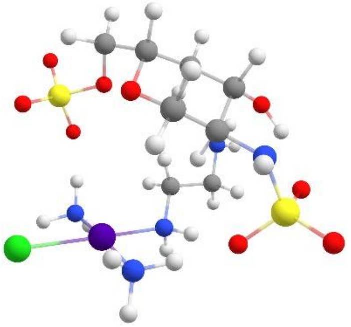

N	-0.513074	-1.294671	1.537624
H	-1.461425	-1.324838	1.142053
H	-0.523413	-1.943495	2.320397
N	-0.734460	-2.570253	-1.070666
Pt	0.800780	-1.970839	0.131891
N	2.393366	-1.459350	1.294080
H	-1.640647	-2.143319	-0.848263
H	-0.846594	-3.576738	-1.000357
H	2.292403	-1.748278	2.261312
H	2.626086	-0.453498	1.287473
H	-0.510788	-2.372904	-2.040536
H	3.209485	-1.943047	0.930489
C	-0.275221	0.060287	2.034745
C	-1.415498	0.540727	2.897085
H	0.662319	0.088174	2.590831
H	-0.145822	0.719348	1.175341
H	-1.414098	-0.020138	3.835349
H	-2.363786	0.315733	2.388242
N	-1.255759	1.946305	3.171923
H	-1.682572	2.481585	2.421005
H	-1.717999	2.202776	4.031366
C	-1.194395	1.254481	-2.289505
C	-2.050306	1.240273	-1.048367
C	-2.142490	2.647709	-0.507782
C	-0.754608	3.201301	-0.318592
C	0.146338	2.995750	-1.512495
C	1.579869	3.249549	-1.162798
N	-3.350397	0.685681	-1.351074
O	-2.787007	2.714943	0.727492
O	0.088482	1.677545	-1.982314
O	1.879509	2.462230	-0.031339
S	-3.991560	-0.351107	-0.243349
O	-2.966572	-1.361527	0.088499
O	-4.267503	0.489398	0.923109
O	-5.154522	-0.915188	-0.894070
S	3.389057	1.979610	0.152313
O	4.183520	3.184292	0.309869
O	3.279874	1.175978	1.365385
O	3.715990	1.210082	-1.035164
H	-1.115078	0.250198	-2.707726
H	-1.535636	0.608360	-0.314353
H	-2.693302	3.239919	-1.253119
H	-0.309299	2.702309	0.544899
H	-0.134702	3.678405	-2.327125
H	2.215423	2.952871	-1.999492
H	1.743358	4.303465	-0.929746
H	-3.441101	0.293477	-2.280674
H	-3.464390	2.017797	0.801748
H	-1.648871	1.906959	-3.050356
H	-0.812169	4.266382	-0.082228
Cl	2.292812	-2.719327	-1.478008

TSb'
(-133.69 cm⁻¹)

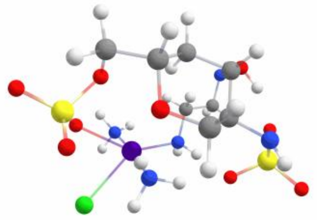

N	-0.524931	-1.961070	0.514527
H	-1.238428	-1.785871	-0.207040
H	-0.436793	-2.972561	0.567044
N	0.458663	-0.166666	-1.599548
Pt	1.274162	-1.188373	-0.038249
N	2.241334	-2.186073	1.446743
H	-0.514569	-0.403442	-1.800457
H	1.015303	-0.408539	-2.415115
H	1.699732	-2.775707	2.069142
H	2.728566	-1.496675	2.013225
H	0.557429	0.843585	-1.458860
H	2.947654	-2.768972	1.004696
C	-1.008068	-1.439498	1.791862
C	-2.251922	-2.130871	2.279355
H	-0.216054	-1.511004	2.537922
H	-1.229366	-0.381213	1.653268
H	-2.012848	-3.150866	2.593224
H	-2.967846	-2.186378	1.450731
N	-2.746330	-1.388454	3.412533
H	-3.011858	-0.454568	3.118600
H	-3.558185	-1.832171	3.816255
C	-1.567456	2.536002	-1.421884
C	-2.285227	1.508269	-0.590940
C	-2.545786	2.072102	0.781245
C	-1.212141	2.478266	1.361694
C	-0.383567	3.330795	0.425560
C	1.039228	3.440069	0.885235
N	-3.471349	1.045455	-1.267900
O	-3.128224	1.130838	1.633396
O	-0.323408	2.788054	-0.865140
O	1.575597	2.139514	1.037647
S	-3.681681	-0.593547	-1.269259
O	-2.360397	-1.208076	-1.512137
O	-4.148357	-0.891381	0.085826
O	-4.640797	-0.845663	-2.323665
S	2.829505	1.731945	0.136588
O	3.942526	2.539420	0.589437
O	2.969638	0.310171	0.475583
O	2.443892	1.953379	-1.242417
H	-1.399954	2.160303	-2.433160
H	-1.594091	0.669733	-0.456301
H	-3.205464	2.945032	0.676486
H	-0.650538	1.565878	1.579857
H	-0.802005	4.345677	0.366049
H	1.617483	4.007412	0.154736
H	1.094368	3.940628	1.851988
H	-3.585151	1.401725	-2.209213
H	-3.682876	0.510405	1.128323
H	-2.161943	3.458001	-1.493889
H	-1.356924	3.009581	2.304894
Cl	3.244633	-1.985555	-1.703042

MSd'

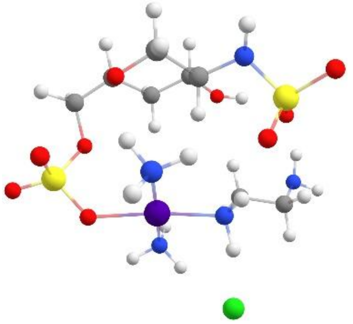

N	-1.119292	-1.668687	-0.345880
H	-1.626566	-1.097152	-1.027461
H	-1.169374	-2.655068	-0.632248
N	0.572589	0.251486	-1.709859
Pt	0.830313	-1.195289	-0.283766
N	1.145584	-2.667509	1.081730
H	-0.409254	0.459108	-1.900872
H	0.998679	-0.045965	-2.581779
H	0.934085	-2.378002	2.030979
H	2.126517	-2.930168	1.065238
H	1.050727	1.113164	-1.428387
H	0.580173	-3.499871	0.857413
C	-1.847610	-1.543081	0.914890
C	-3.261264	-2.039680	0.760361
H	-1.334886	-2.118118	1.686006
H	-1.851969	-0.493902	1.211691
H	-3.244664	-3.129327	0.679659
H	-3.664192	-1.642878	-0.181408
N	-4.033315	-1.646037	1.908504
H	-4.260502	-0.663336	1.816865
H	-4.901361	-2.158993	1.956285
C	-0.135424	3.310083	-0.790785
C	-1.155795	2.506627	-0.030830
C	-0.989418	2.748877	1.445740
C	0.433606	2.390441	1.798992
C	1.445569	3.074091	0.909334
C	2.832890	2.545583	1.110963
N	-2.486241	2.778053	-0.518360
O	-1.846122	1.957581	2.207335
O	1.142988	2.886601	-0.448878
O	2.816369	1.129054	1.027108
S	-3.370441	1.426069	-0.864021
O	-2.495300	0.495158	-1.600193
O	-3.706097	0.895221	0.460636
O	-4.497099	1.901206	-1.639817
S	3.551232	0.399119	-0.170419
O	4.940670	0.254192	0.177329
O	2.865147	-0.924589	-0.141110
O	3.273164	1.143754	-1.374633
H	-0.251440	3.157917	-1.865420
H	-0.925108	1.448550	-0.193289
H	-1.180348	3.813297	1.646620
H	0.539307	1.307078	1.680699
H	1.469035	4.153218	1.118314
H	3.499029	2.964253	0.356132
H	3.200376	2.801195	2.103615
H	-2.531823	3.429449	-1.292605
H	-2.630022	1.705319	1.691806
H	-0.257979	4.382407	-0.583425
H	0.636695	2.636060	2.843836
Cl	-0.876033	-4.807231	-0.078862

Optimized Structures in the Format of xyz Coordinates. 3WAT

Cl-PtN3 + 3 WAT

N	1.316820	-0.748999	0.580514
H	1.342007	-0.105183	1.380703
H	1.452968	-1.689028	0.942278
N	-0.035739	-0.108355	-1.994102
Pt	-0.599127	-0.527222	-0.083009
N	-1.209713	-0.883768	1.829980
H	-0.855194	-0.071409	-2.592126
H	0.605275	-0.780101	-2.400242
H	-1.014517	-1.826263	2.150911
H	-2.212230	-0.743511	1.908930
H	0.399694	0.825129	-1.994525
H	-0.742551	-0.217447	2.451753
C	2.415419	-0.416601	-0.318963
C	3.742753	-0.463268	0.389847
H	2.425218	-1.120711	-1.151699
H	2.232185	0.586272	-0.712582
H	3.930373	-1.481679	0.739237
H	3.692177	0.175991	1.282939
N	4.784928	-0.093453	-0.529094
H	4.747467	0.893565	-0.744120
H	5.698234	-0.281969	-0.143246
Cl	-2.783295	-0.010257	-0.673584
O	-1.064317	2.620363	0.576433
H	-1.520071	3.440863	0.778455
H	-1.726949	1.986561	0.262258
O	0.471772	1.276330	2.391960
H	0.816209	1.803209	3.113654
H	-0.042052	1.865696	1.807656
O	0.914185	2.397964	-1.266998
H	1.087041	3.187337	-1.780270
H	0.216377	2.614310	-0.621916

H_2O-PtN3 + 3 WAT

N	1.989177	0.013376	-0.610179
H	2.487989	-0.853696	-0.796885
H	2.143849	0.571612	-1.446785
N	0.407103	-2.203183	0.405435
Pt	0.013220	-0.372551	-0.415881
N	-0.423268	1.444082	-1.242053
H	1.252746	-2.243744	0.965265
H	0.499771	-2.908849	-0.319295
H	-0.281399	1.442723	-2.247571
H	0.134653	2.202080	-0.861796
H	-0.364814	-2.492877	1.012525
H	-1.408065	1.684030	-1.076879
C	2.603631	0.688422	0.536241
C	4.078716	0.911169	0.337754
H	2.097931	1.641083	0.691261
H	2.425710	0.079659	1.423883
H	4.230796	1.572455	-0.518793
H	4.559279	-0.046312	0.092006
N	4.617551	1.546112	1.509086
H	4.676489	0.898299	2.282663
H	5.547969	1.898494	1.340377
O	-1.973645	-0.777825	-0.254274
H	-2.555006	0.030766	-0.297739
H	-2.197995	-1.340631	0.520555
O	-4.033898	2.773721	1.461141
H	-3.629293	3.622934	1.647007
H	-4.127460	2.317298	2.299184
O	-3.167702	1.436226	-0.559291
H	-3.834868	1.476681	-1.247226
H	-3.500265	1.952347	0.219534
O	-2.135182	-2.461308	1.710625
H	-2.674319	-3.247905	1.596392
H	-2.259455	-2.164214	2.615458

IDOA(2S)-CO-PtN3 + 3 WAT

N	0.971847	1.845927	0.572530
H	0.078301	2.309690	0.356954
H	1.042158	1.840971	1.586667
N	1.849002	-0.720127	1.523540
Pt	0.849966	-0.037269	-0.121780
N	-0.092521	0.631000	-1.794328
H	1.406278	-0.486411	2.405182
H	2.787428	-0.307067	1.495495
H	0.541418	0.812329	-2.564388
H	-0.640590	1.474537	-1.615380
H	1.996135	-1.741530	1.512456
H	-0.723640	-0.128747	-2.056095
C	2.081222	2.625741	0.024032
C	2.124085	4.009256	0.615012
H	3.011730	2.086251	0.208415
H	1.944206	2.684829	-1.058060
H	2.323873	3.935018	1.687336
H	1.134434	4.476152	0.506483
N	3.188483	4.758628	0.003306
H	2.965651	4.989775	-0.955254
H	3.350671	5.627852	0.490006
O	4.253764	0.061451	0.350631
H	5.172686	-0.011335	0.608535
H	4.054220	-0.705178	-0.221961
C	-3.036321	-0.950405	-1.159779
C	-3.013015	-0.430208	0.250183
C	-2.417524	-1.455939	1.197872
C	-2.309469	-2.802130	0.526305
C	-1.521261	-2.708370	-0.771119
C	-0.034455	-2.863993	-0.536473
O	-2.173966	0.710052	0.325099
O	-3.230415	-1.506223	2.331062
O	-1.801779	-1.534146	-1.473425
O	0.327445	-3.935688	-0.074409
O	0.784782	-1.941162	-0.876427
S	-2.740998	2.164570	-0.023918
O	-1.497660	2.823579	-0.438048
O	-3.695301	1.987331	-1.101414
O	-3.305715	2.696777	1.197726
H	-3.194455	-0.127503	-1.853532
H	-4.023589	-0.181383	0.584729
H	-1.417594	-1.096425	1.464292
H	-3.315667	-3.176821	0.328150
H	-1.782247	-3.571240	-1.394899
H	-2.813193	-2.042499	3.007196
H	-1.821099	-3.525723	1.179910
H	-3.853051	-1.667013	-1.290106
O	3.424806	-2.133786	-0.981811
H	3.820657	-2.430456	-1.802369
H	2.448851	-2.122145	-1.096151
O	2.591180	-3.456363	1.246370
H	3.195322	-3.192794	0.537859
H	1.795330	-3.775531	0.775718

IDOA(2S)-SO-PtN3 + 3 WAT

Pt	1.026492	-0.111339	-0.420978
N	1.519462	1.821991	-0.615625
N	1.799069	-0.602575	-2.253636
C	2.650969	2.291287	0.178050
H	0.659468	2.337256	-0.334145
H	1.680941	2.051932	-1.592373
N	0.301709	0.367903	1.418490
H	1.084200	-0.601681	-2.974022
H	2.190683	-1.548631	-2.201292
H	2.541440	0.011061	-2.571045
C	2.861800	3.771859	0.007255
H	3.543244	1.736896	-0.117647
H	2.459021	2.068777	1.227737
H	1.059156	0.421887	2.092699
H	-0.321705	-0.346166	1.783541
H	-0.238624	1.247630	1.356345
N	3.990986	4.188094	0.793930
H	1.971949	4.297745	0.362100
H	2.961239	3.999537	-1.063840
H	4.857275	3.855197	0.393335
H	4.051369	5.194265	0.844323
C	-1.968612	2.835446	0.084741
C	-3.065036	2.030260	0.780196
O	-0.797004	2.697204	0.511623
O	-2.331287	3.595014	-0.814334
C	-4.079887	1.481699	-0.198699
O	-2.483150	1.016951	1.561321
H	-3.571258	2.739856	1.446444
C	-3.542401	0.253373	-0.883738
H	-4.288048	2.262024	-0.930329
H	-5.017078	1.233704	0.304198
C	-3.134114	-0.213620	1.524416
C	-3.106805	-0.787466	0.135090
O	-4.482008	-0.345206	-1.722806
H	-2.644857	0.529511	-1.450567
H	-2.622485	-0.869626	2.225466
H	-4.178307	-0.133535	1.845688
O	-1.774759	-1.128878	-0.262432
H	-3.747096	-1.669540	0.056994
H	-4.734746	0.270125	-2.413141
S	-0.990156	-2.380321	0.272301
O	-0.937777	-2.288683	1.721438
O	0.353974	-2.087552	-0.322402
O	-1.563823	-3.580370	-0.261774
O	1.685112	-1.802533	2.737043
H	2.353083	-2.020293	2.059615
H	0.843133	-2.146399	2.419468
O	3.612249	-2.337797	0.930468
H	3.301206	-2.713028	0.083986
H	4.309568	-2.908589	1.253854
O	2.478562	-3.199587	-1.333852
H	2.655403	-4.050227	-1.738457
H	1.563148	-3.193516	-1.009773

GlcNS(6S)-SO-PtN3 + 3 WAT

N	0.930233	-2.165551	0.425778
H	1.538308	-1.630571	1.058408
H	0.825669	-3.086270	0.846827
N	-0.252073	0.258556	1.459854
Pt	-0.871457	-1.293593	0.307160
N	-1.590457	-2.816331	-0.857529
H	0.726516	0.202534	1.747754
H	-0.832630	0.375355	2.304251
H	-1.062918	-3.681157	-0.812394
H	-1.627710	-2.542880	-1.834597
H	-0.367909	1.115580	0.915189
H	-2.551984	-3.023368	-0.551386
C	1.670239	-2.275776	-0.833544
C	2.933094	-3.075913	-0.644906
H	1.037175	-2.723863	-1.599373
H	1.926588	-1.265794	-1.156349
H	2.684438	-4.136397	-0.553813
H	3.400344	-2.764800	0.299353
N	3.788165	-2.881856	-1.783547
H	4.233539	-1.976259	-1.698566
H	4.516201	-3.580193	-1.815773
C	1.233170	3.042117	0.447008
C	2.082875	1.952197	-0.153572
C	2.145956	2.148199	-1.645448
C	0.724598	2.080472	-2.147713
C	-0.235980	2.986689	-1.411968
C	-1.656968	2.580111	-1.666944
N	3.373795	1.877829	0.483850
O	2.878472	1.156322	-2.288648
O	-0.081010	2.906214	-0.010207
O	-1.759617	1.272865	-1.120656
S	3.819316	0.353377	0.940156
O	2.666781	-0.270311	1.619450
O	4.087514	-0.317990	-0.335405
O	4.968233	0.538201	1.798662
S	-3.157488	0.655090	-0.746838
O	-3.745373	0.076136	-1.921393
O	-2.734909	-0.375173	0.255096
O	-3.926805	1.701520	-0.104657
H	1.198590	2.954294	1.533961
H	1.558492	1.004828	0.008956
H	2.588705	3.133582	-1.848968
H	0.398807	1.042211	-2.030969
H	-0.104216	4.029209	-1.723000
H	-2.359026	3.243881	-1.160550
H	-1.874266	2.557778	-2.735134
H	3.526109	2.531852	1.241878
H	3.464604	0.695835	-1.664573
H	1.629127	4.032483	0.192232
H	0.687795	2.320129	-3.212237
O	-4.053992	-2.755411	0.412648
H	-4.951991	-2.858632	0.097189
H	-3.882230	-1.806952	0.491970
O	-2.303983	3.027413	1.657101
H	-2.986438	2.644593	1.081274
H	-1.514695	3.146395	1.106786
O	-1.966609	1.150280	3.466800
H	-1.689534	1.451822	4.331407
H	-2.110248	1.941893	2.902033

GlcNS(6S)-NS-PtN3 + 3 WAT

N	-0.880005	2.096718	-0.249991
H	-0.806623	2.662006	-1.098347
H	-1.596666	1.371368	-0.383452
N	0.008816	0.222672	1.711093
Pt	0.828666	1.096224	0.051973
N	1.666688	1.910114	-1.616505
H	-1.007931	0.191799	1.638250
H	0.294460	0.694181	2.564011
H	1.358371	2.869180	-1.782437
H	1.394414	1.375331	-2.435699
H	0.324325	-0.747666	1.832892
H	2.695675	1.869600	-1.569558
C	-1.314473	2.983636	0.824035
C	-2.700556	3.505915	0.553964
H	-1.309225	2.445939	1.771879
H	-0.597872	3.803090	0.908368
H	-3.391377	2.657887	0.526127
H	-2.718770	3.969927	-0.442690
N	-3.098148	4.392230	1.614386
H	-2.592946	5.266529	1.567857
H	-4.081658	4.611897	1.558363
C	-0.024045	-2.638651	-1.868442
C	0.665274	-2.158109	-0.613717
C	0.283289	-3.073941	0.524423
C	-1.218291	-3.124959	0.612577
C	-1.895516	-3.377893	-0.709694
C	-3.383014	-3.166380	-0.626992
N	2.090480	-2.094994	-0.869315
O	0.724099	-2.596269	1.764128
O	-1.396412	-2.534616	-1.711976
O	-3.779224	-2.148592	0.270448
S	3.123843	-1.251375	0.068512
O	2.594341	0.105691	0.381567
O	3.271781	-1.979456	1.326893
O	4.332280	-1.144655	-0.726205
S	-3.763031	-0.606200	-0.156201
O	-4.791093	-0.033851	0.687565
O	-2.421657	-0.121305	0.188477
O	-4.036338	-0.573972	-1.578956
H	0.253202	-2.014241	-2.719385
H	0.259042	-1.158792	-0.410748
H	0.706679	-4.067490	0.327334
H	-1.556542	-2.157431	0.988196
H	-1.730937	-4.418278	-1.027525
H	-3.773922	-2.950152	-1.622350
H	-3.859368	-4.064833	-0.238857
H	2.327115	-1.915454	-1.838558
H	1.692618	-2.613534	1.799959
H	0.268005	-3.675721	-2.091148
H	-1.516974	-3.886392	1.335858
O	2.267695	-0.266295	3.347017
H	2.767764	-0.792192	2.708140
H	2.499146	-0.600958	4.213098
O	-0.075407	4.153654	-2.034853
H	-0.013827	5.017518	-1.622024
H	-0.359373	4.306505	-2.938407
O	4.451673	1.469409	-1.526706
H	4.530761	0.532214	-1.281765
H	5.008728	1.611063	-2.292077

Common aquation step

MS1 + 3 WAT

N	1.482977	0.177143	-0.767082
H	1.441905	-0.848977	-0.912186
H	1.675215	0.623333	-1.659401
N	0.181709	0.971732	1.764066
Pt	-0.398458	0.663286	-0.164513
N	-1.072395	0.246643	-2.042598
H	-0.577534	1.395447	2.288781
H	0.996920	1.561763	1.884996
H	-0.458096	0.518264	-2.802174
H	-1.962919	0.706647	-2.203020
H	0.366705	0.048136	2.170836
H	-1.231211	-0.767904	-2.092112
C	2.599081	0.432315	0.132230
C	3.907275	0.004692	-0.478274
H	2.640172	1.493217	0.382851
H	2.425108	-0.132911	1.051274
H	4.102798	0.612292	-1.365567
H	3.815452	-1.036876	-0.816204
N	4.968586	0.219582	0.467688
H	4.931948	-0.458064	1.216802
H	5.873434	0.132750	0.029308
Cl	-2.585416	1.129162	0.494105
O	1.150343	-2.519575	-0.466529
H	0.292167	-2.664578	-0.903842
H	0.929046	-2.385648	0.472922
O	-2.296471	-1.908142	1.026194
H	-3.009535	-2.392658	1.448907
H	-2.570832	-0.974860	0.956779
O	-1.385112	-2.498406	-1.414207
H	-1.823367	-3.205717	-1.889526
H	-1.839579	-2.383955	-0.553789
O	0.208505	-1.864824	2.015333
H	0.371622	-2.424960	2.776045
H	-0.740015	-1.946970	1.785945

TS1 + 3 WAT
(-159.99 cm^{-1})

N	-2.138518	0.209290	-0.669168
H	-2.468333	1.155569	-0.841175
H	-2.286858	-0.274864	-1.551629
N	-0.186318	2.268897	0.002404
Pt	-0.141650	0.246898	-0.262204
N	-0.008315	-1.768301	-0.520345
H	-0.739147	2.548072	0.806364
H	-0.547164	2.770759	-0.802219
H	-0.691924	-2.179209	-1.146827
H	-0.094348	-2.242983	0.373110
H	0.772179	2.601965	0.159960
H	0.925184	-1.995695	-0.891727
C	-2.969693	-0.401327	0.368980
C	-4.425093	-0.425234	-0.014695
H	-2.616740	-1.416771	0.547220
H	-2.821557	0.164110	1.289342
H	-4.550531	-1.046119	-0.905263
H	-4.744375	0.590939	-0.285997
N	-5.185258	-1.004071	1.059351
H	-5.251437	-0.369885	1.843592
H	-6.126858	-1.220074	0.767566
Cl	1.590548	-0.030713	1.704233
O	1.967336	0.590839	-1.376583
H	2.416845	-0.276247	-1.368147
H	2.476903	1.225903	-0.849530
O	3.756942	-2.072331	1.061246
H	3.736291	-2.866597	1.596682
H	3.113088	-1.446085	1.441854
O	2.703640	-1.951216	-1.280614
H	3.127388	-2.414247	-2.004040
H	3.201968	-2.134094	-0.444838
O	2.612273	2.458570	0.435450
H	3.290475	3.100873	0.648317
H	2.564388	1.800534	1.149960

MS2 + 3 WAT

N	-2.183452	0.551654	-0.103823
H	-2.688192	0.937109	0.691361
H	-2.524852	1.090111	-0.897016
N	-0.346447	0.602454	2.148472
Pt	-0.200728	0.881670	0.129160
N	0.000700	1.177980	-1.881637
H	-1.098254	-0.015189	2.436429
H	-0.489135	1.485268	2.629360
H	-0.277553	2.117236	-2.149038
H	-0.544582	0.532764	-2.444239
H	0.526328	0.192613	2.498610
H	0.982786	1.055596	-2.157116
C	-2.557142	-0.853673	-0.283400
C	-4.043252	-1.025674	-0.446476
H	-2.036686	-1.242308	-1.158098
H	-2.198509	-1.413852	0.581593
H	-4.367065	-0.499554	-1.347943
H	-4.557659	-0.553051	0.402131
N	-4.341490	-2.423030	-0.600112
H	-4.238543	-2.918848	0.274578
H	-5.290022	-2.562601	-0.914925
Cl	1.798814	-2.865324	0.620801
O	1.789284	1.235905	0.379693
H	2.297276	1.061191	-0.460948
H	2.146771	0.648693	1.095529
O	3.158528	-1.818431	-1.859110
H	2.894759	-2.339854	-2.618410
H	2.717436	-2.204438	-1.071150
O	2.766970	0.690255	-1.899600
H	3.493737	1.175812	-2.292534
H	2.962872	-0.290295	-1.949768
O	2.267813	-0.436493	2.252547
H	3.001244	-0.459077	2.869361
H	2.201180	-1.314287	1.804822

IDOA(2S)-model (Via aquation)

MS3 + 3 WAT

N	0.224088	-1.276027	1.007962
H	1.011084	-0.671525	0.753120
H	0.011684	-1.051405	1.976911
N	-2.640540	-1.292413	1.313140
Pt	-1.331894	-0.843704	-0.183102
N	-0.011435	-0.300439	-1.654305
H	-2.251054	-1.850759	2.064117
H	-3.445671	-1.806408	0.936557
H	-0.375189	-0.426876	-2.592439
H	0.863393	-0.831467	-1.607564
H	-2.975606	-0.394457	1.706754
H	0.218308	0.694266	-1.544055
C	0.678119	-2.665264	0.904663
C	1.979813	-2.882680	1.638639
H	-0.111016	-3.324658	1.265446
H	0.838392	-2.873683	-0.150898
H	1.791171	-3.111382	2.689780
H	2.562411	-1.951234	1.610882
N	2.673438	-3.984202	1.025976
H	3.019820	-3.680748	0.122579
H	3.469599	-4.265998	1.579326
O	-2.965591	-0.488602	-1.395121
H	-2.783743	-0.529803	-2.337861
H	-3.409716	0.448447	-1.175620
C	2.273840	2.386199	-1.162751
C	2.712657	1.651513	0.074855
C	1.914839	2.114937	1.273839
C	1.334411	3.478417	1.013923
C	0.411747	3.415810	-0.182831
C	-0.992404	2.950909	0.158753
O	2.429281	0.267079	-0.071736
O	2.753895	2.073176	2.385327
O	0.879809	2.501015	-1.156246
O	-1.271652	2.687458	1.332162
O	-1.763982	2.870478	-0.826518
S	3.490435	-0.700841	-0.818800
O	2.587614	-1.775824	-1.216015
O	4.029829	0.077915	-1.914685
O	4.459386	-1.077990	0.187058
H	2.560850	1.824196	-2.048792
H	3.778888	1.794394	0.268185
H	1.076378	1.419942	1.402243
H	2.140996	4.197406	0.854784
H	0.326376	4.405256	-0.646633
H	2.244274	2.229511	3.181843
H	0.755862	3.805790	1.877678
H	2.745719	3.372468	-1.213319
O	-4.556647	-2.436469	-0.388921
H	-5.507737	-2.334653	-0.420698
H	-4.170555	-1.765819	-0.973039
O	-3.861897	1.665558	-0.747567
H	-4.551748	2.073752	-1.273670
H	-2.986794	2.262135	-0.754042
O	-3.449336	1.307180	1.914133
H	-3.934337	1.430605	1.085467
H	-2.633958	1.839761	1.766783

TS2 + 3 WAT
($-158.50\ cm^{-1}$)

N	0.584931	-1.480520	0.975731
H	1.301609	-0.750136	0.937641
H	0.351469	-1.595320	1.957909
N	-2.229460	-1.042676	1.521447
Pt	-0.973755	-0.739976	-0.055068
N	0.236514	-0.377559	-1.651598
H	-1.791293	-0.981339	2.433503
H	-2.647056	-1.975808	1.424631
H	-0.107803	-0.805835	-2.503913
H	1.216206	-0.651551	-1.543936
H	-3.005231	-0.355011	1.517370
H	0.196980	0.643133	-1.755982
C	1.157797	-2.727525	0.466940
C	2.478726	-3.040368	1.129824
H	0.429199	-3.526029	0.600486
H	1.329344	-2.602883	-0.599637
H	2.316165	-3.584900	2.062495
H	2.975765	-2.094397	1.387849
N	3.263399	-3.847651	0.233700
H	3.588934	-3.255619	-0.522507
H	4.074649	-4.226986	0.699833
O	-2.865183	-1.416766	-1.449859
H	-2.609282	-1.691217	-2.332717
H	-3.571104	-0.725589	-1.539282
C	1.705497	2.681442	-1.077880
C	2.166295	2.093376	0.229682
C	1.127104	2.315189	1.306795
C	0.222118	3.454370	0.919648
C	-0.493834	3.110487	-0.373793
C	-1.749254	2.298716	-0.125578
O	2.318440	0.685722	0.111319
O	1.798392	2.532421	2.508163
O	0.342502	2.425667	-1.267822
O	-2.503186	2.710427	0.757447
O	-1.977143	1.297645	-0.864494
S	3.673073	0.056881	-0.513735
O	3.117618	-1.127380	-1.161925
O	4.192864	1.061008	-1.419081
O	4.525564	-0.238977	0.617884
H	2.246141	2.222588	-1.902467
H	3.111490	2.543944	0.542128
H	0.521170	1.401087	1.365937
H	0.809192	4.368992	0.813785
H	-0.840995	4.037772	-0.847227
H	1.173085	2.533262	3.234552
H	-0.530159	3.631384	1.688001
H	1.906742	3.756867	-1.099694
O	-3.283806	-3.347106	0.347163
H	-4.157713	-3.735687	0.371715
H	-3.247733	-2.748602	-0.422403
O	-4.372177	0.634293	-1.337869
H	-4.837627	1.055908	-2.061158
H	-3.481121	1.069422	-1.209421
O	-4.277736	0.865394	1.371880
H	-4.669622	0.694138	0.504274
H	-3.671269	1.622364	1.202085

MS4 + 3 WAT

N	-1.292797	-1.523468	0.625491
H	-0.448658	-2.081967	0.776705
H	-1.788222	-1.392919	1.507139
N	-1.386168	1.188660	1.611512
Pt	-0.720700	0.267955	-0.087040
N	0.015238	-0.611508	-1.765236
H	-0.701238	1.120405	2.357218
H	-2.247053	0.738989	1.942078
H	-0.647396	-0.670807	-2.530048
H	0.372438	-1.546005	-1.560693
H	-1.580283	2.192053	1.466676
H	0.792361	-0.008783	-2.042647
C	-2.182504	-2.278647	-0.253782
C	-2.669728	-3.528885	0.425506
H	-3.021282	-1.641099	-0.533359
H	-1.630242	-2.539158	-1.158741
H	-3.271341	-3.238571	1.290929
H	-1.803211	-4.089978	0.804460
N	-3.494940	-4.281929	-0.481232
H	-2.940075	-4.696883	-1.217410
H	-3.968426	-5.035385	-0.004788
O	-4.185078	0.490129	-0.536870
H	-5.092032	0.657598	-0.795781
H	-3.661342	1.279647	-0.792673
C	3.276899	0.349790	-1.192151
C	3.167706	-0.146508	0.222396
C	2.816366	0.987069	1.169351
C	2.973752	2.322376	0.487385
C	2.159680	2.382540	-0.795212
C	0.735914	2.826610	-0.538596
O	2.103155	-1.074904	0.321690
O	3.639074	0.876112	2.290720
O	2.182354	1.171351	-1.493151
O	0.582616	3.929933	-0.038944
O	-0.240482	2.084964	-0.908958
S	2.327390	-2.622010	-0.015899
O	0.941778	-3.020361	-0.266121
O	3.173472	-2.665862	-1.194535
O	2.915434	-3.224696	1.162578
H	3.252042	-0.492789	-1.880039
H	4.106150	-0.605345	0.546074
H	1.768259	0.836867	1.451722
H	4.031698	2.483375	0.270478
H	2.576295	3.172384	-1.431652
H	3.349831	1.489311	2.968464
H	2.653113	3.134158	1.141159
H	4.220556	0.884324	-1.337937
O	-3.605064	-0.493011	1.816982
H	-4.336453	-0.551165	2.431028
H	-3.944795	-0.133191	0.963691
O	-2.786577	2.683499	-1.219098
H	-3.051952	3.086368	-2.047537
H	-1.816956	2.520960	-1.253161
O	-1.883277	3.929872	1.029933
H	-2.453966	3.720748	0.276283
H	-0.998559	4.073954	0.638951

MS3' + 3 WAT

Pt	1.421851	0.291969	0.125379
N	0.697599	1.998817	-0.653232
N	2.777056	0.127490	-1.407957
C	1.492240	3.193728	-0.382530
H	-0.245913	2.163880	-0.252851
H	0.568129	1.898081	-1.656832
N	0.072777	0.354821	1.638347
H	2.933627	0.977211	-1.938226
H	2.448109	-0.599345	-2.050694
H	3.682376	-0.190706	-1.070562
C	0.861211	4.413748	-0.998212
H	2.505774	3.051462	-0.764442
H	1.565217	3.317568	0.697905
H	0.569152	0.523043	2.514418
H	-0.359809	-0.564123	1.711621
H	-0.686589	1.039546	1.495488
N	1.671084	5.569520	-0.722808
H	-0.125176	4.554534	-0.549777
H	0.702620	4.232045	-2.071326
H	2.523378	5.556994	-1.266043
H	1.182326	6.422141	-0.952313
C	-2.771957	2.461971	0.507003
C	-3.588049	1.210331	0.801215
O	-1.547902	2.438132	0.781435
O	-3.392545	3.404955	0.015933
C	-4.201365	0.667154	-0.478322
O	-2.766438	0.259308	1.422263
H	-4.387049	1.508491	1.489734
C	-3.185357	-0.196685	-1.176870
H	-4.493940	1.509965	-1.104755
H	-5.100544	0.079763	-0.275847
C	-3.104018	-1.061899	1.142090
C	-2.861942	-1.387424	-0.310420
O	-3.625127	-0.676788	-2.409681
H	-2.255476	0.373759	-1.298979
H	-2.498283	-1.698157	1.787782
H	-4.153930	-1.260747	1.383680
O	-1.499106	-1.697318	-0.589031
H	-3.477612	-2.237260	-0.611581
H	-3.706440	0.049584	-3.029376
S	-0.865408	-3.026220	0.005761
O	-0.165451	-2.601331	1.228953
O	0.064540	-3.453123	-1.028654
O	-1.954657	-3.935207	0.265806
O	2.104485	-1.431946	1.020059
H	2.837223	-1.883094	0.501543
H	1.315015	-2.033236	1.095399
O	3.865761	-2.345500	-0.527873
H	3.329663	-2.595180	-1.316397
H	4.466701	-3.059878	-0.312241
O	2.254852	-0.096738	3.400142
H	2.401518	-0.712386	2.666077
H	2.410827	-0.578224	4.212015
O	2.067277	-2.453750	-2.372813
H	2.106708	-2.851477	-3.243660
H	1.264833	-2.793929	-1.918006

TS2' + 3 WAT
(-172.43 cm⁻¹)

Pt	1.233277	0.017063	-0.227840
N	1.145350	1.914621	-0.869604
N	2.493440	-0.575462	-1.732866
C	2.001721	2.860894	-0.157542
H	0.154624	2.204011	-0.746860
H	1.336117	1.969401	-1.865846
N	0.089158	0.608548	1.338150
H	2.772740	0.150714	-2.382393
H	2.105645	-1.343873	-2.279029
H	3.340440	-0.952774	-1.305069
C	1.710470	4.275634	-0.581296
H	3.045036	2.603678	-0.350940
H	1.835800	2.744558	0.913903
H	0.706988	1.056131	2.019289
H	-0.324491	-0.199258	1.799281
H	-0.674701	1.241841	1.049236
N	2.579809	5.177682	0.125543
H	0.673780	4.508993	-0.326239
H	1.794400	4.348929	-1.675260
H	3.530678	5.105597	-0.209695
H	2.291092	6.136773	0.001095
C	-2.600483	2.493253	-0.326840
C	-3.541752	1.666134	0.547175
O	-1.381492	2.473738	-0.038432
O	-3.129775	3.139954	-1.232350
C	-4.468988	0.819130	-0.296049
O	-2.795641	0.894235	1.452861
H	-4.140200	2.389540	1.112564
C	-3.716543	-0.356568	-0.858408
H	-4.854346	1.446470	-1.099590
H	-5.319165	0.462620	0.289694
C	-3.174363	-0.441197	1.572033
C	-3.062932	-1.156337	0.254335
O	-4.536258	-1.225089	-1.580017
H	-2.903688	0.016120	-1.493816
H	-2.511825	-0.896949	2.307152
H	-4.200267	-0.540318	1.943231
O	-1.697823	-1.289655	-0.133465
H	-3.522008	-2.147207	0.306524
H	-4.909639	-0.763393	-2.332672
S	-0.799740	-2.467094	0.417993
O	-0.563683	-2.208399	1.835219
O	0.436262	-2.231062	-0.352166
O	-1.460652	-3.710877	0.122644
O	2.187564	-1.438511	1.571995
H	2.789003	-2.001507	1.032773
H	1.420746	-1.960507	1.844992
O	3.746356	-2.607762	-0.166528
H	3.246061	-3.125975	-0.835626
H	4.548625	-3.082423	0.053310
O	2.495206	0.940923	2.758701
H	2.533889	0.027676	2.416690
H	2.756590	0.913236	3.678555
O	2.055509	-3.426453	-1.968847
H	1.945110	-4.251883	-2.441466
H	1.251567	-3.249339	-1.446079

MS4' + 3 WAT

Pt	0.949973	-0.054450	-0.569860
N	1.382897	1.884291	-0.830078
N	1.695918	-0.573362	-2.410914
C	2.533153	2.400322	-0.090181
H	0.513327	2.375000	-0.544328
H	1.502471	2.094854	-1.817180
N	0.253606	0.501187	1.257080
H	1.002850	-0.453941	-3.142630
H	1.979578	-1.558667	-2.410698
H	2.509892	-0.031851	-2.680979
C	2.620089	3.899081	-0.198367
H	3.436410	1.934475	-0.489599
H	2.454268	2.097683	0.954566
H	1.029782	0.797548	1.858295
H	-0.211393	-0.256334	1.749092
H	-0.432809	1.262970	1.130412
N	3.782943	4.363573	0.509259
H	1.730873	4.338546	0.260941
H	2.605179	4.186943	-1.259543
H	4.631219	4.107639	0.022627
H	3.780505	5.369058	0.597062
C	-2.266404	2.747526	-0.097145
C	-3.250505	1.958834	0.767470
O	-1.052798	2.668854	0.202696
O	-2.758499	3.433838	-0.995047
C	-4.312313	1.267314	-0.058757
O	-2.543874	1.056907	1.581281
H	-3.737567	2.702120	1.410103
C	-3.752670	0.025608	-0.699735
H	-4.648746	1.968486	-0.822028
H	-5.175233	0.999407	0.555226
C	-3.089553	-0.219444	1.687567
C	-3.144318	-0.894510	0.345586
O	-4.720176	-0.703760	-1.390703
H	-2.937148	0.313335	-1.374299
H	-2.460189	-0.781349	2.375437
H	-4.101514	-0.196629	2.106320
O	-1.828711	-1.166456	-0.147217
H	-3.715516	-1.824944	0.392485
H	-5.079047	-0.170169	-2.101747
S	-0.938121	-2.357215	0.353804
O	-0.730428	-2.195851	1.784550
O	0.321710	-2.045172	-0.392945
O	-1.514751	-3.602720	-0.059042
O	2.034556	-1.975308	2.408351
H	2.555473	-2.289924	1.636308
H	1.100578	-2.139932	2.223541
O	3.595444	-2.707810	0.416101
H	3.171968	-3.007405	-0.412866
H	4.257268	-3.358469	0.651740
O	2.613591	0.546783	2.763279
H	2.445891	-0.425512	2.714680
H	2.747937	0.773296	3.682940
O	2.163429	-3.293418	-1.741221
H	2.189107	-4.119372	-2.226741
H	1.298274	-3.209619	-1.307645

GlcNS(6S)-model (Via aquation)

MS5 + 3 WAT

N	0.232921	-2.266918	-0.646136
H	-0.061338	-2.490786	-1.592808
H	1.078721	-1.685662	-0.746510
N	0.304181	-0.473203	1.553161
Pt	-1.151983	-1.154205	0.288939
N	-2.612099	-1.868020	-0.942010
H	1.224681	-0.576510	1.114407
H	0.308663	-1.008312	2.416322
H	-3.373101	-2.314270	-0.407426
H	-2.288500	-2.561601	-1.607553
H	0.218212	0.518230	1.814952
H	-3.039933	-1.111040	-1.489249
C	0.611994	-3.498842	0.045537
C	1.757873	-4.189481	-0.646120
H	0.899961	-3.248705	1.066922
H	-0.260761	-4.151554	0.101267
H	2.641525	-3.547677	-0.589514
H	1.511054	-4.319887	-1.709845
N	2.044105	-5.424386	0.034309
H	1.336022	-6.120805	-0.153302
H	2.929327	-5.808382	-0.261034
C	1.792700	3.141191	-1.205851
C	0.721309	2.787244	-0.204666
C	1.271166	2.905518	1.196987
C	2.578853	2.174842	1.327056
C	3.526525	2.486694	0.196168
C	4.741481	1.602558	0.215497
N	-0.410899	3.673735	-0.370079
O	0.380297	2.360741	2.131830
O	2.902178	2.327701	-1.046934
O	4.483181	0.269598	0.612634
S	-1.913227	3.045945	-0.413046
O	-1.963208	1.979900	-1.417041
O	-2.073005	2.454503	0.943684
O	-2.790604	4.161510	-0.670065
S	3.876703	-0.791986	-0.415286
O	4.337737	-2.056852	0.116675
O	2.417782	-0.642729	-0.299541
O	4.384459	-0.440974	-1.723854
H	1.419949	3.000567	-2.220519
H	0.415336	1.748666	-0.386431
H	1.414624	3.975285	1.399326
H	2.388261	1.103021	1.313806
H	3.881688	3.525625	0.273850
H	5.208650	1.604822	-0.770209
H	5.451158	1.969344	0.954678
H	-0.307199	4.417257	-1.049095
H	-0.522985	2.649420	1.925719
H	2.073811	4.199659	-1.085588
H	3.045780	2.415588	2.284641
O	-2.551453	0.032929	1.248173
H	-2.352548	1.025978	1.115787
H	-2.576043	-0.146284	2.194108
O	-4.912609	-2.710149	0.452787
H	-5.213771	-1.777804	0.366406
H	-5.569372	-3.274215	0.045636
O	-5.149346	-0.118075	0.238968
H	-4.334103	0.022815	0.740522
H	-4.903527	0.134304	-0.671774
O	-3.917685	0.407963	-2.061983
H	-3.232686	1.092736	-1.886520
H	-4.273202	0.549502	-2.939954

TS3 + 3 WAT
(-141.16 cm^{-1})

N	-0.376935	2.330674	-0.549617
H	-0.093977	2.733311	-1.438230
H	-1.228095	1.777625	-0.732759
N	-0.368861	0.425825	1.508303
Pt	0.966914	0.979172	0.066004
N	2.298313	1.501679	-1.386693
H	-1.318071	0.507333	1.131066
H	-0.297604	1.014627	2.331904
H	2.944792	2.245643	-1.082748
H	1.868881	1.798212	-2.256458
H	-0.257427	-0.547401	1.823405
H	2.863197	0.676600	-1.607699
C	-0.719984	3.413536	0.370535
C	-1.928497	4.168330	-0.119006
H	-0.933916	2.992901	1.352140
H	0.144732	4.070090	0.473745
H	-2.783900	3.485499	-0.136053
H	-1.753625	4.498017	-1.153349
N	-2.215281	5.246157	0.789208
H	-1.543529	5.995370	0.694510
H	-3.128806	5.637968	0.614813
C	-1.396608	-3.106493	-1.326212
C	-0.445642	-2.639025	-0.251559
C	-1.042208	-2.956398	1.099422
C	-2.437156	-2.399455	1.185147
C	-3.282381	-2.722642	-0.020963
C	-4.568285	-1.943058	-0.027308
N	0.842656	-3.281703	-0.434701
O	-0.309564	-2.376955	2.144017
O	-2.605480	-2.438864	-1.213365
O	-4.446428	-0.620612	0.460680
S	2.183961	-2.430939	-0.045445
O	2.020015	-1.042471	-0.491181
O	2.276424	-2.467388	1.424666
O	3.276814	-3.088596	-0.741593
S	-3.928278	0.564340	-0.479033
O	-4.518021	1.738982	0.128073
O	-2.464504	0.549106	-0.344680
O	-4.383296	0.255950	-1.818244
H	-0.991595	-2.883573	-2.313916
H	-0.348243	-1.551738	-0.359862
H	-1.052477	-4.048592	1.209734
H	-2.369656	-1.314800	1.264016
H	-3.549617	-3.790104	-0.021957
H	-4.977249	-1.922054	-1.038318
H	-5.284893	-2.412743	0.644001
H	0.977812	-3.714048	-1.341145
H	0.625235	-2.635506	2.086865
H	-1.544965	-4.194503	-1.250108
H	-2.925677	-2.764041	2.091184
O	2.602917	0.165692	1.769086
H	2.518887	-0.806241	1.859656
H	2.526108	0.560419	2.640575
O	4.278900	3.121968	-0.265367
H	4.656214	2.249282	0.005489
H	4.951192	3.597918	-0.751744
O	4.867433	0.685457	0.397530
H	4.095327	0.467040	0.951067
H	4.801040	0.121574	-0.397610
O	4.441699	-0.764877	-1.822779
H	4.101033	-1.641602	-1.576784
H	5.168689	-0.898651	-2.432710

MS6 + 3 WAT

N	-0.291807	2.236248	-0.508667
H	-0.235324	2.556364	-1.471822
H	-1.253472	1.886304	-0.377380
N	-0.057857	0.279175	1.528550
Pt	0.906404	0.654694	-0.240770
N	1.977855	1.097124	-1.911480
H	-0.994068	0.682520	1.537503
H	0.511061	0.663043	2.280224
H	2.400948	2.021905	-1.761852
H	1.456033	1.092159	-2.780214
H	-0.178493	-0.715909	1.755782
H	2.751811	0.430108	-1.996743
C	-0.036372	3.367490	0.381955
C	-1.013709	4.485716	0.138860
H	-0.125273	3.026126	1.414233
H	0.995312	3.691587	0.223428
H	-2.026259	4.125398	0.340889
H	-0.981560	4.767521	-0.923529
N	-0.724464	5.573887	1.033656
H	0.137555	6.035651	0.777595
H	-1.454971	6.270044	1.011865
C	-1.333864	-2.776970	-1.545934
C	-0.457844	-2.476184	-0.354191
C	-1.142494	-2.971229	0.897587
C	-2.527191	-2.386361	0.961330
C	-3.294593	-2.526495	-0.327112
C	-4.577154	-1.738287	-0.306768
N	0.855509	-3.059179	-0.554489
O	-0.488919	-2.541907	2.059283
O	-2.531764	-2.090100	-1.419875
O	-4.527942	-0.564912	0.479569
S	2.178947	-2.395349	0.123902
O	2.199493	-0.921872	-0.068487
O	2.117203	-2.672362	1.559008
O	3.305483	-2.968542	-0.583012
S	-3.974098	0.818074	-0.103144
O	-4.722431	1.801653	0.650158
O	-2.542955	0.828638	0.225751
O	-4.231656	0.795081	-1.528874
H	-0.855523	-2.434133	-2.464329
H	-0.388289	-1.383275	-0.310954
H	-1.173240	-4.067895	0.861810
H	-2.428832	-1.321311	1.180996
H	-3.560321	-3.579976	-0.499328
H	-4.863608	-1.484219	-1.328326
H	-5.364485	-2.333861	0.151666
H	1.070481	-3.311424	-1.512310
H	0.427896	-2.857766	2.071891
H	-1.512474	-3.859286	-1.629368
H	-3.083616	-2.844136	1.781748
O	2.586623	-0.115852	2.620814
H	2.488316	-1.053858	2.388367
H	2.969231	-0.085212	3.498849
O	3.141303	3.191680	-0.515869
H	3.547145	2.454895	0.003954
H	3.828995	3.834648	-0.687019
O	4.142201	1.077533	0.672165
H	3.564851	0.652764	1.333594
H	4.251359	0.439270	-0.054643
O	4.393040	-0.549289	-1.550572
H	4.153243	-1.460397	-1.315503
H	5.215751	-0.582219	-2.040961

MS5’ + 3 WAT

N	0.739859	-1.915979	-0.939344
H	1.555933	-1.896323	-0.311673
H	0.679590	-2.874326	-1.275785
N	0.135750	-0.868638	1.709328
Pt	-0.941858	-1.465329	0.077802
N	-2.098634	-1.950057	-1.529144
H	1.110754	-1.181352	1.708185
H	-0.312776	-1.140328	2.583609
H	-1.640646	-2.457877	-2.276778
H	-2.456891	-1.067166	-1.905572
H	0.134740	0.149105	1.697006
H	-2.901307	-2.508287	-1.211893
C	1.006885	-1.015481	-2.061331
C	2.263859	-1.372883	-2.806641
H	0.150153	-1.004775	-2.735559
H	1.110222	-0.009426	-1.655631
H	2.117654	-2.311751	-3.347452
H	3.072253	-1.523809	-2.079194
N	2.535972	-0.317141	-3.748954
H	2.811990	0.519886	-3.244925
H	3.289054	-0.570062	-4.371634
C	1.672626	2.111787	1.772262
C	2.447804	1.353092	0.727628
C	2.670513	2.256905	-0.459821
C	1.325942	2.748351	-0.939371
C	0.422924	3.263801	0.156698
C	-0.992613	3.397395	-0.313776
N	3.668883	0.824670	1.281845
O	3.277166	1.592107	-1.524509
O	0.401127	2.391927	1.268690
O	-1.373399	2.152073	-0.870977
S	4.048828	-0.727218	0.859455
O	2.814313	-1.533060	0.959519
O	4.480813	-0.625030	-0.534788
O	5.072461	-1.122884	1.801349
S	-2.922952	1.827123	-0.990154
O	-3.579786	3.051287	-1.373762
O	-2.962356	0.743938	-1.948080
O	-3.320117	1.377677	0.364620
H	1.543682	1.516317	2.678151
H	1.811688	0.526044	0.389913
H	3.297520	3.096502	-0.128671
H	0.827623	1.912937	-1.434609
H	0.756670	4.249192	0.504257
H	-1.642536	3.658565	0.524296
H	-1.072005	4.174399	-1.074390
H	3.771555	0.940075	2.282438
H	3.886778	0.903738	-1.201589
H	2.190102	3.038855	2.049059
H	1.460454	3.532249	-1.687511
O	-2.693206	-1.018979	1.070370
H	-2.540162	-0.923147	2.059472
H	-3.013167	-0.129617	0.751543
O	-4.101851	-3.081408	0.053345
H	-5.054760	-3.036584	-0.023159
H	-3.817317	-2.346842	0.619811
O	-1.911173	1.919197	2.611883
H	-2.498262	1.857464	1.837379
H	-1.045218	2.185841	2.259357
O	-2.029036	-0.501712	3.449274
H	-2.533927	-0.694724	4.240542
H	-1.978599	0.483578	3.318180

TS3’ + 3 WAT

(-159.74 cm⁻¹)

N	0.641309	-2.423149	0.133087
H	1.265293	-2.068478	0.864823
H	0.408289	-3.377256	0.397554
N	-0.156039	0.067299	1.249032
Pt	-1.041432	-1.335472	0.067917
N	-1.979385	-2.728045	-1.086793
H	0.831125	-0.131863	1.432065
H	-0.634051	0.117138	2.153913
H	-1.456715	-3.568808	-1.305148
H	-2.237445	-2.290187	-1.966423
H	-0.214238	0.992374	0.818277
H	-2.849670	-3.004251	-0.603461
C	1.436753	-2.420990	-1.096933
C	2.625286	-3.338767	-0.972089
H	0.809282	-2.704274	-1.941601
H	1.789330	-1.401871	-1.257047
H	2.295332	-4.379546	-1.024981
H	3.073620	-3.190057	0.020837
N	3.536449	-3.068475	-2.050310
H	4.019384	-2.200360	-1.848997
H	4.228378	-3.798433	-2.134538
C	1.680155	2.755733	1.186997
C	2.478858	1.850529	0.285951
C	2.705849	2.543553	-1.030912
C	1.337504	2.814658	-1.610164
C	0.381532	3.501082	-0.658719
C	-1.037263	3.329797	-1.114379
N	3.689484	1.382465	0.913972
O	3.436132	1.767249	-1.924818
O	0.407994	2.940654	0.638254
O	-1.272688	1.932413	-1.064119
S	3.843540	-0.260433	0.890071
O	2.600808	-0.839497	1.437875
O	3.960470	-0.557508	-0.542078
O	5.013697	-0.551243	1.687521
S	-2.757318	1.397745	-1.213098
O	-3.318135	1.988576	-2.398427
O	-2.545466	-0.046323	-1.267058
O	-3.438798	1.785975	0.030161
H	1.536169	2.301546	2.168746
H	1.849098	0.982459	0.063110
H	3.245860	3.481696	-0.844671
H	0.921425	1.845777	-1.900389
H	0.607610	4.570668	-0.585237
H	-1.727406	3.849078	-0.445452
H	-1.173920	3.694078	-2.132958
H	3.880756	1.752280	1.837165
H	3.721245	0.937670	-1.505492
H	2.185809	3.719566	1.322622
H	1.421894	3.419768	-2.514913
O	-3.115123	-0.633850	1.318973
H	-2.822637	-0.342729	2.209650
H	-3.461714	0.164638	0.886985
O	-4.128246	-2.954630	0.627920
H	-5.064859	-2.962422	0.433227
H	-3.902052	-2.070431	0.984263
O	-1.957461	2.779388	2.069776
H	-2.543992	2.528758	1.333521
H	-1.091868	2.975976	1.677911
O	-1.984453	0.549817	3.368145
H	-2.109672	0.526153	4.317021
H	-1.992151	1.488710	3.052120

MS6’ + 3 WAT

N	-0.723811	2.432092	0.334466
H	-1.249089	1.998897	1.097432
H	-0.444470	3.352304	0.665683
N	0.195185	-0.120584	1.106376
Pt	0.885393	1.334222	-0.138886
N	1.724847	2.828405	-1.242316
H	-0.790433	0.008590	1.351097
H	0.736427	-0.103962	1.979474
H	1.202093	3.691897	-1.332122
H	1.947552	2.509525	-2.179232
H	0.294202	-1.052497	0.702189
H	2.607301	3.024201	-0.739947
C	-1.670654	2.557271	-0.777564
C	-2.877163	3.371261	-0.389746
H	-1.165170	2.993908	-1.638725
H	-1.995354	1.551247	-1.042294
H	-2.606958	4.427552	-0.312983
H	-3.205990	3.046747	0.608068
N	-3.883949	3.206116	-1.403487
H	-4.266887	2.270033	-1.326488
H	-4.637478	3.866313	-1.280772
C	-1.526776	-2.927432	1.039996
C	-2.354862	-2.015090	0.174795
C	-2.511751	-2.632791	-1.188140
C	-1.115658	-2.788066	-1.746901
C	-0.140399	-3.477021	-0.815415
C	1.276966	-3.184640	-1.212739
N	-3.601013	-1.649813	0.800236
O	-3.261952	-1.836665	-2.047894
O	-0.233747	-3.009864	0.514226
O	1.414457	-1.777075	-1.058807
S	-3.829839	-0.016631	0.887343
O	-2.614748	0.582686	1.469794
O	-3.968183	0.368537	-0.522832
O	-5.011640	0.162980	1.701402
S	2.849904	-1.129452	-1.027522
O	3.523901	-1.437398	-2.254947
O	2.520554	0.302911	-0.892292
O	3.509791	-1.637343	0.173337
H	-1.428888	-2.523567	2.048873
H	-1.771608	-1.102233	0.020248
H	-3.002331	-3.609480	-1.080276
H	-0.753699	-1.779511	-1.966776
H	-0.295345	-4.561511	-0.822613
H	1.981432	-3.698492	-0.555573
H	1.467797	-3.462387	-2.249270
H	-3.796348	-2.091404	1.690309
H	-3.608188	-1.057491	-1.580727
H	-1.980128	-3.923713	1.107417
H	-1.143413	-3.338149	-2.689442
O	3.948660	0.582408	1.842341
H	3.256010	0.333685	2.483207
H	3.971874	-0.158030	1.220929
O	3.805084	2.886728	0.558049
H	4.698501	3.227864	0.537805
H	3.832673	2.020942	1.030021
O	2.005513	-2.780099	2.121076
H	2.625238	-2.496202	1.428173
H	1.182168	-3.013464	1.664150
O	1.952161	-0.474957	3.305477
H	1.823020	-0.413012	4.252341
H	1.957957	-1.427882	3.041734

Direct Substitution

IDOA(2S)-model (Direct substitution)

Msa + 3 WAT

N	-0.636720	-1.015747	-1.305931
H	-1.306295	-0.371142	-0.874601
H	-0.462104	-0.653559	-2.239017
N	2.105979	-0.123535	-1.666505
Pt	1.016326	-0.924834	-0.141217
N	-0.072520	-1.608348	1.450785
H	1.749921	-0.333754	-2.592358
H	3.078101	-0.452768	-1.647377
H	0.263073	-2.541024	1.713653
H	-1.088704	-1.625547	1.318740
H	2.089500	0.904185	-1.549378
H	0.104575	-1.002970	2.246261
C	-1.287482	-2.321688	-1.412796
C	-2.683696	-2.197643	-1.981167
H	-0.657870	-2.987260	-2.001904
H	-1.367584	-2.742325	-0.412798
H	-2.659644	-2.256964	-3.071291
H	-3.079136	-1.205062	-1.721934
N	-3.493520	-3.267393	-1.461089
H	-3.687529	-3.073946	-0.484489
H	-4.377871	-3.324454	-1.944937
C	-1.695739	2.785319	1.323266
C	-2.231373	1.990394	0.157357
C	-1.370968	2.224737	-1.052553
C	-0.829850	3.625036	-1.013108
C	0.120867	3.719668	0.160629
C	1.519219	3.201548	-0.162982
O	-2.169713	0.594531	0.414160
O	-2.129209	1.922369	-2.183470
O	-0.310071	2.912103	1.239134
O	1.690713	2.599576	-1.236694
O	2.381632	3.407041	0.710453
S	-3.460651	-0.150175	1.026456
O	-2.959821	-1.521250	1.056504
O	-3.678705	0.446723	2.328687
O	-4.534923	0.069593	0.079693
H	-1.928812	2.268829	2.253542
H	-3.265821	2.263350	-0.071156
H	-0.507101	1.553766	-0.976319
H	-1.647220	4.346393	-0.932420
H	0.208581	4.756236	0.505470
H	-1.587586	2.019089	-2.968651
H	-0.276643	3.836928	-1.928183
H	-2.172908	3.771777	1.349458
Cl	2.905221	-0.939573	1.253061
O	1.678955	-3.756388	1.917585
H	2.351783	-3.068400	1.833014
H	1.877801	-4.246127	2.716035
O	4.711987	-1.196596	-1.239393
H	4.434305	-1.402327	-0.337706
H	5.014580	-2.014439	-1.635313
O	4.474381	1.847218	0.667948
H	4.101209	0.978383	0.839951
H	3.697434	2.457337	0.633772

TSa + 3 WAT
(-140.06 cm⁻¹)

N	-0.413172	-1.134437	-1.332254
H	-1.229012	-0.564080	-1.094692
H	-0.233388	-0.976888	-2.319336
N	2.227246	0.100066	-1.808849
Pt	1.099787	-0.415362	-0.187748
N	-0.064648	-0.646676	1.465976
H	1.799018	-0.078532	-2.710179
H	3.144875	-0.362351	-1.816596
H	0.386996	-1.180358	2.217050
H	-0.989085	-1.049061	1.304001
H	2.350293	1.117385	-1.729046
H	-0.200808	0.320883	1.771233
C	-0.765807	-2.538315	-1.124782
C	-2.068325	-2.891387	-1.804200
H	0.058505	-3.162756	-1.466823
H	-0.884475	-2.696676	-0.055044
H	-1.892672	-3.159450	-2.848410
H	-2.716979	-2.003175	-1.803382
N	-2.661355	-4.007111	-1.115955
H	-3.016931	-3.674938	-0.226155
H	-3.438581	-4.386331	-1.636945
C	-2.031176	2.301650	1.414109
C	-2.541054	1.804768	0.087684
C	-1.691129	2.342533	-1.042828
C	-0.924767	3.550216	-0.578885
C	-0.007666	3.143482	0.554432
C	1.274534	2.500674	0.047552
O	-2.436348	0.389428	0.026079
O	-2.531662	2.598785	-2.124856
O	-0.635201	2.249114	1.444469
O	1.726759	2.867182	-1.040088
O	1.819860	1.648292	0.800459
S	-3.623061	-0.538969	0.611696
O	-2.864778	-1.748102	0.910953
O	-4.133885	0.162755	1.772516
O	-4.576479	-0.688704	-0.468100
H	-2.404168	1.662444	2.211829
H	-3.579954	2.107145	-0.067374
H	-0.960186	1.563980	-1.300295
H	-1.617983	4.336461	-0.272638
H	0.301047	4.038400	1.110436
H	-2.008223	2.836913	-2.891694
H	-0.303734	3.940133	-1.384833
H	-2.393116	3.318458	1.598107
Cl	3.249040	-1.203529	1.156306
O	1.409700	-2.208511	3.341113
H	2.190642	-1.991061	2.805414
H	1.588166	-1.908723	4.232723
O	4.627034	-1.397870	-1.535853
H	4.333958	-1.590488	-0.629993
H	4.721645	-2.236792	-1.987626
O	4.444178	1.563714	0.300300
H	4.589554	0.647229	0.549647
H	3.478906	1.660693	0.443756

MSb + 3 WAT

N	-1.097790	1.343563	-0.788504
H	-0.256559	1.922471	-0.857303
H	-1.514484	1.233311	-1.712104
N	-1.127445	-1.402833	-1.806567
Pt	-0.538167	-0.460968	-0.085749
N	-0.013741	0.410080	1.666689
H	-0.373210	-1.583940	-2.458895
H	-1.856081	-0.867359	-2.286872
H	-0.834841	0.409420	2.282087
H	0.329369	1.360167	1.540635
H	-1.548676	-2.296716	-1.531525
H	0.735842	-0.162040	2.052835
C	-2.067152	2.072090	0.025214
C	-2.436364	3.375360	-0.626985
H	-2.949163	1.447744	0.164712
H	-1.631255	2.256452	1.008025
H	-2.928008	3.160955	-1.579958
H	-1.514362	3.930418	-0.851344
N	-3.355574	4.095182	0.215188
H	-2.893334	4.430882	1.049399
H	-3.731522	4.901971	-0.261016
C	3.301676	-0.299200	1.248323
C	3.211931	0.224312	-0.159872
C	2.880228	-0.895401	-1.125420
C	3.166722	-2.236520	-0.502084
C	2.370625	-2.405374	0.785177
C	1.002596	-3.002877	0.520358
O	2.146831	1.149468	-0.278997
O	3.612184	-0.684229	-2.293948
O	2.262827	-1.204181	1.491583
O	0.975715	-4.181888	0.219562
O	-0.059398	-2.301445	0.665325
S	2.324920	2.682637	0.137859
O	0.918808	3.070454	0.221137
O	3.014027	2.686614	1.416517
O	3.063181	3.322387	-0.932819
H	3.196095	0.522159	1.953794
H	4.153755	0.693314	-0.460516
H	1.803642	-0.812851	-1.322836
H	4.237472	-2.316307	-0.301531
H	2.875747	-3.147551	1.412273
H	3.305294	-1.277622	-2.981174
H	2.909612	-3.045011	-1.188008
H	4.275928	-0.770513	1.414230
Cl	-4.308964	-0.939527	0.340963
O	-2.635100	0.252262	2.683400
H	-3.139880	-0.134562	1.944175
H	-2.956345	-0.169884	3.480231
O	-3.277635	0.347744	-2.243012
H	-3.726541	-0.027111	-1.458500
H	-3.940070	0.466282	-2.924002
O	-2.378554	-3.381890	-0.171884
H	-3.012248	-2.684850	0.063336
H	-1.548267	-3.152582	0.285313

MSa' + 3 WAT

N	0.191705	1.050427	-0.983980
H	0.956714	1.234107	-0.307932
H	0.461363	0.219635	-1.513394
N	-2.290400	0.003763	-1.830253
Pt	-1.518801	0.582970	-0.026657
N	-0.663736	1.011324	1.771926
H	-2.721438	0.748613	-2.365461
H	-2.975461	-0.746866	-1.687317
H	-1.269782	1.600743	2.348792
H	0.258364	1.453279	1.678507
H	-1.542875	-0.420702	-2.378692
H	-0.554363	0.107166	2.236810
C	0.103428	2.201168	-1.871704
C	1.410695	2.470369	-2.568390
H	-0.678429	2.039693	-2.616076
H	-0.189110	3.067577	-1.274509
H	1.677078	1.598327	-3.172828
H	2.198477	2.578192	-1.813907
N	1.259852	3.607603	-3.440617
H	1.151150	4.458764	-2.905582
H	2.075325	3.728691	-4.023359
C	3.102243	-0.864657	-1.036291
C	2.551021	-1.746902	0.051903
C	2.967314	-1.243532	1.414906
C	4.164335	-0.340590	1.280692
C	3.786056	0.855062	0.425420
C	3.010775	1.861487	1.266865
O	1.128774	-1.745312	0.066800
O	3.206995	-2.354678	2.223816
O	3.011616	0.478383	-0.681240
O	3.648445	2.407906	2.169581
O	1.802043	2.040405	0.993435
S	0.328986	-2.565850	-1.039822
O	-0.896488	-2.897201	-0.310727
O	0.103764	-1.636697	-2.143192
O	1.144896	-3.705590	-1.391809
H	2.532387	-0.993333	-1.957526
H	2.897735	-2.774015	-0.081226
H	2.124699	-0.660882	1.808224
H	4.996101	-0.898362	0.843389
H	4.695258	1.358055	0.079770
H	3.324205	-2.073152	3.132309
H	4.489449	0.022908	2.255885
H	4.137770	-1.153671	-1.246799
Cl	-3.532196	0.030452	1.077543
O	-2.860467	2.399471	2.960038
H	-3.375493	1.715342	2.510650
H	-3.140731	2.404612	3.875476
O	-3.476390	-2.408768	-0.937908
H	-2.551536	-2.647778	-0.746876
H	-3.786522	-1.951274	-0.149176
O	-1.186319	-1.845515	2.288064
H	-2.068617	-1.480779	2.157480
H	-0.943788	-2.196158	1.416751

TSa' + 3 WAT
(-125.82 cm⁻¹)

N	-0.305119	1.742486	-0.834483
H	0.586138	1.872660	-0.309959
H	-0.019142	1.636148	-1.803490
N	-1.655672	-0.575300	-2.014011
Pt	-1.100719	0.033613	-0.144198
N	-0.455623	0.609588	1.698833
H	-1.634246	0.147177	-2.724498
H	-2.605030	-0.973973	-2.012538
H	-1.137950	1.259449	2.099106
H	0.453007	1.080536	1.642284
H	-0.998218	-1.309340	-2.288369
H	-0.399554	-0.204606	2.315997
C	-1.137646	2.929776	-0.683327
C	-0.379512	4.174113	-1.063460
H	-2.032639	2.828629	-1.298873
H	-1.468114	2.981582	0.357389
H	-0.110900	4.123424	-2.122320
H	0.558786	4.197078	-0.492646
N	-1.217519	5.327492	-0.859434
H	-1.370997	5.490724	0.126507
H	-0.788913	6.160787	-1.234891
C	2.519764	-0.436500	-1.634659
C	2.720533	-1.418754	-0.510303
C	3.656359	-0.855742	0.536777
C	4.428154	0.305553	-0.027758
C	3.454405	1.386024	-0.452902
C	2.952160	2.164860	0.759548
O	1.501147	-1.679027	0.171944
O	4.474182	-1.894885	0.981635
O	2.359423	0.861304	-1.154676
O	3.822021	2.670556	1.471939
O	1.713423	2.245274	0.929809
S	0.483223	-2.758336	-0.393079
O	-0.758478	-2.338686	0.284056
O	0.418488	-2.583218	-1.835415
O	0.954736	-4.056001	0.030628
H	1.621066	-0.692964	-2.191009
H	3.137272	-2.354880	-0.893549
H	3.024371	-0.492688	1.357417
H	5.042297	-0.032330	-0.865908
H	3.976918	2.109168	-1.089785
H	4.987391	-1.600853	1.735834
H	5.093461	0.730041	0.723951
H	3.371586	-0.503107	-2.321020
Cl	-3.387678	-0.833730	1.177327
O	-2.855153	2.065660	2.101990
H	-3.221593	1.212555	1.811821
H	-3.335489	2.319565	2.890397
O	-4.259891	-1.556704	-1.632838
H	-4.473619	-2.479663	-1.771668
H	-4.150153	-1.430298	-0.673458
O	-1.050301	-1.945104	2.928909
H	-1.961878	-1.650494	2.809720
H	-0.833348	-2.278577	2.042020

MSb' + 3 WAT

N	-0.728416	1.691173	-0.939993
H	0.144355	2.108604	-0.559873
H	-0.664981	1.771330	-1.950337
N	-1.198238	-0.988250	-2.179418
Pt	-0.640786	-0.228993	-0.368051
N	-0.034179	0.440874	1.449211
H	-1.070199	-0.372551	-2.974338
H	-2.195413	-1.254263	-2.138644
H	-0.839395	0.693781	2.037309
H	0.561381	1.271390	1.323389
H	-0.637991	-1.820467	-2.356301
H	0.507784	-0.279273	1.915240
C	-1.878231	2.452916	-0.463746
C	-1.763570	3.906362	-0.836331
H	-2.790975	2.023468	-0.879139
H	-1.932976	2.343934	0.622383
H	-1.770765	4.002427	-1.925551
H	-0.791719	4.279975	-0.485008
N	-2.892361	4.625772	-0.306575
H	-2.845793	4.676801	0.702148
H	-2.916295	5.574015	-0.651796
C	2.579441	-0.222263	-1.431617
C	3.017711	-1.038643	-0.240420
C	3.715305	-0.148542	0.762888
C	4.281016	1.065580	0.074732
C	3.150207	1.871896	-0.540104
C	2.528239	2.834400	0.465478
O	1.938527	-1.619789	0.502616
O	4.682537	-0.914357	1.410097
O	2.142924	1.041478	-1.047466
O	3.307538	3.606189	1.026675
O	1.285408	2.781986	0.628104
S	0.984626	-2.746909	-0.061968
O	-0.365322	-2.169483	0.270944
O	1.185282	-2.839594	-1.489112
O	1.209891	-3.936516	0.708234
H	1.765176	-0.709461	-1.960907
H	3.694991	-1.835420	-0.555066
H	2.950202	0.179726	1.478206
H	5.004747	0.749783	-0.680543
H	3.553882	2.493536	-1.349177
H	5.038759	-0.425838	2.153760
H	4.806002	1.700748	0.788494
H	3.427985	-0.152181	-2.122313
Cl	-4.389009	-0.579915	1.111382
O	-2.455802	1.258645	2.599376
H	-3.119838	0.697772	2.153958
H	-2.663938	1.242529	3.533960
O	-3.897795	-1.461256	-1.773096
H	-4.328800	-2.305319	-1.908213
H	-4.075743	-1.197002	-0.849865
O	-2.205791	-2.508595	2.285360
H	-2.897776	-1.927212	1.935492
H	-1.499782	-2.487180	1.627010

GlcNS(6S)-model (Direct substitution)

MSc + 3 WAT

N	-0.034744	0.516530	-1.340888
H	0.203164	-0.401112	-1.729627
H	-0.934233	0.433191	-0.858371
N	0.033445	1.210866	1.467372
Pt	1.424214	0.937310	0.002798
N	2.774672	0.469472	-1.462651
H	-0.883936	1.456401	1.088160
H	0.307993	1.925358	2.149135
H	3.748137	0.682377	-1.218227
H	2.590250	0.942187	-2.340807
H	-0.061682	0.308498	1.945012
H	2.674373	-0.534514	-1.623761
C	-0.257972	1.495454	-2.399739
C	-1.413974	1.083604	-3.273229
H	-0.467879	2.459787	-1.938136
H	0.649687	1.602937	-2.997117
H	-2.317332	1.055105	-2.657546
H	-1.238622	0.060935	-3.638319
N	-1.594743	2.048769	-4.324406
H	-0.866554	1.973006	-5.021304
H	-2.476401	1.914700	-4.796907
C	-2.457084	-2.779978	-0.495175
C	-1.129986	-2.456407	0.143885
C	-1.298412	-2.258271	1.632955
C	-2.449723	-1.325318	1.907271
C	-3.687083	-1.657310	1.114788
C	-4.753167	-0.606648	1.248879
N	-0.194529	-3.514083	-0.167620
O	-0.161013	-1.681762	2.209708
O	-3.383892	-1.782798	-0.246817
O	-4.261013	0.713561	1.350697
S	1.338155	-3.090424	-0.582112
O	1.291651	-2.048950	-1.616189
O	1.893603	-2.549051	0.673546
O	1.954182	-4.323646	-1.024150
S	-3.790710	1.537693	0.061558
O	-4.058827	2.905834	0.450500
O	-2.353488	1.269106	-0.063846
O	-4.564222	1.042621	-1.057639
H	-2.338302	-2.858005	-1.576619
H	-0.778239	-1.511623	-0.278292
H	-1.490350	-3.245695	2.075305
H	-2.144856	-0.316885	1.628132
H	-4.122317	-2.607577	1.459756
H	-5.438034	-0.678211	0.402664
H	-5.304501	-0.758849	2.175055
H	-0.539634	-4.210233	-0.817575
H	0.644841	-2.051534	1.804900
H	-2.822627	-3.747913	-0.117932
H	-2.674649	-1.319658	2.976215
Cl	3.130858	1.430998	1.541570
O	5.305987	1.482576	-0.619409
H	5.686285	2.232072	-1.078663
H	4.880778	1.822350	0.177926
O	1.186578	3.227309	3.121181
H	2.042128	2.853425	2.873267
H	1.160803	3.261532	4.077753
O	4.324037	-1.391702	0.543222
H	4.192319	-0.510679	0.905407
H	3.454020	-1.821235	0.607537

TSb + 3 WAT
(-120.77 cm^{-1})

N	-0.445761	1.895551	-1.001510
H	-0.382558	2.036094	-2.005163
H	-1.306656	1.355989	-0.835295
N	0.013746	0.406145	1.345537
Pt	1.114642	0.811356	-0.322249
N	2.233276	1.145234	-1.991106
H	-0.988329	0.488765	1.159550
H	0.256176	1.072903	2.085262
H	2.871307	1.941731	-1.857299
H	1.718626	1.285010	-2.852900
H	0.173277	-0.554684	1.668848
H	2.792675	0.303497	-2.108925
C	-0.588113	3.191263	-0.337096
C	-1.894345	3.849877	-0.695034
H	-0.521538	3.041943	0.741652
H	0.256401	3.819912	-0.626915
H	-2.713583	3.240480	-0.304171
H	-2.000726	3.871631	-1.789476
N	-1.953521	5.151049	-0.084024
H	-1.321741	5.796599	-0.537767
H	-2.880945	5.543974	-0.148538
C	-1.554097	-2.775840	-1.560326
C	-0.434266	-2.432725	-0.603842
C	-0.808431	-2.933000	0.775822
C	-2.185481	-2.446878	1.142568
C	-3.205288	-2.671778	0.057553
C	-4.516825	-2.002815	0.356454
N	0.756748	-3.063077	-1.134487
O	0.053711	-2.464681	1.774376
O	-2.745868	-2.189200	-1.172508
O	-4.399092	-0.740699	0.983578
S	2.256781	-2.691827	-0.571826
O	2.412974	-1.234795	-0.456047
O	2.329623	-3.309265	0.766906
O	3.149207	-3.288268	-1.542631
S	-4.021505	0.561305	0.136558
O	-4.566883	1.634544	0.940121
O	-2.554020	0.582571	0.102814
O	-4.622321	0.393529	-1.170090
H	-1.321596	-2.406809	-2.560631
H	-0.328672	-1.339546	-0.573084
H	-0.785195	-4.031108	0.743713
H	-2.129118	-1.375848	1.338824
H	-3.411027	-3.748084	-0.050235
H	-5.090434	-1.898843	-0.565797
H	-5.080774	-2.603502	1.067750
H	0.797472	-3.038363	-2.147023
H	0.938160	-2.843710	1.621717
H	-1.660479	-3.870036	-1.615427
H	-2.505902	-2.936325	2.064752
Cl	3.278214	1.227555	1.217057
O	4.015963	3.103660	-1.073777
H	3.782620	4.026144	-0.966201
H	3.898834	2.676787	-0.208084
O	1.013771	2.646491	2.721778
H	1.867513	2.367527	2.349325
H	1.162396	2.840285	3.647588
O	3.400820	-1.581303	2.603059
H	3.363696	-0.730704	2.144589
H	3.100541	-2.231131	1.950986

MSd + 3 WAT

N	0.789029	1.894528	-0.685184
H	1.264303	2.243403	-1.516540
H	-0.224027	2.029409	-0.773146
N	0.238308	-0.041414	1.255171
Pt	1.044328	-0.099221	-0.628961
N	1.840500	-0.088583	-2.502419
H	-0.580751	0.568950	1.219591
H	0.927309	0.348768	1.915494
H	2.534566	0.663591	-2.585033
H	1.137380	0.037974	-3.222844
H	-0.075471	-0.934121	1.636869
H	2.292072	-0.980904	-2.677376
C	1.261784	2.702410	0.434108
C	0.975201	4.159195	0.191886
H	0.766990	2.387232	1.351174
H	2.333442	2.528925	0.548961
H	-0.107899	4.300632	0.128490
H	1.396859	4.447767	-0.782015
N	1.478847	4.935707	1.292849
H	2.489724	4.948596	1.294898
H	1.167544	5.894242	1.237442
C	-2.730016	-2.101424	-1.286275
C	-1.714605	-2.226780	-0.178461
C	-2.439901	-2.302240	1.143475
C	-3.336299	-1.103495	1.273538
C	-4.197175	-0.883561	0.055197
C	-4.909890	0.440648	0.107847
N	-0.856691	-3.373677	-0.386686
O	-1.563969	-2.278704	2.230651
O	-3.442915	-0.921628	-1.126675
O	-4.180292	1.467163	0.749323
S	0.743536	-3.278519	-0.098053
O	1.338545	-2.129045	-0.851208
O	0.870642	-3.002765	1.334557
O	1.291442	-4.520087	-0.573809
S	-3.096460	2.342553	-0.038388
O	-3.101727	3.587183	0.701973
O	-1.829959	1.615648	0.093063
O	-3.563801	2.432485	-1.407890
H	-2.231249	-2.055072	-2.254766
H	-1.129440	-1.303245	-0.219714
H	-3.026040	-3.232172	1.149075
H	-2.704303	-0.224266	1.411272
H	-4.967092	-1.666999	-0.007849
H	-5.173560	0.754142	-0.903304
H	-5.816138	0.343225	0.703031
H	-1.034737	-3.929663	-1.213785
H	-0.853644	-2.922153	2.105835
H	-3.408635	-2.968093	-1.284883
H	-3.966304	-1.200783	2.160482
Cl	4.504094	0.859349	0.578007
O	3.316163	2.170434	-1.901562
H	3.934937	2.735554	-2.364793
H	3.780906	1.805676	-1.119001
O	2.388493	0.768237	2.915983
H	3.009779	1.153282	2.280264
H	2.641690	-0.174928	2.889271
O	3.157513	-1.641764	2.079345
H	3.529019	-1.132822	1.343708
H	2.386761	-2.132086	1.751028

MSc’ + 3 WAT

N	0.230461	-1.534434	1.060843
H	-0.525018	-1.947945	0.500509
H	0.542904	-2.253141	1.718334
N	0.391540	-0.330006	-1.499436
Pt	1.760181	-1.056382	-0.170861
N	3.130158	-1.682517	1.206897
H	-0.491705	-0.837225	-1.433876
H	0.723467	-0.404345	-2.467726
H	2.734872	-2.198852	1.994742
H	3.585216	-0.825735	1.543054
H	0.260783	0.668038	-1.299176
H	3.842670	-2.261725	0.776746
C	-0.312405	-0.425701	1.843354
C	-1.398340	-0.913474	2.770242
H	0.494672	0.052028	2.400514
H	-0.697207	0.333381	1.157991
H	-0.941307	-1.553047	3.530494
H	-2.091292	-1.543118	2.195744
N	-2.059290	0.199064	3.407021
H	-2.855126	0.485186	2.844545
H	-2.408102	-0.060167	4.317514
C	-2.570524	1.250954	-1.933295
C	-3.027003	0.348123	-0.819829
C	-3.827568	1.147202	0.174396
C	-2.985069	2.311043	0.633210
C	-2.334388	3.073968	-0.498001
C	-1.257057	3.982849	0.011008
N	-3.746412	-0.794630	-1.330220
O	-4.172070	0.393685	1.298767
O	-1.721882	2.219406	-1.422931
O	-0.358494	3.248979	0.820791
S	-3.228584	-2.247154	-0.742145
O	-1.757119	-2.271449	-0.841690
O	-3.651721	-2.219740	0.660283
O	-3.879482	-3.241951	-1.568754
S	1.161757	3.140293	0.339013
O	1.636336	4.505567	0.202894
O	1.756110	2.404543	1.450367
O	1.152643	2.404170	-0.913076
H	-2.007634	0.684254	-2.677564
H	-2.122869	0.003564	-0.305223
H	-4.737229	1.500312	-0.331639
H	-2.197978	1.915449	1.279598
H	-3.079564	3.690626	-1.021500
H	-0.728491	4.441275	-0.825821
H	-1.687755	4.771271	0.628865
H	-3.828649	-0.833598	-2.338992
H	-4.173189	-0.557335	1.089286
H	-3.436220	1.708088	-2.435200
H	-3.587024	2.995455	1.235836
Cl	3.584670	-0.535289	-1.560224
O	1.499352	-3.061203	3.136915
H	1.454925	-2.750659	4.043721
H	1.516166	-4.019577	3.177747
O	1.733859	-0.430174	-4.008207
H	2.538490	-0.520244	-3.477094
H	1.714712	-1.169015	-4.617023
O	4.051681	1.011139	1.243292
H	4.135213	0.816559	0.302986
H	3.223162	1.521819	1.322220

TSb’ + 3 WAT
(-131.06 cm⁻¹)

N	0.505142	-1.822757	-0.655757
H	1.222779	-1.778773	0.080431
H	0.226091	-2.804193	-0.718354
N	-0.246457	0.024901	1.498071
Pt	-1.193799	-0.879653	-0.071396
N	-2.148317	-1.759470	-1.647711
H	0.689925	-0.346999	1.659378
H	-0.777568	-0.101332	2.369973
H	-1.738527	-1.541563	-2.549064
H	-3.128248	-1.448985	-1.675258
H	-0.188470	1.033398	1.329586
H	-2.126885	-2.771882	-1.520381
C	1.094134	-1.353966	-1.905175
C	2.243848	-2.205266	-2.371304
H	0.329382	-1.304249	-2.679892
H	1.456791	-0.338558	-1.740830
H	1.872475	-3.172388	-2.721375
H	2.914876	-2.381994	-1.522535
N	2.880973	-1.515926	-3.466173
H	3.264164	-0.635490	-3.139108
H	3.637566	-2.061412	-3.852201
C	2.157276	2.390284	1.471273
C	2.744974	1.312335	0.601970
C	3.111265	1.893438	-0.738759
C	1.864669	2.517818	-1.318582
C	1.157609	3.443231	-0.353226
C	-0.215871	3.808770	-0.828370
N	3.841942	0.661782	1.275248
O	3.567592	0.916311	-1.626539
O	0.979345	2.848730	0.902881
O	-0.955475	2.624382	-1.057609
S	3.830915	-0.989040	1.196143
O	2.436811	-1.431613	1.393770
O	4.280190	-1.279630	-0.166834
O	4.730168	-1.418238	2.246607
S	-2.285235	2.394697	-0.205719
O	-3.227634	3.410783	-0.625564
O	-2.650793	1.040117	-0.639293
O	-1.910054	2.459677	1.191469
H	1.908375	1.991316	2.456528
H	1.948783	0.583999	0.417512
H	3.888709	2.655192	-0.583967
H	1.177794	1.710322	-1.587180
H	1.732401	4.372384	-0.229889
H	-0.710480	4.427118	-0.078078
H	-0.164972	4.359201	-1.767802
H	3.975702	0.951689	2.236417
H	4.020415	0.201653	-1.143937
H	2.875479	3.211779	1.605905
H	2.107137	3.062009	-2.234043
Cl	-3.354399	-1.494934	1.309385
O	-1.487971	-3.970088	-0.066094
H	-1.679656	-4.888353	0.128702
H	-1.963759	-3.432677	0.582220
O	-1.934926	-0.380814	3.737813
H	-2.556308	-0.827578	3.137907
H	-1.715752	-1.003720	4.431205
O	-4.705108	-0.507722	-1.293480
H	-4.707138	-0.909448	-0.414613
H	-4.138450	0.271085	-1.164400

MSd’ + 3 WAT

N	0.392426	-1.933367	-0.127672
H	1.035275	-1.825891	0.662788
H	-0.147017	-2.797818	-0.007505
N	-0.238135	0.307808	1.574023
Pt	-0.960255	-0.454682	-0.179967
N	-1.785817	-1.276328	-1.858540
H	0.684782	-0.047470	1.822796
H	-0.909472	0.019416	2.299279
H	-1.402614	-0.912269	-2.723370
H	-2.796866	-1.092344	-1.838513
H	-0.209601	1.328697	1.541911
H	-1.676916	-2.292531	-1.836717
C	1.230970	-2.081384	-1.315171
C	2.101534	-3.305421	-1.197030
H	0.602690	-2.150110	-2.203495
H	1.856247	-1.193353	-1.410395
H	1.489737	-4.201417	-1.333547
H	2.510207	-3.338478	-0.178095
N	3.123222	-3.262554	-2.208440
H	3.824783	-2.591534	-1.919085
H	3.577976	-4.158686	-2.304522
C	2.167896	2.590860	1.259441
C	2.679229	1.468993	0.396750
C	2.887237	1.969178	-1.008026
C	1.573832	2.539015	-1.483325
C	0.997398	3.545775	-0.515353
C	-0.399382	3.949483	-0.874998
N	3.865131	0.887012	0.978330
O	3.267439	0.949472	-1.877659
O	0.939575	3.029456	0.786538
O	-1.193481	2.790535	-1.083669
S	3.842138	-0.760449	1.082806
O	2.518178	-1.158434	1.593575
O	4.021293	-1.192148	-0.307294
O	4.933087	-1.095053	1.974866
S	-2.340284	2.420342	-0.062295
O	-3.543627	3.096625	-0.469273
O	-2.467449	0.953102	-0.325350
O	-1.866576	2.706415	1.268187
H	2.017611	2.245988	2.284130
H	1.889099	0.711850	0.342951
H	3.659443	2.752393	-0.983389
H	0.870285	1.706167	-1.584328
H	1.610935	4.458730	-0.508067
H	-0.823036	4.562683	-0.079396
H	-0.409381	4.507220	-1.809967
H	4.139350	1.284795	1.868452
H	3.724145	0.240684	-1.395041
H	2.894533	3.416060	1.280899
H	1.695162	2.999400	-2.466595
Cl	-3.755807	-2.814208	1.253780
O	-1.587126	-3.818706	-0.624679
H	-1.619016	-4.763163	-0.780691
H	-2.315922	-3.598117	-0.008126
O	-2.367092	-0.717557	3.059800
H	-2.792872	-1.390148	2.496445
H	-2.432104	-1.030966	3.961899
O	-4.451869	-0.772194	-0.987936
H	-4.349466	-1.422044	-0.271250
H	-4.027894	0.031625	-0.658922

Optimized Structures in the Format of xyz Coordinates (6WAT)

Cl-PtN3 + 6 WAT

N	1.438213	0.015269	-0.266205
H	1.811468	-0.874826	-0.613834
H	1.526090	0.718915	-1.021138
N	-0.522838	-1.566392	-1.556895
Pt	-0.563472	-0.237530	-0.013319
N	-0.602053	1.164352	1.471861
H	0.284695	-2.194609	-1.511193
H	-0.485992	-1.030835	-2.429598
H	0.049026	0.958873	2.221736
H	-1.528511	1.265321	1.906193
H	-1.368435	-2.126785	-1.570709
H	-0.341831	2.070225	1.069773
C	2.250641	0.429828	0.867996
C	3.715169	0.412180	0.517781
H	1.968635	1.439840	1.168405
H	2.060266	-0.246775	1.705793
H	3.893312	1.120433	-0.295358
H	3.975370	-0.585475	0.135895
N	4.488445	0.816556	1.660629
H	4.482534	0.101935	2.375612
H	5.452368	0.981610	1.411024
Cl	-2.857083	-0.636243	0.160134
O	1.212388	2.040284	-2.127256
H	0.750491	2.635143	-1.514300
H	0.546106	1.608261	-2.687099
O	0.117613	3.499367	-0.087951
H	0.754513	4.122488	0.270411
H	-0.672412	4.009045	-0.283954
O	-0.491439	0.488356	-3.597902
H	-0.168268	0.330864	-4.488156
H	-1.391193	0.810531	-3.692641
O	-3.204229	1.178133	2.586739
H	-3.798543	1.929012	2.577311
H	-3.468478	0.593040	1.862410
O	1.917269	-2.728367	-0.746348
H	1.681032	-2.911658	0.187159
H	2.637342	-3.301867	-1.008118
O	0.916935	-2.586611	1.660205
H	0.208472	-1.977702	1.395241
H	0.507642	-3.291344	2.165394

H_2O-PtN3 + 6 WAT

N	-1.541597	-0.470714	0.059857
H	-1.681535	-1.476339	-0.088158
H	-1.654592	-0.305327	1.065808
N	0.882157	-1.890710	0.543737
Pt	0.410094	-0.058332	-0.229790
N	-0.004787	1.798965	-0.969040
H	0.136917	-2.582065	0.435716
H	1.103104	-1.779583	1.532342
H	-0.519041	2.317305	-0.242316
H	-0.571201	1.767518	-1.809538
H	1.718237	-2.256243	0.085955
H	0.845875	2.314015	-1.197835
C	-2.566647	0.264201	-0.671832
C	-3.942715	-0.242232	-0.328258
H	-2.497733	1.319727	-0.411989
H	-2.379966	0.148840	-1.741851
H	-4.125799	-0.072099	0.736036
H	-3.978235	-1.329477	-0.488114
N	-4.923841	0.487692	-1.084585
H	-4.900661	0.226077	-2.060647
H	-5.857321	0.304159	-0.747658
O	2.431822	0.335103	-0.209669
H	2.696433	1.171534	-0.669187
H	2.951024	-0.435792	-0.553022
O	1.833489	-0.061084	2.550108
H	2.251582	0.289546	1.749878
H	2.433763	0.083663	3.283523
O	2.767691	2.604105	-1.364326
H	3.178092	3.313466	-0.863209
H	3.123268	2.647044	-2.255624
O	3.423417	-1.923684	-0.907076
H	4.252014	-2.230427	-0.530835
H	3.431062	-2.165469	-1.836588
O	-1.456782	2.564734	1.272317
H	-1.206337	1.880267	1.927547
H	-1.532679	3.400059	1.732659
O	-1.509493	-3.307986	-0.164297
H	-1.512957	-3.733917	-1.024149
H	-2.055238	-3.847064	0.411901
O	-0.803447	0.454552	2.731507
H	0.165344	0.300216	2.736148
H	-1.121639	0.345926	3.629030

IDOA(2S)-CO-PtN3 + 6 WAT

N	0.391965	1.867857	0.647597
H	-0.482170	2.213546	0.230719
H	0.170683	1.646639	1.625062
N	2.013749	-0.303640	1.418972
Pt	0.821044	0.100717	-0.194251
N	-0.154797	0.578719	-1.906155
H	1.590601	-0.000190	2.295662
H	2.885327	0.209376	1.247836
H	0.574837	0.771484	-2.598972
H	-0.794599	1.367181	-1.823679
H	2.303459	-1.280137	1.499108
H	-0.670222	-0.258286	-2.179264
C	1.419996	2.897949	0.567224
C	1.007742	4.144292	1.302777
H	2.354917	2.508345	0.972865
H	1.596014	3.120493	-0.487598
H	0.891013	3.909084	2.363959
H	0.021742	4.464463	0.936599
N	2.031684	5.144900	1.163655
H	2.063832	5.502278	0.218540
H	1.861306	5.930561	1.773999
C	-2.844495	-1.457040	-1.474199
C	-2.998915	-0.856524	-0.099842
C	-2.254342	-1.690968	0.916871
C	-2.002474	-3.069779	0.364306
C	-1.085906	-2.911511	-0.826711
C	0.326903	-2.609709	-0.364194
O	-2.394315	0.423641	-0.041226
O	-2.926423	-1.695881	2.130340
O	-1.529058	-1.899878	-1.690262
O	0.755168	-3.200250	0.624373
O	1.026980	-1.786263	-1.052917
S	-3.165319	1.733564	-0.535494
O	-2.017550	2.617679	-0.755039
O	-3.864276	1.366859	-1.753200
O	-4.031494	2.145282	0.549227
H	-3.054444	-0.702994	-2.229992
H	-4.056791	-0.790645	0.171424
H	-1.282668	-1.188909	1.035980
H	-2.937491	-3.556990	0.082902
H	-1.014889	-3.856358	-1.380392
H	-2.279333	-1.979706	2.788853
H	-1.517691	-3.702257	1.105739
H	-3.556949	-2.275901	-1.605770
O	4.240301	0.678046	0.031202
H	5.172506	0.731364	0.242440
H	4.132962	-0.044825	-0.608463
O	3.509905	-1.366860	-1.633227
H	3.957130	-1.948163	-2.251958
H	2.593746	-1.697390	-1.482900
O	3.399919	-2.812247	0.938261
H	3.724470	-2.420977	0.120357
H	2.495554	-3.121578	0.752095
O	2.373712	0.635413	-3.235450
H	2.873966	0.023168	-2.673050
H	2.914484	1.417608	-3.345076
O	-0.072769	0.470909	3.147318
H	-0.253703	-0.488620	3.158127
H	-0.238840	0.803913	4.029792
O	-0.331682	-2.231857	3.003768
H	-0.038861	-2.688198	3.795388
H	0.107138	-2.662194	2.247935

IDOA(2S)-SO-PtN3 + 6 WAT

Pt	0.850567	-0.265509	-0.503403
N	1.922631	1.432373	-0.437683
N	1.649405	-0.873990	-2.278426
C	2.810870	1.678446	0.691887
H	1.197400	2.166672	-0.436478
H	2.492276	1.492341	-1.280462
N	0.140887	0.236330	1.338597
H	1.029446	-0.743865	-3.069315
H	1.820993	-1.873153	-2.150554
H	2.550604	-0.430035	-2.473796
C	3.537805	2.984410	0.520540
H	3.519639	0.853919	0.764290
H	2.227545	1.706158	1.611013
H	0.905990	0.133940	2.004541
H	-0.609513	-0.370832	1.655714
H	-0.220195	1.203835	1.297068
N	4.416720	3.196440	1.639934
H	2.806992	3.796791	0.489284
H	4.053096	2.975114	-0.450591
H	5.198891	2.556525	1.612887
H	4.796589	4.131618	1.634477
C	-1.391180	3.312639	0.111697
C	-2.645803	2.818096	0.832862
O	-0.299076	2.798235	0.439023
O	-1.560003	4.219321	-0.708767
C	-3.813617	2.632784	-0.109347
O	-2.354702	1.645451	1.552390
H	-2.900799	3.608302	1.548948
C	-3.652427	1.372702	-0.917860
H	-3.852077	3.501948	-0.765390
H	-4.755270	2.585888	0.442448
C	-3.273449	0.607197	1.423196
C	-3.403553	0.178357	-0.010769
O	-4.781355	1.086764	-1.686211
H	-2.768174	1.471166	-1.558665
H	-2.914630	-0.216730	2.038312
H	-4.265114	0.888998	1.793874
O	-2.173268	-0.384675	-0.476792
H	-4.208711	-0.550083	-0.136882
H	-4.948167	1.808907	-2.294369
S	-1.764479	-1.873110	-0.201591
O	-1.690032	-2.054988	1.248844
O	-0.411828	-1.904443	-0.801020
O	-2.699041	-2.749050	-0.858765
O	4.053317	-1.441275	0.227950
H	3.470179	-2.157167	-0.146925
H	4.894027	-1.847137	0.452363
O	2.492137	-1.072569	2.405425
H	3.146416	-1.122235	1.677313
H	2.974635	-1.119331	3.232048
O	2.322995	-3.160429	-0.645845
H	2.608185	-4.060399	-0.812181
H	1.690778	-3.183483	0.121417
O	0.952505	-3.079392	1.577853
H	1.453240	-2.357946	2.011180
H	0.025683	-2.806930	1.549600
O	-3.918351	-3.757789	1.495801
H	-3.187783	-3.224651	1.826291
H	-3.818620	-3.672188	0.542230
O	4.112105	0.345720	-1.776118
H	4.244895	-0.294712	-1.047173
H	4.946420	0.455277	-2.231913

GlcNS(6S)-SO-PtN3 + 6 WAT

N	1.364229	-2.271694	0.381649
H	1.946214	-1.752015	1.045995
H	1.266157	-3.212234	0.756880
N	0.180806	0.134763	1.400254
Pt	-0.436993	-1.411570	0.222219
N	-1.238950	-2.959773	-0.824125
H	1.179877	0.115970	1.615974
H	-0.347448	0.105488	2.280247
H	-0.652137	-3.771110	-0.976011
H	-1.587270	-2.628321	-1.734795
H	-0.029032	1.029510	0.955739
H	-2.048711	-3.237239	-0.253265
C	2.128196	-2.305349	-0.867623
C	3.457871	-2.987367	-0.675973
H	1.541998	-2.798100	-1.643030
H	2.301905	-1.272773	-1.171112
H	3.311950	-4.066539	-0.583962
H	3.891990	-2.635821	0.270870
N	4.290000	-2.710939	-1.815241
H	4.618833	-1.754902	-1.743859
H	5.096543	-3.317458	-1.833808
C	1.514739	3.109765	0.865978
C	2.370984	2.191643	0.032333
C	2.354397	2.673927	-1.394206
C	0.913309	2.613950	-1.842039
C	-0.053684	3.310191	-0.910036
C	-1.459005	2.845052	-1.157795
N	3.693688	2.038952	0.585558
O	3.124968	1.886842	-2.242081
O	0.183652	3.004662	0.448501
O	-1.446052	1.476825	-0.760005
S	4.159433	0.463294	0.757736
O	3.082014	-0.256204	1.463148
O	4.265432	-0.002318	-0.630097
O	5.402466	0.510533	1.494955
S	-2.776032	0.667730	-0.624917
O	-3.344054	0.495171	-1.946523
O	-2.315727	-0.584934	-0.014602
O	-3.644621	1.403873	0.287628
H	1.537277	2.816237	1.916345
H	1.884149	1.210863	0.026717
H	2.727287	3.707046	-1.418579
H	0.649318	1.554286	-1.913820
H	-0.008821	4.396411	-1.044090
H	-2.179499	3.393098	-0.548459
H	-1.727241	2.923888	-2.211417
H	3.878831	2.561872	1.432838
H	3.627842	1.223755	-1.737935
H	1.862307	4.146835	0.788175
H	0.808678	3.046667	-2.838711
O	-3.890341	-0.731653	2.372053
H	-3.107567	-0.385416	2.845228
H	-4.088188	-0.058572	1.712322
O	-3.333448	-3.016011	1.066915
H	-3.796455	-3.689761	1.563424
H	-3.505885	-2.158791	1.515600
O	-1.996806	2.672055	2.147922
H	-2.682672	2.382786	1.527710
H	-1.222131	2.923669	1.620062
O	-1.734140	0.452027	3.465618
H	-1.629256	0.491314	4.417140
H	-1.817609	1.374438	3.119778
O	-2.275559	-1.730442	-3.141397
H	-2.859247	-2.184282	-3.749525
H	-2.742564	-0.938865	-2.832390
O	-5.719891	2.000088	-1.545986
H	-5.281548	2.024863	-0.689832
H	-5.119249	1.465121	-2.073491

GlcNS(6S)-NS-PtN3 + 6 WAT

N	-0.656436	2.168385	-0.887786
H	-0.595700	2.355239	-1.885067
H	-1.574025	1.720859	-0.739414
N	-0.318532	0.567051	1.384440
Pt	0.706476	0.793672	-0.369584
N	1.857569	1.209122	-1.998583
H	-1.320597	0.698211	1.234402
H	0.048667	1.292358	2.005151
H	2.235316	2.144748	-1.847652
H	1.390578	1.186150	-2.898011
H	-0.213704	-0.340028	1.853627
H	2.654918	0.562809	-2.040178
C	-0.595449	3.436209	-0.164173
C	-1.697654	4.364761	-0.596855
H	-0.677895	3.235303	0.904424
H	0.389235	3.876767	-0.333729
H	-2.661415	3.904155	-0.362457
H	-1.659605	4.485741	-1.689164
N	-1.590104	5.603629	0.125400
H	-0.792316	6.139682	-0.187749
H	-2.408557	6.178479	-0.010364
C	-1.266435	-3.056229	-1.046897
C	-0.393880	-2.444956	0.021900
C	-0.962537	-2.799938	1.375176
C	-2.403802	-2.372797	1.426026
C	-3.198172	-2.816919	0.223854
C	-4.557306	-2.173099	0.182015
N	0.966472	-2.898868	-0.162997
O	-0.310786	-2.127181	2.416748
O	-2.538072	-2.507868	-0.972979
O	-4.591187	-0.853732	0.689132
S	2.235928	-2.058633	0.374898
O	2.139063	-0.614754	0.031963
O	2.296524	-2.182841	1.830020
O	3.383804	-2.618775	-0.333462
S	-4.205993	0.403527	-0.221153
O	-4.977255	1.473346	0.374869
O	-2.760157	0.584832	-0.036886
O	-4.567969	0.053153	-1.579224
H	-0.868667	-2.829862	-2.036901
H	-0.464555	-1.361408	-0.123973
H	-0.866792	-3.885379	1.509276
H	-2.430814	-1.282607	1.466964
H	-3.354242	-3.905582	0.255714
H	-4.933243	-2.181555	-0.842047
H	-5.242635	-2.724219	0.823504
H	1.193744	-3.325423	-1.071985
H	0.630127	-2.362911	2.438700
H	-1.305703	-4.149802	-0.930903
H	-2.869749	-2.748745	2.339243
O	3.266172	0.399121	2.592497
H	2.852873	-0.462525	2.444713
H	2.565675	1.079258	2.590254
O	2.760504	3.222810	0.025862
H	3.474610	2.527138	0.116087
H	3.191632	4.078383	0.002355
O	4.421455	1.303482	0.368065
H	4.041528	0.860566	1.159761
H	4.397999	0.662828	-0.365239
O	4.247744	-0.417925	-1.734065
H	4.054398	-1.280798	-1.327052
H	4.994149	-0.529710	-2.324854
O	1.566215	2.490234	2.302036
H	1.620185	3.165801	2.979517
H	1.939092	2.868977	1.470516
O	2.215065	-4.076554	-2.329235
H	2.976856	-3.684460	-1.882640
H	2.234197	-3.779400	-3.239500

Common Aquation Step

MS1 + 6 WAT

N	1.526941	0.520813	-0.073566
H	1.996578	0.349695	-0.969352
H	1.324569	1.535737	-0.000572
N	-0.277515	0.033711	-2.194992
Pt	-0.306054	-0.342151	-0.191397
N	-0.379978	-0.622247	1.829193
H	0.639129	-0.147675	-2.613407
H	-0.535817	1.018180	-2.323107
H	0.497070	-0.925976	2.235066
H	-1.094719	-1.305366	2.111259
H	-0.966301	-0.532696	-2.678536
H	-0.627234	0.287950	2.232196
C	2.446181	0.143457	0.990765
C	3.796279	0.777325	0.779104
H	2.047521	0.472875	1.951218
H	2.546168	-0.945145	1.006421
H	3.685149	1.864352	0.802418
H	4.155656	0.514033	-0.226153
N	4.686477	0.380555	1.836055
H	4.951293	-0.590689	1.744242
H	5.536266	0.924956	1.823208
Cl	-2.472607	-1.209441	-0.461978
O	0.556077	3.078689	0.282866
H	-0.027118	2.839073	1.025993
H	-0.028450	3.095591	-0.496456
O	-3.009174	1.780815	0.063834
H	-3.909016	2.112317	0.112858
H	-3.054783	0.820327	-0.094602
O	-1.247127	2.097184	2.061955
H	-1.556106	2.593827	2.821205
H	-1.993513	2.021539	1.432987
O	-1.231733	2.684199	-1.716178
H	-1.532477	3.357691	-2.327928
H	-1.990956	2.430111	-1.152108
O	-2.388387	-2.551434	2.304141
H	-3.076524	-2.477127	2.966091
H	-2.785511	-2.332659	1.451353
O	2.443624	-0.667463	-2.465190
H	2.440220	-1.525142	-1.990988
H	3.210871	-0.627293	-3.035872
O	1.970676	-2.608657	-0.775116
H	1.107590	-2.261718	-0.501153
H	1.849056	-3.539638	-0.970204

TS1 + 6 WAT
(-145.98 cm^{-1})

N	-2.066130	0.194221	-0.741616
H	-2.355256	0.996066	-0.161182
H	-2.272894	0.433090	-1.707282
N	-0.054647	2.160504	-0.822187
Pt	-0.049414	0.147914	-0.480646
N	0.036309	-1.869884	-0.204161
H	-0.719073	2.636556	-0.207796
H	-0.322386	2.364977	-1.779656
H	-0.679886	-2.390745	-0.695187
H	-0.023973	-2.097663	0.795580
H	0.885309	2.544924	-0.697234
H	0.945402	-2.202387	-0.547976
C	-2.853974	-0.971127	-0.364313
C	-4.328243	-0.662664	-0.374379
H	-2.656022	-1.788326	-1.058201
H	-2.541470	-1.287271	0.633040
H	-4.628738	-0.385693	-1.387988
H	-4.513030	0.212729	0.264262
N	-5.062648	-1.834353	0.018129
H	-4.956151	-2.018890	1.006147
H	-6.049642	-1.725801	-0.162177
Cl	1.900693	0.356292	1.237120
O	1.960139	0.151567	-1.961442
H	2.399692	-0.688369	-1.732056
H	2.495248	0.892863	-1.639994
O	4.105958	-1.782638	0.949034
H	4.206825	-2.407278	1.668605
H	3.517774	-1.075923	1.258787
O	2.702378	-2.284635	-1.168468
H	3.030564	-2.945422	-1.779180
H	3.315461	-2.238708	-0.396450
O	2.802690	2.426083	-0.729482
H	3.488425	3.084381	-0.851182
H	2.916409	2.025071	0.142931
O	0.195537	-1.890485	2.596145
H	0.569169	-2.557421	3.172968
H	0.859117	-1.194811	2.497777
O	-2.209712	2.384512	0.972199
H	-1.654660	2.139881	1.750037
H	-2.910347	2.966451	1.266359
O	-0.522834	1.607054	2.798905
H	0.266271	1.266822	2.348013
H	-0.219735	2.204299	3.484239

MS2 + 6 WAT

N	-1.983223	-0.588733	-0.406765
H	-2.178670	0.304482	-0.884690
H	-2.451665	-1.307127	-0.954044
N	-0.060272	-0.042616	-2.438890
Pt	0.006592	-0.899773	-0.585607
N	0.157488	-1.671902	1.300523
H	-0.803211	0.662369	-2.497396
H	-0.203697	-0.724966	-3.175402
H	-0.473161	-2.443548	1.483526
H	-0.030938	-0.908949	1.963593
H	0.825301	0.435608	-2.615076
H	1.108661	-1.993313	1.493139
C	-2.586459	-0.540404	0.921649
C	-4.028381	-0.114184	0.846813
H	-2.526020	-1.525151	1.385503
H	-2.009706	0.156247	1.533965
H	-4.593806	-0.850075	0.269150
H	-4.089580	0.836871	0.298482
N	-4.577168	-0.054397	2.174858
H	-4.193149	0.724764	2.691893
H	-5.579008	0.065905	2.152174
Cl	1.747498	2.510928	0.701282
O	2.004031	-1.217053	-0.890601
H	2.495982	-1.450377	-0.055901
H	2.394492	-0.391938	-1.272028
O	3.479046	0.581249	2.358895
H	3.314620	0.739902	3.289659
H	2.970453	1.246242	1.860342
O	3.003081	-1.742512	1.387444
H	3.722802	-2.363113	1.510308
H	3.240268	-0.881272	1.826638
O	2.520705	1.096778	-1.870572
H	3.314473	1.409730	-2.307660
H	2.343628	1.678564	-1.103409
O	-0.120996	0.822231	2.546307
H	0.035885	1.103362	3.448357
H	0.439046	1.376648	1.977214
O	-2.089548	1.806261	-1.822362
H	-1.678523	2.543545	-1.305188
H	-2.852889	2.151299	-2.286048
O	-0.919596	3.597926	-0.365596
H	-0.059141	3.291391	-0.027042
H	-0.786608	4.481465	-0.711470

IdoA(2S)-model (Via aquation)

MS3 + 6 WAT

N	0.479430	-1.159716	1.108692
H	1.212486	-0.527366	0.781599
H	0.256685	-0.887292	2.076493
N	-2.375056	-1.125213	1.543822
Pt	-1.135538	-0.934795	-0.060671
N	0.102613	-0.636491	-1.670927
H	-1.875060	-1.200117	2.428374
H	-3.007781	-1.916798	1.413986
H	-0.332341	-0.960449	-2.534732
H	1.012243	-1.094158	-1.580514
H	-2.942126	-0.263166	1.606441
H	0.272214	0.369233	-1.759023
C	1.030253	-2.513304	1.083496
C	2.364028	-2.581266	1.789438
H	0.305131	-3.200018	1.520686
H	1.178648	-2.797727	0.042893
H	2.219187	-2.722052	2.863067
H	2.881641	-1.620495	1.657824
N	3.114213	-3.687978	1.256848
H	3.405204	-3.447578	0.315303
H	3.947599	-3.858327	1.800966
O	-2.808515	-0.883916	-1.275343
H	-2.568423	-1.105976	-2.206672
H	-3.321416	-0.007053	-1.271288
C	2.124954	2.314827	-1.625308
C	2.599560	1.760989	-0.302765
C	1.679193	2.234011	0.792902
C	1.123122	3.581004	0.412181
C	0.215701	3.376913	-0.776934
C	-1.178538	2.905591	-0.398053
O	2.492744	0.346602	-0.303168
O	2.330221	2.212305	2.016525
O	0.725207	2.372264	-1.635390
O	-1.459263	2.716004	0.805061
O	-1.956332	2.735936	-1.349530
S	3.676411	-0.561520	-0.924668
O	2.931545	-1.788545	-1.179343
O	4.123044	0.138222	-2.113209
O	4.677480	-0.674573	0.115006
H	2.438369	1.658635	-2.434214
H	3.629672	2.058335	-0.087787
H	0.833593	1.531964	0.808408
H	1.933744	4.276927	0.185282
H	0.100573	4.304878	-1.348029
H	1.629234	2.268593	2.683850
H	0.552885	4.006951	1.236263
H	2.553191	3.305554	-1.805367
O	-4.055809	-2.870003	0.093519
H	-5.005766	-2.959181	0.023000
H	-3.776546	-2.199808	-0.551482
O	-3.987602	1.319794	-1.180334
H	-4.658172	1.532258	-1.831237
H	-3.211122	1.977751	-1.256581
O	-3.665952	1.376367	1.491649
H	-4.127626	1.337376	0.641464
H	-2.874006	1.916195	1.285272
O	-1.875910	-1.494017	-3.624329
H	-1.945219	-2.411574	-3.899267
H	-2.096945	-0.955639	-4.388166
O	-0.518403	-0.301225	3.573718
H	-0.437285	0.674268	3.472950
H	-0.149076	-0.540186	4.424271
O	-0.214594	2.265422	3.039797
H	-0.508199	2.889986	3.705574
H	-0.676718	2.492490	2.191611

TS2 + 6 WAT
$(-137.36\ cm^{-1})$

N	0.601511	-1.220304	1.199713
H	1.370558	-0.613952	0.905323
H	0.399293	-0.940057	2.165738
N	-2.182242	-0.604695	1.628610
Pt	-0.922742	-0.651015	0.026312
N	0.276116	-0.785162	-1.614151
H	-1.695904	-0.374347	2.496412
H	-2.594594	-1.540222	1.708713
H	-0.141918	-1.411556	-2.304406
H	1.233418	-1.091936	-1.431327
H	-2.956252	0.062009	1.503340
H	0.305575	0.163780	-1.997721
C	1.051536	-2.604086	1.109776
C	2.363146	-2.801723	1.837623
H	0.266278	-3.258141	1.488235
H	1.204227	-2.838006	0.057286
H	2.183855	-3.083982	2.877108
H	2.898893	-1.841344	1.852740
N	3.113931	-3.840085	1.182522
H	3.458444	-3.471676	0.302742
H	3.913297	-4.116237	1.734200
O	-2.875866	-1.719113	-1.225552
H	-2.464402	-2.041039	-2.048768
H	-3.563149	-1.058268	-1.445632
C	1.858816	2.219273	-1.880872
C	2.336006	1.996508	-0.465706
C	1.325741	2.550115	0.510400
C	0.500073	3.605375	-0.178388
C	-0.315407	2.909542	-1.240976
C	-1.480818	2.148437	-0.628934
O	2.432162	0.608467	-0.182181
O	1.955853	2.999648	1.661475
O	0.474594	2.016625	-1.986583
O	-2.015486	2.635746	0.379449
O	-1.870287	1.095968	-1.203768
S	3.728590	-0.244569	-0.631665
O	3.101539	-1.546649	-0.837868
O	4.222980	0.391168	-1.836796
O	4.641434	-0.202183	0.491011
H	2.338971	1.506910	-2.547878
H	3.305866	2.476742	-0.310529
H	0.657493	1.712663	0.760292
H	1.139021	4.379591	-0.606957
H	-0.765405	3.645746	-1.919177
H	1.252845	3.103174	2.317702
H	-0.171581	4.092524	0.524845
H	2.130868	3.224665	-2.213924
O	-3.164602	-3.163016	0.951472
H	-4.014000	-3.592031	1.047582
H	-3.143081	-2.740202	0.067082
O	-4.337263	0.439417	-1.495111
H	-4.820638	0.756318	-2.258973
H	-3.433153	0.839747	-1.494815
O	-4.225724	1.313906	1.086265
H	-4.565719	0.990782	0.238701
H	-3.479756	1.897960	0.841698
O	-1.386923	-2.494570	-3.304451
H	-1.190779	-3.431630	-3.374776
H	-1.597809	-2.191304	-4.190399
O	-0.210136	0.250328	3.480072
H	-0.327743	1.201469	3.269316
H	-0.050720	0.173076	4.420966
O	-0.622197	2.752581	2.680216
H	-1.038430	3.326550	3.326354
H	-1.187835	2.758980	1.877610

MS4 + 6 WAT

N	0.536323	1.719072	0.821797
H	-0.320955	2.144090	0.446459
H	0.320703	1.473088	1.793593
N	1.850465	-0.697071	1.499129
Pt	0.789150	-0.037463	-0.116483
N	-0.108342	0.674921	-1.792722
H	1.358403	-0.483577	2.366580
H	2.748111	-0.192060	1.493057
H	0.618675	0.925797	-2.465670
H	-0.700171	1.487514	-1.625552
H	2.083948	-1.695084	1.476535
H	-0.675721	-0.093384	-2.152967
C	1.617817	2.695798	0.754490
C	1.281019	3.932295	1.543959
H	2.541237	2.240187	1.114043
H	1.771122	2.953891	-0.296599
H	1.190296	3.666944	2.600682
H	0.297593	4.304911	1.222725
N	2.340196	4.896205	1.403174
H	2.360541	5.277135	0.466930
H	2.215722	5.671566	2.037402
O	4.112280	0.040502	-1.550076
H	3.491424	0.515658	-2.127802
H	3.832023	-0.895775	-1.540783
C	-3.050441	-0.987544	-1.682971
C	-3.226795	-0.475328	-0.276020
C	-2.679557	-1.476237	0.713953
C	-2.567563	-2.831559	0.066605
C	-1.549731	-2.712466	-1.043816
C	-0.147972	-2.635844	-0.464457
O	-2.459524	0.697931	-0.071175
O	-3.434414	-1.477509	1.877908
O	-1.793043	-1.590947	-1.851375
O	0.105623	-3.323457	0.521729
O	0.714713	-1.899069	-1.060048
S	-3.018084	2.139062	-0.476921
O	-1.749179	2.867038	-0.556967
O	-3.684111	1.976736	-1.755988
O	-3.886029	2.573018	0.597391
H	-3.101478	-0.157563	-2.385039
H	-4.283385	-0.280399	-0.070203
H	-1.665119	-1.119043	0.937201
H	-3.534202	-3.165867	-0.314001
H	-1.559118	-3.615300	-1.667528
H	-2.867799	-1.867063	2.556728
H	-2.224843	-3.575311	0.783384
H	-3.853315	-1.687947	-1.928719
O	4.202522	0.750044	0.951399
H	5.076850	0.571505	1.296578
H	4.211232	0.524149	-0.012559
O	3.232226	-2.484000	-1.384701
H	3.429027	-3.065708	-2.120821
H	2.258495	-2.337639	-1.362592
O	2.728469	-3.434106	1.132641
H	3.149044	-3.230050	0.283209
H	1.791049	-3.577696	0.915090
O	2.353516	1.379713	-3.181241
H	2.499434	2.328625	-3.160720
H	2.460289	1.115857	-4.098224
O	-0.229468	0.206571	3.206427
H	-0.588028	-0.700394	3.145434
H	-0.333254	0.492492	4.114499
O	-0.996405	-2.374297	2.879225
H	-0.824646	-2.923327	3.647068
H	-0.566135	-2.805023	2.117704

MS3' + 6 WAT

Pt	1.057466	-0.829946	0.169920
N	2.411386	0.525239	-0.432993
N	1.462495	-1.989155	-1.467741
C	3.780647	0.258595	0.000239
H	2.136149	1.452142	-0.051469
H	2.392891	0.622330	-1.445142
N	0.639738	0.322671	1.795711
H	2.056058	-1.534249	-2.152600
H	0.600926	-2.251675	-1.957100
H	1.934762	-2.862861	-1.193079
C	4.717671	1.342604	-0.461141
H	4.096197	-0.713252	-0.385599
H	3.790379	0.199292	1.088684
H	1.171148	0.005436	2.600561
H	-0.348179	0.306638	2.080362
H	0.867536	1.310001	1.605194
N	6.055658	1.037992	-0.031129
H	4.404514	2.287471	-0.010491
H	4.620729	1.460113	-1.550304
H	6.441348	0.266305	-0.557893
H	6.671047	1.827113	-0.162061
C	1.139108	3.779727	0.434791
C	-0.381196	3.753546	0.408732
O	1.730586	2.771254	0.892018
O	1.668424	4.805600	0.007026
C	-0.917709	3.976159	-0.997983
O	-0.822259	2.532283	0.927195
H	-0.717040	4.570833	1.059656
C	-1.122392	2.640347	-1.663819
H	-0.207988	4.586764	-1.556363
H	-1.869912	4.513359	-0.982665
C	-2.121678	2.204461	0.552025
C	-2.202612	1.899493	-0.927079
O	-1.513736	2.746432	-2.996823
H	-0.204774	2.043458	-1.578072
H	-2.416031	1.345243	1.151564
H	-2.809684	3.025248	0.787036
O	-1.997518	0.521905	-1.237068
H	-3.177129	2.191305	-1.323399
H	-0.788546	3.089272	-3.520924
S	-3.065983	-0.555160	-0.794592
O	-2.563308	-1.148672	0.444682
O	-3.084064	-1.494175	-1.904612
O	-4.315260	0.151050	-0.566432
O	-0.325647	-2.184833	0.862981
H	-0.303752	-3.090614	0.448982
H	-1.236680	-1.798872	0.681566
O	-0.366812	-4.424588	-0.397424
H	-0.734213	-4.078903	-1.252673
H	-0.945615	-5.114236	-0.065897
O	-0.354980	-1.976841	3.536709
H	-0.271675	-2.241254	2.603380
H	-0.463423	-2.773346	4.056203
O	-1.059409	-3.067121	-2.450378
H	-1.125669	-3.412759	-3.342233
H	-1.837125	-2.484770	-2.288725
O	-1.906771	0.165683	3.134658
H	-1.582533	-0.707320	3.409326
H	-2.812388	0.082686	2.788952
O	-4.440759	0.444037	2.166356
H	-5.189815	0.032125	2.596950
H	-4.532800	0.284571	1.212100
O	2.287242	-4.560601	-0.680054
H	1.352764	-4.746159	-0.482610
H	2.790040	-4.731244	0.116278

TS2' + 6 WAT

(-149.64 cm^{-1})

Pt	1.220820	0.091196	-0.345529
N	1.555232	2.043065	-0.638174
N	2.460049	-0.411501	-1.895407
C	2.453029	2.692722	0.313090
H	0.620624	2.494949	-0.580331
H	1.899334	2.205835	-1.580061
N	0.055959	0.637342	1.224831
H	2.415114	0.221608	-2.686090
H	2.255910	-1.346339	-2.255266
H	3.430980	-0.442191	-1.552255
C	2.536106	4.172950	0.050190
H	3.437762	2.227390	0.243074
H	2.077270	2.522675	1.322021
H	0.630431	0.842693	2.040815
H	-0.580108	-0.091989	1.534627
H	-0.529371	1.456043	0.980501
N	3.434364	4.778342	0.995748
H	1.540985	4.605702	0.179701
H	2.822508	4.337371	-0.998432
H	4.394948	4.538281	0.792301
H	3.366093	5.785022	0.972080
C	-2.100999	3.166730	-0.306806
C	-3.233526	2.452884	0.429982
O	-0.927757	2.894280	0.039980
O	-2.440928	3.982807	-1.164399
C	-4.259493	1.905775	-0.535961
O	-2.708672	1.455253	1.268263
H	-3.710331	3.216541	1.054681
C	-3.716410	0.684040	-1.227248
H	-4.483161	2.686811	-1.262129
H	-5.187212	1.649800	-0.019333
C	-3.313124	0.202222	1.187597
C	-3.247747	-0.342554	-0.210436
O	-4.662824	0.064282	-2.043571
H	-2.835431	0.971068	-1.814238
H	-2.781477	-0.444147	1.886430
H	-4.361829	0.235812	1.503014
O	-1.890250	-0.634786	-0.554292
H	-3.850440	-1.248767	-0.311102
H	-4.947460	0.674142	-2.726371
S	-1.289041	-2.069055	-0.345633
O	-1.159633	-2.293533	1.098315
O	0.036747	-1.903161	-0.955461
O	-2.147764	-3.023128	-1.004469
O	1.637887	-1.839358	1.267043
H	2.336979	-2.423350	0.896440
H	0.803138	-2.327981	1.267989
O	3.474091	-3.203890	-0.021701
H	2.970942	-3.401476	-0.848929
H	3.852837	-4.019547	0.311010
O	1.502740	-0.388106	3.435727
H	1.762897	-0.990636	2.705506
H	2.143110	-0.470659	4.142408
O	1.942757	-3.256868	-2.104497
H	1.869924	-3.920405	-2.792218
H	1.051632	-3.038341	-1.777795
O	-1.136508	-0.989108	3.573627
H	-0.187629	-0.804557	3.691174
H	-1.193626	-1.675770	2.903429
O	-3.054410	-4.422738	1.246953
H	-2.426127	-3.811060	1.641821
H	-2.993279	-4.198571	0.311878
O	4.890866	-1.017160	-0.645118
H	4.514781	-1.853648	-0.312624
H	5.173008	-0.508398	0.115017

MS4' + 6 WAT

Pt	0.994175	-0.037496	-0.585564
N	1.945155	1.720874	-0.499865
N	1.931603	-0.503266	-2.342465
C	3.008335	1.843852	0.495719
H	1.189275	2.408266	-0.318015
H	2.328320	1.952745	-1.412414
N	0.164195	0.484862	1.199450
H	1.676196	0.099272	-3.116731
H	1.747537	-1.468044	-2.621883
H	2.943127	-0.440515	-2.173363
C	3.588027	3.232703	0.505447
H	3.770957	1.097561	0.262510
H	2.599381	1.615426	1.480294
H	0.847441	0.486534	1.956942
H	-0.578315	-0.121363	1.535310
H	-0.253179	1.425830	1.089399
N	4.632649	3.304632	1.491525
H	2.804735	3.946770	0.772390
H	3.921939	3.490261	-0.510137
H	5.442421	2.772673	1.203106
H	4.935355	4.256620	1.635675
C	-1.479001	3.415853	-0.155018
C	-2.735621	2.863660	0.519222
O	-0.375263	3.000098	0.267418
O	-1.657658	4.263320	-1.032097
C	-3.827009	2.537886	-0.475126
O	-2.395972	1.755669	1.313555
H	-3.091664	3.666642	1.175670
C	-3.523369	1.247893	-1.189405
H	-3.885013	3.360910	-1.187013
H	-4.795835	2.454518	0.022801
C	-3.232031	0.647206	1.214036
C	-3.269401	0.126818	-0.194486
O	-4.565042	0.838750	-2.021667
H	-2.598826	1.371752	-1.766029
H	-2.838972	-0.098599	1.904404
H	-4.256839	0.878636	1.525033
O	-1.984242	-0.395668	-0.561174
H	-4.026998	-0.650805	-0.316754
H	-4.723223	1.505178	-2.692580
S	-1.524204	-1.856418	-0.270324
O	-1.496338	-2.061860	1.175004
O	-0.132833	-1.799451	-0.803657
O	-2.354998	-2.783261	-0.989468
O	1.253465	-2.625820	1.927620
H	1.883720	-2.678796	1.181521
H	0.361190	-2.577132	1.564160
O	3.090412	-2.883974	-0.033615
H	2.594220	-3.184205	-0.831273
H	3.671054	-3.601642	0.229507
O	1.384376	-0.598964	3.508893
H	1.481522	-1.417649	2.959008
H	2.009194	-0.636627	4.232985
O	1.552335	-3.335313	-2.096821
H	1.477130	-4.149431	-2.597372
H	0.685864	-3.118018	-1.719882
O	-1.311694	-0.704003	3.680603
H	-0.344076	-0.656949	3.786873
H	-1.482395	-1.460090	3.115270
O	-3.335846	-4.264092	1.201468
H	-2.756382	-3.636474	1.642061
H	-3.240388	-4.021866	0.274605
O	4.355785	-0.702005	-0.998301
H	3.990661	-1.502313	-0.571564
H	5.245811	-0.914042	-1.280258

GlcNS(6S)-model (Via aquation)

MS5 + 6 WAT

N	1.282619	-1.418652	-1.065139
H	1.124285	-1.073236	-2.008062
H	2.009869	-0.814074	-0.654744
N	0.650720	-0.807056	1.734380
Pt	-0.393650	-1.219757	0.026275
N	-1.550105	-1.574263	-1.609806
H	1.663926	-0.829831	1.619229
H	0.370141	-1.476449	2.450831
H	-2.163092	-2.383295	-1.455521
H	-1.059793	-1.722648	-2.484499
H	0.404775	0.136168	2.060538
H	-2.130918	-0.739514	-1.709906
C	1.837442	-2.768566	-1.129579
C	3.072307	-2.805123	-1.989956
H	2.081990	-3.084924	-0.115438
H	1.075927	-3.449216	-1.515300
H	3.819164	-2.130012	-1.563845
H	2.824853	-2.412671	-2.987311
N	3.605141	-4.140443	-2.012122
H	3.020362	-4.758399	-2.557848
H	4.526693	-4.159823	-2.423163
C	0.774317	3.143288	-1.159721
C	-0.102685	2.533492	-0.093146
C	0.456089	2.814057	1.286226
C	1.934862	2.547117	1.344409
C	2.659017	3.159635	0.177157
C	4.132764	2.843469	0.165752
N	-1.447016	3.055990	-0.222159
O	-0.148634	1.999298	2.252814
O	2.087864	2.713007	-1.024958
O	4.490231	1.681992	0.887511
S	-2.698600	2.035482	-0.120282
O	-2.616483	0.973426	-1.111753
O	-2.608238	1.475204	1.250397
O	-3.892287	2.867400	-0.304847
S	4.500804	0.236718	0.210863
O	5.575734	-0.438321	0.902199
O	3.185198	-0.340989	0.523261
O	4.692539	0.439805	-1.210729
H	0.422700	2.844224	-2.147154
H	-0.102613	1.446380	-0.249306
H	0.256258	3.871264	1.508377
H	2.106658	1.469684	1.313000
H	2.562594	4.255344	0.202821
H	4.477073	2.757437	-0.865699
H	4.682035	3.641099	0.661741
H	-1.625961	3.809979	-0.892265
H	-1.107511	1.974546	2.102234
H	0.729308	4.241773	-1.097027
H	2.341096	2.917885	2.288105
O	-2.110976	-1.001425	1.137019
H	-2.282186	-0.029180	1.296507
H	-1.964540	-1.511665	1.990379
O	-3.425148	-3.538316	-0.757702
H	-4.052186	-2.795468	-0.545537
H	-3.878282	-4.183585	-1.301758
O	-4.584655	-1.424451	0.045099
H	-3.748947	-1.172207	0.467573
H	-4.967176	-0.617431	-0.359248
O	-5.670993	0.834251	-0.904396
H	-5.141141	1.632297	-0.735785
H	-6.551001	1.005881	-0.567667
O	-1.389053	-2.578803	2.929908
H	-1.782961	-2.707765	3.793766
H	-1.586665	-3.377778	2.368383
O	-1.915714	-4.418774	1.222512
H	-2.519149	-4.125691	0.501401
H	-2.189380	-5.296176	1.491349
O	-2.867982	4.958710	-1.685707
H	-3.542056	4.354557	-1.339491
H	-3.020172	5.048947	-2.626330

TS3 + 6 WAT

(-149.75 cm⁻¹)

N	-0.376971	2.164157	-1.006476
H	-0.094933	2.416252	-1.949099
H	-1.278550	1.671257	-1.081651
N	-0.387850	0.673538	1.365996
Pt	0.877872	0.809470	-0.226160
N	2.167564	0.951761	-1.802591
H	-1.349488	0.849866	1.069265
H	-0.090503	1.364000	2.059220
H	2.806896	1.744782	-1.684385
H	1.711139	1.040163	-2.703801
H	-0.386401	-0.247288	1.822029
H	2.731271	0.097438	-1.841662
C	-0.585445	3.387319	-0.231982
C	-1.706230	4.205054	-0.817342
H	-0.837742	3.115261	0.792971
H	0.355062	3.939536	-0.190052
H	-2.634883	3.629437	-0.749337
H	-1.508540	4.378870	-1.885259
N	-1.858786	5.416772	-0.056585
H	-1.093987	6.054853	-0.228614
H	-2.711581	5.897652	-0.301919
C	-1.804849	-3.227504	-0.807611
C	-0.806468	-2.666083	0.173897
C	-1.413899	-2.679630	1.555909
C	-2.747115	-1.983706	1.530854
C	-3.632329	-2.452787	0.404120
C	-4.859659	-1.594642	0.258529
N	0.419904	-3.437087	0.115047
O	-0.613614	-1.997755	2.481896
O	-2.950640	-2.445781	-0.819990
O	-4.654567	-0.226401	0.552991
S	1.818831	-2.628133	0.309824
O	1.757285	-1.363253	-0.428470
O	1.960238	-2.369114	1.750988
O	2.860490	-3.495135	-0.243994
S	-4.061558	0.778673	-0.540164
O	-4.583577	2.060651	-0.115424
O	-2.603511	0.694697	-0.388129
O	-4.531535	0.308295	-1.826970
H	-1.390012	-3.218711	-1.816049
H	-0.617738	-1.626580	-0.122169
H	-1.529201	-3.728672	1.859551
H	-2.574645	-0.915564	1.397654
H	-3.977335	-3.479943	0.596838
H	-5.257411	-1.695150	-0.752338
H	-5.613581	-1.912241	0.976544
H	0.508035	-4.093450	-0.669593
H	0.298064	-2.331323	2.453142
H	-2.048994	-4.268090	-0.543867
H	-3.256746	-2.119771	2.487216
O	2.485261	0.273653	1.559454
H	2.318508	-0.628970	1.885300
H	2.201901	0.954125	2.211318
O	4.033067	2.783287	-0.726893
H	4.422437	1.923162	-0.387629
H	4.734119	3.311297	-1.110608
O	4.689740	0.498197	0.159216
H	3.923709	0.374574	0.761671
H	4.620885	-0.209131	-0.510490
O	4.205669	-1.389128	-1.704225
H	3.799770	-2.173530	-1.302030
H	4.919158	-1.691681	-2.268230
O	1.471135	2.346205	2.751978
H	1.627973	2.670643	3.639326
H	1.818883	3.020230	2.110236
O	2.395484	3.964515	0.949046
H	3.041897	3.543112	0.333167
H	2.782936	4.787717	1.248802
O	1.448314	-5.199831	-1.797282
H	2.232537	-4.735839	-1.468489
H	1.441775	-5.112491	-2.750592

MS6 + 6 WAT

N	-0.439981	2.122636	-0.963076
H	-0.220492	2.426709	-1.907103
H	-1.367860	1.675619	-0.995811
N	-0.260126	0.544659	1.344827
Pt	0.774631	0.619851	-0.419775
N	1.890193	0.762314	-2.119939
H	-1.241450	0.765210	1.165521
H	0.158718	1.242152	1.970146
H	2.458926	1.613469	-2.055550
H	1.365356	0.771671	-2.987014
H	-0.262092	-0.354356	1.838060
H	2.544752	-0.026351	-2.154616
C	-0.538417	3.300048	-0.102020
C	-1.678159	4.182585	-0.536942
H	-0.709354	2.978523	0.924968
H	0.414719	3.830561	-0.112512
H	-2.614648	3.624480	-0.435458
H	-1.560726	4.426112	-1.603108
N	-1.734918	5.340581	0.314997
H	-0.962493	5.966420	0.131340
H	-2.585970	5.862779	0.166415
C	-1.661598	-3.047029	-0.904432
C	-0.746297	-2.482250	0.154234
C	-1.382438	-2.702308	1.506004
C	-2.760779	-2.100122	1.500713
C	-3.580601	-2.508262	0.301880
C	-4.841539	-1.696709	0.182136
N	0.554564	-3.103487	0.037328
O	-0.671838	-2.072521	2.534448
O	-2.864967	-2.359289	-0.893375
O	-4.703443	-0.345201	0.574969
S	1.910740	-2.355473	0.490675
O	2.002980	-0.978553	-0.064627
O	1.928141	-2.274438	1.949762
O	2.988462	-3.142756	-0.100285
S	-4.096511	0.750049	-0.420388
O	-4.675071	1.982890	0.071747
O	-2.643822	0.696374	-0.216678
O	-4.502206	0.360085	-1.755431
H	-1.221098	-2.920639	-1.894098
H	-0.684631	-1.407120	-0.046479
H	-1.425535	-3.784862	1.685334
H	-2.652483	-1.014390	1.488367
H	-3.880229	-3.563182	0.392847
H	-5.207229	-1.742611	-0.844682
H	-5.599182	-2.095357	0.854540
H	0.731828	-3.670919	-0.803113
H	0.242010	-2.394883	2.564420
H	-1.823958	-4.122015	-0.732544
H	-3.287208	-2.370729	2.418442
O	3.207304	0.219404	2.405551
H	2.709133	-0.608765	2.363628
H	2.568508	0.948932	2.537176
O	3.614089	2.726956	-1.022176
H	4.024203	1.931445	-0.553831
H	4.314496	3.205922	-1.467698
O	4.494816	0.660917	0.130872
H	3.988426	0.455296	0.952204
H	4.361197	-0.072236	-0.497706
O	4.080533	-1.224713	-1.780421
H	3.793597	-2.011311	-1.286393
H	4.822064	-1.478887	-2.332091
O	1.529843	2.297141	2.624954
H	1.457234	2.706751	3.488091
H	1.867941	2.984670	1.991234
O	2.419789	4.007574	0.896941
H	2.898289	3.568692	0.148568
H	2.997337	4.686867	1.247266
O	1.670300	-4.698561	-1.923374
H	2.469574	-4.348571	-1.508350
H	1.741019	-4.531780	-2.863967

MS5' + 6 WAT

N	-1.754591	-1.879752	0.448279
H	-2.508327	-1.414622	-0.076794
H	-2.044844	-2.851340	0.528499
N	-0.760239	-0.392964	-1.870640
Pt	-0.017014	-1.791054	-0.572200
N	0.784340	-3.158726	0.708251
H	-1.782113	-0.340673	-1.876998
H	-0.423126	-0.555172	-2.819276
H	0.118125	-3.856584	1.021501
H	1.157549	-2.689595	1.549873
H	-0.395064	0.517705	-1.593838
H	1.541100	-3.673188	0.241826
C	-1.672705	-1.278701	1.781071
C	-2.933288	-1.462226	2.580160
H	-0.808448	-1.679283	2.312070
H	-1.500134	-0.210244	1.648294
H	-3.044934	-2.513093	2.862230
H	-3.790061	-1.185736	1.952399
N	-2.817000	-0.660989	3.773181
H	-2.780250	0.320815	3.519950
H	-3.609021	-0.797251	4.383987
C	-1.405415	2.869640	-1.255256
C	-2.269335	2.100365	-0.289089
C	-2.054422	2.623216	1.109795
C	-0.579908	2.527713	1.415574
C	0.273127	3.166210	0.349147
C	1.731088	2.900138	0.540277
N	-3.646084	2.138264	-0.714700
O	-2.743535	1.875460	2.062845
O	-0.061273	2.673756	-0.930862
O	1.916617	1.496466	0.435461
S	-4.465303	0.704991	-0.628630
O	-3.561300	-0.353249	-1.124143
O	-4.726103	0.526123	0.800347
O	-5.636969	0.900127	-1.453771
S	3.402010	0.991688	0.252805
O	4.129988	1.378157	1.456542
O	3.244461	-0.443347	0.113589
O	3.905513	1.667677	-0.939724
H	-1.548818	2.509659	-2.275198
H	-1.921376	1.062227	-0.288530
H	-2.382935	3.671883	1.137715
H	-0.309330	1.470833	1.497977
H	0.133291	4.254603	0.349817
H	2.314144	3.402466	-0.234494
H	2.060117	3.244037	1.522019
H	-3.804241	2.566763	-1.618639
H	-3.580197	1.533615	1.697675
H	-1.652256	3.938530	-1.231816
H	-0.366995	2.997386	2.377950
O	1.785597	-1.711747	-1.565790
H	1.712007	-1.235894	-2.452286
H	2.401803	-1.189509	-0.987568
O	2.594815	-4.239425	-1.158821
H	3.534332	-4.418964	-1.126668
H	2.477940	-3.359626	-1.551339
O	1.889167	1.973035	-2.735684
H	2.688265	1.902288	-2.183942
H	1.185137	2.307119	-2.154542
O	1.396708	-0.358285	-3.648665
H	1.782886	-0.555904	-4.503246
H	1.612495	0.585209	-3.396690
O	1.532809	-1.756568	3.000017
H	1.282001	-2.118219	3.850194
H	2.376469	-1.268247	3.137701
O	5.584294	3.515235	0.351564
H	5.172427	3.102296	-0.414734
H	5.284603	2.948315	1.070219
O	3.758684	-0.414493	3.424095
H	4.546936	-0.946602	3.535478
H	3.954959	0.248978	2.737406

TS3' + 6 WAT
(-155.93 cm^{-1})

N	-1.380903	-2.262402	-0.457035
H	-2.044449	-1.718868	-1.019855
H	-1.365030	-3.196988	-0.858153
N	-0.335806	0.174207	-1.514314
Pt	0.446889	-1.459547	-0.565832
N	1.242663	-3.083282	0.357500
H	-1.357721	0.150063	-1.559016
H	0.015447	0.230394	-2.474687
H	0.652922	-3.907962	0.359716
H	1.448625	-2.847830	1.342934
H	-0.069916	1.037797	-1.038168
H	2.109217	-3.339223	-0.134901
C	-1.947013	-2.300045	0.893552
C	-3.280172	-3.002624	0.903828
H	-1.241552	-2.775891	1.575098
H	-2.087818	-1.267369	1.213233
H	-3.132139	-4.081805	0.813594
H	-3.849172	-2.678182	0.020619
N	-3.947815	-2.711754	2.143250
H	-4.295200	-1.760456	2.101362
H	-4.736637	-3.325333	2.284913
C	-1.685264	3.089888	-0.910542
C	-2.468870	2.224825	0.041295
C	-2.376608	2.807973	1.425983
C	-0.910226	2.824236	1.786714
C	-0.017684	3.455444	0.740057
C	1.408706	3.039608	0.941586
N	-3.822446	2.006356	-0.404814
O	-3.072130	2.058287	2.368846
O	-0.329889	3.030676	-0.570857
O	1.400153	1.629037	0.761638
S	-4.233866	0.410911	-0.485428
O	-3.210118	-0.279620	-1.295040
O	-4.157506	-0.018186	0.915526
O	-5.551605	0.380340	-1.079148
S	2.758581	0.853060	0.621363
O	3.580234	1.202637	1.769535
O	2.331999	-0.532863	0.584047
O	3.352824	1.297471	-0.646239
H	-1.770443	2.720102	-1.933323
H	-1.959176	1.256121	0.085721
H	-2.785017	3.827262	1.403936
H	-0.611788	1.782547	1.933721
H	-0.080668	4.548379	0.781770
H	2.064330	3.504255	0.201935
H	1.758043	3.280823	1.945565
H	-4.095752	2.496500	-1.247618
H	-3.541875	1.321372	1.942552
H	-2.043869	4.125886	-0.885869
H	-0.758306	3.345803	2.733672
O	2.440658	-0.953627	-2.076905
H	2.078423	-0.545705	-2.892849
H	2.992760	-0.269245	-1.671189
O	3.281815	-3.389694	-1.525764
H	4.233178	-3.466729	-1.462244
H	3.079082	-2.492596	-1.859421
O	1.729673	2.663718	-2.390207
H	2.385856	2.282638	-1.784413
H	0.979014	2.945335	-1.843343
O	1.244322	0.574177	-3.840004
H	1.225515	0.624765	-4.795939
H	1.432893	1.468626	-3.462250
O	1.538683	-2.359143	3.019760
H	1.457339	-2.998556	3.726965
H	2.244008	-1.726950	3.280972
O	5.513902	2.764234	0.433510
H	4.979679	2.410146	-0.284357
H	5.096055	2.377091	1.209398
O	3.397575	-0.627108	3.744483
H	4.258149	-0.992474	3.951645
H	3.531460	0.056984	3.065782

MS6' + 6 WAT

N	-1.425614	-2.192233	-0.741033
H	-2.011174	-1.567051	-1.302068
H	-1.320849	-3.049152	-1.279065
N	-0.277678	0.325380	-1.360564
Pt	0.365144	-1.363619	-0.406798
N	1.109655	-3.079799	0.385223
H	-1.293145	0.340481	-1.484336
H	0.149509	0.379829	-2.293048
H	0.517660	-3.897118	0.292458
H	1.342455	-2.973488	1.386057
H	-0.020688	1.175165	-0.857470
H	1.960481	-3.251676	-0.166254
C	-2.191174	-2.449487	0.481878
C	-3.533532	-3.060872	0.175123
H	-1.611432	-3.086829	1.149429
H	-2.347945	-1.489109	0.972623
H	-3.412799	-4.110390	-0.104690
H	-3.959658	-2.540269	-0.694697
N	-4.356390	-2.969211	1.350931
H	-4.631220	-2.000844	1.474221
H	-5.194141	-3.523837	1.254488
C	-1.625231	3.233522	-0.553614
C	-2.415654	2.259098	0.278166
C	-2.293490	2.636904	1.729490
C	-0.821255	2.568193	2.064690
C	0.074480	3.324159	1.105855
C	1.493522	2.850233	1.214839
N	-3.776139	2.133329	-0.181497
O	-2.991326	1.771177	2.565155
O	-0.266558	3.103217	-0.246751
O	1.442259	1.479044	0.823548
S	-4.220007	0.571635	-0.482899
O	-3.190645	-0.032390	-1.350123
O	-4.193996	-0.038968	0.851753
O	-5.522952	0.654826	-1.104670
S	2.749977	0.711184	0.448630
O	3.636763	0.725291	1.593869
O	2.247752	-0.628025	0.128912
O	3.329791	1.384179	-0.711810
H	-1.730037	3.011334	-1.616516
H	-1.929274	1.283070	0.181913
H	-2.677969	3.657029	1.862159
H	-0.548881	1.508877	2.055526
H	0.044142	4.400156	1.309391
H	2.149024	3.400432	0.537255
H	1.866403	2.924092	2.235981
H	-4.038302	2.737870	-0.950469
H	-3.502627	1.127959	2.045259
H	-1.962713	4.262350	-0.379065
H	-0.644219	2.942627	3.074648
O	3.285732	-0.581274	-2.755979
H	2.529592	-0.171434	-3.217666
H	3.522680	0.061426	-2.076569
O	3.037655	-2.988401	-1.649030
H	3.789178	-3.489299	-1.963770
H	3.102244	-2.089885	-2.044188
O	1.663482	2.954625	-2.227760
H	2.359744	2.535626	-1.699399
H	0.953759	3.183017	-1.607040
O	1.218741	0.859330	-3.700476
H	0.961636	0.950345	-4.618597
H	1.351744	1.757791	-3.312779
O	1.599752	-2.678601	3.090638
H	1.572399	-3.407991	3.709361
H	2.406286	-2.152357	3.289435
O	5.704987	2.342966	0.517074
H	5.101964	2.235201	-0.224205
H	5.276602	1.833395	1.211387
O	3.699027	-1.164060	3.507695
H	4.557663	-1.588426	3.503418
H	3.714636	-0.477994	2.819095

Direct Substitution

IdoA(2S)-model (Direct substitution)

Msa + 6 WAT

N	0.300879	-1.163533	1.066045
H	1.167235	-0.689410	0.803683
H	0.139566	-0.989458	2.060951
N	-2.476534	-0.535609	1.541015
Pt	-1.209061	-0.422107	-0.050194
N	0.120634	-0.214359	-1.597141
H	-2.031368	-0.939228	2.365190
H	-3.314714	-1.093197	1.336903
H	-0.326929	-0.271654	-2.517791
H	0.877388	-0.900154	-1.571734
H	-2.741600	0.432291	1.790297
H	0.543782	0.713725	-1.509730
C	0.507173	-2.599577	0.888225
C	1.773801	-3.065345	1.565911
H	-0.369843	-3.128630	1.263107
H	0.592639	-2.799595	-0.178139
H	1.589557	-3.257220	2.625824
H	2.518988	-2.259127	1.510598
N	2.221411	-4.275523	0.928909
H	2.570520	-4.035646	0.007291
H	2.980624	-4.698291	1.443178
C	2.816970	2.027398	-1.276185
C	3.241530	1.229854	-0.071061
C	2.643035	1.820583	1.182673
C	2.350768	3.280662	0.969801
C	1.332470	3.434947	-0.139962
C	-0.108971	3.310114	0.333510
O	2.732240	-0.092507	-0.168066
O	3.527883	1.578849	2.233317
O	1.485841	2.425887	-1.123253
O	-0.312229	2.877590	1.479885
O	-0.972664	3.645948	-0.498686
S	3.585565	-1.212703	-0.962236
O	2.520143	-2.147069	-1.302469
O	4.174629	-0.523376	-2.093967
O	4.547022	-1.726951	-0.009086
H	2.886289	1.410131	-2.169666
H	4.330328	1.201581	0.022546
H	1.684903	1.321772	1.366180
H	3.277619	3.809944	0.735043
H	1.442756	4.409947	-0.626645
H	3.106568	1.799867	3.065253
H	1.939831	3.710724	1.883133
H	3.467946	2.897556	-1.410217
Cl	-2.997217	0.309013	-1.396471
O	-1.440865	-0.278548	-3.988171
H	-2.167073	0.045628	-3.439171
H	-1.315021	0.357246	-4.693320
O	-4.658661	-2.272788	0.937136
H	-4.437258	-2.550596	0.024169
H	-4.704417	-3.062763	1.475007
O	-3.502825	3.219402	-0.243415
H	-3.621419	2.391218	-0.720275
H	-2.531293	3.417866	-0.336782
O	-2.782603	2.200356	2.130920
H	-3.254711	2.583311	1.367182
H	-1.847253	2.455669	1.949671
O	-0.685544	-1.353356	3.712907
H	-0.662379	-2.216029	4.131819
H	-0.602704	-0.707232	4.417181
O	-3.853325	-2.671419	-1.539300
H	-4.468789	-2.893159	-2.239402
H	-3.567071	-1.757495	-1.691696

TSa + 6 WAT
(-130.40 cm^{-1})

N	-0.500714	1.274367	0.959087
H	-1.348821	0.719350	0.826576
H	-0.308724	1.267704	1.965818
N	2.112554	0.282802	1.644920
Pt	0.945076	0.270445	-0.026506
N	-0.247069	0.175017	-1.678317
H	1.557821	0.121532	2.484205
H	2.538692	1.208760	1.769648
H	0.219103	0.516523	-2.525347
H	-1.145147	0.650022	-1.591704
H	2.842707	-0.442909	1.600354
H	-0.429139	-0.828207	-1.775937
C	-0.773900	2.630561	0.493664
C	-2.012905	3.192804	1.150441
H	0.108701	3.249293	0.656301
H	-0.947821	2.587324	-0.580554
H	-1.761171	3.652948	2.108837
H	-2.702095	2.362103	1.362741
N	-2.595849	4.181714	0.281691
H	-3.009980	3.699007	-0.508302
H	-3.331559	4.690852	0.749875
C	-2.345244	-2.542251	-1.152891
C	-2.740444	-1.823230	0.110591
C	-1.814240	-2.200188	1.245182
C	-1.149641	-3.516436	0.946690
C	-0.314526	-3.376164	-0.312541
C	1.066231	-2.815998	-0.013661
O	-2.605125	-0.420737	-0.058041
O	-2.562898	-2.213589	2.421149
O	-0.952283	-2.576859	-1.274254
O	1.654833	-3.314333	0.952053
O	1.538631	-1.939501	-0.783876
S	-3.798820	0.445520	-0.718068
O	-3.027625	1.560225	-1.253301
O	-4.403110	-0.415454	-1.716296
O	-4.678685	0.801976	0.376329
H	-2.739561	-2.010641	-2.016369
H	-3.769398	-2.066279	0.388852
H	-1.033402	-1.427882	1.294276
H	-1.907333	-4.295327	0.836384
H	-0.137447	-4.372939	-0.736832
H	-1.981714	-2.340661	3.172597
H	-0.486220	-3.809935	1.759859
H	-2.763505	-3.553897	-1.157086
Cl	3.161841	0.870590	-1.476568
O	1.312091	1.168151	-3.858196
H	2.075806	1.085232	-3.261269
H	1.495060	0.626289	-4.625999
O	2.838276	3.012399	2.005471
H	2.622724	3.365948	1.115322
H	2.277392	3.460257	2.638236
O	4.151892	-1.997451	-1.154036
H	4.290052	-1.083516	-1.428503
H	3.171346	-2.032022	-1.058238
O	3.858463	-1.966109	1.524986
H	4.226080	-1.933206	0.623345
H	3.058095	-2.523834	1.400063
O	0.138203	0.782641	3.706522
H	0.429472	1.454701	4.325881
H	-0.493465	0.235575	4.177799
O	2.294945	3.638537	-0.485867
H	2.825374	4.309152	-0.918484
H	2.536654	2.784615	-0.891815

MSb + 6 WAT

N	0.903027	1.392196	0.824918
H	0.078689	1.999202	0.802968
H	1.174047	1.215114	1.795863
N	0.801808	-1.411178	1.624762
Pt	0.395547	-0.336742	-0.067215
N	-0.059931	0.696384	-1.758023
H	-0.025282	-1.490865	2.228947
H	1.532290	-0.958595	2.180058
H	0.762337	0.794998	-2.366087
H	-0.422516	1.623991	-1.542047
H	1.095691	-2.370733	1.397011
H	-0.792346	0.158084	-2.217744
C	2.007769	2.114343	0.200903
C	2.273789	3.409881	0.917407
H	2.896501	1.483488	0.215575
H	1.763617	2.301370	-0.846906
H	2.583567	3.186687	1.942073
H	1.334511	3.978193	0.977025
N	3.339484	4.117063	0.256272
H	3.040685	4.456731	-0.647936
H	3.632741	4.919942	0.793162
C	-3.425705	-0.071219	-1.498708
C	-3.372112	0.391313	-0.065194
C	-3.036646	-0.776681	0.842340
C	-3.342237	-2.086690	0.171471
C	-2.507576	-2.198589	-1.096722
C	-1.136630	-2.768340	-0.792755
O	-2.313072	1.307393	0.131538
O	-3.611800	-0.656451	2.111437
O	-2.385414	-0.972004	-1.754885
O	-1.097206	-3.910400	-0.360015
O	-0.092701	-2.075864	-1.041675
S	-2.460490	2.860174	-0.223556
O	-1.045683	3.225223	-0.256466
O	-3.118747	2.926900	-1.515997
O	-3.212770	3.465170	0.856759
H	-3.295753	0.779014	-2.164826
H	-4.324089	0.844747	0.229203
H	-1.956846	-0.714337	1.011424
H	-4.407214	-2.156332	-0.065653
H	-2.975348	-2.929323	-1.766300
H	-4.537777	-0.910177	2.098867
H	-3.098516	-2.918813	0.832298
H	-4.398307	-0.526958	-1.709856
Cl	4.319709	-0.683018	-1.186284
O	2.467858	1.050308	-2.922028
H	3.078004	0.514317	-2.380978
H	2.715861	0.906242	-3.835553
O	2.527539	0.474883	2.900213
H	3.413698	0.500580	2.465424
H	2.653108	0.613107	3.838774
O	2.370829	-3.090812	-1.413813
H	2.997821	-2.352896	-1.331764
H	1.480566	-2.692936	-1.405482
O	1.345390	-4.135022	0.827180
H	1.917779	-3.878861	0.079415
H	0.456764	-4.190551	0.428765
O	-1.513613	-1.759006	3.293701
H	-1.406020	-1.732044	4.244180
H	-2.382397	-1.370704	3.085177
O	4.757258	0.536283	1.554563
H	5.555254	0.184562	1.951047
H	4.661467	0.109952	0.678280

MSa' + 6 WAT

N	0.735467	-1.320410	0.770762
H	1.490114	-1.188964	0.074969
H	0.898400	-0.657714	1.530935
N	-1.789444	-0.483316	1.804334
Pt	-1.071970	-0.983065	-0.043042
N	-0.302642	-1.317871	-1.905445
H	-1.106073	-0.619853	2.550171
H	-2.659774	-0.973463	2.028641
H	-0.839416	-2.025299	-2.413637
H	0.690059	-1.575732	-1.885492
H	-1.963261	0.522555	1.778310
H	-0.382074	-0.424847	-2.397368
C	0.889313	-2.658838	1.324725
C	2.257548	-2.836876	1.927914
H	0.121981	-2.832823	2.081651
H	0.733494	-3.384479	0.523139
H	2.371442	-2.129833	2.754244
H	3.007431	-2.568093	1.173175
N	2.388493	-4.176409	2.442239
H	2.430341	-4.848397	1.687787
H	3.238917	-4.279666	2.976422
C	3.017730	1.329177	0.939397
C	2.101554	2.117186	0.032794
C	2.288847	1.679251	-1.401667
C	3.730054	1.297121	-1.589251
C	3.981415	0.036157	-0.772556
C	3.554024	-1.171395	-1.595944
O	0.724024	1.884537	0.342061
O	1.881691	2.650983	-2.303730
O	3.249251	0.054539	0.423801
O	4.335578	-1.523053	-2.482821
O	2.437653	-1.677683	-1.339679
S	0.018375	2.841313	1.408770
O	-1.366295	2.379515	1.335099
O	0.649829	2.543600	2.690917
O	0.221735	4.198367	0.961269
H	2.564929	1.207827	1.922894
H	2.294695	3.190737	0.107243
H	1.687743	0.771267	-1.537542
H	4.384753	2.116034	-1.277997
H	5.048586	-0.067552	-0.553028
H	0.931503	2.496195	-2.462563
H	3.935638	1.097265	-2.640477
H	3.959986	1.873056	1.069284
Cl	-3.235506	-0.717407	-0.968816
O	-2.359580	-3.070755	-2.803763
H	-2.924678	-2.441652	-2.334651
H	-2.737570	-3.189728	-3.675353
O	-4.237153	-1.921937	1.710918
H	-5.089980	-1.689693	2.079029
H	-4.241856	-1.638005	0.786598
O	-0.535850	1.612145	-2.193500
H	-1.479417	1.868787	-2.334626
H	-0.429561	1.541328	-1.234800
O	-3.087850	2.146231	-2.289445
H	-3.193343	2.760152	-1.527447
H	-3.370501	1.279311	-1.970301
O	-3.187584	3.633065	-0.127705
H	-3.023945	4.575167	-0.178124
H	-2.493207	3.245531	0.442302
O	0.607456	-0.026671	3.302916
H	0.629046	0.949329	3.230548
H	1.029630	-0.272492	4.126471

TSa' + 6 WAT
(-122.81 cm⁻¹)

N	-0.984849	1.850225	0.385376
H	-1.735538	1.390398	-0.164312
H	-1.282426	1.835887	1.362185
N	1.097561	0.827807	2.149016
Pt	0.647795	0.715777	0.156633
N	0.123675	0.585574	-1.810865
H	0.431861	1.392812	2.677118
H	2.043079	1.196686	2.304166
H	0.477630	1.412659	-2.297891
H	-0.896297	0.550852	-1.910889
H	1.048668	-0.120423	2.524646
H	0.539823	-0.246073	-2.246571
C	-0.834559	3.235957	-0.034339
C	-2.153696	3.959740	0.028245
H	-0.103996	3.728431	0.610133
H	-0.428934	3.255463	-1.048989
H	-2.506290	3.970138	1.063675
H	-2.889624	3.391219	-0.556875
N	-1.980796	5.320058	-0.411855
H	-1.781890	5.354583	-1.402596
H	-2.818483	5.862276	-0.258782
C	-2.325382	-1.353971	1.550062
C	-1.833620	-2.513768	0.722840
C	-2.734975	-2.743467	-0.469649
C	-4.069723	-2.088079	-0.244030
C	-3.866352	-0.596471	-0.074138
C	-3.672417	0.092518	-1.422926
O	-0.547934	-2.257536	0.165726
O	-2.823457	-4.119887	-0.677267
O	-2.769730	-0.305578	0.750483
O	-4.526487	-0.149564	-2.279868
O	-2.691624	0.860734	-1.545715
S	0.771990	-2.441217	1.015351
O	1.670585	-1.503605	0.294339
O	0.494704	-2.017321	2.373783
O	1.209335	-3.810185	0.876187
H	-1.516778	-0.972046	2.169343
H	-1.797933	-3.424293	1.327236
H	-2.247703	-2.260331	-1.326395
H	-4.554008	-2.529159	0.630512
H	-4.772016	-0.160090	0.364536
H	-3.273583	-4.293029	-1.505456
H	-4.723880	-2.240803	-1.102246
H	-3.119894	-1.710794	2.215791
Cl	3.274116	0.945305	-0.735484
O	1.602372	2.948738	-2.386881
H	2.261091	2.452273	-1.871095
H	2.044790	3.252151	-3.179860
O	3.743577	1.840193	2.128046
H	4.472682	1.412391	2.577380
H	3.794529	1.579501	1.191449
O	1.467550	-1.823989	-2.471350
H	2.400935	-1.688458	-2.726976
H	1.500974	-1.838350	-1.504335
O	4.121288	-1.393949	-2.609094
H	4.245290	-1.990487	-1.844462
H	4.027475	-0.521783	-2.197170
O	4.018070	-2.696481	-0.279378
H	3.743165	-3.612800	-0.212217
H	3.247420	-2.180474	0.020113
O	-1.238728	2.190616	3.221394
H	-1.808227	1.682470	3.802653
H	-1.242667	3.090002	3.555110

MSb' + 6 WAT

N	1.520938	1.769991	-0.343213
H	2.209017	1.118097	0.073356
H	1.832167	1.994595	-1.290514
N	-0.643658	1.313940	-2.333116
Pt	-0.183318	0.721773	-0.434255
N	0.242380	0.139388	1.469129
H	0.090649	1.854177	-2.791252
H	-1.497909	1.880476	-2.257898
H	-0.075486	0.872448	2.112570
H	1.259408	0.031382	1.566683
H	-0.847552	0.492788	-2.898295
H	-0.207290	-0.738752	1.755770
C	1.482735	3.002974	0.432448
C	2.861538	3.586724	0.584992
H	0.824749	3.716408	-0.067337
H	1.041084	2.794061	1.410078
H	3.253419	3.841728	-0.403833
H	3.520237	2.813560	1.004129
N	2.794307	4.788644	1.375474
H	2.561465	4.574325	2.335802
H	3.682404	5.268501	1.385961
C	1.619320	-1.872203	-1.611118
C	0.814760	-2.883685	-0.826853
C	1.554819	-3.236270	0.439218
C	3.038494	-3.136698	0.203717
C	3.393960	-1.688879	-0.098890
C	3.714924	-0.901620	1.166328
O	-0.462836	-2.432554	-0.351606
O	1.131233	-4.503027	0.836045
O	2.352593	-1.040284	-0.773105
O	4.588717	-1.384524	1.891093
O	3.091827	0.167575	1.358526
S	-1.622674	-1.809391	-1.223062
O	-1.850717	-0.492807	-0.562087
O	-1.163400	-1.701541	-2.586334
O	-2.784612	-2.643105	-1.002183
H	0.977690	-1.247403	-2.224999
H	0.650465	-3.783538	-1.423264
H	1.267546	-2.490521	1.191486
H	3.322338	-3.800748	-0.616694
H	4.301984	-1.662645	-0.715200
H	1.438653	-4.684198	1.725565
H	3.590735	-3.452506	1.088976
H	2.282942	-2.428205	-2.284198
Cl	-3.612278	1.748837	1.386237
O	-0.927829	2.388474	2.740774
H	-1.803846	2.256308	2.332664
H	-1.082196	2.548069	3.672182
O	-2.892402	2.738983	-1.465461
H	-3.736900	2.941317	-1.868925
H	-3.086582	2.391476	-0.575705
O	-1.006894	-2.273779	2.408328
H	-1.898137	-1.894927	2.583509
H	-1.038861	-2.705310	1.549834
O	-3.342916	-1.108684	2.838619
H	-3.887847	-1.307755	2.046684
H	-3.216353	-0.153896	2.759801
O	-4.549551	-1.146096	0.486724
H	-3.997998	-1.698449	-0.089812
H	-4.193868	-0.246631	0.416006
O	1.778293	2.735651	-3.034214
H	2.384761	2.397295	-3.696252
H	1.776273	3.690139	-3.129984

GlcNS(6S)-model (Direct substitution)

MSc + 6 WAT

N	0.680337	-1.673268	-1.241868
H	0.394450	-1.629920	-2.215592
H	1.471225	-1.029303	-1.135868
N	0.521078	-0.851211	1.513160
Pt	-0.811394	-1.085537	-0.005234
N	-2.159088	-1.333338	-1.522073
H	1.492884	-0.887716	1.200795
H	0.339068	-1.600102	2.187026
H	-2.534357	-2.287002	-1.501747
H	-1.766029	-1.172761	-2.442805
H	0.358194	0.064889	1.945894
H	-2.959017	-0.697848	-1.437490
C	1.184066	-3.014659	-0.948445
C	2.264206	-3.412614	-1.916838
H	1.587435	-3.005347	0.064783
H	0.349051	-3.718325	-0.957190
H	3.098121	-2.712740	-1.815771
H	1.882181	-3.311851	-2.943555
N	2.730466	-4.736821	-1.603733
H	2.031176	-5.431652	-1.827286
H	3.560399	-4.966901	-2.130104
C	1.578223	3.241557	-0.994003
C	0.462262	2.768315	-0.086379
C	0.927767	2.754733	1.359823
C	2.309397	2.168843	1.491832
C	3.271396	2.711858	0.473192
C	4.629669	2.071452	0.549319
N	-0.624024	3.702835	-0.298646
O	0.093191	1.983558	2.176824
O	2.749937	2.525875	-0.813736
O	4.624900	0.725399	0.979422
S	-2.172546	3.262520	-0.003756
O	-2.482408	2.069117	-0.801987
O	-2.247016	2.999218	1.432094
O	-2.952150	4.426409	-0.427280
S	4.342029	-0.465463	-0.048443
O	5.013849	-1.586358	0.573663
O	2.884173	-0.622410	-0.063351
O	4.882474	-0.044498	-1.325451
H	1.278477	3.128650	-2.036790
H	0.178996	1.743948	-0.368986
H	0.932659	3.799995	1.699628
H	2.253266	1.091955	1.336841
H	3.422041	3.792308	0.623049
H	5.122756	2.143884	-0.421423
H	5.230391	2.586794	1.296582
H	-0.592411	4.207872	-1.187347
H	-0.831589	2.270947	2.051386
H	1.757834	4.311571	-0.805071
H	2.680131	2.340791	2.504949
O	-3.500595	-3.714538	-0.762338
H	-4.146936	-3.063709	-0.377366
H	-3.988829	-4.371315	-1.259822
O	-4.904115	-1.813752	0.272756
H	-4.272766	-1.557402	0.962248
H	-4.909998	-1.022179	-0.300049
O	-4.426899	0.407377	-1.149464
H	-3.779951	1.111715	-0.901888
H	-4.917712	0.721817	-1.909422
O	-0.885838	-3.027721	2.775976
H	-1.569234	-2.351889	2.697400
H	-1.029385	-3.605029	1.997976
O	-1.381451	-4.551564	0.612150
H	-2.189469	-4.267022	0.133662
H	-1.480965	-5.486477	0.792089
O	-1.456604	5.448543	-2.387725
H	-2.214970	5.222109	-1.820750
H	-1.714336	5.279841	-3.293473
Cl	-2.461212	-0.530301	1.565155

TSb + 6 WAT
(-118.95 cm⁻¹)

N	-0.374558	1.921856	-1.116752
H	-0.075623	2.033128	-2.080916
H	-1.272733	1.420321	-1.139214
N	-0.458811	0.705718	1.400649
Pt	0.893155	0.722859	-0.113996
N	2.243467	0.754699	-1.646310
H	-1.412083	0.782003	1.039188
H	-0.250592	1.510365	1.995903
H	2.905371	1.530486	-1.541415
H	1.820374	0.830594	-2.564594
H	-0.393405	-0.165082	1.937810
H	2.780960	-0.117844	-1.646805
C	-0.617362	3.244423	-0.540171
C	-1.729336	3.953295	-1.266763
H	-0.883757	3.124189	0.510105
H	0.314794	3.812100	-0.568507
H	-2.659687	3.396960	-1.119744
H	-1.514832	3.954989	-2.345764
N	-1.891835	5.271051	-0.711028
H	-1.114289	5.868758	-0.955796
H	-2.730887	5.713167	-1.056277
C	-2.028892	-3.042913	-0.919482
C	-0.930856	-2.538784	-0.012220
C	-1.408175	-2.659430	1.419622
C	-2.747295	-1.983878	1.562117
C	-3.739586	-2.372704	0.496483
C	-4.960841	-1.497413	0.515018
N	0.237053	-3.344010	-0.303337
O	-0.555378	-2.036926	2.339551
O	-3.179360	-2.286872	-0.781595
O	-4.692242	-0.131084	0.762606
S	1.696427	-2.865503	0.262235
O	1.877377	-1.431359	0.044657
O	1.729992	-3.223211	1.677605
O	2.663349	-3.620762	-0.555414
S	-4.147693	0.823517	-0.400259
O	-4.567969	2.136720	0.043124
O	-2.688953	0.667603	-0.371834
O	-4.755245	0.360732	-1.630935
H	-1.717612	-2.973831	-1.963035
H	-0.753761	-1.478372	-0.238302
H	-1.490371	-3.730865	1.649492
H	-2.592822	-0.906812	1.501434
H	-4.080503	-3.407213	0.657282
H	-5.493204	-1.596836	-0.432086
H	-5.615273	-1.796058	1.332089
H	0.334975	-3.580893	-1.295449
H	0.309949	-2.484730	2.332877
H	-2.229430	-4.101375	-0.692992
H	-3.158945	-2.201328	2.549998
O	4.193996	2.605901	-0.749524
H	4.573850	1.752796	-0.398419
H	4.873979	3.058236	-1.249633
O	4.867385	0.290676	0.138903
H	4.183302	0.237420	0.833420
H	4.677975	-0.433258	-0.489689
O	4.117881	-1.567692	-1.651508
H	3.671957	-2.353214	-1.281168
H	4.729940	-1.866709	-2.325224
O	0.907114	3.114721	2.431583
H	1.523087	2.378855	2.574461
H	1.311676	3.570760	1.670645
O	2.341132	4.185373	0.358243
H	3.040443	3.592288	0.010936
H	2.766188	5.017819	0.566210
O	1.264604	-4.323506	-2.750793
H	2.016525	-4.181437	-2.154253
H	1.453692	-3.853769	-3.563068
Cl	2.600134	0.601758	2.100765

MSd + 6 WAT

N	-0.257548	1.857151	-1.186241
H	-0.079152	1.976363	-2.179183
H	-1.241580	1.567665	-1.090401
N	-0.189186	0.567635	1.319505
Pt	0.777173	0.300794	-0.451796
N	1.841559	0.139771	-2.187265
H	-1.143202	0.894581	1.157991
H	0.349249	1.270671	1.851494
H	2.368841	1.012822	-2.287622
H	1.280396	-0.012494	-3.017765
H	-0.260139	-0.298543	1.862802
H	2.537372	-0.611907	-2.131846
C	-0.102492	3.148348	-0.519277
C	-1.164780	4.116433	-0.966544
H	-0.171463	3.003758	0.558811
H	0.896227	3.533402	-0.723289
H	-2.143815	3.728161	-0.668811
H	-1.159443	4.176460	-2.064822
N	-0.954506	5.383908	-0.319003
H	-0.124161	5.840057	-0.671738
H	-1.731020	6.008980	-0.477027
C	-2.368000	-2.735877	-0.886011
C	-1.249805	-2.307240	0.034204
C	-1.710629	-2.526640	1.456029
C	-2.991266	-1.757861	1.651679
C	-4.018824	-1.983704	0.569227
C	-5.129896	-0.972137	0.636344
N	-0.070648	-3.051537	-0.351003
O	-0.811652	-2.035998	2.411229
O	-3.463554	-1.907305	-0.713178
O	-4.706330	0.333139	0.978824
S	1.389456	-2.796878	0.306515
O	1.737699	-1.342144	0.344381
O	1.380843	-3.318788	1.661716
O	2.309241	-3.496337	-0.595958
S	-4.054258	1.307085	-0.110606
O	-4.303115	2.618982	0.450242
O	-2.626277	0.970002	-0.128274
O	-4.727459	1.030237	-1.363388
H	-2.057757	-2.650600	-1.928776
H	-1.108219	-1.231897	-0.129122
H	-1.866400	-3.603466	1.605759
H	-2.735183	-0.697482	1.667587
H	-4.475212	-2.977973	0.688234
H	-5.654676	-0.945581	-0.319943
H	-5.828832	-1.243774	1.425421
H	0.044313	-3.144640	-1.368133
H	-0.010446	-2.585560	2.415915
H	-2.633256	-3.786083	-0.692430
H	-3.422194	-2.007818	2.623260
O	3.475012	2.350524	-1.397514
H	3.926178	1.616758	-0.879238
H	4.131244	2.738570	-1.978779
O	4.530466	0.449977	-0.088148
H	4.309572	0.413185	0.862839
H	4.320104	-0.409500	-0.500361
O	3.959790	-1.684844	-1.583934
H	3.486594	-2.449022	-1.199468
H	4.667499	-2.016675	-2.138271
O	1.598412	2.424973	2.414200
H	2.334277	1.836905	2.661838
H	1.976416	3.021082	1.736181
O	2.752001	3.973848	0.546183
H	3.060198	3.410965	-0.201811
H	3.516978	4.441707	0.882087
O	0.865789	-3.643005	-2.909629
H	1.664327	-3.723014	-2.370806
H	1.075801	-3.068545	-3.646478
Cl	3.998670	0.552630	2.927738

MSc' + 6 WAT

N	-2.24910300	-1.07957700	0.36825900
H	-1.89827200	-0.53060500	1.16167300
H	-2.88540400	-0.42421800	-0.09957800
N	-1.50520200	-0.25708600	-2.32532200
Pt	-0.73442300	-1.51548600	-0.91455600
N	0.09535000	-2.75682700	0.47222600
H	-2.32842200	0.26459000	-2.01840600
H	-1.76271300	-0.78589700	-3.15155900
H	-0.43149100	-3.61832500	0.56851700
H	0.18413400	-2.32826800	1.40596000
H	-0.79466100	0.43250300	-2.61280200
H	1.04957600	-3.02057900	0.20371300
C	-3.03948900	-2.19557200	0.87045300
C	-4.11826700	-1.71266900	1.80504700
H	-3.48310000	-2.72729200	0.02838000
H	-2.38306700	-2.89426400	1.39332800
H	-4.76815400	-1.01628300	1.26853500
H	-3.65129600	-1.13638100	2.61695100
N	-4.90015400	-2.83043900	2.26252600
H	-4.36965300	-3.40947300	2.89915100
H	-5.72373800	-2.52168000	2.75767800
C	-0.25522400	3.35416800	-0.12626600
C	-1.33527300	2.38466900	0.30037700
C	-1.09432100	2.00369600	1.74731600
C	0.29765200	1.45079900	1.86894300
C	1.31831600	2.42842400	1.36285800
C	2.70240700	1.86815100	1.33612900
N	-2.59814500	3.05689200	0.08716500
O	-1.96429100	1.02097600	2.22056000
O	1.01564500	2.81462400	0.04776000
O	2.68929700	0.73205600	0.48623900
S	-3.97718200	2.16086500	-0.07715800
O	-3.66908000	1.00442100	-0.93679100
O	-4.31470300	1.70967600	1.27673600
O	-4.92899800	3.08216300	-0.66037900
S	4.11011400	0.21242600	0.01724800
O	4.85072300	-0.06381000	1.24699400
O	3.79614600	-0.95509300	-0.77648300
O	4.69671600	1.30366500	-0.75696100
H	-0.35394100	3.57249600	-1.19073900
H	-1.24830500	1.48113900	-0.31948800
H	-1.19534500	2.91795900	2.34998900
H	0.36395200	0.52120700	1.29433300
H	1.33760200	3.32220200	2.00163300
H	3.40097100	2.60949400	0.94015000
H	3.01622600	1.57239900	2.33849700
H	-2.57127400	3.72310400	-0.67631500
H	-2.89249300	1.29240700	2.07260500
H	-0.35264200	4.28621300	0.44624200
H	0.50355600	1.19368000	2.90913900
O	2.92348200	-3.21756400	0.25307800
H	3.23004800	-2.44684000	-0.26789700
H	3.33537900	-4.00241800	-0.10991900
O	2.58219800	2.53741800	-2.08092600
H	3.39058500	2.06772300	-1.82875100
H	2.10185500	2.69361000	-1.24712600
O	0.25407600	1.81829500	-3.06610200
H	0.14363600	2.13213900	-3.96358100
H	1.19106600	1.97681500	-2.81859100
O	0.78895400	-1.56321900	2.93678300
H	0.51768800	-1.96303500	3.76290200
H	1.76890600	-1.65673900	2.87987300
O	6.49207700	2.20033500	1.18486500
H	6.04962900	2.21354900	0.32849400
H	6.13845400	1.39233900	1.57340500
O	3.35739200	-1.98907300	2.57820900
H	3.27919200	-2.58789300	1.81011700
H	3.93570600	-1.26973600	2.28065800
Cl	0.94134200	-2.05824300	-2.43323600

TSb' + 6 WAT
(-109.50 cm⁻¹)

N	-1.148448	-2.092486	-0.140191
H	-1.927888	-1.651319	-0.648459
H	-1.154326	-3.066780	-0.431274
N	-0.308914	0.316169	-1.548852
Pt	0.619264	-1.260047	-0.648540
N	1.549733	-2.842082	0.236492
H	-1.290029	0.128465	-1.761725
H	0.137922	0.605881	-2.429469
H	1.199776	-3.734208	-0.095101
H	1.472539	-2.820536	1.263770
H	-0.276788	1.130142	-0.932645
H	2.549931	-2.818967	0.017392
C	-1.422124	-2.005218	1.296233
C	-2.659194	-2.761533	1.698285
H	-0.553861	-2.350501	1.858867
H	-1.568892	-0.951686	1.536241
H	-2.474926	-3.837753	1.635934
H	-3.467211	-2.511532	0.999932
N	-2.959176	-2.410655	3.064496
H	-3.179306	-1.422193	3.123125
H	-3.753964	-2.928490	3.409676
C	-2.238192	2.953956	-0.331448
C	-2.859951	1.687000	0.190331
C	-2.874695	1.734574	1.696561
C	-1.440234	1.884248	2.146060
C	-0.678365	2.987791	1.445593
C	0.802558	2.805842	1.595485
N	-4.150421	1.462647	-0.409485
O	-3.376492	0.562939	2.262568
O	-0.897612	2.987397	0.052556
O	1.102012	1.582197	0.938645
S	-4.439123	-0.097446	-0.886109
O	-3.189039	-0.618928	-1.472590
O	-4.757025	-0.797031	0.360013
O	-5.529319	0.003018	-1.832570
S	2.593403	1.298738	0.508345
O	3.381551	1.206154	1.731832
O	2.485461	0.044176	-0.218724
O	3.000452	2.433543	-0.315362
H	-2.254438	2.972430	-1.422290
H	-2.193861	0.865613	-0.088733
H	-3.477924	2.597420	2.011844
H	-0.938146	0.933437	1.948062
H	-0.956805	3.968445	1.848384
H	1.343198	3.621502	1.110997
H	1.092325	2.742817	2.644887
H	-4.376570	2.090977	-1.170877
H	-4.042550	0.153449	1.680867
H	-2.769958	3.838454	0.040399
H	-1.397502	2.055654	3.223290
O	4.271083	-2.209336	-0.423844
H	3.821178	-1.834484	-1.211794
H	4.903425	-2.857408	-0.736722
O	1.033098	3.562799	-1.793447
H	1.817717	3.215765	-1.335554
H	0.310171	3.502982	-1.148614
O	0.678340	1.824046	-3.726604
H	0.078461	2.109087	-4.415009
H	0.783870	2.568640	-3.092582
O	1.733480	-2.359550	2.959869
H	1.816914	-3.011082	3.655704
H	2.639365	-2.019653	2.753348
O	4.773976	3.630458	1.493303
H	4.298406	3.551721	0.659219
H	4.514494	2.819416	1.943379
O	4.011614	-1.397215	2.131829
H	4.164976	-1.673036	1.208177
H	3.862741	-0.438745	2.088753
Cl	2.473269	-1.580789	-2.772396

MSd' + 6 WAT

N	-0.968142	-2.203407	-0.758021
H	-1.606834	-1.693579	-1.377694
H	-0.629828	-3.003016	-1.288845
N	-0.220670	0.514723	-1.282298
Pt	0.582102	-1.045903	-0.239570
N	1.439372	-2.595655	0.763827
H	-1.172282	0.321026	-1.601039
H	0.332886	0.769465	-2.114136
H	1.044226	-3.505785	0.556923
H	1.406898	-2.460448	1.786465
H	-0.269922	1.348933	-0.694595
H	2.435671	-2.633665	0.512283
C	-1.795350	-2.657239	0.362301
C	-2.895499	-3.572952	-0.107306
H	-1.172536	-3.148924	1.109221
H	-2.239959	-1.772181	0.817162
H	-2.478551	-4.549046	-0.368200
H	-3.329954	-3.148848	-1.023635
N	-3.853867	-3.728913	0.952794
H	-4.401798	-2.878446	1.012437
H	-4.485063	-4.493520	0.763934
C	-2.313024	2.944802	-0.402627
C	-2.973741	1.725630	0.181587
C	-3.137814	1.921886	1.665105
C	-1.749512	2.116651	2.228455
C	-0.934875	3.172988	1.514445
C	0.525460	3.032310	1.823535
N	-4.199432	1.404282	-0.506453
O	-3.725836	0.826985	2.289539
O	-1.017271	3.054678	0.109749
O	0.879536	1.731334	1.366338
S	-4.280145	-0.168046	-1.004880
O	-3.018807	-0.486665	-1.700175
O	-4.376398	-0.908489	0.258395
O	-5.445939	-0.239941	-1.857793
S	2.360746	1.406317	0.967194
O	3.177281	1.393770	2.158430
O	2.230060	0.064411	0.373431
O	2.765803	2.415908	-0.001128
H	-2.216826	2.853134	-1.485616
H	-2.275614	0.890433	0.057913
H	-3.755707	2.814894	1.832577
H	-1.239591	1.152039	2.149000
H	-1.261374	4.178159	1.804622
H	1.110780	3.781447	1.287662
H	0.715576	3.106476	2.893970
H	-4.453701	2.030574	-1.260674
H	-4.072313	0.199098	1.632786
H	-2.892673	3.849368	-0.181355
H	-1.803804	2.373259	3.288216
O	4.191928	-2.290454	0.151929
H	4.147418	-1.858522	-0.732818
H	4.800391	-3.026175	0.065358
O	1.023247	3.716334	-1.617486
H	1.727338	3.355874	-1.055165
H	0.224759	3.676171	-1.069912
O	1.162938	1.757443	-3.373406
H	0.773460	1.891400	-4.237003
H	1.074263	2.593907	-2.867388
O	1.867380	-2.040485	3.462361
H	1.942236	-2.713612	4.137967
H	2.779554	-1.737243	3.231688
O	4.681013	1.733962	-1.922978
H	4.452404	0.821568	-2.165132
H	4.039676	1.993745	-1.251542
O	4.138845	-1.129760	2.569713
H	4.224412	-1.484782	1.662981
H	3.920437	-0.190735	2.471262
Cl	3.994556	-1.209898	-2.64279
